# Individual honey bee tracking in a beehive environment using deep learning and Kalman filter

**DOI:** 10.1038/s41598-023-44718-y

**Published:** 2024-01-11

**Authors:** Panadda Kongsilp, Unchalisa Taetragool, Orawan Duangphakdee

**Affiliations:** 1 Department of Computer Engineering, King Mongkut’s University of Technology Thonburi, Bangkok, 10140 Thailand; 2 Native Honeybee and Pollinator Research Center, Ratchaburi Campus, King Mongkut’s University of Technology Thonburi, Rang Bua, Chom Bueng, Ratchaburi Thailand

**Keywords:** Zoology, Engineering

## Abstract

The honey bee is the most essential pollinator and a key contributor to the natural ecosystem. There are numerous ways for thousands of bees in a hive to communicate with one another. Individual trajectories and social interactions are thus complex behavioral features that can provide valuable information for an ecological study. To study honey bee behavior, the key challenges that have resulted from unreliable studies include complexity (high density of similar objects, small objects, and occlusion), the variety of background scenes, the dynamism of individual bee movements, and the similarity between the bee body and the background in the beehive. This study investigated the tracking of individual bees in a beehive environment using a deep learning approach and a Kalman filter. Detection of multiple bees and individual object segmentation were performed using Mask R-CNN with a ResNet-101 backbone network. Subsequently, the Kalman filter was employed for tracking multiple bees by tracking the body of each bee across a sequence of image frames. Three metrics were used to assess the proposed framework: mean average precision (mAP) for multiple-object detection and segmentation tasks, CLEAR MOT for multiple object tracking tasks, and MOTS for multiple object tracking and segmentation tasks. For CLEAR MOT and MOTS metrics, accuracy (MOTA and MOTSA) and precision (MOTP and MOTSP) are considered. By employing videos from a custom-designed observation beehive, recorded at a frame rate of 30 frames per second (fps) and utilizing a continuous frame rate of 10 fps as input data, our system displayed impressive performance. It yielded satisfactory outcomes for tasks involving segmentation and tracking of multiple instances of bee behavior. For the multiple-object segmentation task based on Mask R-CNN, we achieved a 0.85 mAP. For the multiple-object-tracking task with the Kalman filter, we achieved 77.48% MOTA, 79.79% MOTSP, and 79.56% recall. For the overall system for multiple-object tracking and segmentation tasks, we achieved 77.00% MOTSA, 75.60% MOTSP, and 80.30% recall.

## Introduction

Communication and interaction among social animals are crucial behaviors, especially for honey bees. In recent years, honey bee behaviors have received increased attention because this knowledge is useful to study and understand natural ecosystems^1–4^. According to a survey, besides being crucial pollinators, honey bees can be used as bio-indicators and ground surveyors for surveying, monitoring, reporting, and evaluating the health of the landscape by using their behavioral information (e.g., dancing)^5–7^.

In cavity nesting honey bees such as *Apis mellifera* and *A. cerana*, dancing bees land on vertical comb and perform the waggle, a figure-eight dance in which the bee waggles its abdomen. The dancer starts by running in a straight line and returns in a semicircle to the starting point, then again runs through the same straight-line portion and returns in a semicircle in the opposite direction^8^, creating a figure-eight pattern. On the straight part of the run, the bees perform wing buzzing and abdomen vibrating, the so-called waggle phase. The angle of the axis of this waggle phase relative to the vertical represents the angle of the target to the sun’s azimuth. This is the symbolic language that provides essential information by the dancers and decoding by the nest members. The liveliness of the waggle phase represents the richness of the food sources.

The traditional way to analyze behavior is by experts to observe and decode information manually, which is time-consuming, costly, not capable of analyzing a large group of bees, and limited to human knowledge^5,6^. During the past few decades, image-based methods have gained popularity because they appear to be a potential solution for various tasks, including animal tracking and behavior recognition as a result of the automated, non-disturbing, and cost-effective techniques. The combination of computer vision techniques and deep learning approaches is a powerful system that has received increased attention for both its academic and commercial potential in the study and management of bees^9,10^. However, the core and most difficult tasks the system has to manage are honey bee recognition and tracking.

To recognize a thousand bee behaviors in a beehive, three main tasks need to be considered: (1) a multiple-bee detection task for detecting and localizing the body of each bee with bounding box predictions in a hive frame area, (2) a multiple-bee segmentation task for cropping and extracting the body area of each bee depending on the shape of the bee’s body, and (3) tracking of multiple bees for extracting individual bee trajectories across continuous image frames.

Feldman and Balch^11^ were among the first research teams to apply image-based techniques for recognizing honey bee behavior, including dancers, followers (bees following a dancer), active bees, and inactive bees. For the detection and tracking tasks, they employed a manual tool to mark bees. Kernel regression was used to classify motions, such as arcing, waggling, moving, and loitering. Then, the sequence of motions was identified as behavior using a Hidden Markov Model (HMM). They achieved 79.8% accuracy in identifying these behaviors. The primary limitation of their approach was allowing only one behavior to be attributed to each individual bee, potentially leading to a reduction in the accuracy of the system. To deal with unmarked bees and automate detection, Kimura and team proposed a combination of Vector Quantization and temporal contextual information for honey bee detection and tracking tasks, respectively^12^. The study revealed a successful detection rate of 72% for individual bees, with approximately 350 bees (50% of those detected) being accurately tracked for 10 s. Yamanaka and Takeuchi^13^ also developed UMATracker, a software designed for animal tracking tasks. It offers users the flexibility to choose from multiple tracking algorithms, including RMOT^14^, Optical Flow DualTVL1^15^, K-means^16^, and GroupTracker^17^. However, this system requires manual input from the user to indicate each bee’s body position in the initial image frame. Additionally, it lacked evidence for dense object tracking.

Bozek et al.^18^ proposed a novel method of individual recognition and localization by integrating the FCN model with recurrent components for image-based dense object tracking. They aimed to automatically recognize all individual bees in bee hive environment, with expected outputs including bee body segmentation, prediction of bee body orientation, and trajectories of individual bees. For data labeling, their annotation class label consisted of two classes: full bee bodies and bee abdomens (when a partial body is hidden inside a comb cell). Their results demonstrated that deep learning approaches provided high accuracy and precision for dense object detection and segmentation in a complicated environment, such as a bee colony in a hive. Building on this work, Bozek et al.^19^ embarked on a subsequent study to address those limitations. For segmentation, their system could detect both bees and brood cells within the densely populated bee frame. To improve tracking accuracy, they combined Euclidean distance measurements for object positions across image frames with CNN-based analysis of visual features, including orientation, posture, and background information. Their experimental results indicated that 77% of detected bees were accurately tracked. Despite the improved accuracy, challenges persisted in the CNN training process for the tracking model.

For image processing using deep learning algorithms, data annotation is an essential task when training a supervised model^20^. Data annotation involves labeling or tagging the interesting features or object classes within each image frame. The expected output of this process is ground truth data, which deep learning models rely on to learn and predict objects. However, the need to annotate every object with a unique identifier (ID) across multiple image frames makes the annotation process for tasks like multiple-object tracking both time-consuming and costly. Consequently, data annotation remains a challenging problem, particularly for small and dense objects.

To overcome the data annotation step for tracking tasks, the Kalman filter is a potential approach. Bewley proposed a lean implementation of a tracking-by-detection framework for the problem of Multiple Object Tracking (MOT), which focused on frame-to-frame prediction and association, called SORT (Simple Online and Realtime Tracking)^21^. SORT combines a CNN-based approach, a Kalman filter, and the Hungarian algorithm for object detection and tracking. For object detection, the Faster R-CNN framework was employed to detect objects in each frame and represent them using bounding boxes. When a detection was associated with a target object, the Kalman filter framework together with a Hungarian algorithm predicted and updated the target state for each detected bounding box based on the optimal velocity. Referring to the MOTChallenge 2015^22^, SORT provided high tracking performance for both accuracy and speed. In terms of evaluation metrics, SORT achieved the highest Multiple Object Tracking Accuracy (MOTA) scores for the online system. The Kalman filter was also applied to small animal tracking problems in a dense environment. Xioyan and co-workers applied the Kalman filter to track multiple ants^23^. Their approach combined online training with an appearance model to recognize appearance variations in each object. This was followed by online tracking using a motion model. The motion model employed the Kalman filter to predict object motion states for real-time tracking. Their experimental results demonstrated the effectiveness of this multi-ant tracking approach using the Kalman filter, with 33.4% MOTA and 72.1% Multiple Object Tracking Precision (MOTP) in tasks involving small and dense object environments.

Over the recent years, numerous research efforts have focused on addressing the challenges of honey bee detection, tracking, and the integration of both tasks. Notably, the studies conducted by Bozek et al. in both 2018 and 2021 highlighted that deep learning approaches could offer substantial accuracy and precision for dense object detection and tracking within complex environments, such as the confined space of a bee colony within a hive. However, these two studies identified a couple of primary limitations. Firstly, the utilization of fixed shapes and sizes for bee body segmentation imposed a constraint on the research's scope. This limitation not only led to the oversight of bees with varying sizes but also impeded the ability to encompass a wide range of bee behaviors, including intricate joint movements and diverse postures, which are particularly relevant for activities such as bee dancing. Secondly, the utilization of CNN-based tracking required labor-intensive manual annotations for training the models, leading to inefficiencies. These research gaps motivated us to study and propose the combination of deep learning and the Kalman filter for multiple object segmentation to address the complexity of multiple bee segmentation and tracking. This includes challenges such as the high density of small members, dynamic individual bee behavior, occlusion, interaction behavior, and the similarity between the bee body and the background in a beehive. We employed Mask R-CNN^24^, a deep learning-based model that has the ability to detect and segment multiple objects in a flexible area instead of a fixed shape and size. The Kalman filter was used for each bee body tracking task to eliminate the need for data annotation during the model training step. Our study offers three notable contributions: (1) a framework for multi-object tracking tasks that do not require specific ID annotations, (2) the capability to handle the tracking and segmentation of multiple small objects within a complex and densely populated environment, and (3) a flexible output that predicts segmentation areas based on the actual positions of each bee’s body parts and postures.

## Methods

### Framework overview

**Figure 1:**
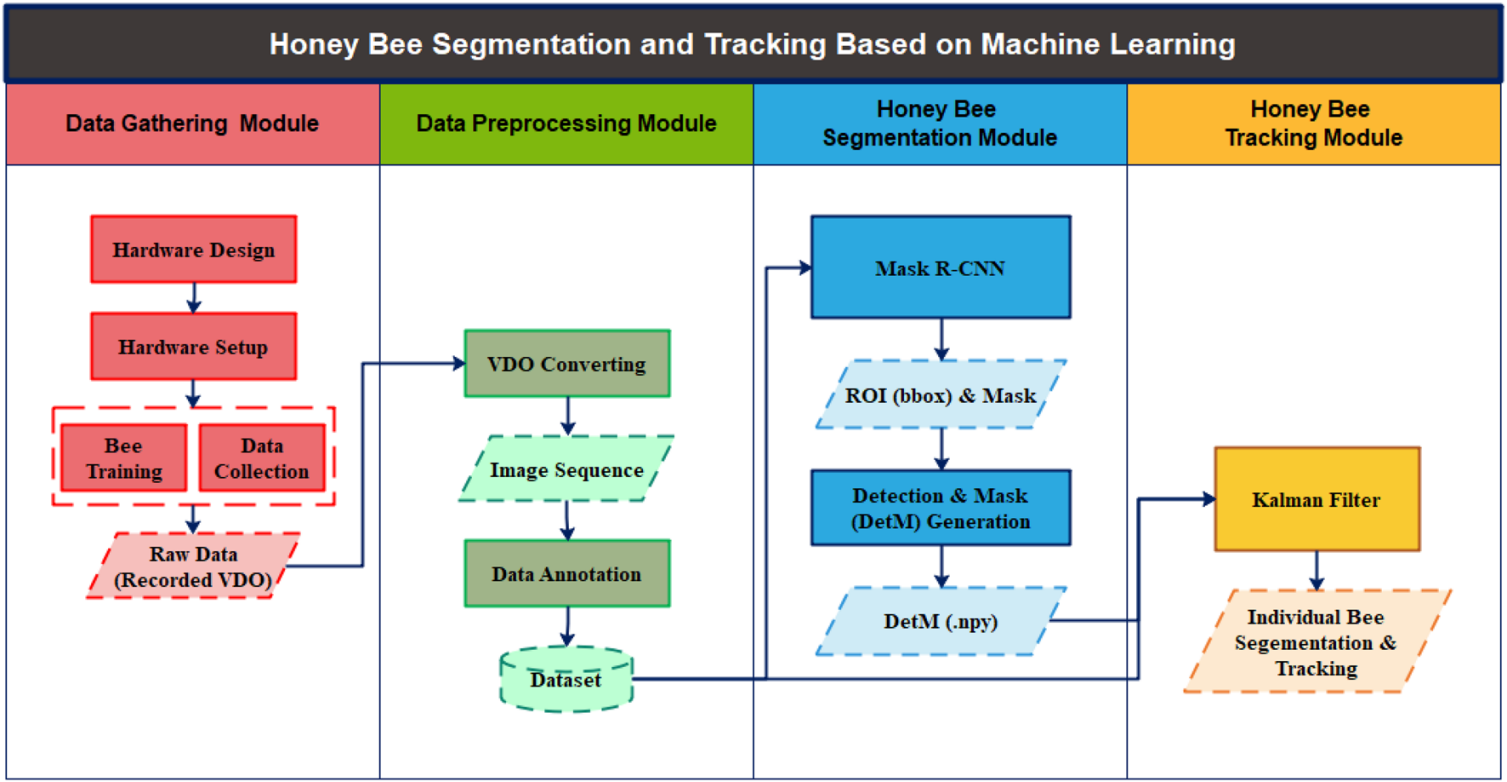
Research framework: honey bee segmentation and tracking.

In this study, we proposed the combination of a CNN-based approach and the Kalman filter for the segmentation and tracking of multiple bees within a beehive. As shown in Fig. 1, our proposed system comprised four main modules: a data gathering module, a data preparation and annotation module, a honey bee segmentation module, and a honey bee tracking module. The data gathering module involved fieldwork and included two main parts: hardware design and hardware setup. The output of this initial module was recorded as videos that served as the raw input data for the whole system. Then, the data preparation and annotation module was executed to create a dataset for model training and system evaluation. For the honey bee segmentation module, we employed Mask R-CNN featuring a ResNet-101 backbone. This architecture facilitated the extraction of region proposals for each object, representing them as bounding boxes (for detection) and predicting the mask area of each individual bee instance (for segmentation). The selection of an appropriate backbone for feature extraction plays a pivotal role in many CNN models, with options like ResNet^25^, VGG^26^, GoogLeNet^27^, and PreLU-net^28^ frequently utilized. Residual Neural Network (ResNet) has garnered significant attention due to its recurrent units positioned between convolutional and pooling layer blocks, making it ideal for image recognition, object detection, and segmentation tasks. Among the ResNet family versions—ResNet-34, ResNet-50, ResNet-101, and ResNet-152—ResNet-101 and ResNet-152 stand out for their enhanced accuracy^29^. However, the incremental gain in accuracy from ResNet-101 to ResNet-152 is not substantial^25^. Hence, we chose to construct our segmentation model based on Mask R-CNN utilizing a ResNet-101 backbone. Detection with a mask, called DetM, was the output from this module, which contained an object class as well as the detection with a bounding box and a mask area for the individual bee body for each image frame. The last step was the honey-bee-tracking module. The detected bounding box and mask area data from the previous module were used to update the target state where the velocity components were solved optimally using a Kalman filter framework with a Hungarian algorithm. The final output from our proposed system was individual bee tracking with a segmentation area for all bee bodies in a hive in an observation frame.

### Data-gathering module

#### Hardware design (observation hive)

**Figure 2:**
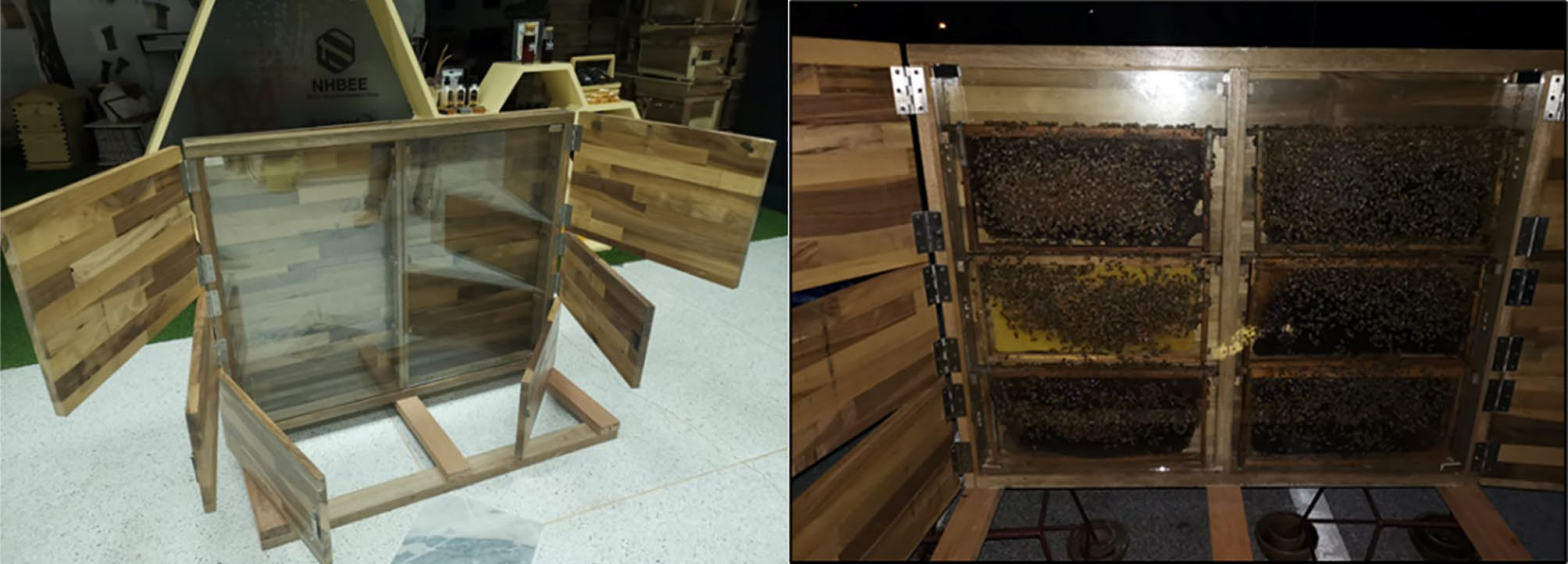
A customized observation hive.

To generate a dataset for our system, we designed and built a customized observation hive to collect video input data of honey bee behavior as closely as possible to their natural state. As shown in Fig. 2, the observation hive contained six hive frames. The total hive size was 1200 × 650 mm (width × height), and each frame size was 500 × 200 mm. The outer layer was made of hardwood to keep the hive dark and weatherproof, while the inner layer was made of transparent glass to allow observation of the behavior of the bees in the hive. We separated the outer door (non-transparent) to be able to individually open each hive frame. This way, we were able to open only the frame of interest and close other frames to keep the hive environment as dark as possible. This minimized disturbances to honey bee behavior and made bees in the colony feel comfortable and secure.

#### Hardware setup and environment

**Figure 3:**
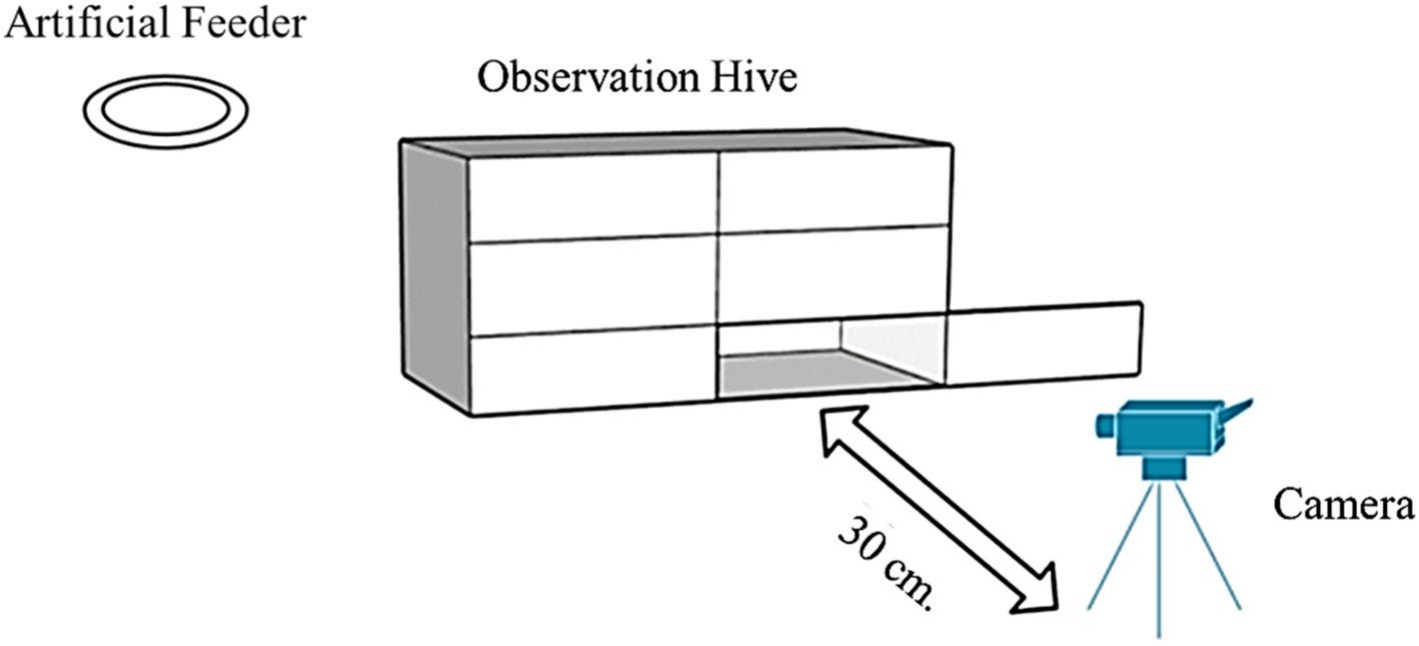
Recording hardware setup.

In this study, we set up the observation hive and collected input video data in the field near Bee Park, Native Honeybee and Pollinator Research Center, Ratchaburi Campus, King Mongkut’s University of Technology Thonburi (KMUTT), Ratchaburi, Thailand. We used a smartphone camera with a resolution of 1920 × 1080 pixels at a frame rate of 30 fps (frames per second) as a recording tool. We set the field of view (FOV) as 40 × 20 cm, which covered one hive frame area. As illustrated in Fig. 3, the camera was positioned behind the observation hive at the frame’s location, allowing for the observation of various movements and behaviors (e.g., dance behavior). To avoid interference from artificial light sources, we relied solely on natural light to illuminate the objects for the video-capturing task.

All video data were recorded during the summer (April 2021). Bees start their trip at sunrise and are active around 6 AM–6 PM. To ensure that all bee dances were caught on the videos, the collection process started at 7 AM and ended at 6 PM. We selected only one hive frame (the one on the bottom right position) to record the data because this frame was the closest to the nest entrances and contained a dense number of bees that exhibited various activities.

#### Data preparation and annotation module

In our experiments, we engaged with a small colony comprised of approximately 1000 bees (*A. mellifera*). As previously mentioned, the key challenges in this kind of research include the complexity, variety, and dynamism of individual bee behaviors, the interaction behavior, and the similarity between the bee body and the background in a beehive. Addressing the computational demands posed by the significant number of bees present in each frame, which also had implications for the data preparation and annotation resources, we conducted experiments aimed at reducing the frame rate of our original input data, initially captured at 30 fps. This initiative yielded three distinct input datasets, each characterized by varying frame rates—specifically, 5, 10, and 15 fps. The aim was to determine the most suitable frame rate for the image sequence or input data, thereby ensuring optimal performance aligned with our research conditions and the environment. Throughout all three configurations, a consistent set of image sequences, each spanning a duration of 10 s, formed the basis of our analysis. Table 1 showcases the specifics: 50 image sequences for 5 fps, 100 for 10 fps, and 150 for 15 fps. This approach facilitated a comprehensive investigation into the impact of frame rate on our research objectives.

**Table 1:** Experimental data preparation.

Data Characteristic	Mask R-CNN (Training)	Mask R-CNN + Kalman (Testing)
Image frame size	1920 × 1080 pixels	1920 × 1080 pixels
Number of images	Training Set: 35 frames	–
	Validate Set: 5 frames	
	Test Set:	
	rames (at frame rate 5 fps)	
	100 frames (at frame rate 10 fps)	
	150 frames (at frame rate 15 fps)	
Frame rate (fps)	–	5 fps, 10 fps, 15 fps
Sequence length	–	10 s/sequence
Number of image sequences	–	50 frames (at frame rate 5 fps)
		100 frames (at frame rate 10 fps)
		150 frames (at frame rate 15 fps)
Number of bees per image frame	≈ 370 bees	≈ 370 bees

After obtaining the video input data, data preparation and annotation were conducted. The first step was data preparation, where we extracted the recorded video file into a series of image frames. Each image frame contained 1920 × 1080 pixels to represent our FOV for one hive frame area (40 × 20 cm). The second step was data annotation. Within the framework of our system, we tackled three distinct tasks—multiple object segmentation (MOS), multiple object tracking (MOT), and multiple object tracking and segmentation (MOTS). To effectively carry out both the training and testing procedures, these tasks required the utilization of three corresponding data formats. Notably, these annotations were structured in alignment with the COCO format, which includes essential attributes such as image ID, image height, image width, class ID, object instance ID, and polygon area. To facilitate these annotations and ensure compliance with the COCO format, we chose to use CVAT, an interactive annotation tool designed for computer vision tasks. This versatile tool offers diverse annotation formats, including the COCO format, which seamlessly aligned with the requirements of our study^30^.

Class “b” was assigned to every single bee body in an image frame, which served as the only object in this study. Other environments and backgrounds were disregarded. However, we defined the data annotation rules for our system in accordance with the dense and occlusion situation in a hive frame, as follows:

1. When interference between two or more bee bodies (“occlusion”) was found, all bee bodies were annotated as full bee bodies. In the case of a missing part or when the area under the occlusion was found, we annotated data like a full bee body by estimating the missing area manually.
2. When we were unable to see the whole bee body (“imperfect bee;” for instance, when the head of the bee was sticking to the comb and the body of the bee body was at the boundary), we annotated and labeled the body “b” only when we could see more than 80% of it.
3. Because the observation hive was covered with glass, some bees climbed to the glass surface instead of the frames. In these cases, the bee’s belly would be visible, and we would also label the tip-over bees “b”.

Therefore, in the annotation process, objects that were considered and labeled “b” included the full bee body with the forward direction (a bee that faces the frame), partial bees with 80% of their body visible, occlusions, and tip-over bees. Other objects with different conditions were ignored.

**Figure 4:**
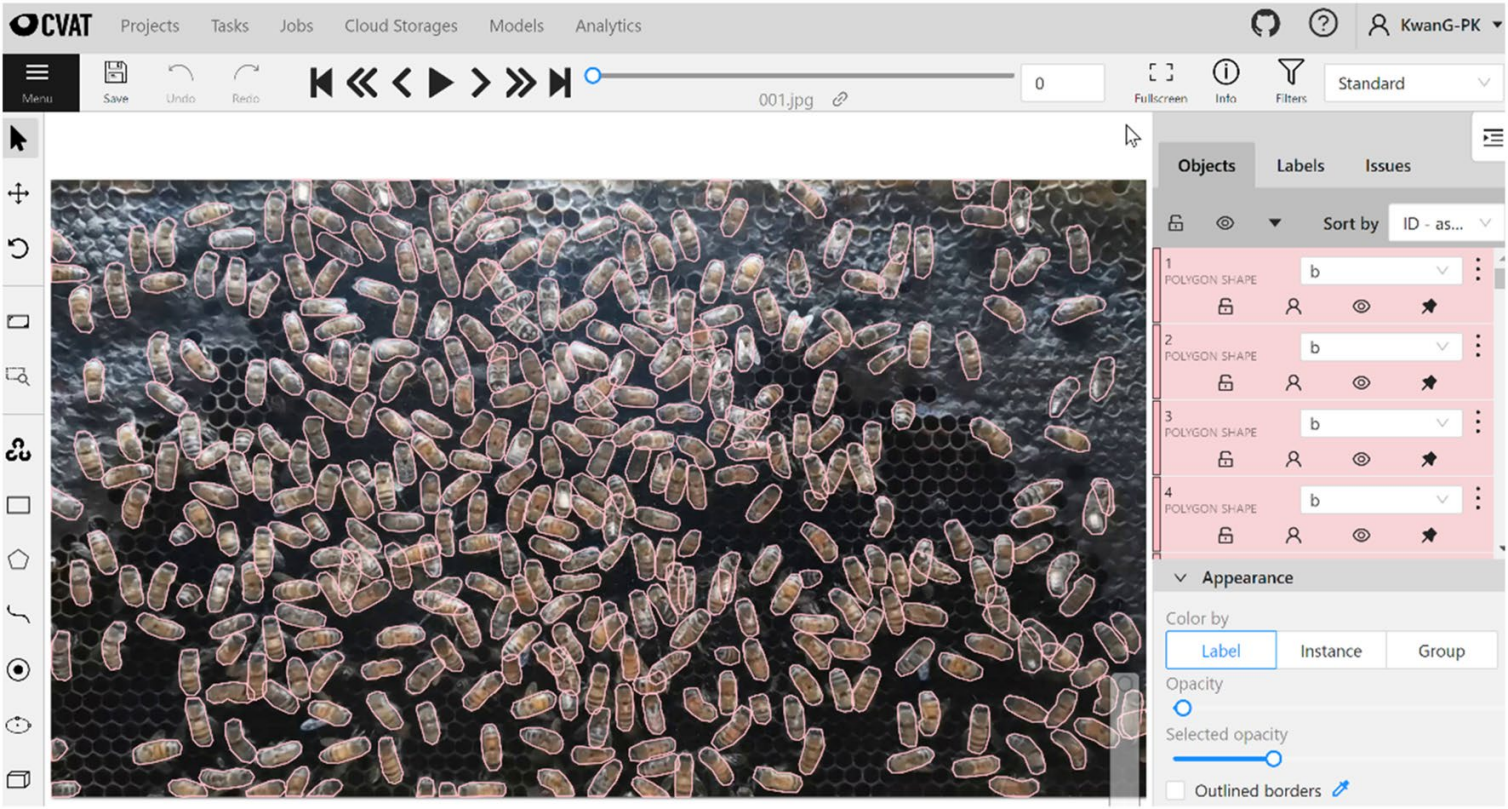
Data annotation (manual) for Mask R-CNN training using cvat.

**Figure 5:**
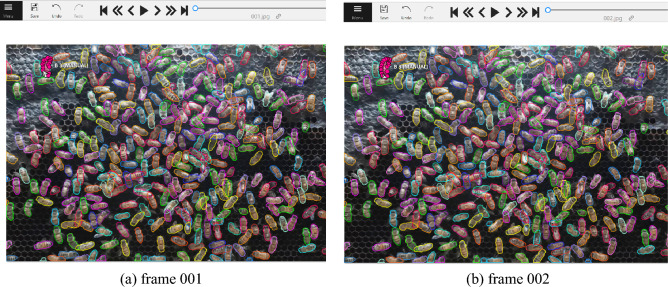
Data annotation “b3” (manual) for system evaluation using cvat.

The process of data annotation was split into two main parts for two distinct purposes: (1) data annotation for training the multiple object segmentation model, using Mask R-CNN, and (2) data annotation for the overall system evaluation. First, we annotated the data used to train the Mask R-CNN model. We selected a random sample of image frames and divided them into three sets: a training set, a validation set, and a test set. As shown in Fig. 4, each region of the bee body was manually defined and assigned the letter “b” based on the actual posture of the bee within the frame. The information on the polygon area, identity, and trajectory of each bee body in the image frame was then included in the exported file. Second, data annotation was performed for the entire system evaluation, covering multiple object segmentation (MOS) based on the polygon area format, multiple object tracking (MOT) with object instance ID across the frames, and multiple object tracking and segmentation (MOTS). To annotate the test set for all three aspects simultaneously, each region of the bee body or polygon was manually defined with object instance ID across the continuous frames, and each annotation polygon was labeled “b with an ID.” As seen in Fig. 5a,b, frame 001 and frame 002 are examples of continuous frames where the same object is annotated with the same polygon area and a specific ID (“b3”) in each frame.

Table 1 summarizes the data preparation for our experiment. We annotated 40 image frames with polygon areas for Mask R-CNN training and 300 frames with polygon areas and individual tracking IDs across continuous frames for the overall system evaluation.

### Honey bee segmentation module

#### Model operation

Mask R-CNN has two main states, according to the original model. First, the region proposal network employs the FCN model to propose candidate objects. Second, the prediction state returns three predicted outputs: an object-bounding box to indicate the position of each bee body, an instance mask polygon for each ROI (region of interest) to indicate the area of each bee body, and the class ID “b.” These Mask R-CNN outputs are referred to as DetM (detection with mask) in this study and were used to generate each bee sequence across the image frame in the following honey-bee-tracking module (Fig. 1).

#### Model configuration and implementation

To configure and implement our core model for honey bee segmentation, which is Mask R-CNN, we utilized the feature pyramid network (FPN) and a ResNet101 backbone concept from the Matterport Mask R-CNN repository^31^. To build deep learning models, we then needed a high-level library, and Keras^32^ proved to be an ideal choice. Keras is an open-source high-level library for deep learning model implementation that offers a user-friendly interface. However, to operate Keras at a low level, we required a specialized tensor manipulation library known as a backend engine. Currently, there are two backends that support Keras: the TensorFlow backend (an open-source tensor framework developed by Google) and the Theano backend (an open-source tensor framework developed by LISA/MILA Lab). Since our system was developed and operated in the Colab environment developed by Google, we thus chose to implement our Mask R-CNN model using the Keras framework with the TensorFlow backend. This implementation was performed using Python 3.7, Keras 2.2.4, and TensorFlow 1.15.

#### Training and testing

During the training process, we used 35 images of the training set and 5 images of the validation set. As previously mentioned, our model was trained on Colab, specifically using Colab Pro+ with access to the NVIDIA Tesla V100-PCI-E-16GB GPU, which is the highest GPU provided by the Colab Pro+ package. We trained our model for 100 epochs. To optimize the hyperparameters for the Mask R-CNN model, we conducted a series of experiments. We found that training the model using our dataset without any pre-trained weights led to increased accuracy and was suitable for our specific conditions. After fine-tuning the hyperparameters under various scenarios, the optimized model hyperparameters for our settings and dataset were as follows:

- Minimum probability value to accept a detected instance = 0.7
- Learning rate = 0.001
- Learning momentum = 0.9
- Non-max suppression threshold to filter RPN proposals = 0.7
- Number of ROIs (Region of Interest) kept after non-maximum suppression for training = 6000
- Number of ROIs kept after non-maximum suppression for prediction = 3000
- Percent of positive ROIs used to train classifier/mask heads = 0.5
- Number of ROIs per image to feed to the classifier/mask heads = 1000
- Maximum number of ground truth instances to use in one image = 1000
- Maximum number of detected bees in each frame = 1000

For the testing process, we performed model predictions on Google Cloud using an n1-highmem-16 compute engine featuring a 16-core CPU and 105 GB of RAM. These computational system specifications were found to be adequate for conducting our evaluations, as determined through our experiments.

### Honey-bee-tracking module

The honey-bee-tracking module was designed to associate the bee bodies detected across continuous frames. Inspired by SORT, we adopted the Kalman filter and the Hungarian algorithm to deal with motion prediction and data association. In the initial state, all the data in DetM, which was the output from the previous segmentation module, were used as the input data for the Kalman filter. First, the state estimation model was used. The inter-frame displacement of individual bee bodies was approximated by a linear constant velocity model. For the state of the individual target, we followed the formulation of the original paper^21^ with the dimensional space including the bounding-box center point (horizontal and vertical pixel) of the target, scale, area, and aspect ratio. The original Kalman filter framework with a constant velocity motion and linear observation model was used to update the target state by determining the optimal velocity of the detected bounding box for each detected bee. Second, data association was performed. The assignment cost was computed as the intersection over union (IoU) between each detection and all predicted locations of the existing target. The assignment problem was then constructed for assigning detections to existing targets while minimizing assignment costs and was solved using the Hungarian algorithm to find the optimal solution. The minimum IoU (IoU_min_) in this study was set at 0.5 (50%). The bee track was assigned if the overlap between the detection and the target was greater than the IoU_min_. Finally, the track creation and deletion processes were executed. These processes manage the unique identity in each frame when new objects enter the frame or existing objects move out of the frame to monitor the number of bee tracks. A new track is created for each detection of an untracked bee when the overlap distance between the detection and the target is less than the IoU_min_ value. A bee track is terminated if it is not detected for T_Lost_, which is the threshold for the missing time of an object. Each deleted track that reappears in the frame is considered a new track.

### Evaluation metrics

**Figure 6:**
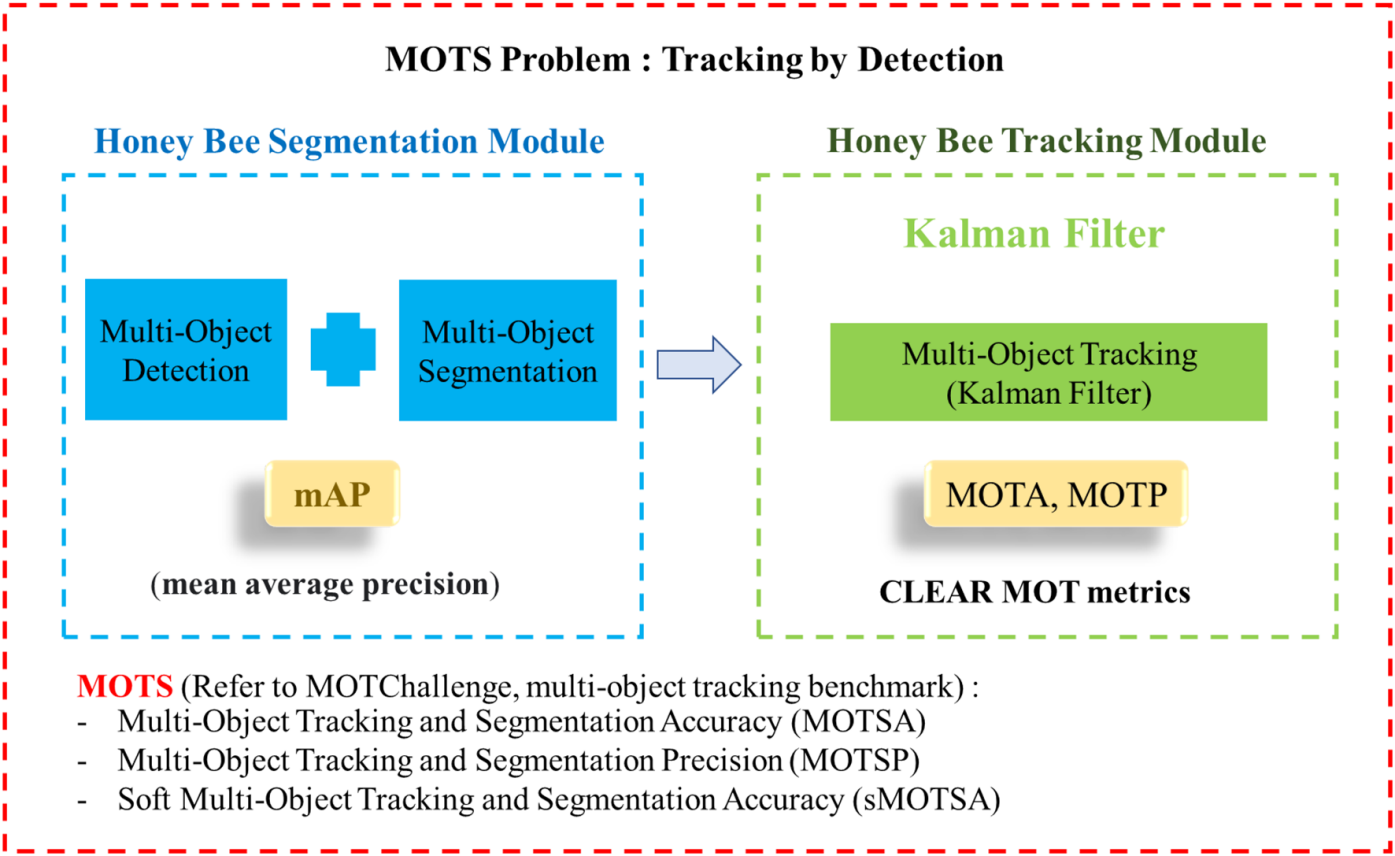
Evaluation metrics for tracking and segmentation of multiple bees.

Our core system is the combination of two main methods: Mask R-CNN for the detection and segmentation tasks and the Kalman filter for tracking tasks. We evaluated the proposed system for both individual performance (Mask R-CNN for multi-object detection and segmentation and the Kalman filter for multi-object tracking), and whole system performance (multi-object tracking and segmentation), as shown in Fig. 6.

For evaluating the Mask R-CNN model’s performance, we used the mean average precision (mAP), a widely-used metric for the instance segmentation task. To calculate mAP, we computed several related values including IoU, precision, and recall. IoU measures the overlap between predicted and ground truth segmentation masks and is calculated by dividing the intersection area by the union area of the predicted and ground truth masks (Eq. 1). IoU varies from 0 to 1, where a value of 1 indicates a perfect match, and 0 indicates no overlap. In our evaluation, a prediction was considered correct if the IoU was greater than or equal to 0.5 (IoU threshold = 0.5).

1 $$IoU= \frac{Intersection\, Area}{Union\, Area}$$

**Figure 7:**
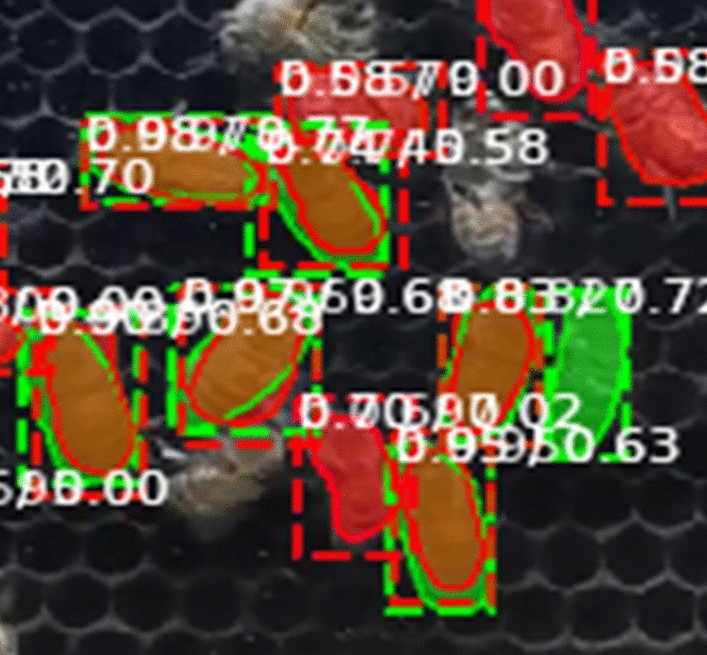
Four cases of comparison between the segmented objects in the prediction and the ground truth.

Furthermore, we categorized the comparison between segmented objects in predictions and ground truth into four cases (Fig. 7):

- *True positives (TP)* Pixels that exist in both the prediction and ground truth.
- *True negatives (TN)* Pixels outside the object in both the prediction and the ground truth.
- *False positives (FP)* Pixels that were present in the prediction but not in the ground truth.
- *False negatives (FN)* Pixels in the ground truth that were not predicted.

In Fig. 7, the color green represents FN, the color red represents FP, the color orange (the intersection or combination of green and red) represents TP when IoU ≥ 0.5, and the remaining area or background represents TN.

Precision represents the percentage of true positive predictions from all positive cases, while recall represents the percentage of true positive predictions from all predictions. Precision and recall values are plotted to generate the precision-recall curve. The area under the curve is measured as the average precision (AP), which can range from 0 to 1. An AP of 1 indicates the model has the highest precision and predicts results perfectly. Additionally, the averaged AP over all the object categories is referred to as the mean average precision (mAP). Since our model had only one class (“bee body”), mAP had the same value and meaning as the AP.

The CLEAR MOT metrics^33^ based on the MOT Challenge benchmark for the multiple object tracking problem^34^ were employed to evaluate the performance of the multiple object tracking task. The two most critical metrics in this evaluation were MOTA and MOTP.

In addition, we employed the evaluation process and tool from MOTS^35^, which relies on the CLEAR MOT metrics, to assess the overall system for honey bee tracking and segmentation. The three main matrices under consideration were multiple object tracking and segmentation accuracy (MOTSA), multiple object tracking and segmentation precision (MOTSP), and recall.

## Experimental results and discussion

### Data-gathering module

The biggest challenge in studying honey bee behavior in their natural environment is fieldwork. Hardware design and data collection are essential processes for the data-gathering module. We designed and built a customized observation hive to minimize disturbances. As shown in Figs. 2 and 3, the final hive design has only two rows (hive frames) to minimize the height. Moreover, the outer door (non-transparent) can be opened individually for each hive frame to minimize the penetration of ambient light into other areas of the hive. However, we still found some problems with the data collection process. Honey bees feel safe and comfortable in a vertical comb and in a dark environment^36^. Nevertheless, some light is needed for the video-capturing process. Thus, the optimized solution for data collection should not disturb honey bee behavior, which is still a key challenge for this kind of research. Instead of a traditional camera, we suggested that another camera technology such as an infrared camera or thermal imager may be an opportunity to overcome this issue.

### Data preprocessing module

Data annotation plays an important role in training the Mask R-CNN model for segmentation tasks and in conducting the overall performance evaluation. As previously mentioned, we lacked an existing dataset for honey bee tracking and segmentation tasks. In this study, we collected the input video data and also annotated it to create a honey bee dataset. Our dataset was built on the COCO format that could be applied for segmentation and tracking tasks. However, due to resource constraints, our capacity for annotating a large dataset was limited. Expanding both the quantity and diversity of input video data, including various observation hives, bee colonies, and honey bee species, holds the potential to enhance generalization and boost the system's overall performance. Additionally, our annotation process encountered specific challenges related to small objects and occlusions. Since the annotation process relies on human knowledge and skill, creating a clean and clear annotation for training and evaluation requires the establishment of annotation rules that must be strictly followed.

### Honey bee segmentation module based on mask R-CNN

Due to the characteristics of our problem, which involve small, similar objects in densely occluded situations, high resolution input data is required to cover details for each object area or individual bee body. Based on our collected data, each image frame contained approximately 370 samples (bee bodies). The combination of high image resolution and a large number of objects in each frame affects both the training and testing phases. The more complex the model, the higher the system or server specifications required to run it. Another factor that we need to consider is the compatibility between the segmentation module and the tracking module. Moreover, the accuracy of individual bee tracking is influenced by the performance of detection and segmentation. To address this situation, we optimized the model to meet the requirements by applying a trade-off between accuracy and the limitations of the computing system. We experimented with different image sizes and determined that the optimal image size for Mask R-CNN, which provided high accuracy and precision, was 512 × 512. Therefore, we downscaled the images from 1920 × 1080 to 512 × 512 for the Mask R-CNN process. Then, we reshaped the images from a rectangular to a square format by adding zeros to the outer areas. This square format helped us avoid complexity and misalignment problems when using the Kalman filter for tracking tasks. Finally, we upscaled the images back to their original size at the end of the process.

**Figure 8:**
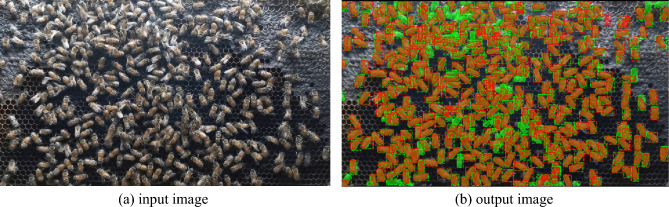
Detection and segmentation of multiple bees based on Mask R-CNN: predictions versus ground truth.

As shown in Table 2, the mAP of the instance segmentation performance of Mask R-CNN on the training set and validation set was 0.91 and 0.84, respectively. We conducted tests on three datasets with varying frame rates of input data. The mAP at frame rates of 5 fps, 10 fps, and 15 fps was 0.86, 0.85, and 0.83, respectively. These results suggest that the frame rate has only a minor effect on the multiple object segmentation task using the Mask R-CNN model. Figure 8a shows the input image frame, while Fig. 8b illustrates predicted output image for multiple object segmentation using Mask R-CNN. Each bee’s bounding box is represented by a dotted line, and a mask is shown as a solid polygon. In this visualization, green represents the ground truth (manual annotation), and red represents the prediction (output). The areas where green and red blend together (appearing orange) indicate overlap between the ground truth and the prediction, signifying correct or true positive predictions.

**Table 2:** Performance evaluation of the honey bee tracking and segmentation system.

Dataset	Evaluation matrix	Frame rate		
		5 fps	10 fps	15 fps
Mask R-CNN (segmentation)	Training set (mAP)	0.910		
	Validation set (mAP)	0.84		
	Test set (mAP)	0.86	0.85	0.86
Kalman (tracking)	MOTA (%)	75.85	77.48	77.66
	MOTP (%)	78.61	79.79	77.97
	Recall (%)	78.50	79.56	79.34
Full system (tracking and segmentation)	MOTSA (%)	75.40	77.00	77.70
	MOTSP (%)	74.00	75.60	72.20
	Recall (%)	81.90	80.30	79.60

### Honey-bee-tracking module based on the Kalman filter

**Figure 9:**
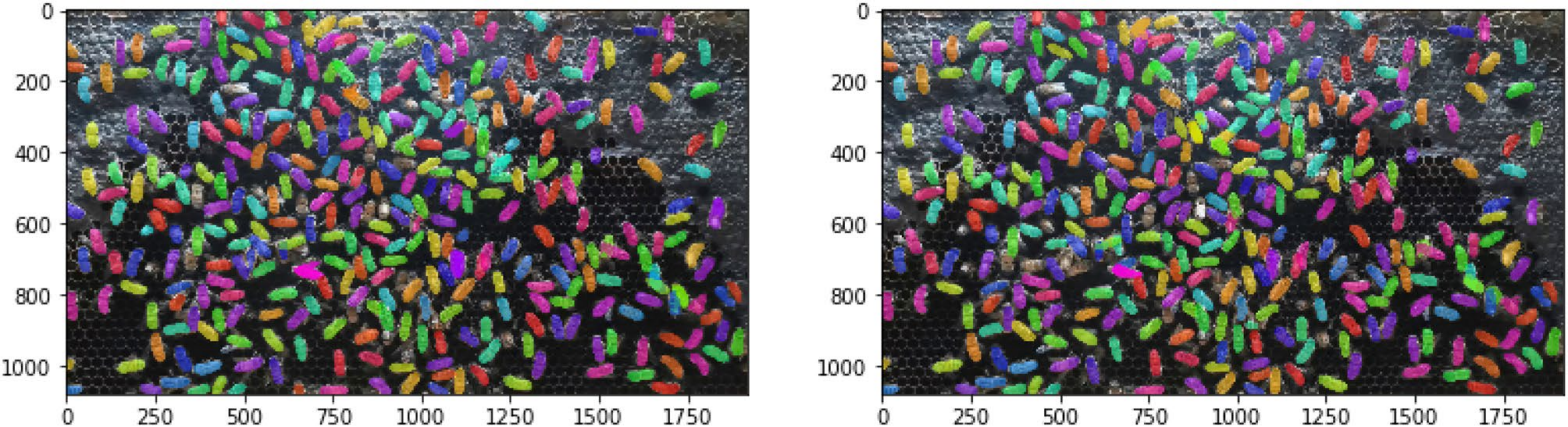
Kalman filter prediction results: the illustration of two continuous frames.

As previously described, we utilized the output from Mask R-CNN, which included the bounding-box positions and segmentation areas, as the input data for the Kalman filter. For the evaluation of multiple object tracking tasks, we employed TrackEval^37^, an open-source toolkit that offers several tracking evaluation metrics, such as the CLEAR MOT metrics (e.g., MOTA, MOTP, etc.). Referring to the data annotation process, we annotated individual bee positions and identities in each continuous frame, serving as ground truth for comparison with the predictions generated by our tracking module. Subsequently, we calculated the accuracy and precision of the trajectories or tracking steps for each object. Finally, MOTA (accuracy) and MOTP (precision) scores were computed for all trajectories throughout the video sequence, which spanned 10 s. Figure 9 displays the results of multiple object tracking based on the Kalman filter using two consecutive frames. Each polygon area represents the segmentation of an individual object, and the color of each polygon area corresponds to the instance ID assigned to each object by the Kalman filter. The results from our tracking module revealed that a frame rate of 15 fps yielded the highest tracking accuracy, reaching 77.66% (as shown in Table 2).

### Honey bee tracking and segmentation system

In Table 2, it is evident that a frame rate of 15 fps provided the highest accuracy at 77.70%, while the accuracy at the frame rate of 10 fps was approximately 77.00%. Moreover, our model achieved its highest precision (75.60%) at a frame rate of 10 fps. A higher accuracy reflects more accurate predictions, and a high precision value obtained here indicates that our model is proficient at predicting positive cases. However, it is important to note that a higher frame rate requires more resources to run the system. The accuracy gap between 10 and 15 fps was approximately 0.70%, but this increase in fps required a resource upgrade and about 33.33% more time to execute the system. Hence, as a trade-off between system performance and required resources, we concluded that input video data at a frame rate of 10 fps was adequate to capture bee behavior in a beehive and was appropriate for addressing the honey bee tracking and segmentation problem.

When comparing the performance of several online trackers based on two key perspectives, accuracy and speed, SORT, as presented by Bewley, achieved accuracy comparable to state-of-the-art trackers and was faster than most other trackers. However, this system only focuses on tracking tasks that require manual bounding box annotation in the first image frame for each object to track their positions across continuous frames. SORT is unable to automatically recognize, detect, and track new objects in subsequent frames. In contrast, our system has been designed to address the complexity of multiple bee segmentation and tracking tasks automatically, eliminating the need for manual intervention (Supplementary Information).

### Honey bee trajectory

**Figure 10:**
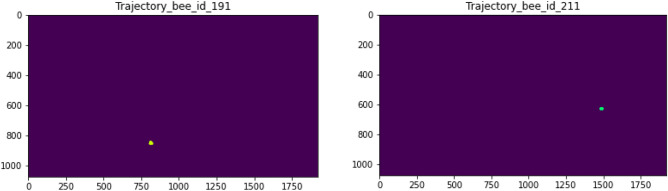
An example of inactive bee trajectories at a frame rate of 10 fps.

**Figure 11:**
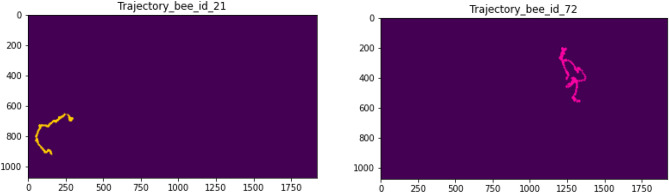
An example of active bee trajectories at a frame rate of 10 fps.

**Figure 12:**
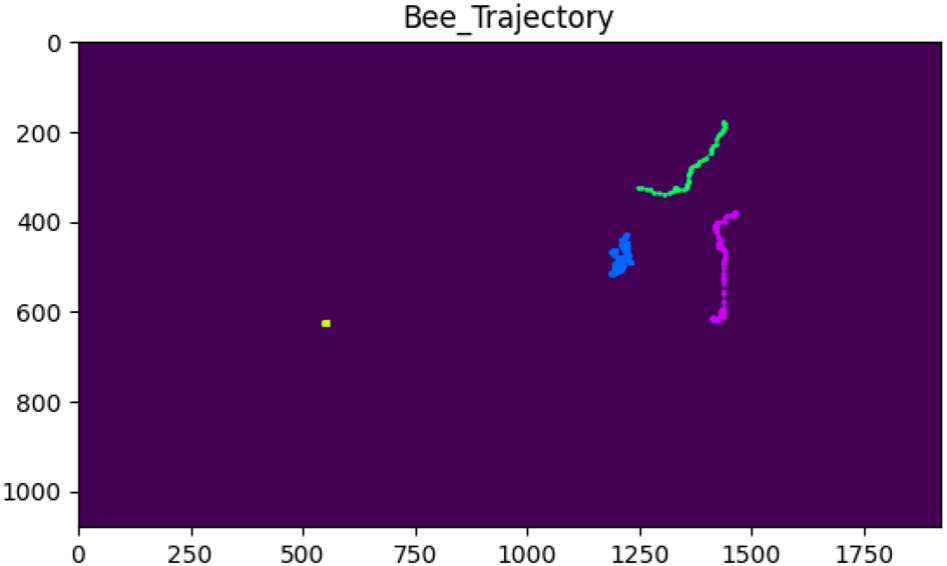
An example of bee trajectories with the various behaviors at a frame rate of 10 fps.

**Figure 13:**
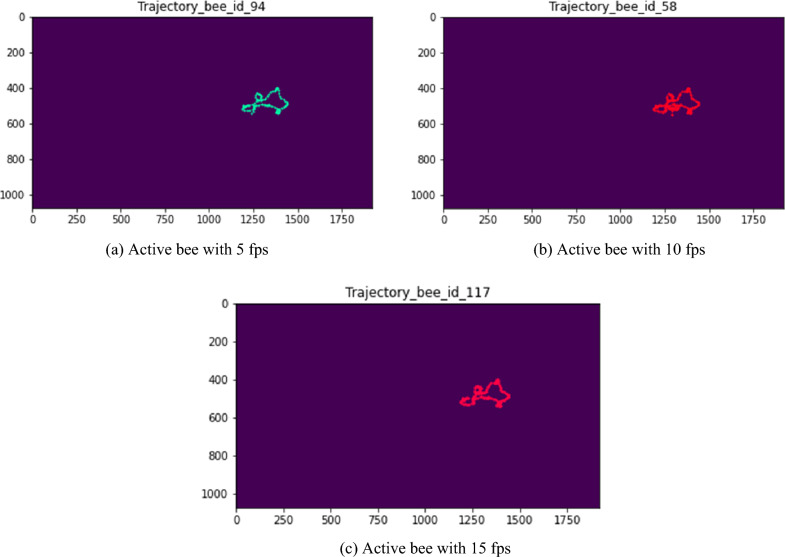
Comparison of trajectories for the same active bees at different frame rates (5 fps, 10 fps, and 15 fps).

With our proposed system, individual bee tracking can be represented as trajectories that illustrate the bees’ paths over a 10-second period. We categorized each bee in a hive into two main groups based on their behavior: inactive bees and active bees. For the inactive bees, which did not exhibit any movement or trajectory, we were unable to recognize any trajectory for them. As shown in Fig. 10, a very short line illustrates the slightly different positions of the bees between consecutive frames, indicating that the inactive bees were in a resting mode. For the active bees (Fig. 11), we extracted their movement trajectories to observe their paths around the hive frame. The length of the trajectory is related to the velocity of the movement during the same period. Fig. 12 shows the individual bee trajectories predicted from our system, capturing various types of behavior. As described, we compared all the individual predicted bee trajectories with manually labeled (ground truth) bee trajectories to calculate MOTA and MOTP based on the CLEAR MOT metric. In terms of video frame rate, Fig. 13 illustrates the comparison of the final output between different input frame rates for the same individual active bee. We noticed slight differences in the trajectory outputs between 5 fps, 10 fps, and 15 fps.

### Future work

**Figure 14:**
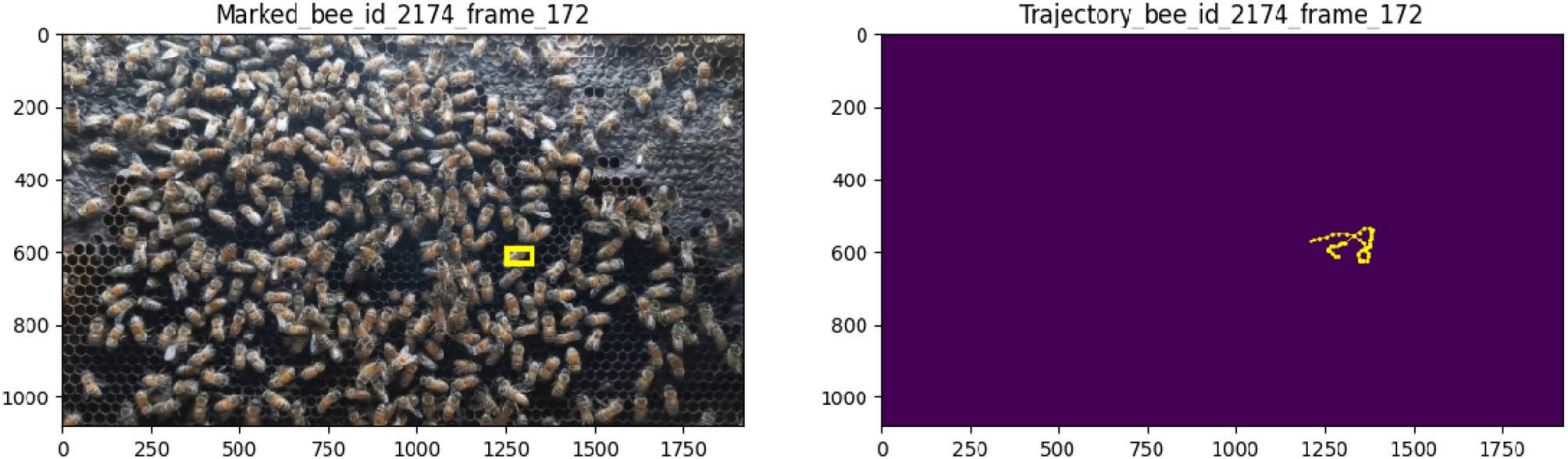
Example of bee behavior trajectory.

The literature survey revealed that honey bee dance behavior provides information about the location of a food source (both direction and distance), not only for other bees but also for humans^38^. Besides pollination, many studies have used bee dance behavior as a bio-indicator and ground surveyor for surveying, monitoring, reporting, and evaluating the health of the landscape. Bee dance studies began with an understanding of how the physiology and anatomy of the animal integrate with its behavior. We, thus, believe that we can extend our output data for individual bee tracking with a mask to achieve automated honey bee dance pattern recognition. As seen in the videos provided in the supplementary material, our system has the ability to track the walking paths or trajectories of honey bees while they perform dance behavior. The extracted data from these trajectories serves as the primary input data for behavior recognition. Moreover, to validate this idea, we collaborated with specialists in the field of bee behavior from Bee Park, Native Honeybee Research Laboratory, and KMUTT Ratchaburi campus. These experts identified honey bee bodies with a dance pattern found in a hive frame from our input video data. We then compared individual dancing bees to the trajectories obtained by our system. Figure 14 shows an example of the bee behavior trajectory labeled as dance behavior. We noticed some trajectory patterns from dancing bees that were different from those of other active bees in the hive. Bees repeat the dance pattern in a loop in a small area. Some dancers perform a circular motion called the round dance, while others may perform a more complex dance pattern by walking and shaking their bodies, known as the waggle dance. To recognize honey bee dance patterns or any other behaviors, honey bee trajectory information is crucial for further studies.

## Conclusion

In this work, we present an automatic system for multiple-object tracking and segmentation, based on Mask R-CNN and the Kalman filter. Our proposed system aims to handle small and densely packed objects in complex environments, such as a honey bee colony within a beehive. There are three main advantages to this work. First, our proposed system can distinguish small and dense objects within complex backgrounds, such as cells containing brood, pollen, and nectar, while also differentiating between honey bees and the honeycomb. It is also capable of tracking multiple honey bees in dense and occluded situations. Second, we employed an annotation-free framework for multi object tracking tasks. The Kalman filter is a suitable method for addressing multiple-object tracking within our problem statement and environment. Unlike a supervised deep learning-based approach, which requires both object position (bounding box) and instance-ID ground truth for training, the Kalman filter only requires a bounding box from the detection model to determine the object's position. It is a simple yet high-performance method. Third, our system provides predicted segmentation areas with free-moving joints for the head, thorax, and abdomen, based on the true position of each body part and its posture. This flexibility can be valuable for further studying bee dancing language, a crucial behavior among bees. At a frame rate of 10 fps (optimal for our situation), we achieved 77.00% MOTSA, 75.60% MOTSP, and 80.30% recall for the entire system. This performance evaluation demonstrates that Mask R-CNN (a deep learning method for multiple-object segmentation tasks) and the Kalman filter (the core method for multiple-object tracking) exhibit high performance and yield acceptable results for tracking and instance segmentation tasks. In conclusion, the combination of Mask R-CNN and the Kalman filter is an effective approach for tracking and segmenting multiple bees under natural conditions. Furthermore, our trajectory results indicate the potential to extend our findings to other bee behaviors, such as honey bee dance pattern recognition. In reference to honey bee behavior in their natural life, the most complex and high-speed motion is the dance behavior, especially the waggle dance. Each dance pattern consists of two phases: the waggle phase and the return phase^39^. During the waggle phase, the dancer shakes her abdomen, waving her body from side to side at a frequency of about 13 Hz while moving in a specific direction before returning to the starting point. The waggle dance is performed in multiple cycles, depending on the quality of the food source. To extend our system to recognize and analyze bee dance behavior, the frame rate of the extracted image frames should cover the highest frequency (13 Hz). We recommend using a frame rate of 15 fps for proper honey bee behavior recognition.

### Supplementary Information

Supplementary Information.

## Data Availability

All data generated or analysed during this study are included in this published article [and its supplementary information files]. The related datasets used and/or analysed during the current study available from the corresponding author on reasonable request.
