# Supplementary figures and images for: Individual honey bee tracking in a beehive environment using deep learning and Kalman filter (part 2 of 2)
Source: Sci Rep. 2024 Jan 11;14:1061. doi: 10.1038/s41598-023-44718-y (PMC10784501; doi:10.1038/s41598-023-44718-y)

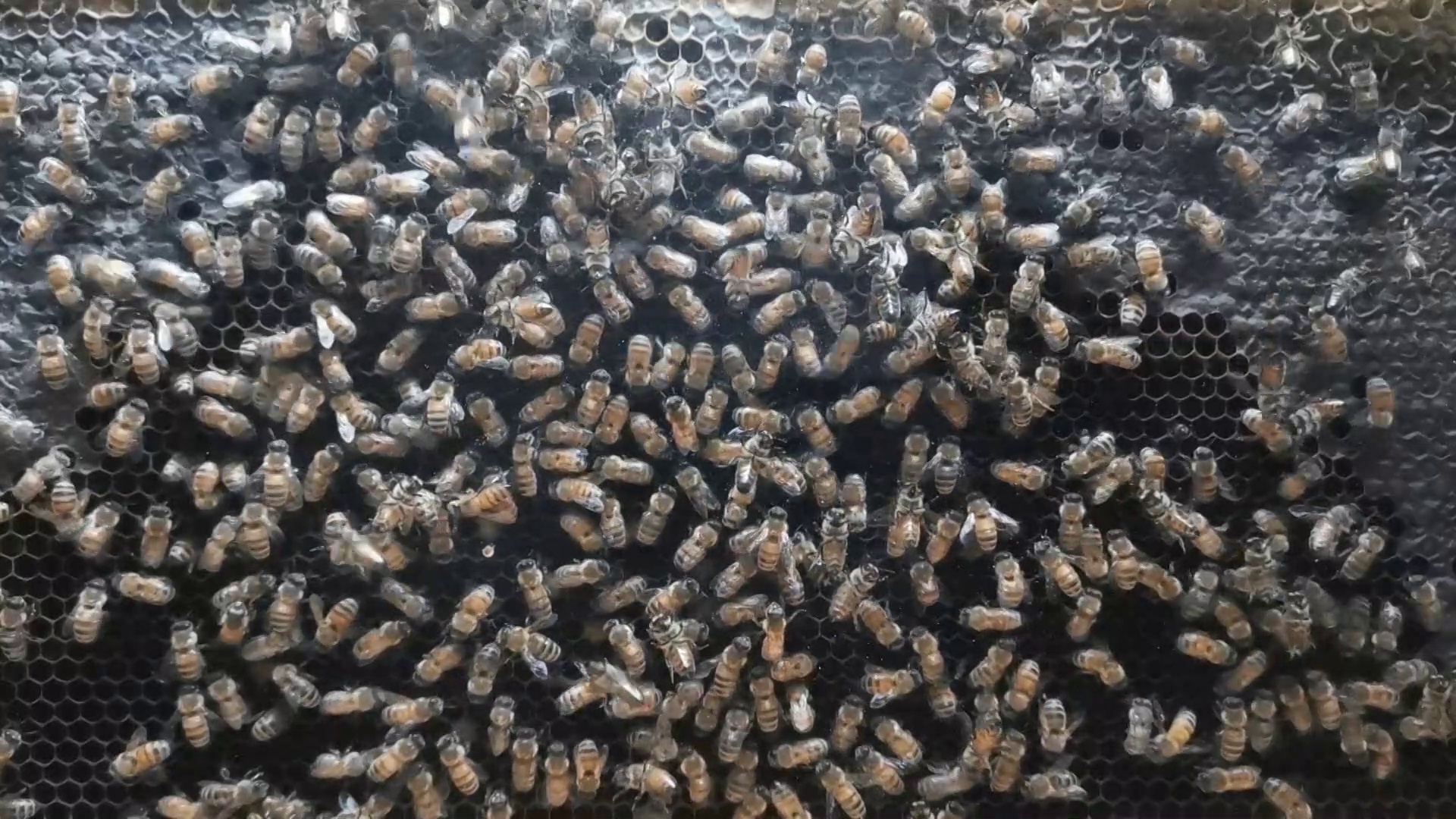

Supplement: Supplementary file 1 — Supplementary Information. [file 41598_2023_44718_MOESM1_ESM.zip › Dataset/test set-system_evaluation/test_set_15fps/070.jpg]

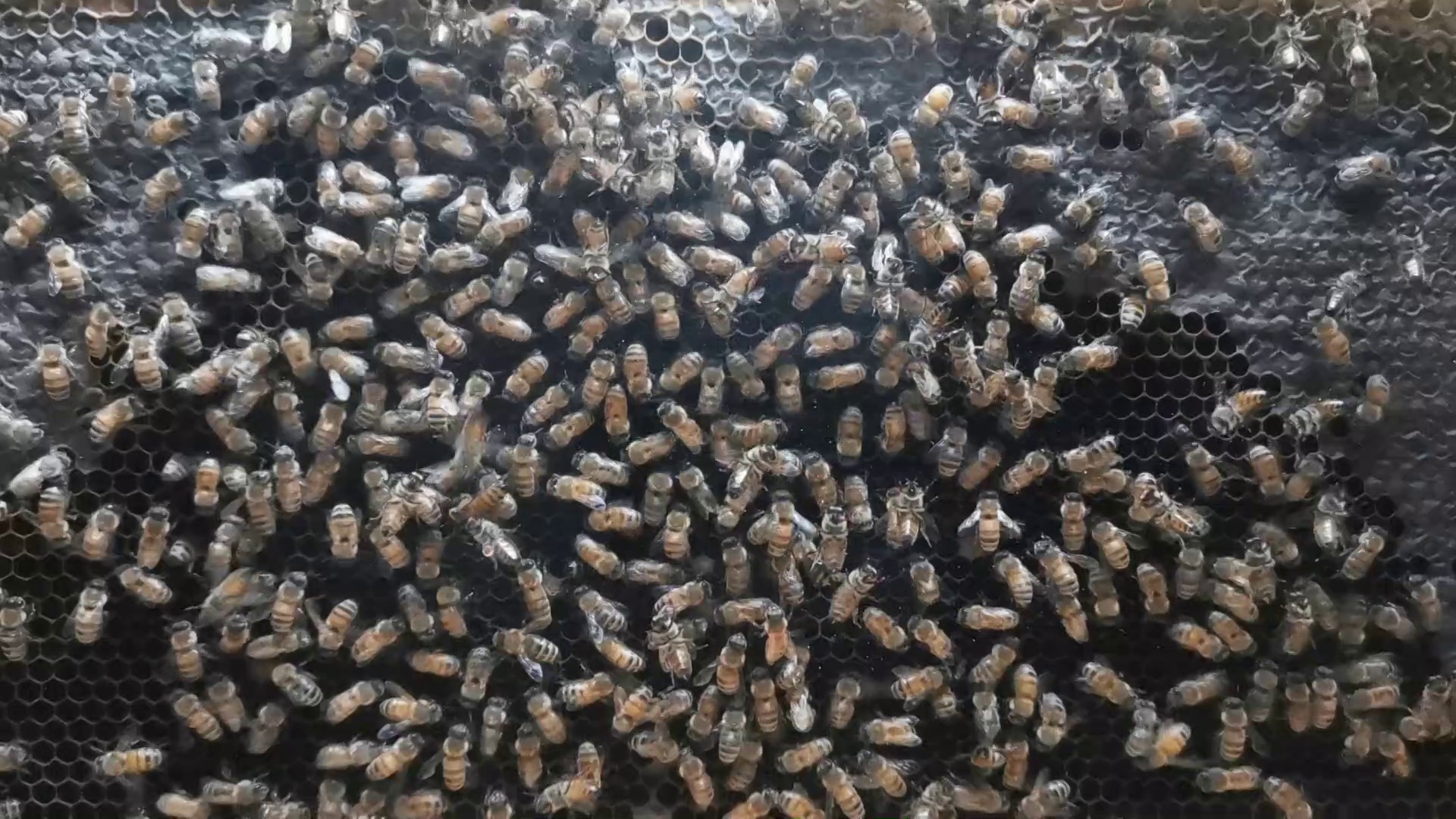

Supplement: Supplementary file 1 — Supplementary Information. [file 41598_2023_44718_MOESM1_ESM.zip › Dataset/test set-system_evaluation/test_set_15fps/088.jpg]

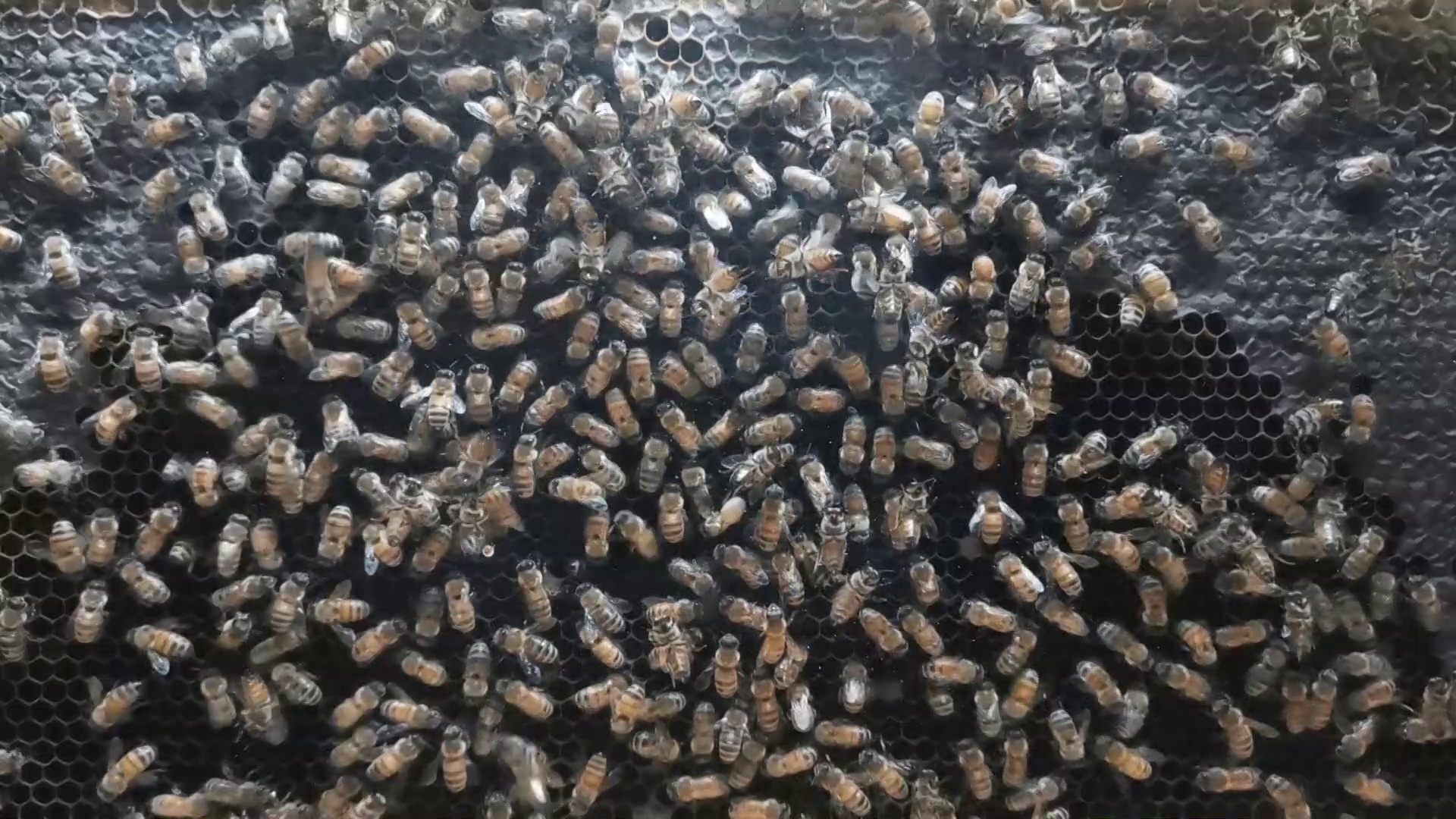

Supplement: Supplementary file 1 — Supplementary Information. [file 41598_2023_44718_MOESM1_ESM.zip › Dataset/test set-system_evaluation/test_set_15fps/106.jpg]

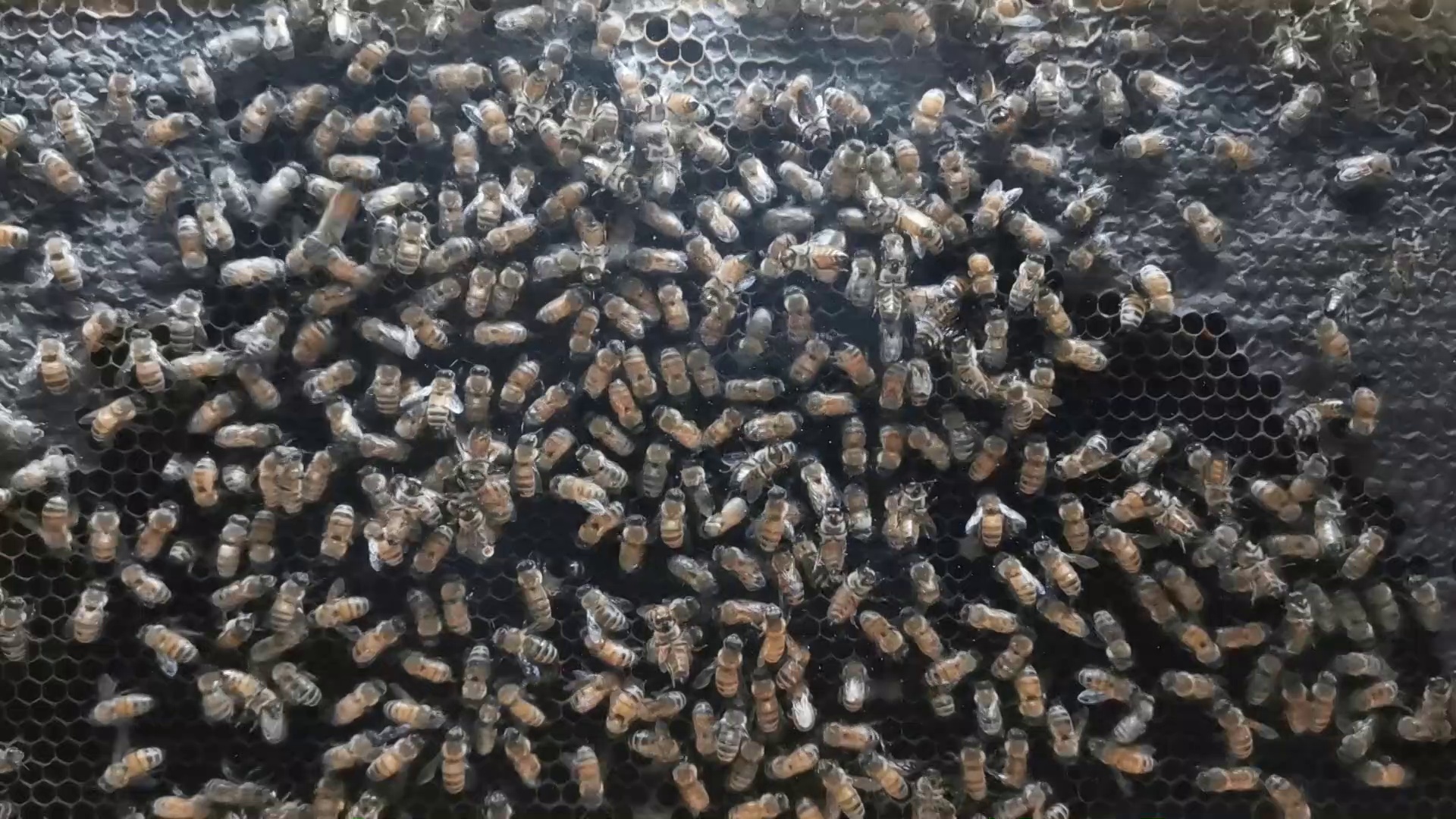

Supplement: Supplementary file 1 — Supplementary Information. [file 41598_2023_44718_MOESM1_ESM.zip › Dataset/test set-system_evaluation/test_set_15fps/102.jpg]

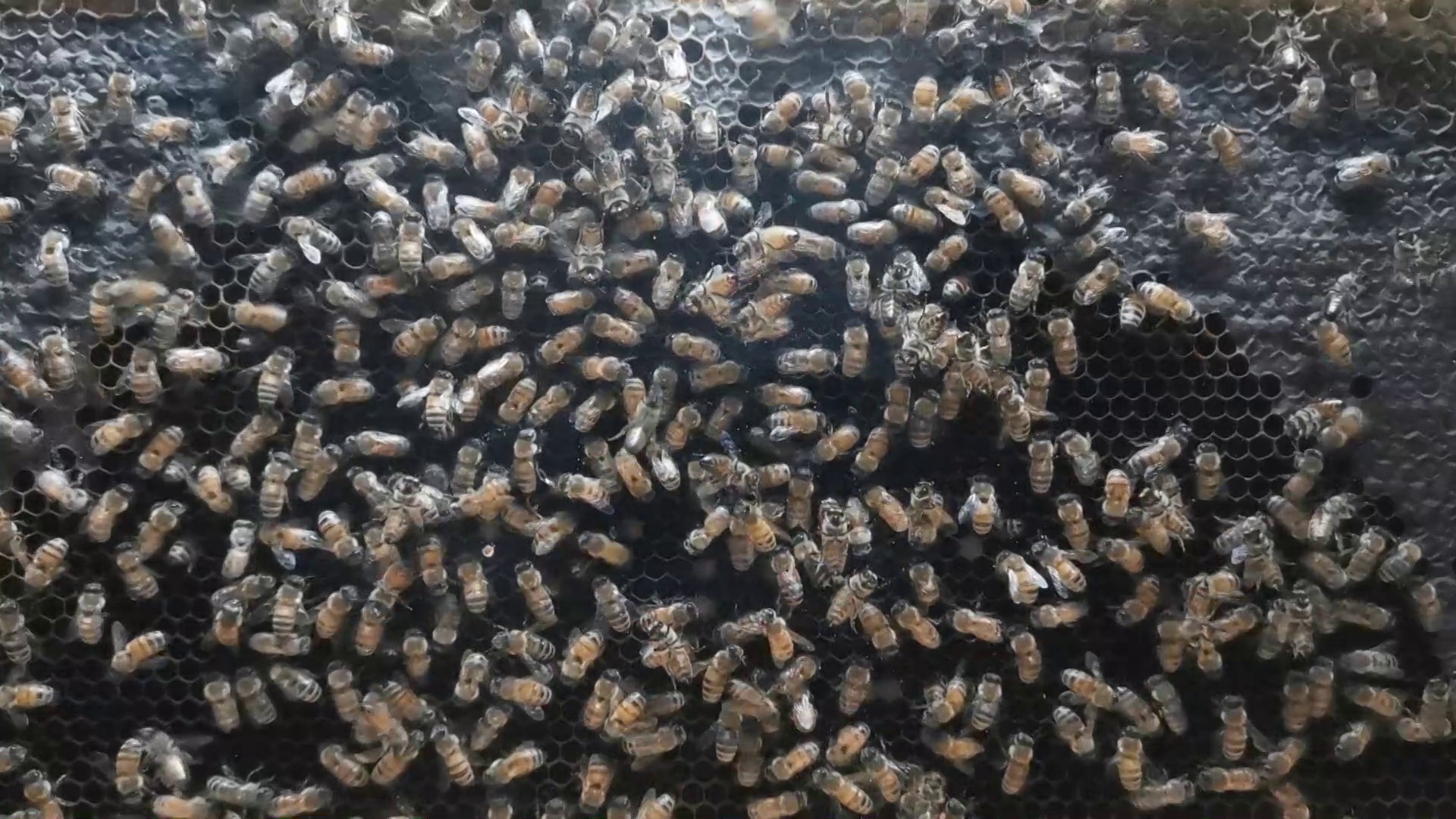

Supplement: Supplementary file 1 — Supplementary Information. [file 41598_2023_44718_MOESM1_ESM.zip › Dataset/test set-system_evaluation/test_set_15fps/138.jpg]

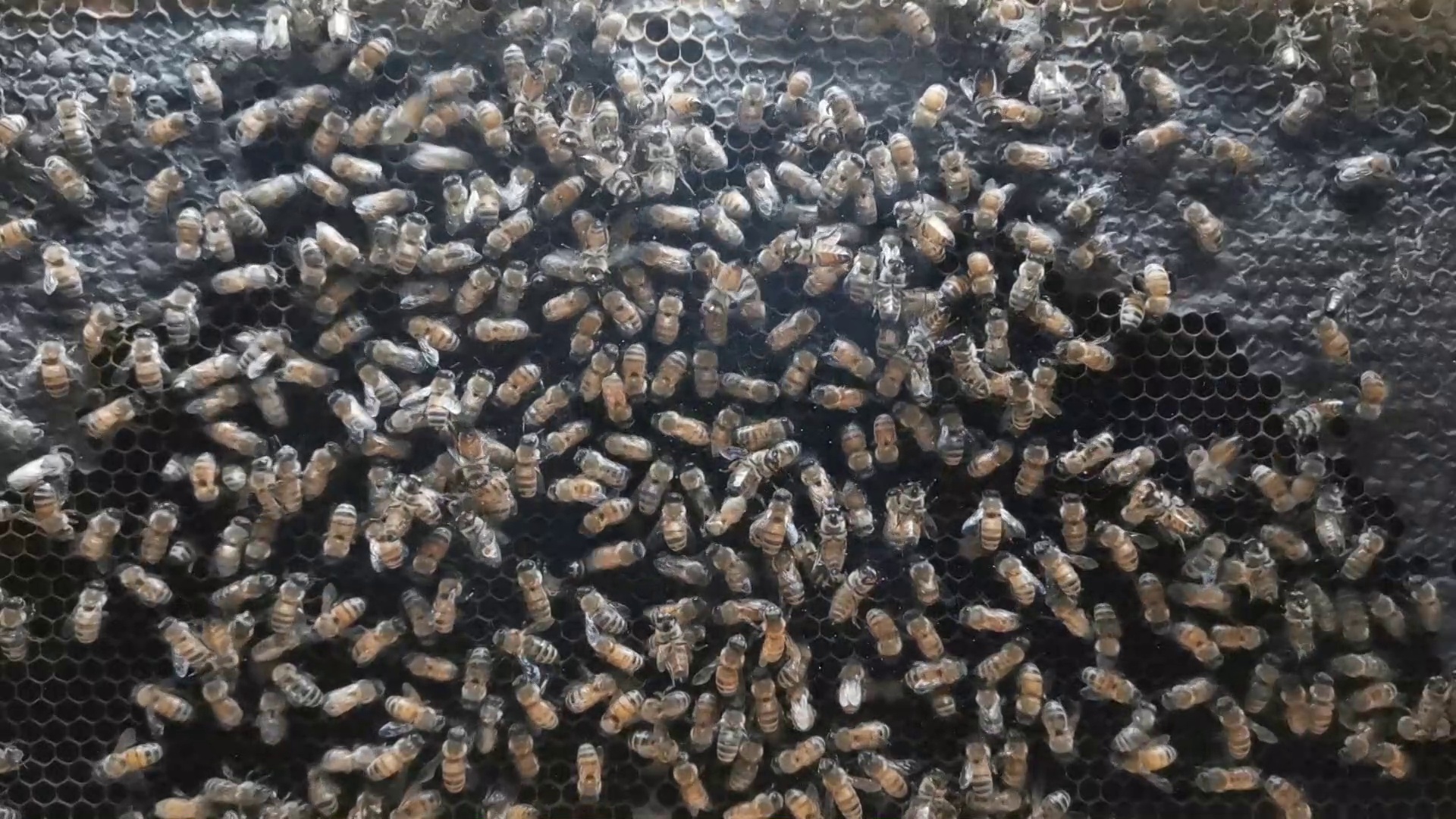

Supplement: Supplementary file 1 — Supplementary Information. [file 41598_2023_44718_MOESM1_ESM.zip › Dataset/test set-system_evaluation/test_set_15fps/096.jpg]

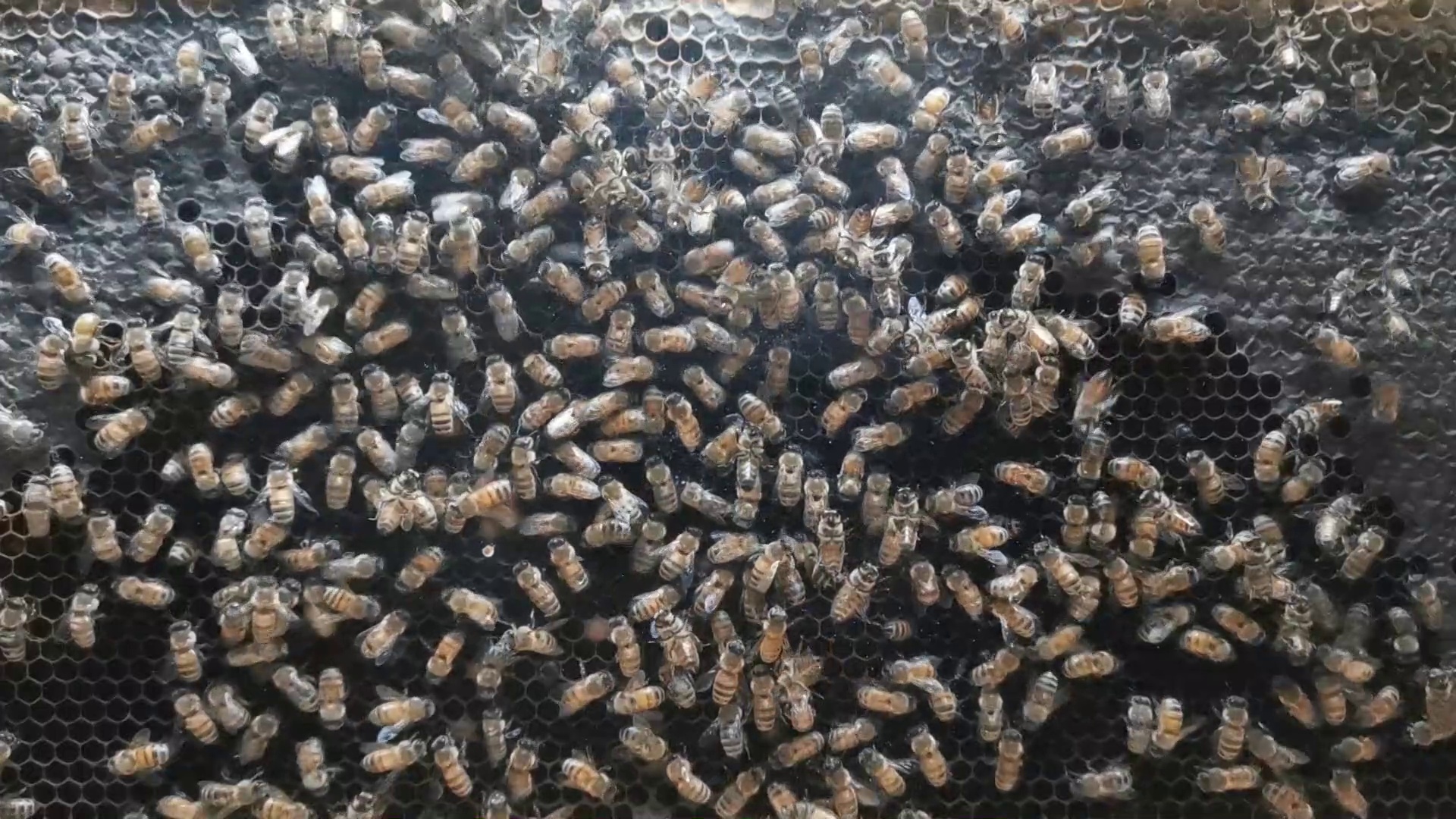

Supplement: Supplementary file 1 — Supplementary Information. [file 41598_2023_44718_MOESM1_ESM.zip › Dataset/test set-system_evaluation/test_set_15fps/036.jpg]

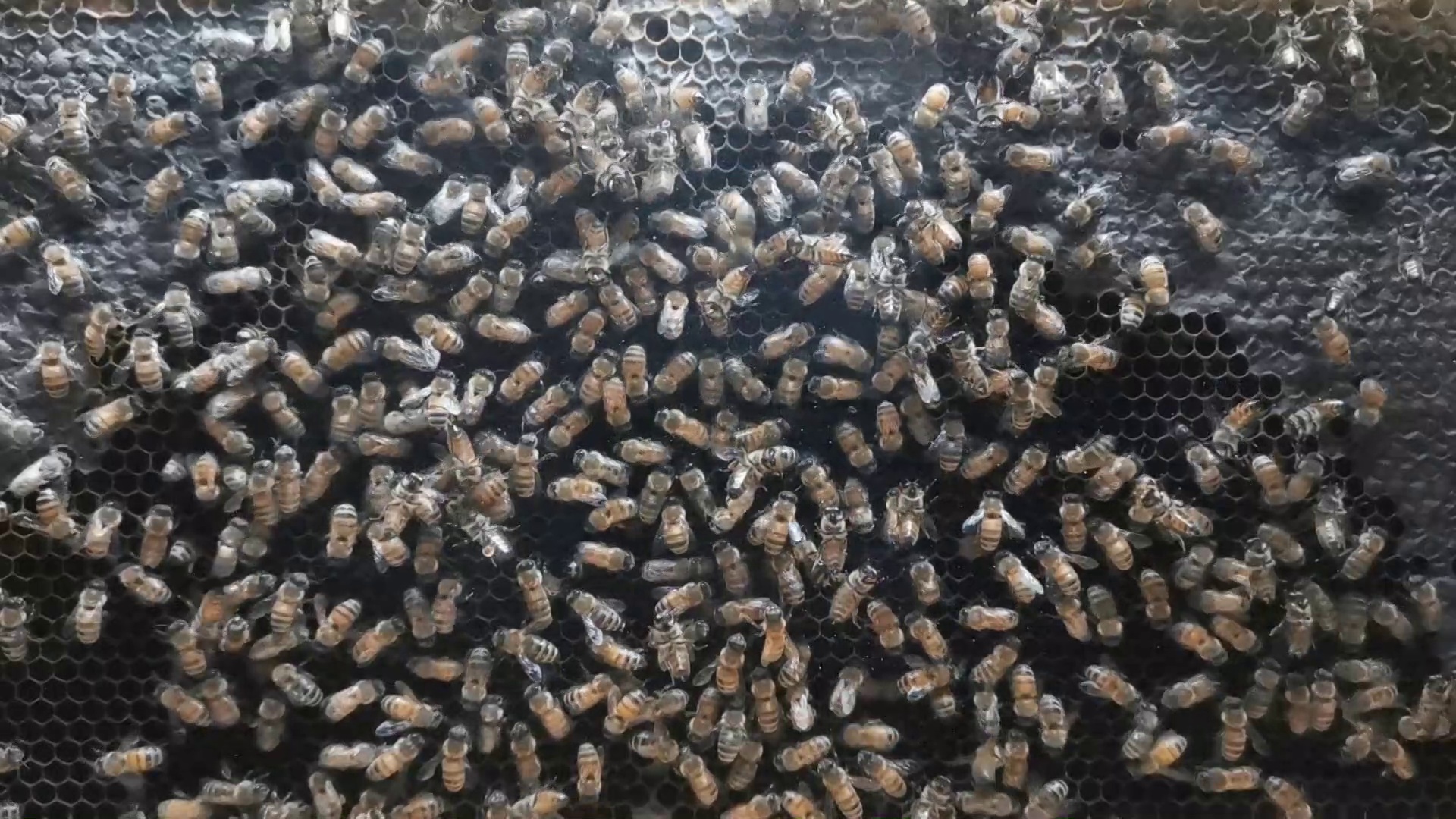

Supplement: Supplementary file 1 — Supplementary Information. [file 41598_2023_44718_MOESM1_ESM.zip › Dataset/test set-system_evaluation/test_set_15fps/092.jpg]

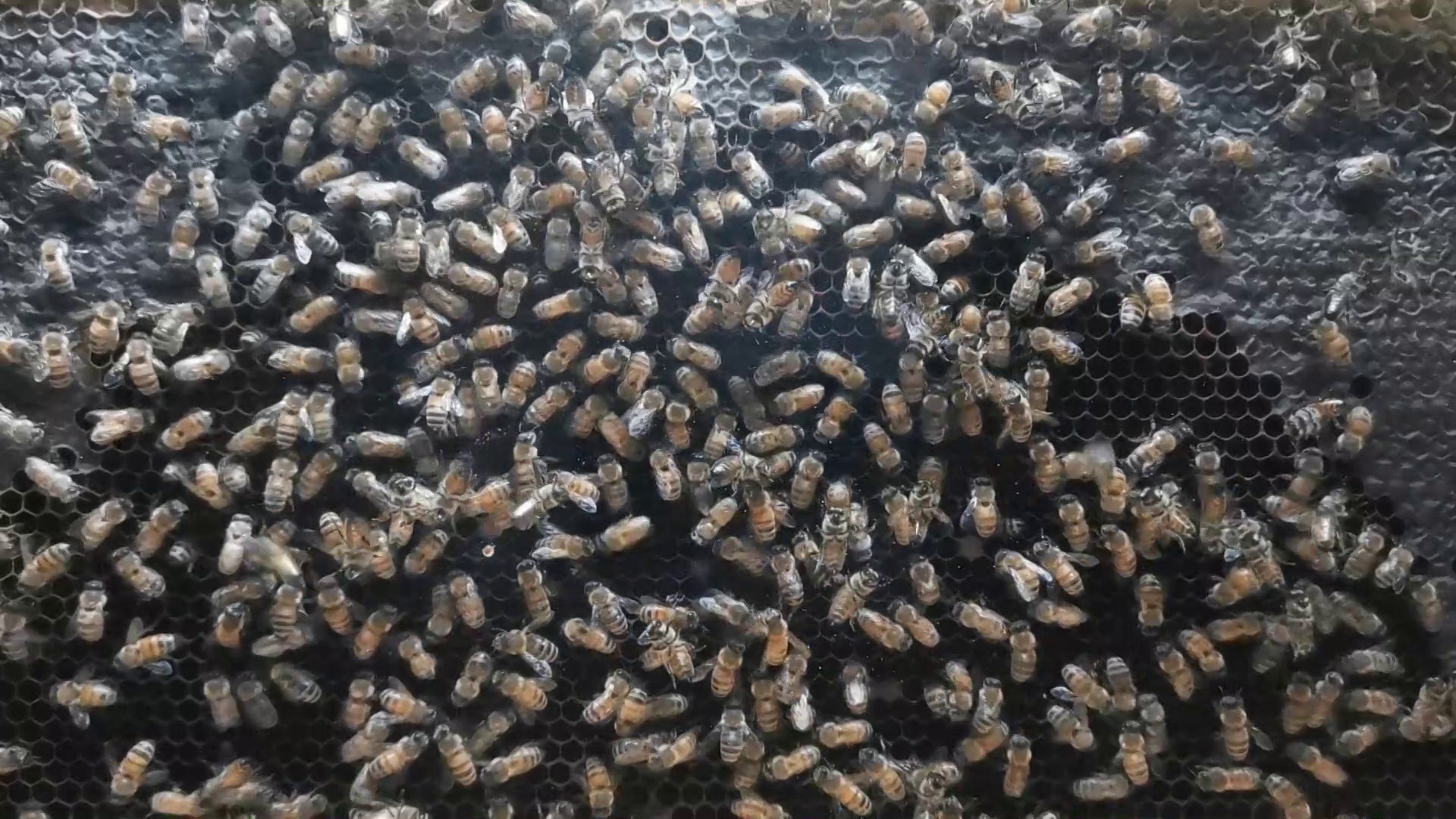

Supplement: Supplementary file 1 — Supplementary Information. [file 41598_2023_44718_MOESM1_ESM.zip › Dataset/test set-system_evaluation/test_set_15fps/124.jpg]

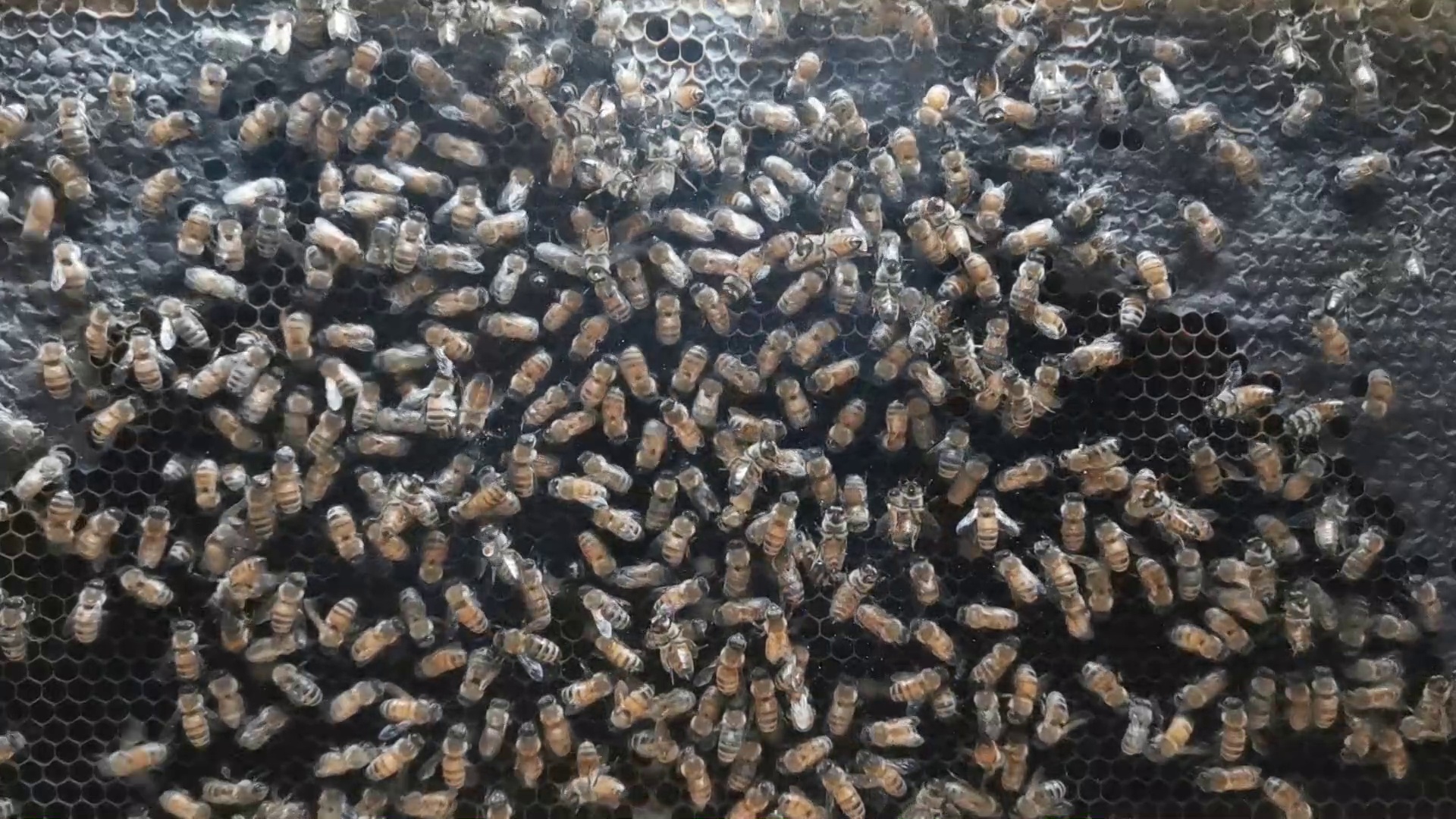

Supplement: Supplementary file 1 — Supplementary Information. [file 41598_2023_44718_MOESM1_ESM.zip › Dataset/test set-system_evaluation/test_set_15fps/085.jpg]

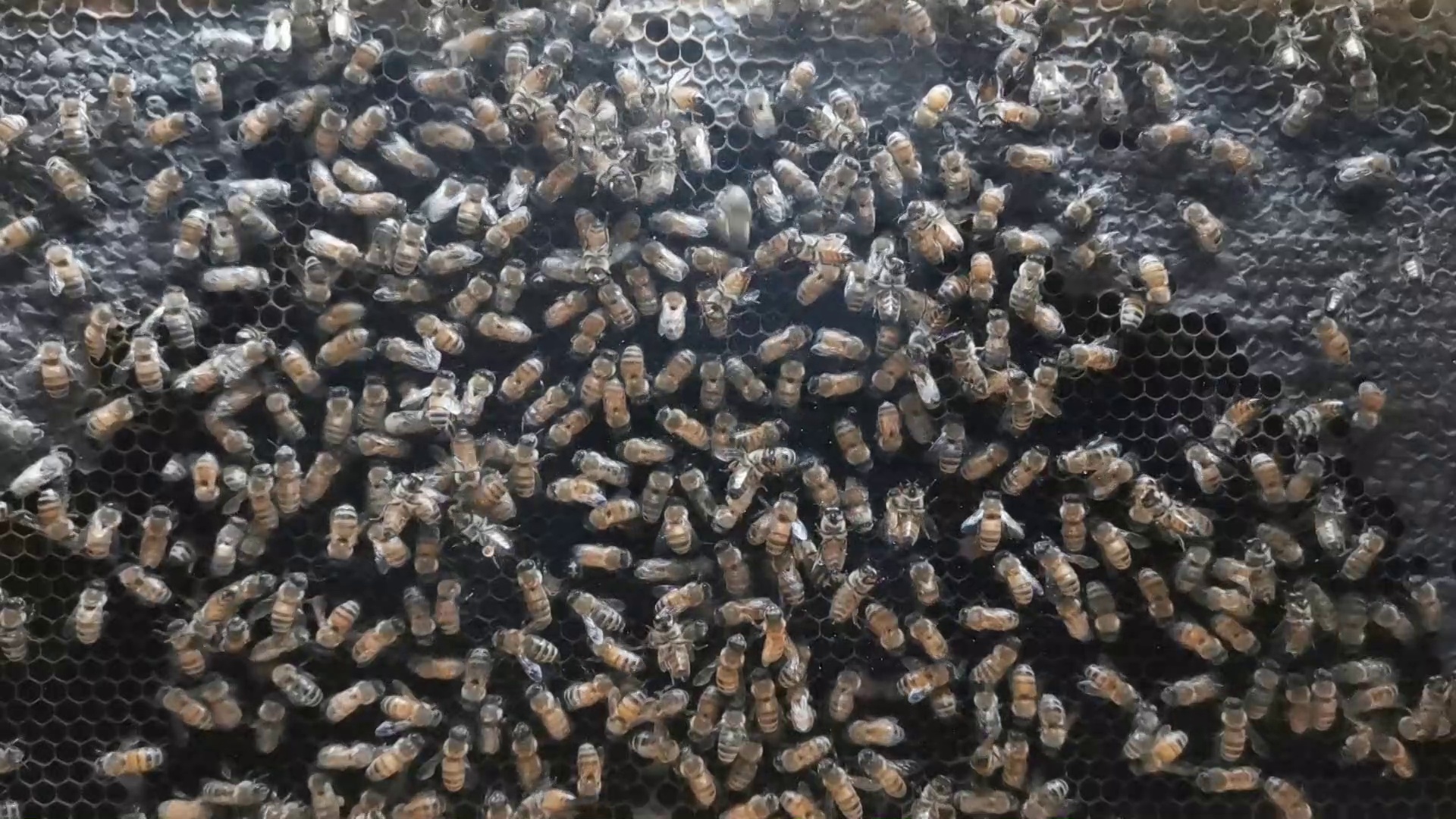

Supplement: Supplementary file 1 — Supplementary Information. [file 41598_2023_44718_MOESM1_ESM.zip › Dataset/test set-system_evaluation/test_set_15fps/091.jpg]

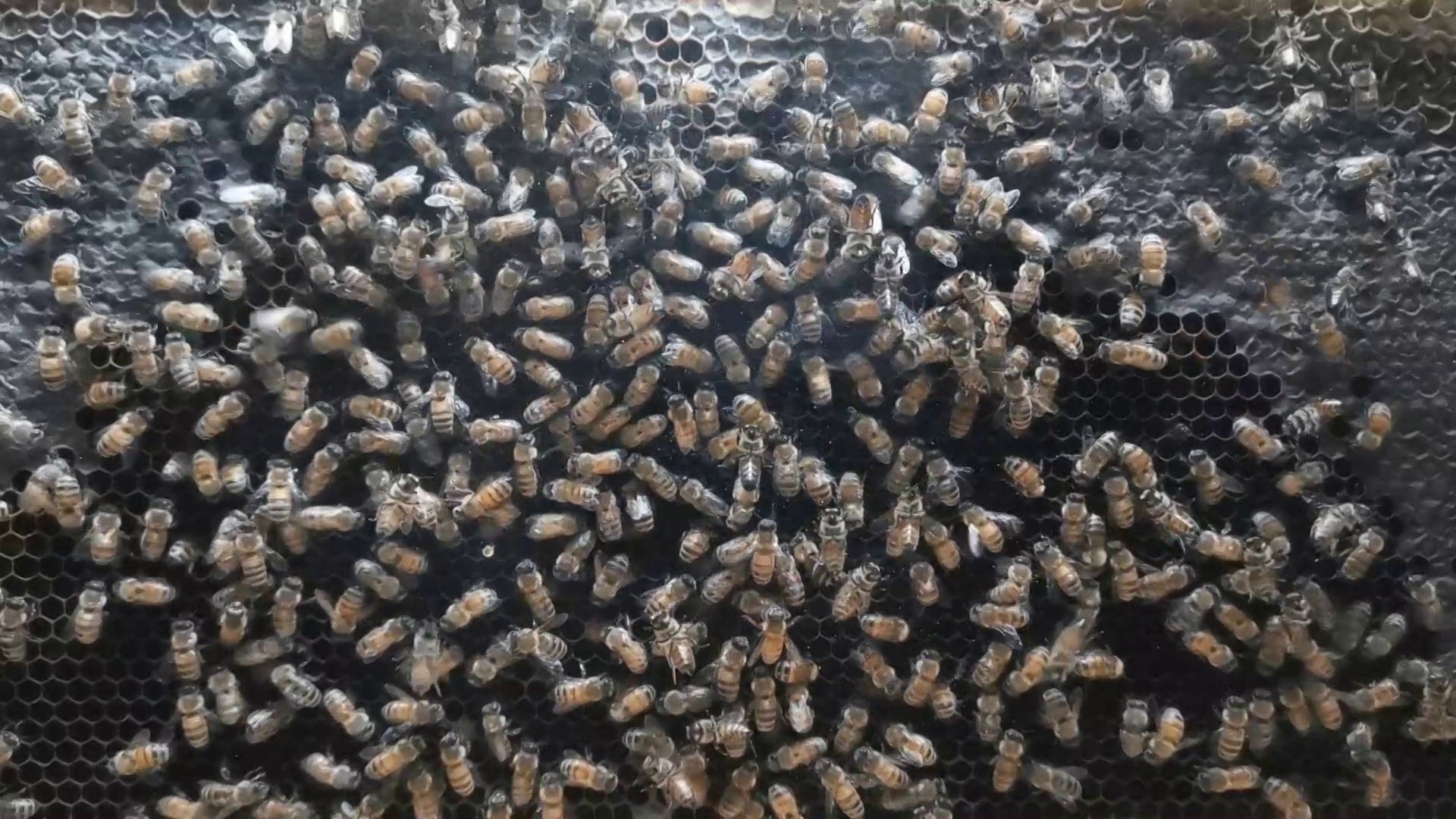

Supplement: Supplementary file 1 — Supplementary Information. [file 41598_2023_44718_MOESM1_ESM.zip › Dataset/test set-system_evaluation/test_set_15fps/056.jpg]

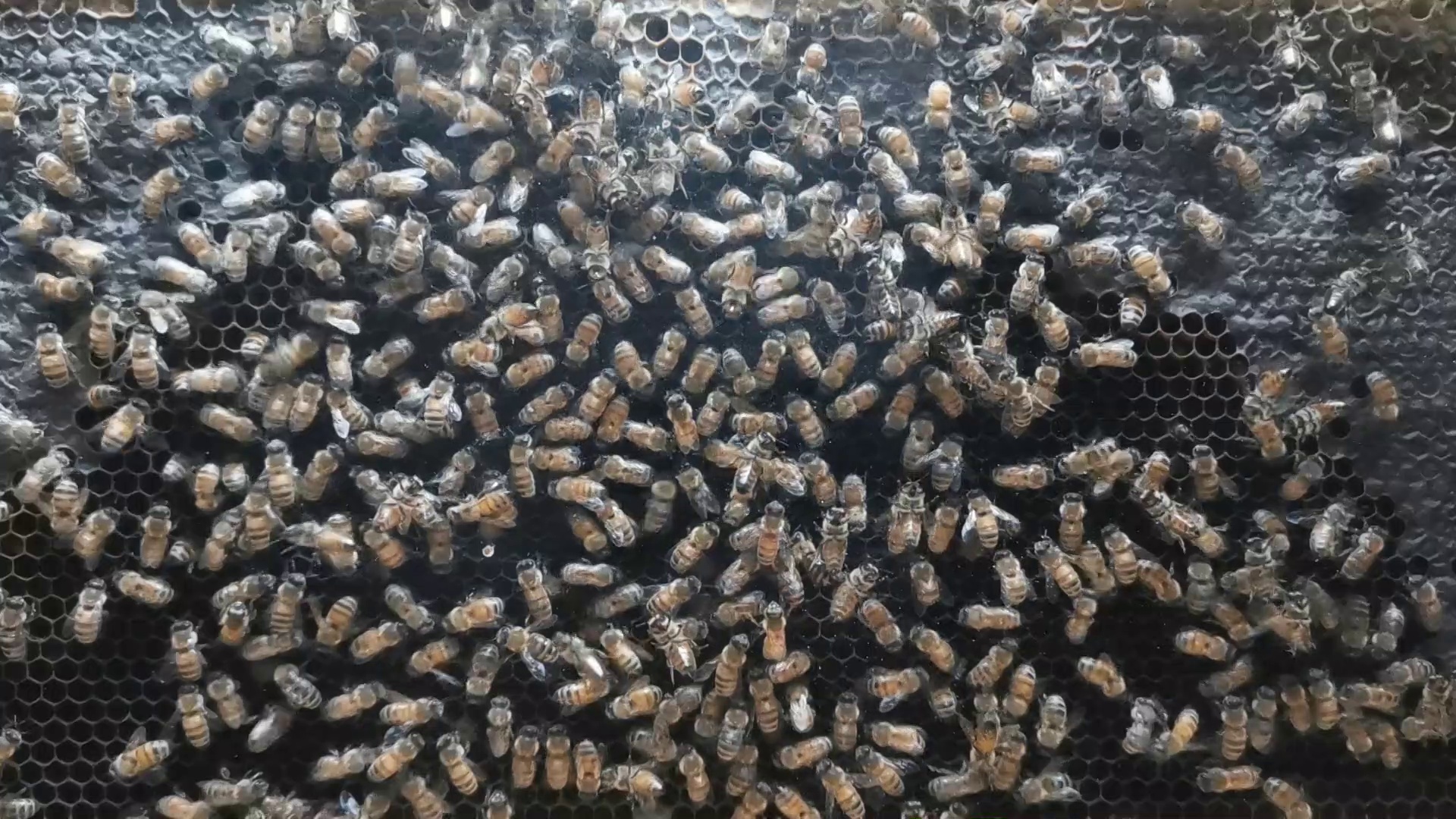

Supplement: Supplementary file 1 — Supplementary Information. [file 41598_2023_44718_MOESM1_ESM.zip › Dataset/test set-system_evaluation/test_set_15fps/074.jpg]

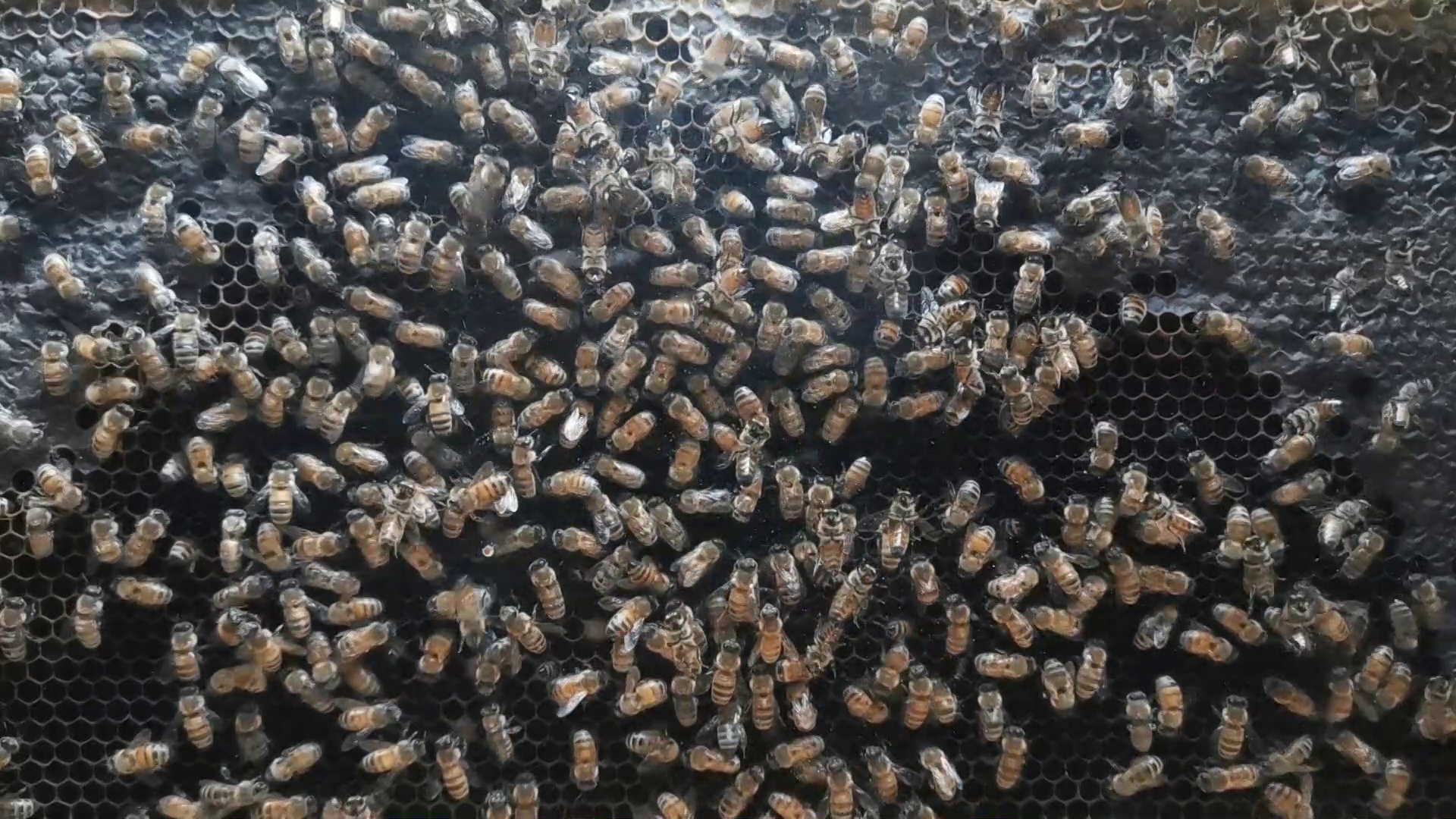

Supplement: Supplementary file 1 — Supplementary Information. [file 41598_2023_44718_MOESM1_ESM.zip › Dataset/test set-system_evaluation/test_set_15fps/015.jpg]

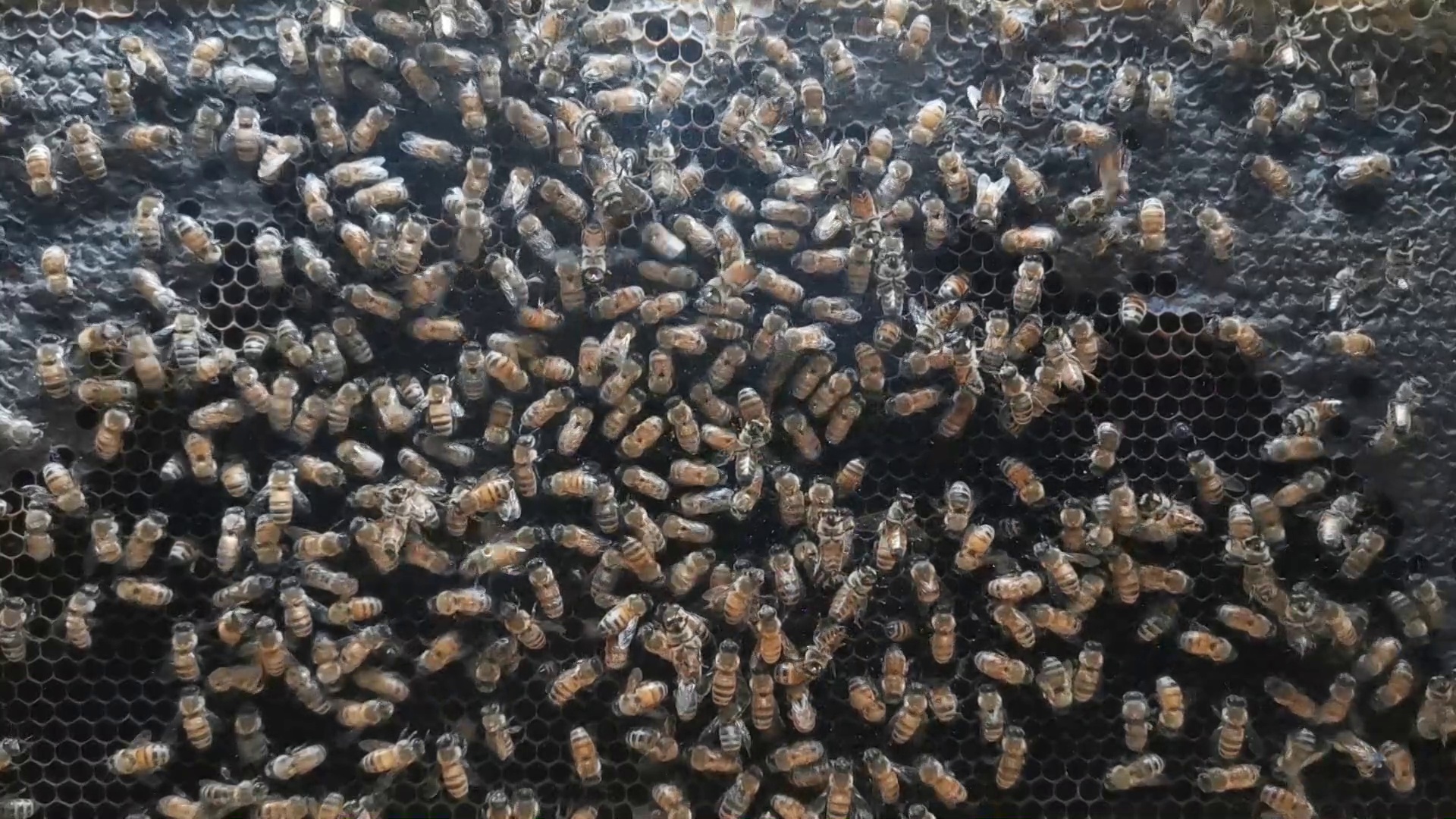

Supplement: Supplementary file 1 — Supplementary Information. [file 41598_2023_44718_MOESM1_ESM.zip › Dataset/test set-system_evaluation/test_set_15fps/009.jpg]

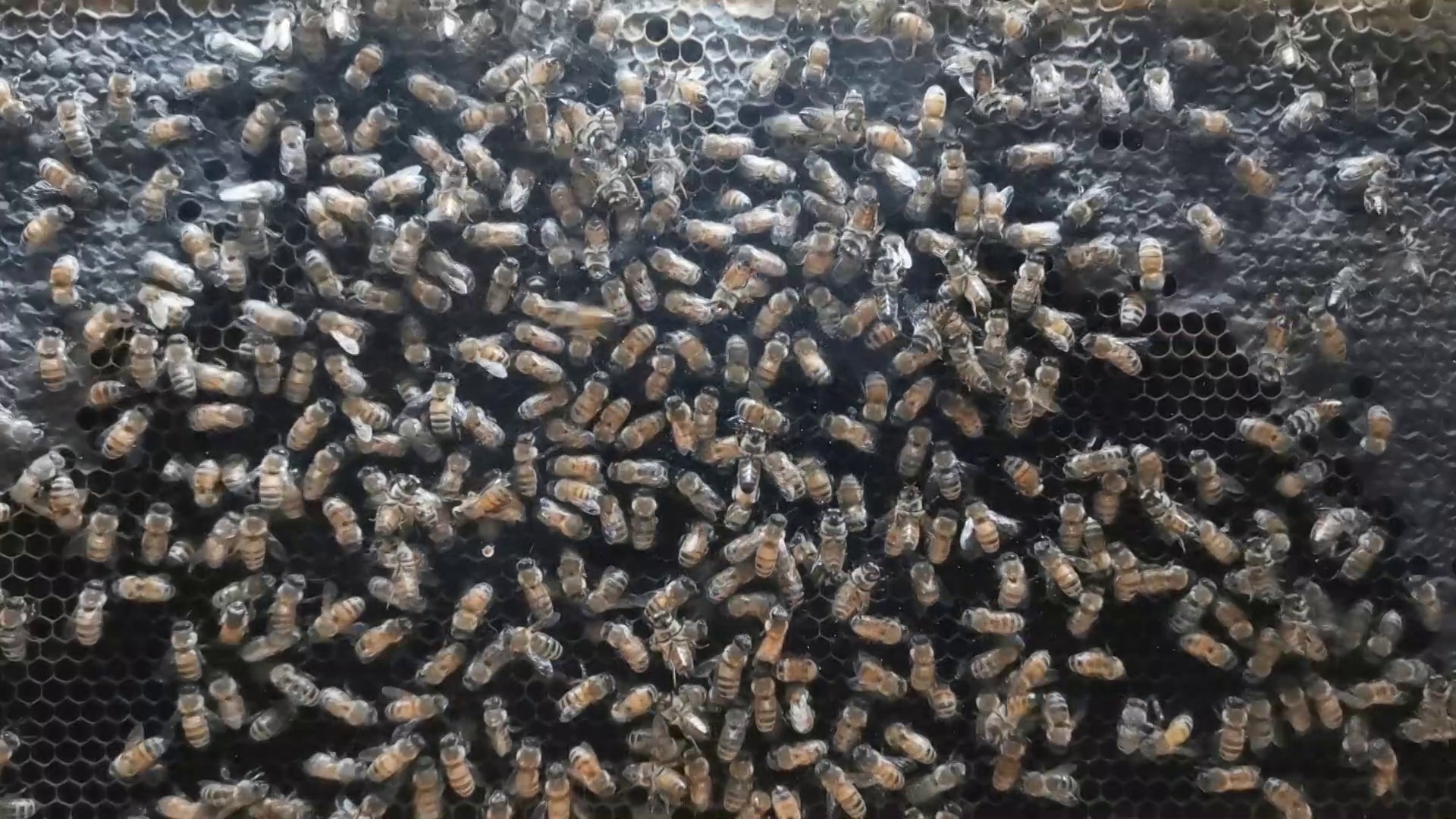

Supplement: Supplementary file 1 — Supplementary Information. [file 41598_2023_44718_MOESM1_ESM.zip › Dataset/test set-system_evaluation/test_set_15fps/063.jpg]

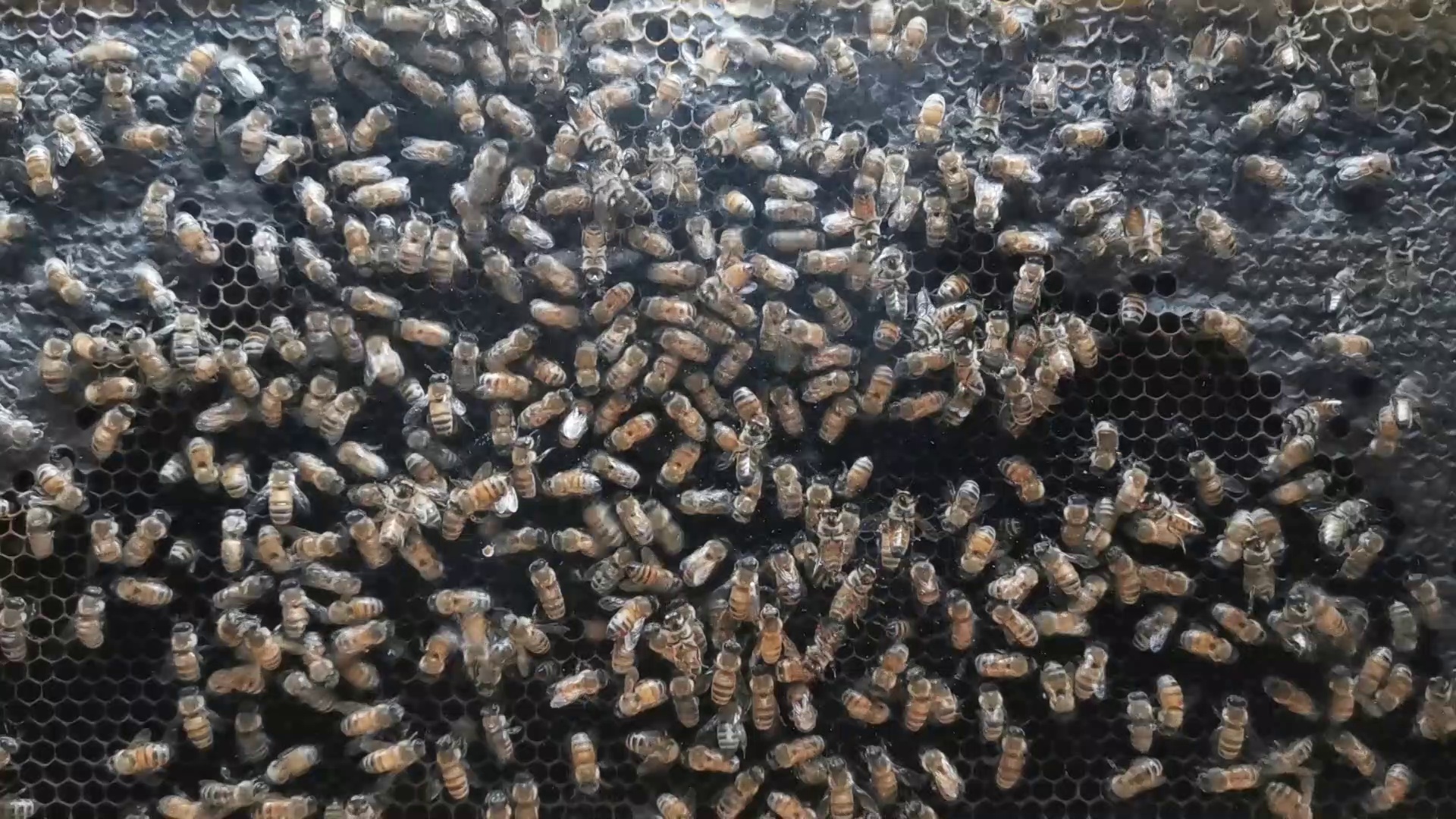

Supplement: Supplementary file 1 — Supplementary Information. [file 41598_2023_44718_MOESM1_ESM.zip › Dataset/test set-system_evaluation/test_set_15fps/016.jpg]

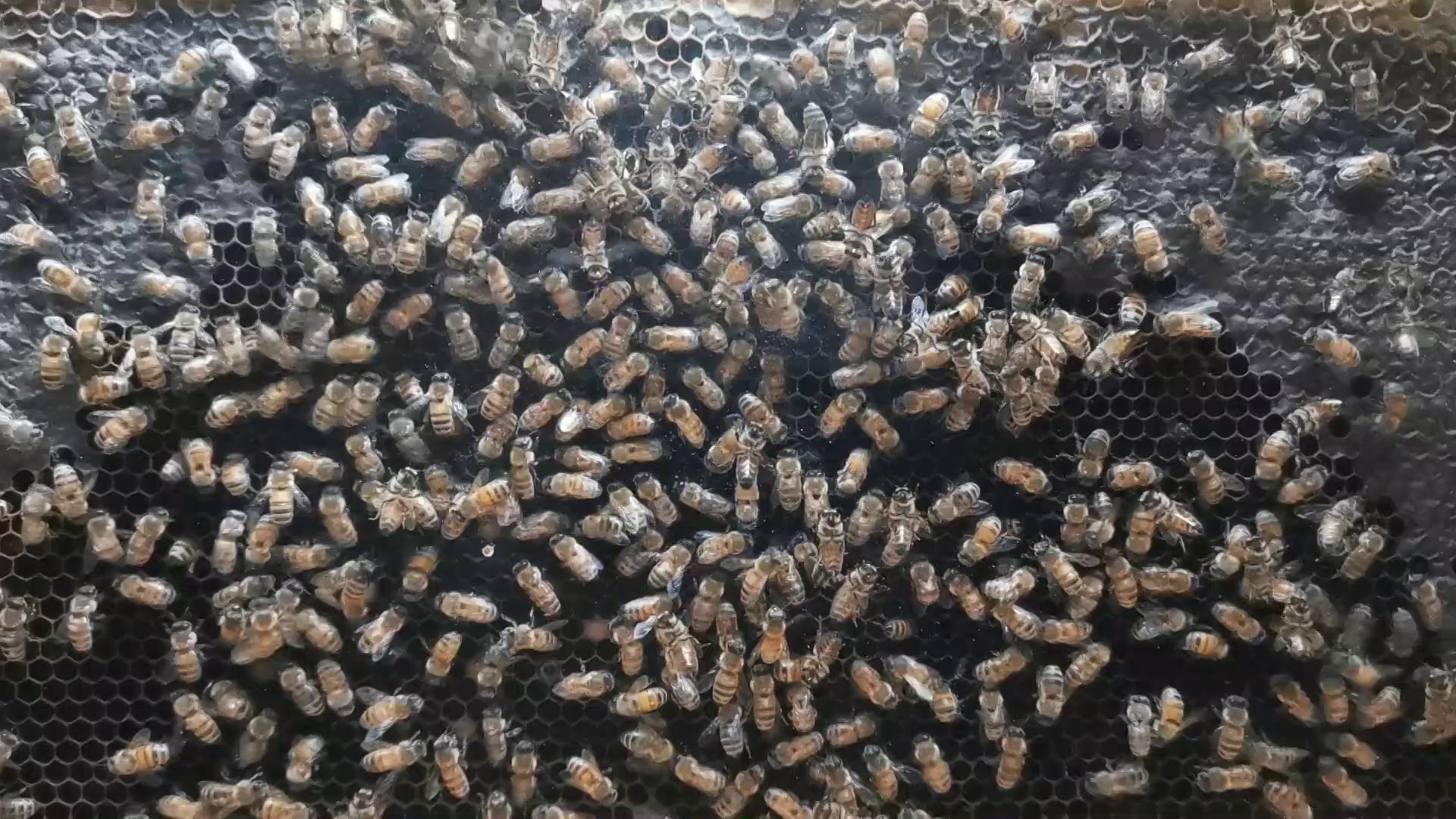

Supplement: Supplementary file 1 — Supplementary Information. [file 41598_2023_44718_MOESM1_ESM.zip › Dataset/test set-system_evaluation/test_set_15fps/030.jpg]

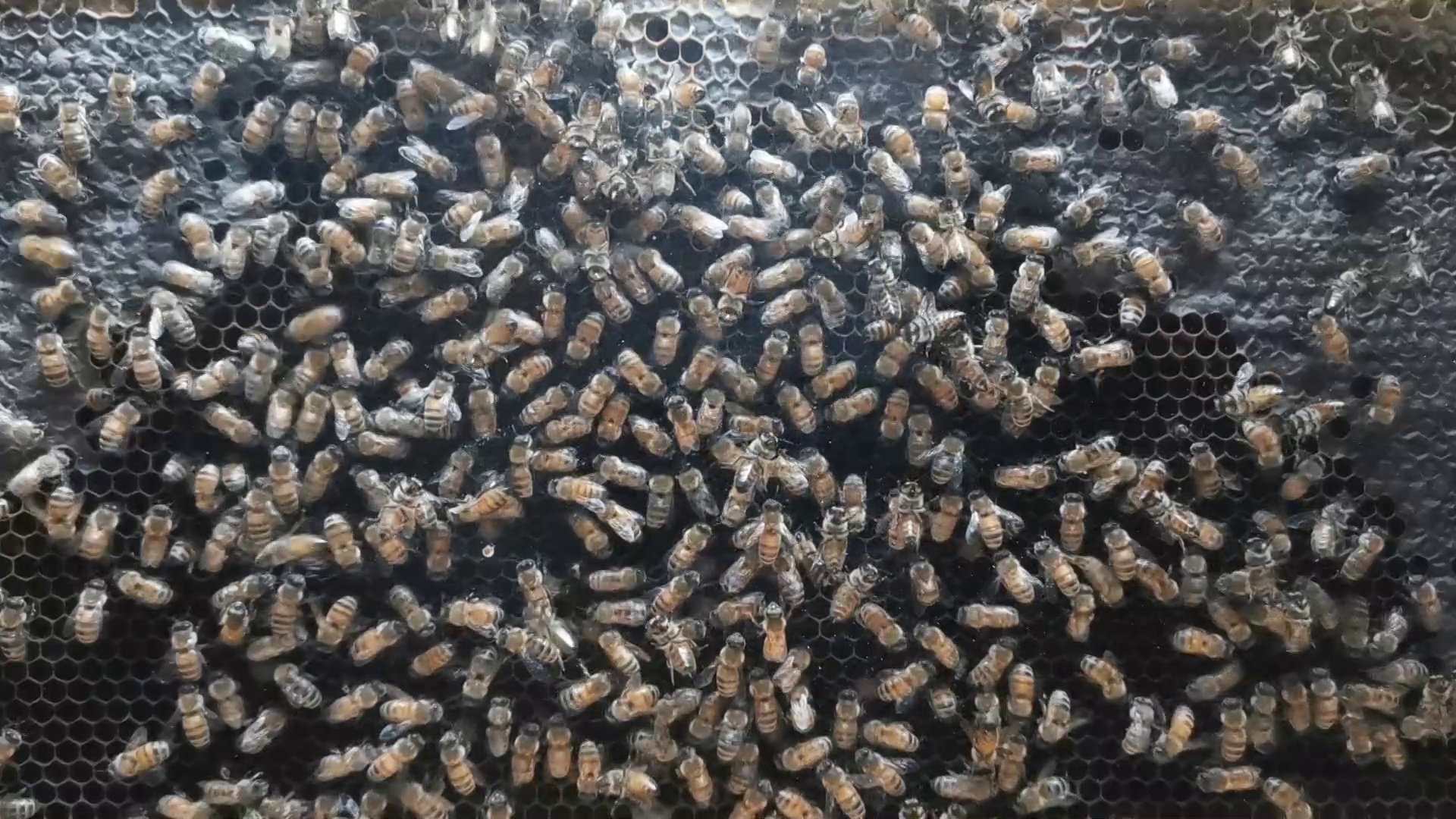

Supplement: Supplementary file 1 — Supplementary Information. [file 41598_2023_44718_MOESM1_ESM.zip › Dataset/test set-system_evaluation/test_set_15fps/077.jpg]

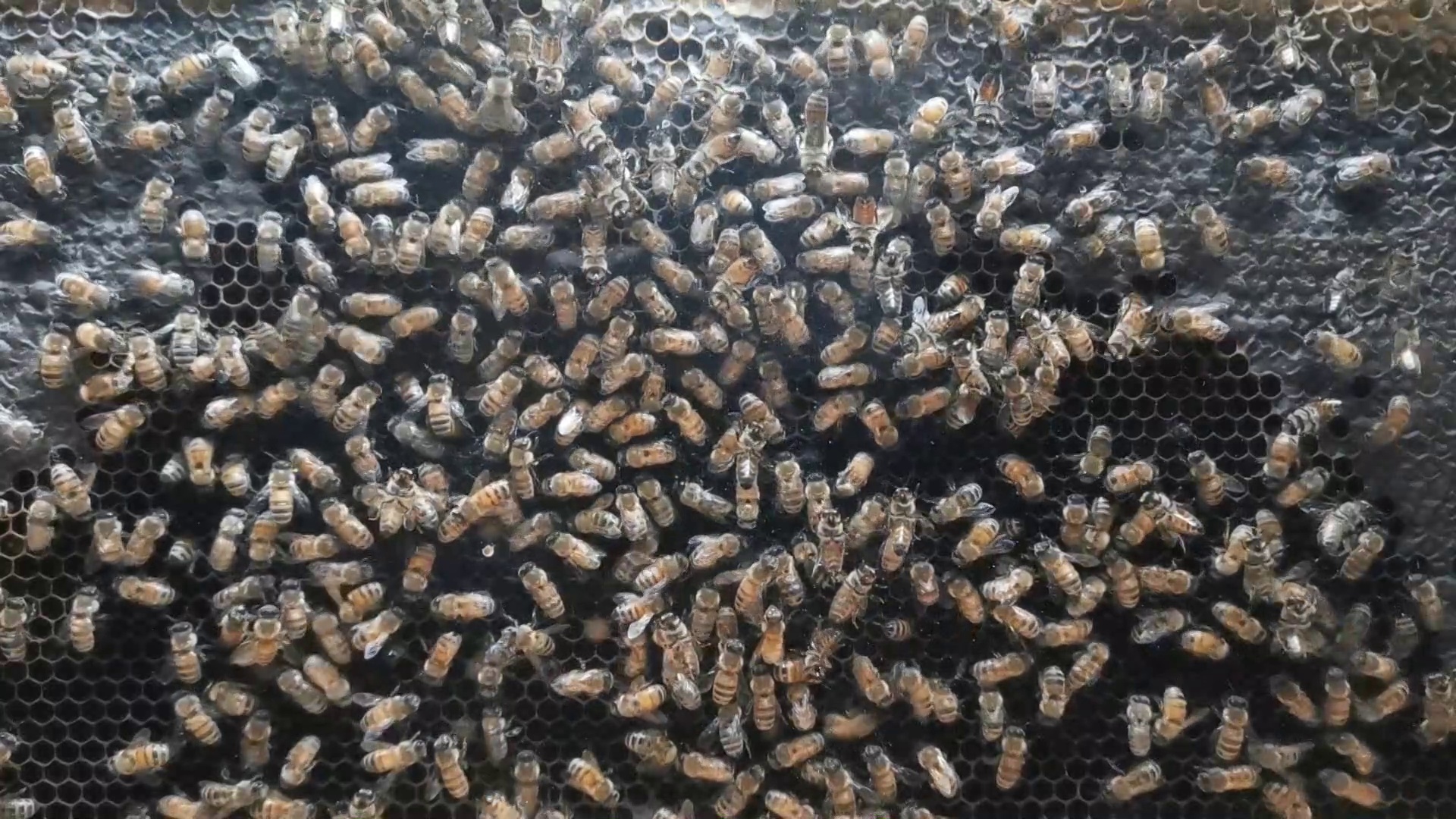

Supplement: Supplementary file 1 — Supplementary Information. [file 41598_2023_44718_MOESM1_ESM.zip › Dataset/test set-system_evaluation/test_set_15fps/026.jpg]

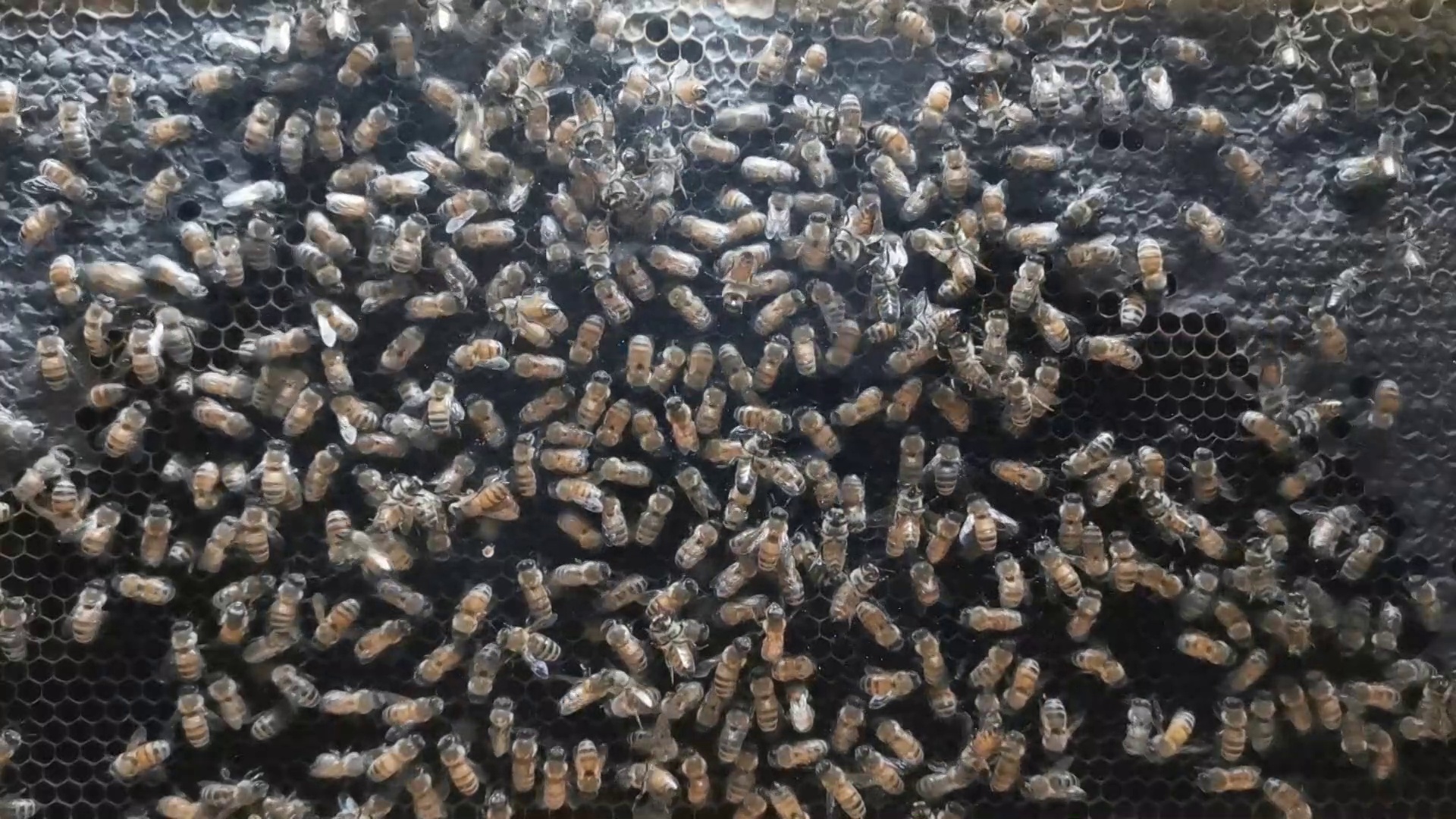

Supplement: Supplementary file 1 — Supplementary Information. [file 41598_2023_44718_MOESM1_ESM.zip › Dataset/test set-system_evaluation/test_set_15fps/069.jpg]

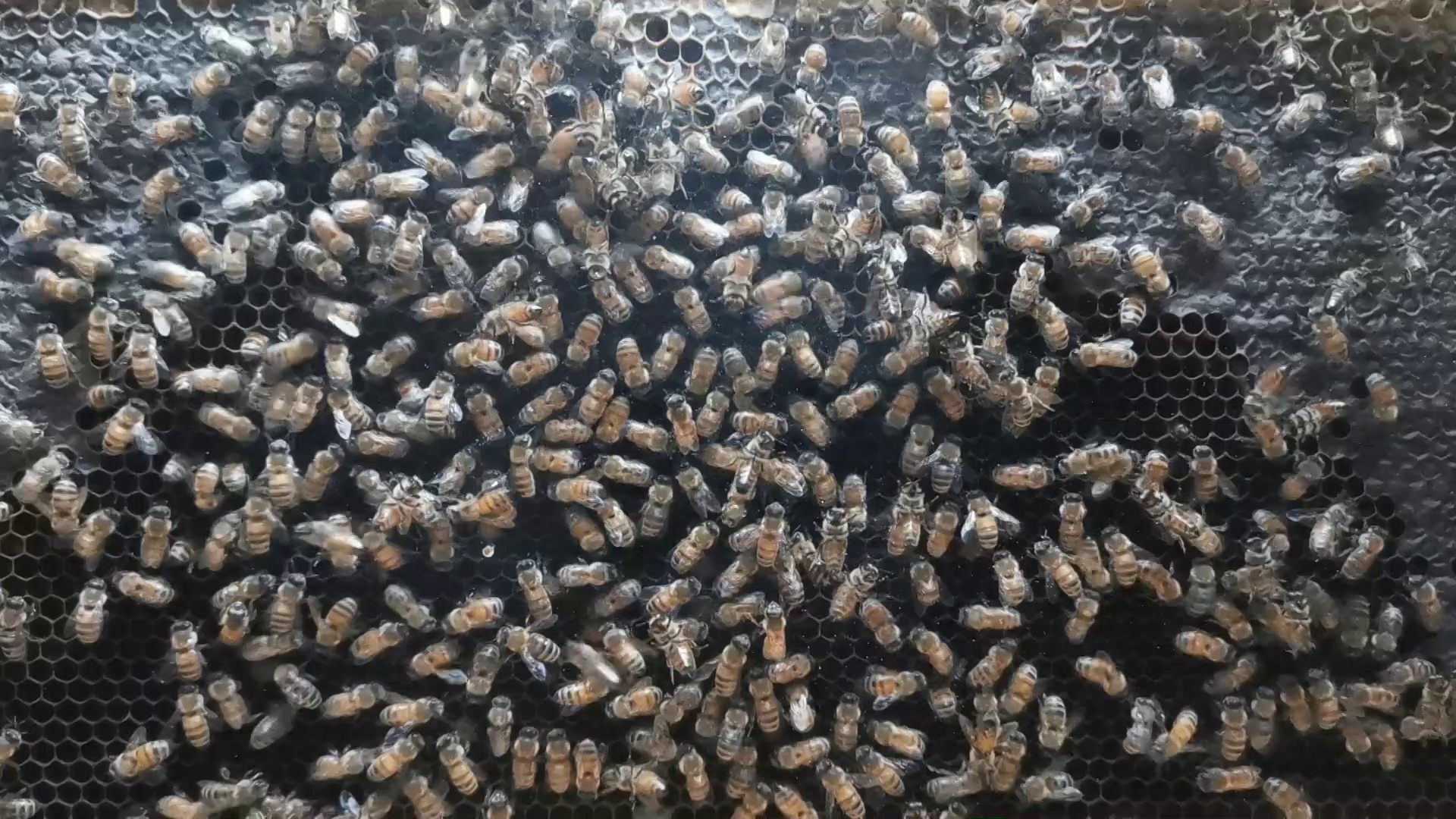

Supplement: Supplementary file 1 — Supplementary Information. [file 41598_2023_44718_MOESM1_ESM.zip › Dataset/test set-system_evaluation/test_set_15fps/073.jpg]

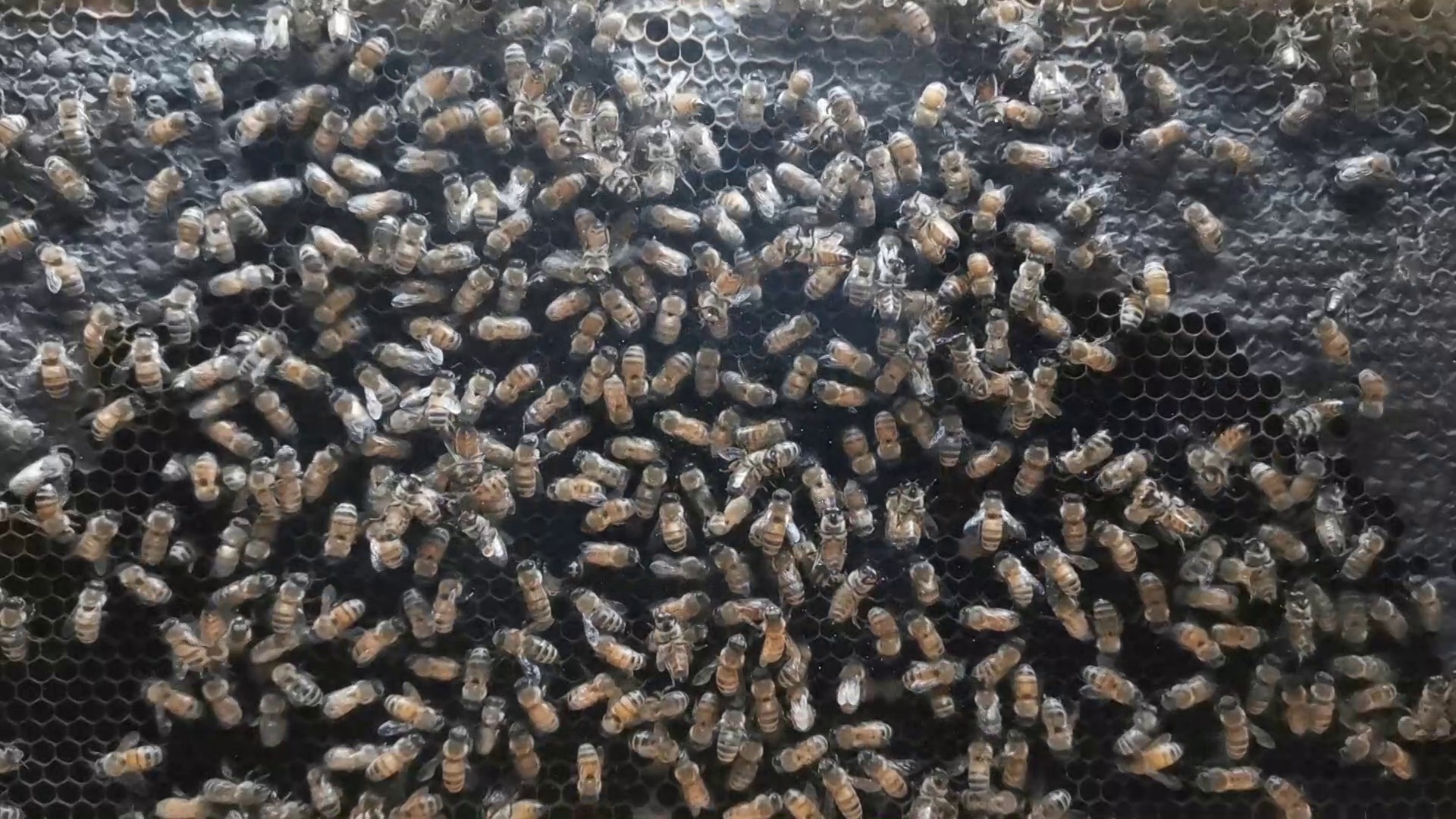

Supplement: Supplementary file 1 — Supplementary Information. [file 41598_2023_44718_MOESM1_ESM.zip › Dataset/test set-system_evaluation/test_set_15fps/095.jpg]

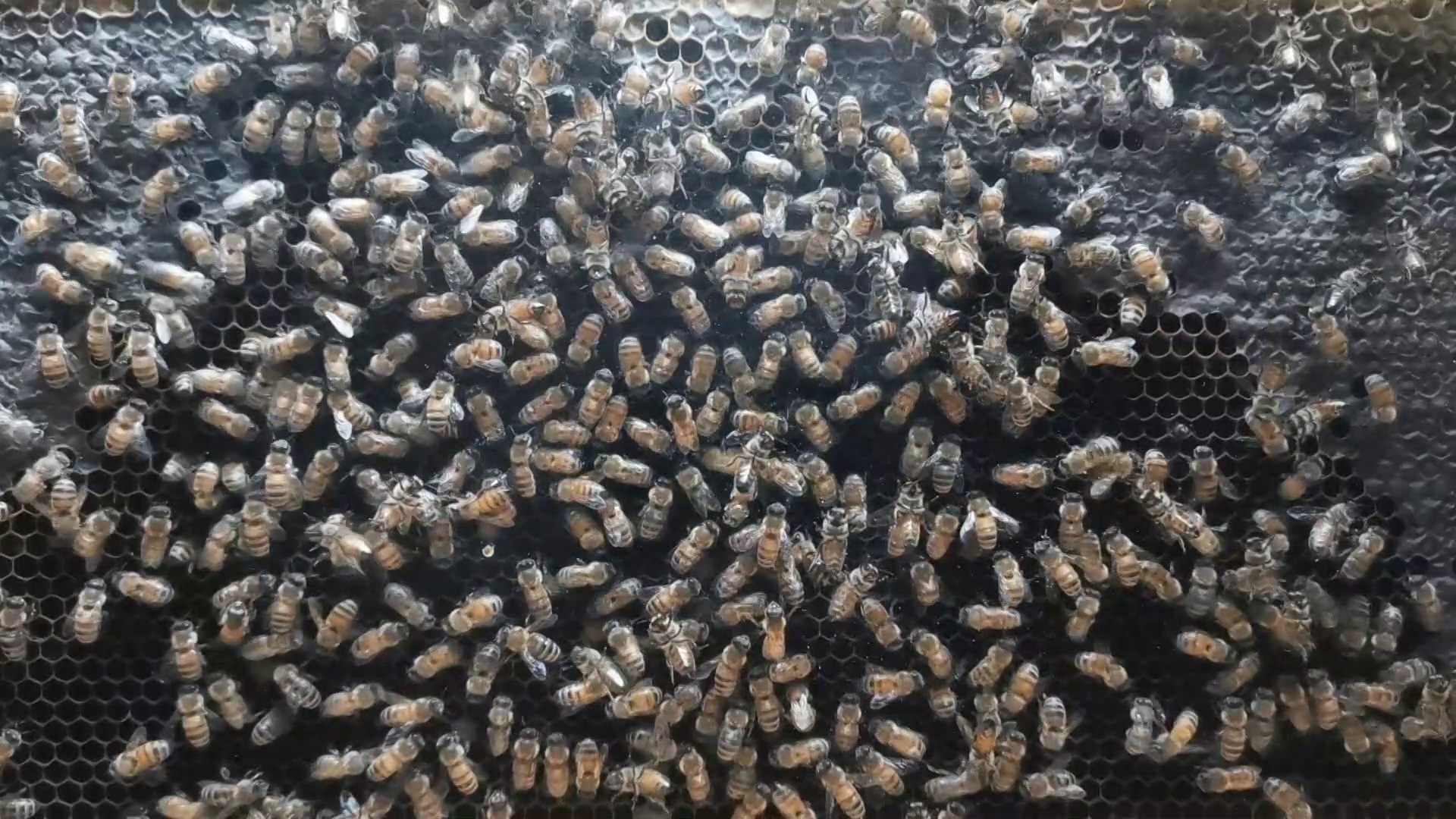

Supplement: Supplementary file 1 — Supplementary Information. [file 41598_2023_44718_MOESM1_ESM.zip › Dataset/test set-system_evaluation/test_set_15fps/072.jpg]

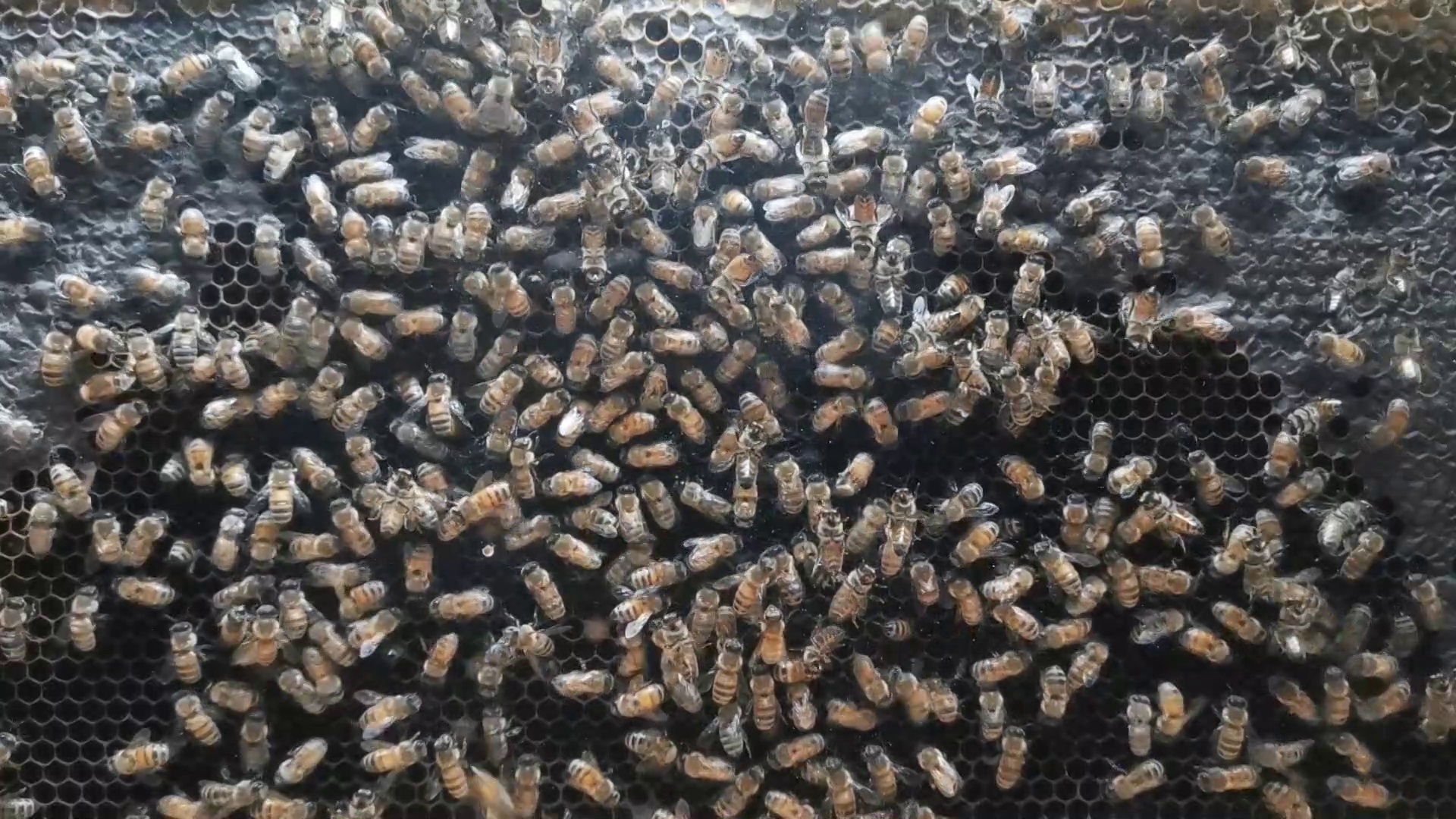

Supplement: Supplementary file 1 — Supplementary Information. [file 41598_2023_44718_MOESM1_ESM.zip › Dataset/test set-system_evaluation/test_set_15fps/025.jpg]

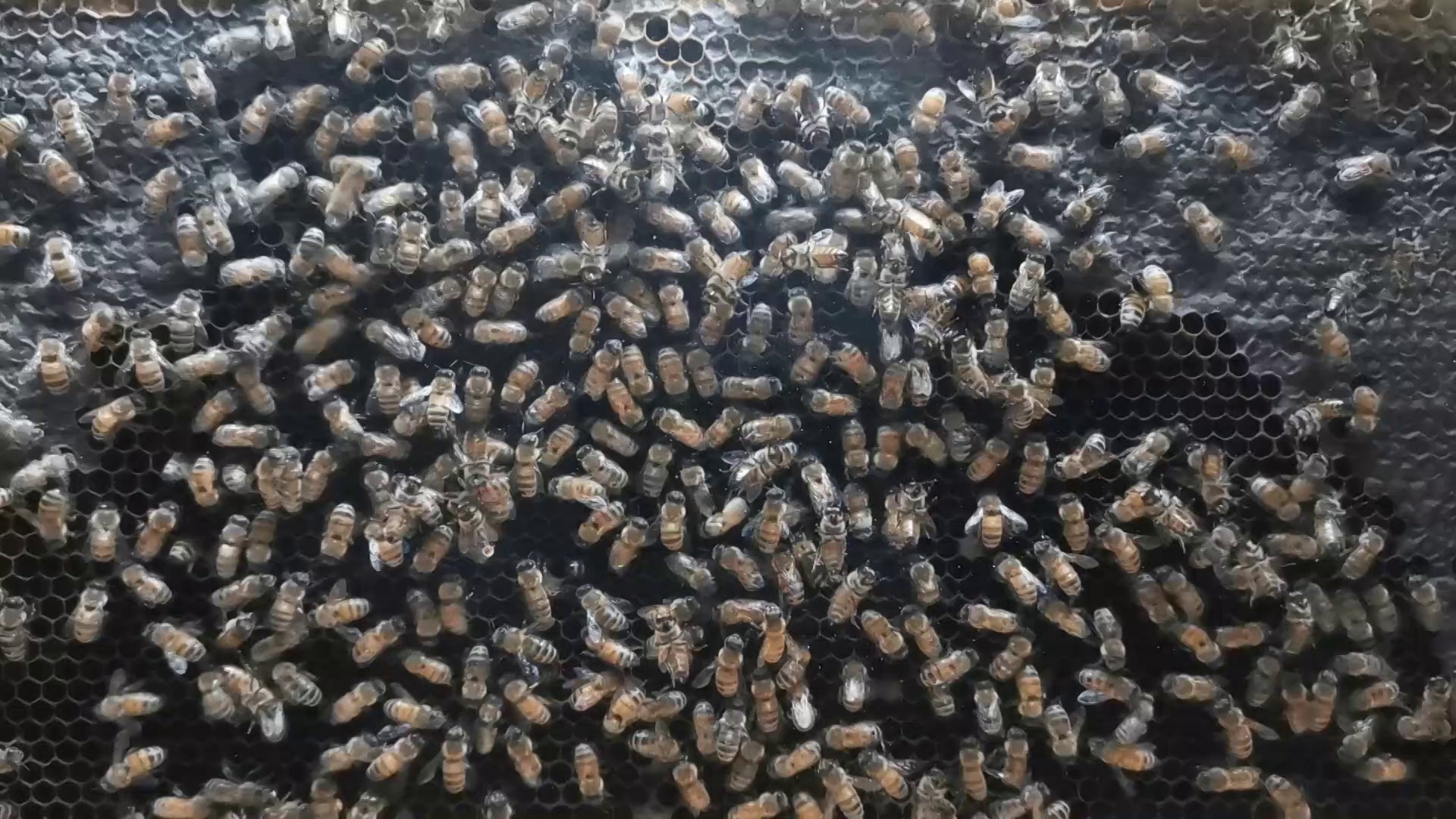

Supplement: Supplementary file 1 — Supplementary Information. [file 41598_2023_44718_MOESM1_ESM.zip › Dataset/test set-system_evaluation/test_set_15fps/101.jpg]

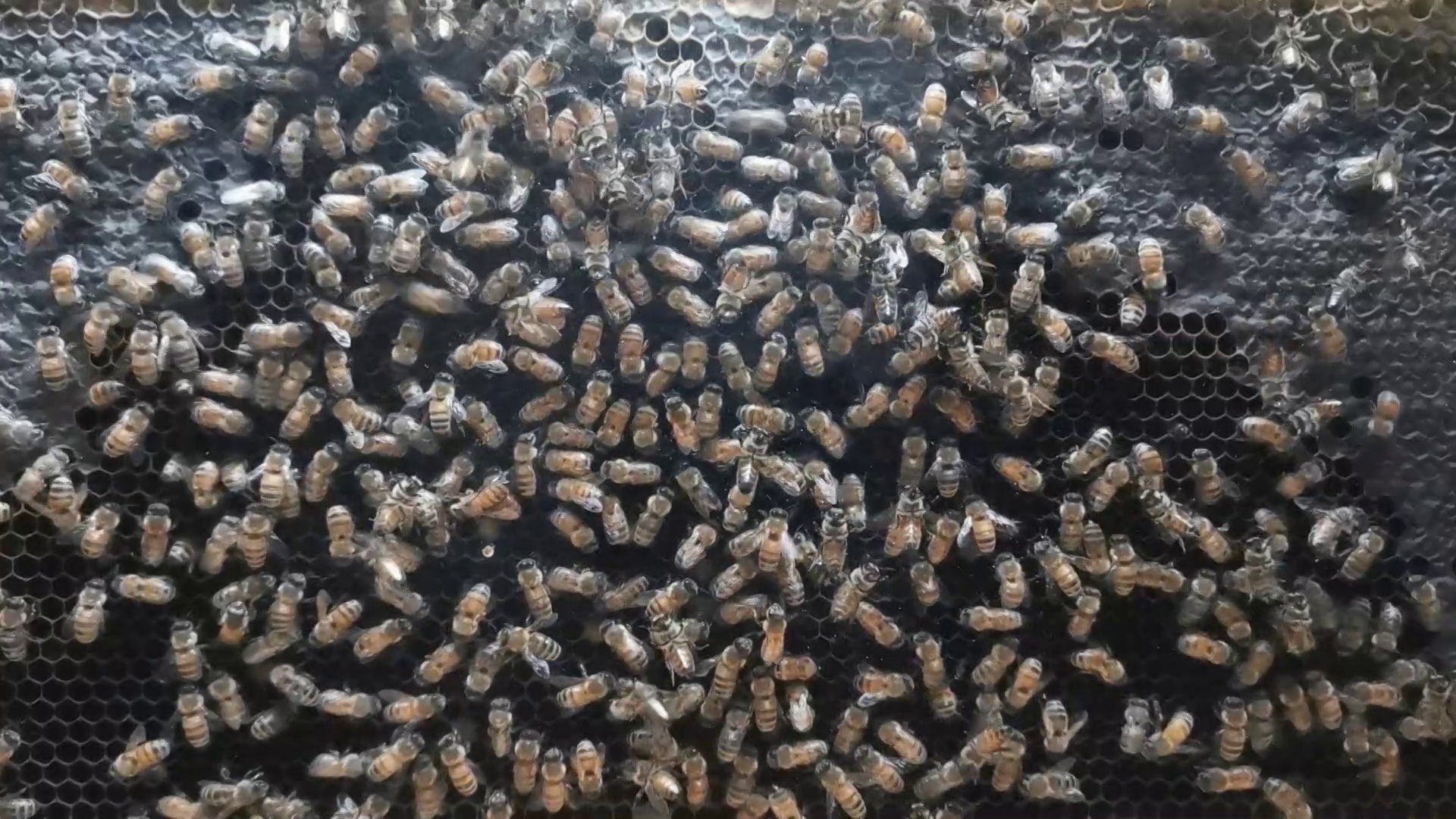

Supplement: Supplementary file 1 — Supplementary Information. [file 41598_2023_44718_MOESM1_ESM.zip › Dataset/test set-system_evaluation/test_set_15fps/067.jpg]

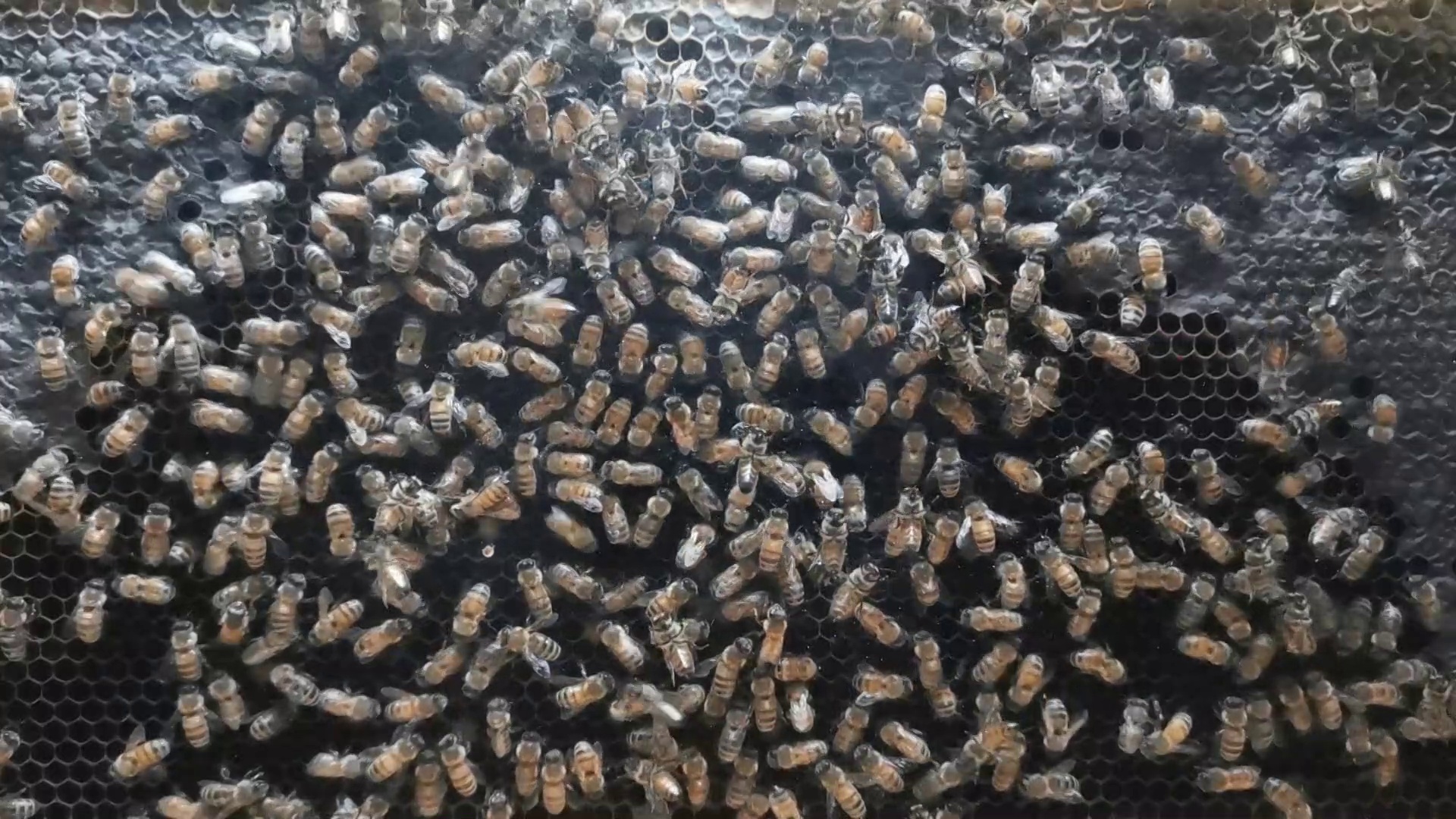

Supplement: Supplementary file 1 — Supplementary Information. [file 41598_2023_44718_MOESM1_ESM.zip › Dataset/test set-system_evaluation/test_set_15fps/066.jpg]

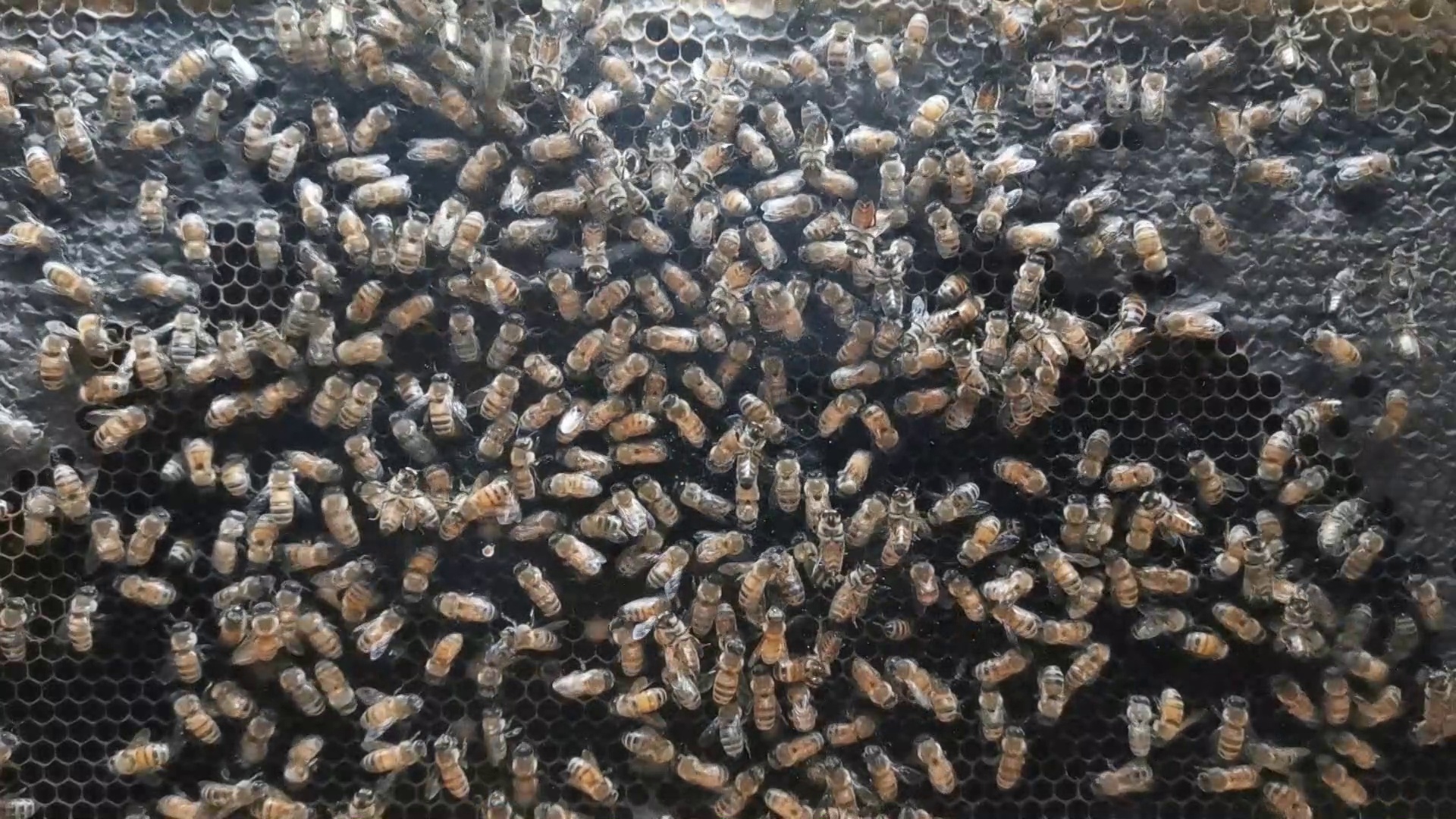

Supplement: Supplementary file 1 — Supplementary Information. [file 41598_2023_44718_MOESM1_ESM.zip › Dataset/test set-system_evaluation/test_set_15fps/029.jpg]

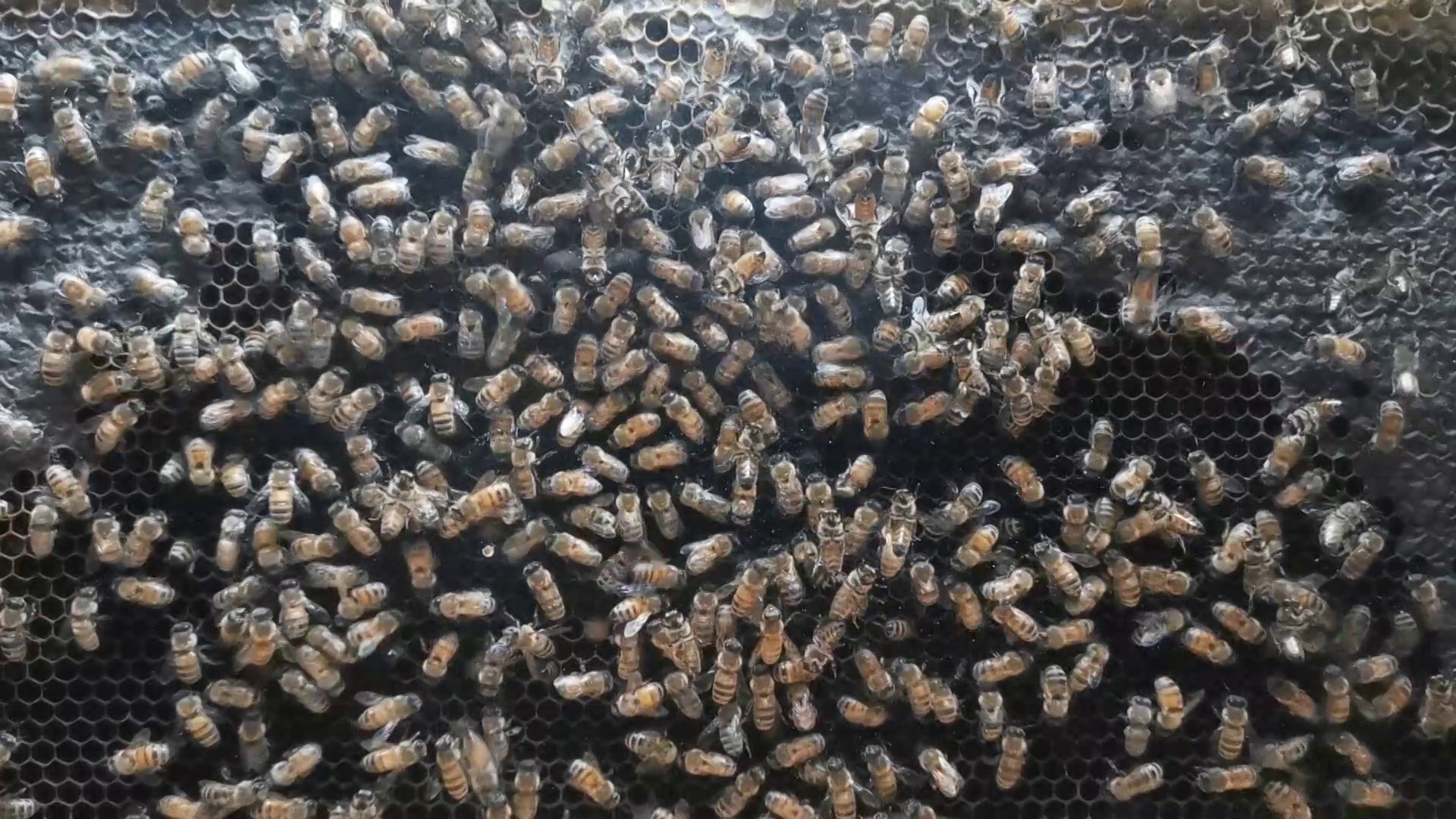

Supplement: Supplementary file 1 — Supplementary Information. [file 41598_2023_44718_MOESM1_ESM.zip › Dataset/test set-system_evaluation/test_set_15fps/023.jpg]

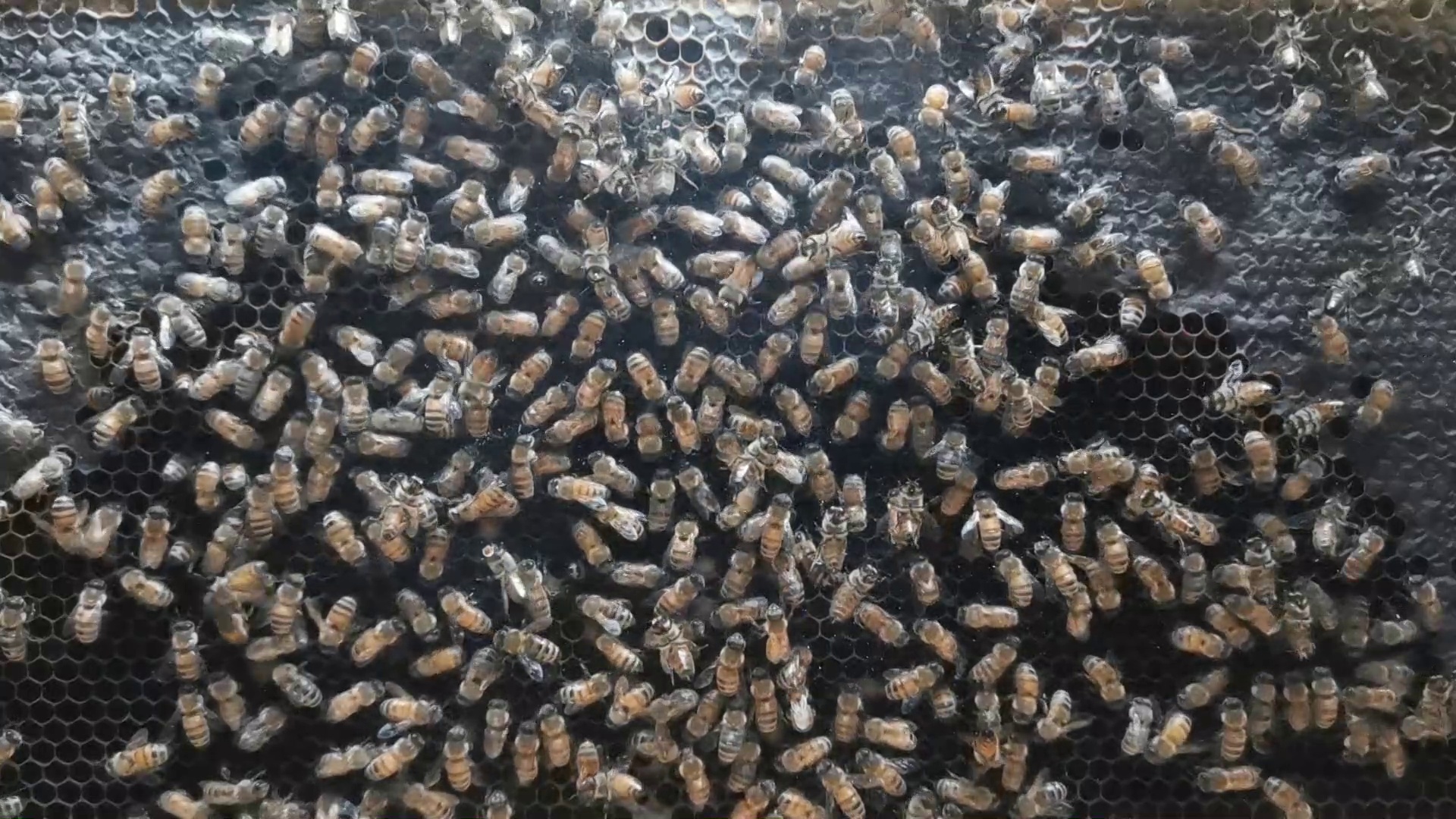

Supplement: Supplementary file 1 — Supplementary Information. [file 41598_2023_44718_MOESM1_ESM.zip › Dataset/test set-system_evaluation/test_set_15fps/082.jpg]

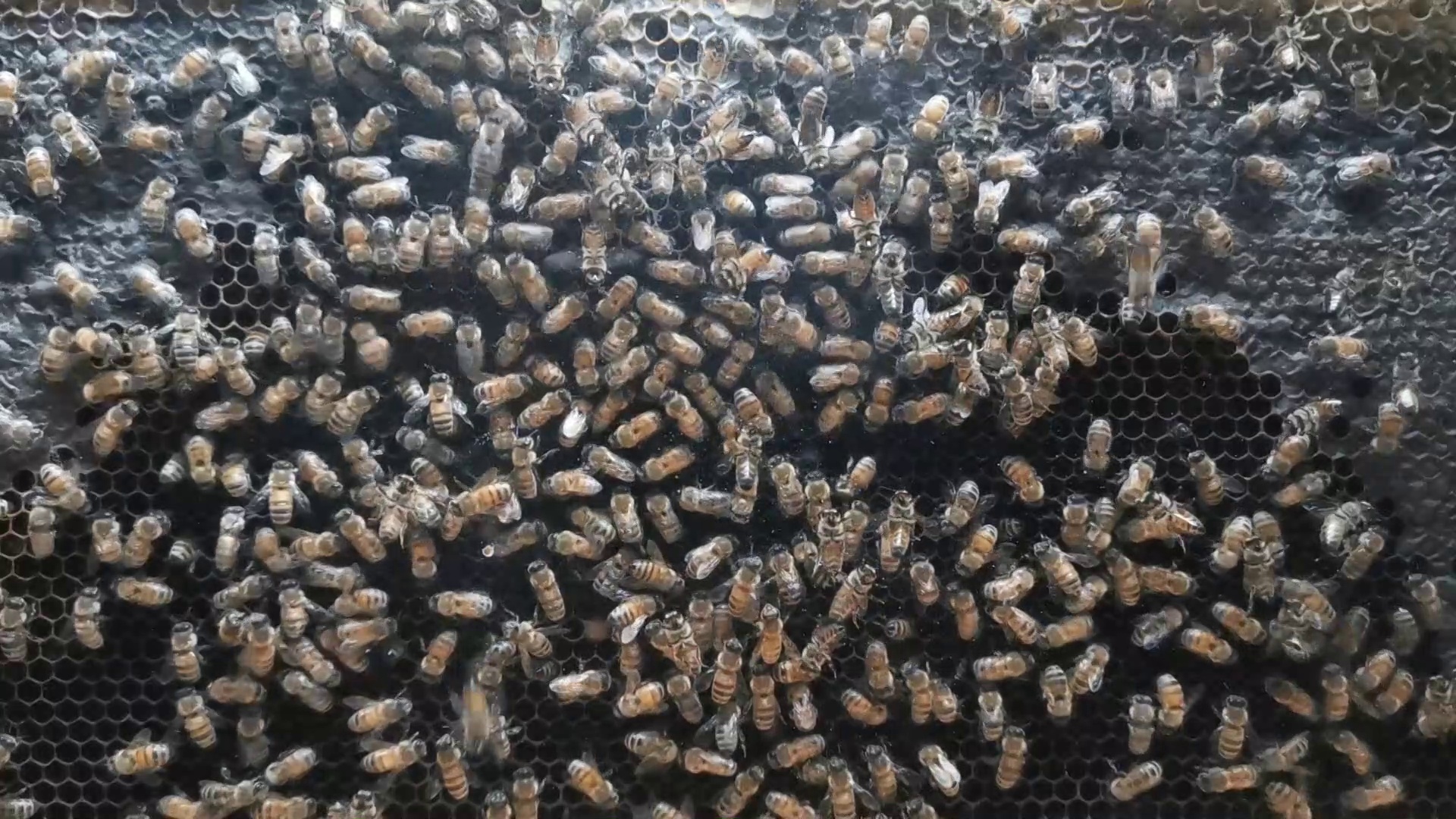

Supplement: Supplementary file 1 — Supplementary Information. [file 41598_2023_44718_MOESM1_ESM.zip › Dataset/test set-system_evaluation/test_set_15fps/020.jpg]

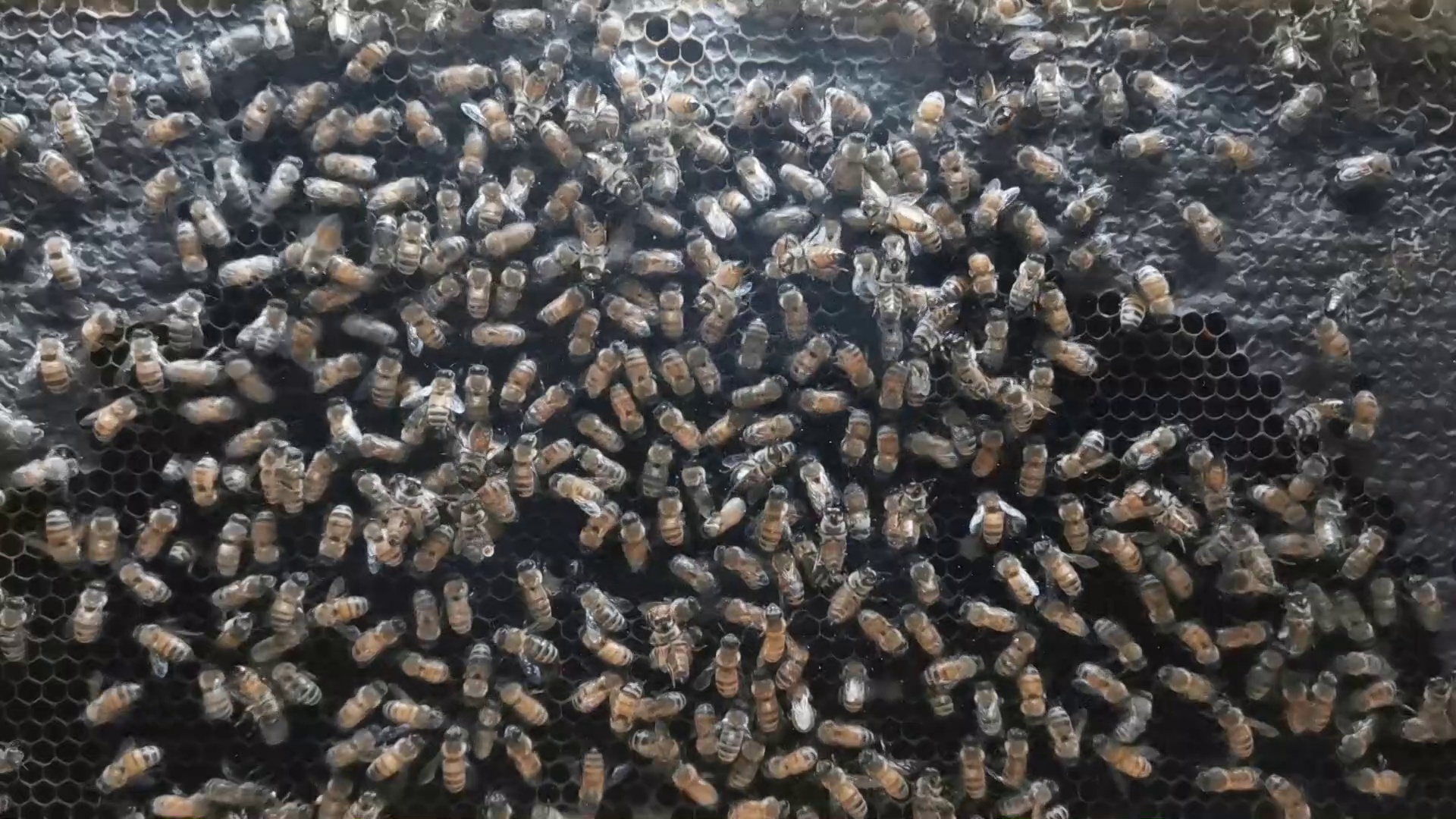

Supplement: Supplementary file 1 — Supplementary Information. [file 41598_2023_44718_MOESM1_ESM.zip › Dataset/test set-system_evaluation/test_set_15fps/104.jpg]

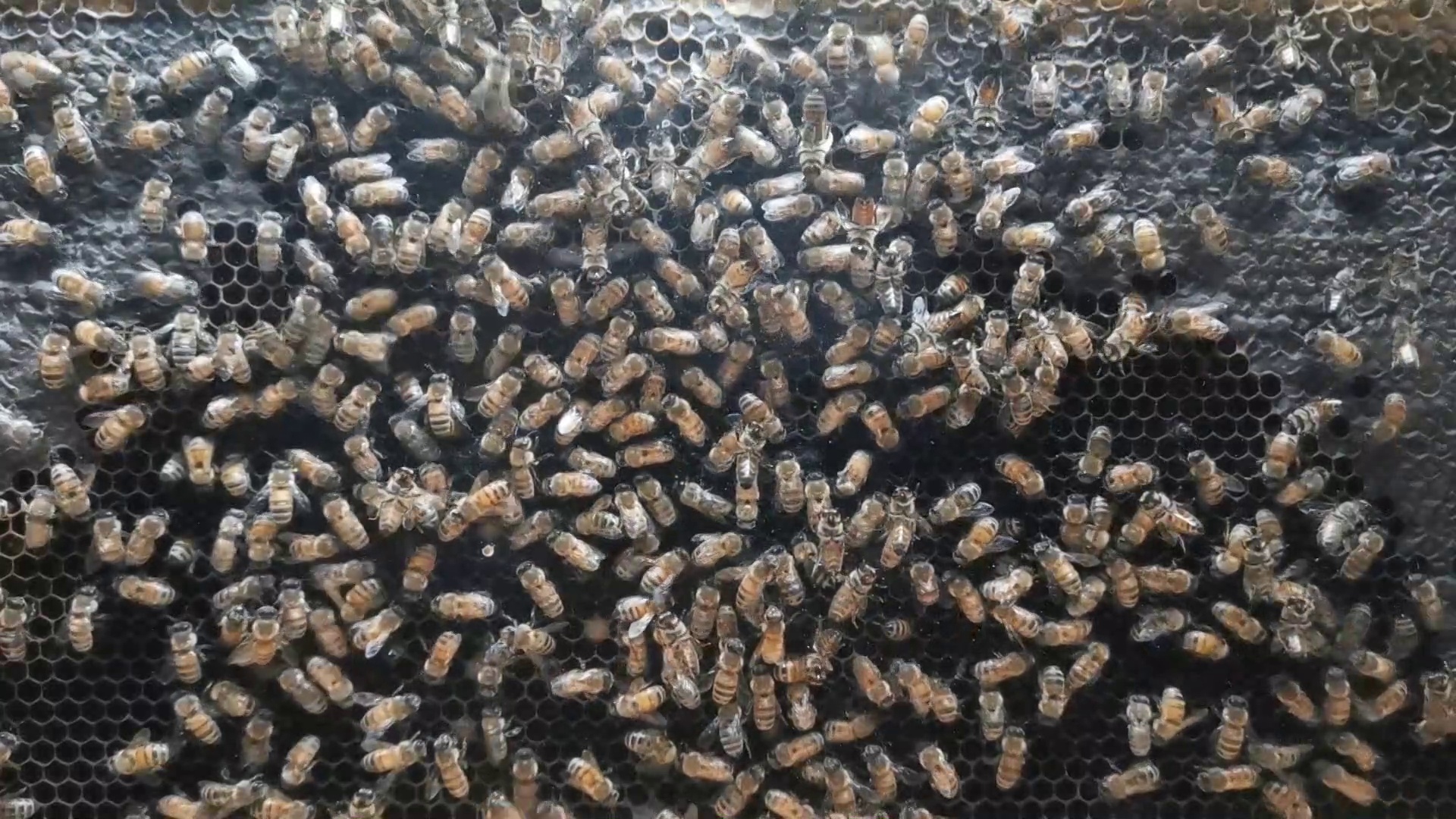

Supplement: Supplementary file 1 — Supplementary Information. [file 41598_2023_44718_MOESM1_ESM.zip › Dataset/test set-system_evaluation/test_set_15fps/027.jpg]

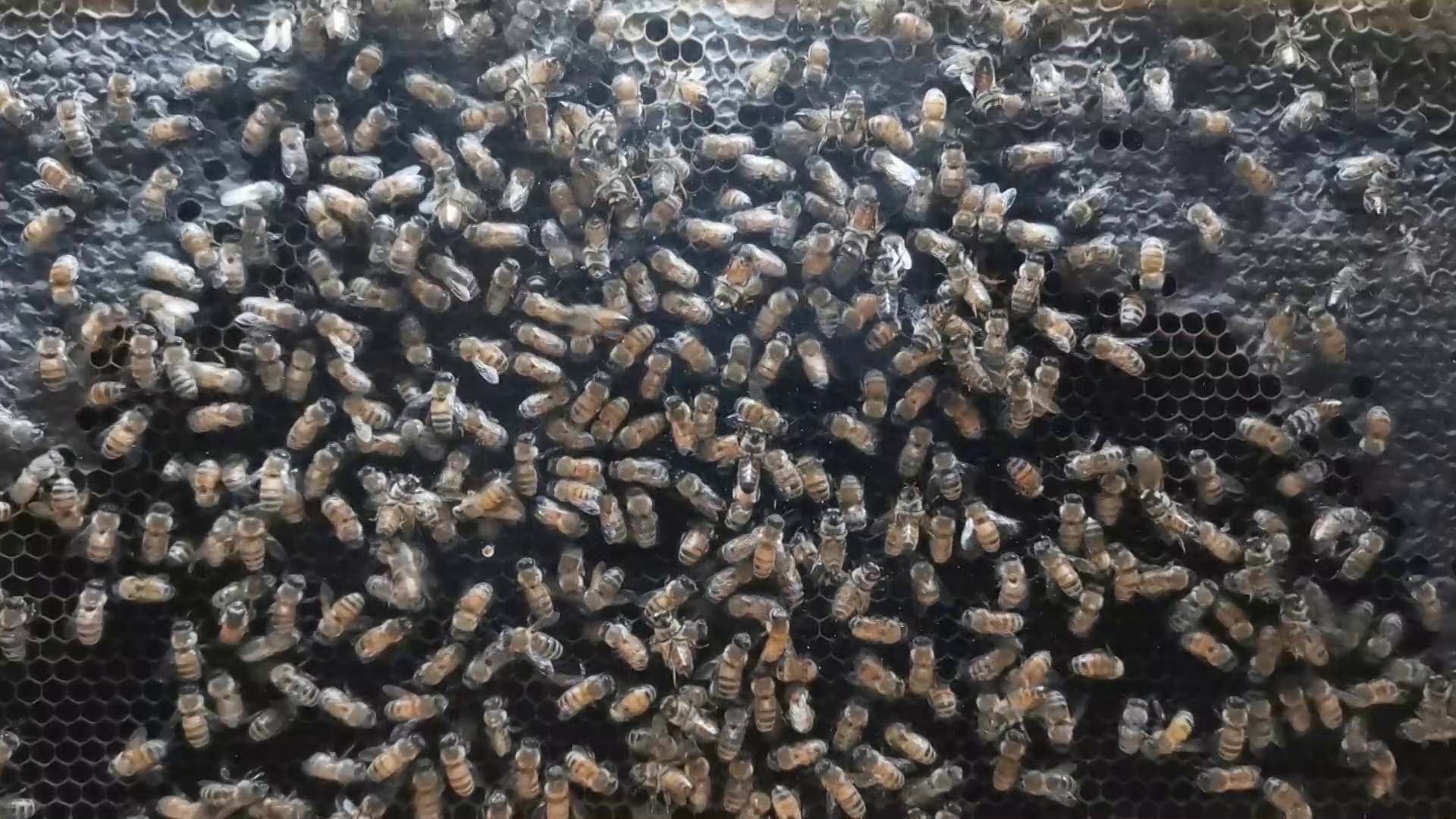

Supplement: Supplementary file 1 — Supplementary Information. [file 41598_2023_44718_MOESM1_ESM.zip › Dataset/test set-system_evaluation/test_set_15fps/062.jpg]

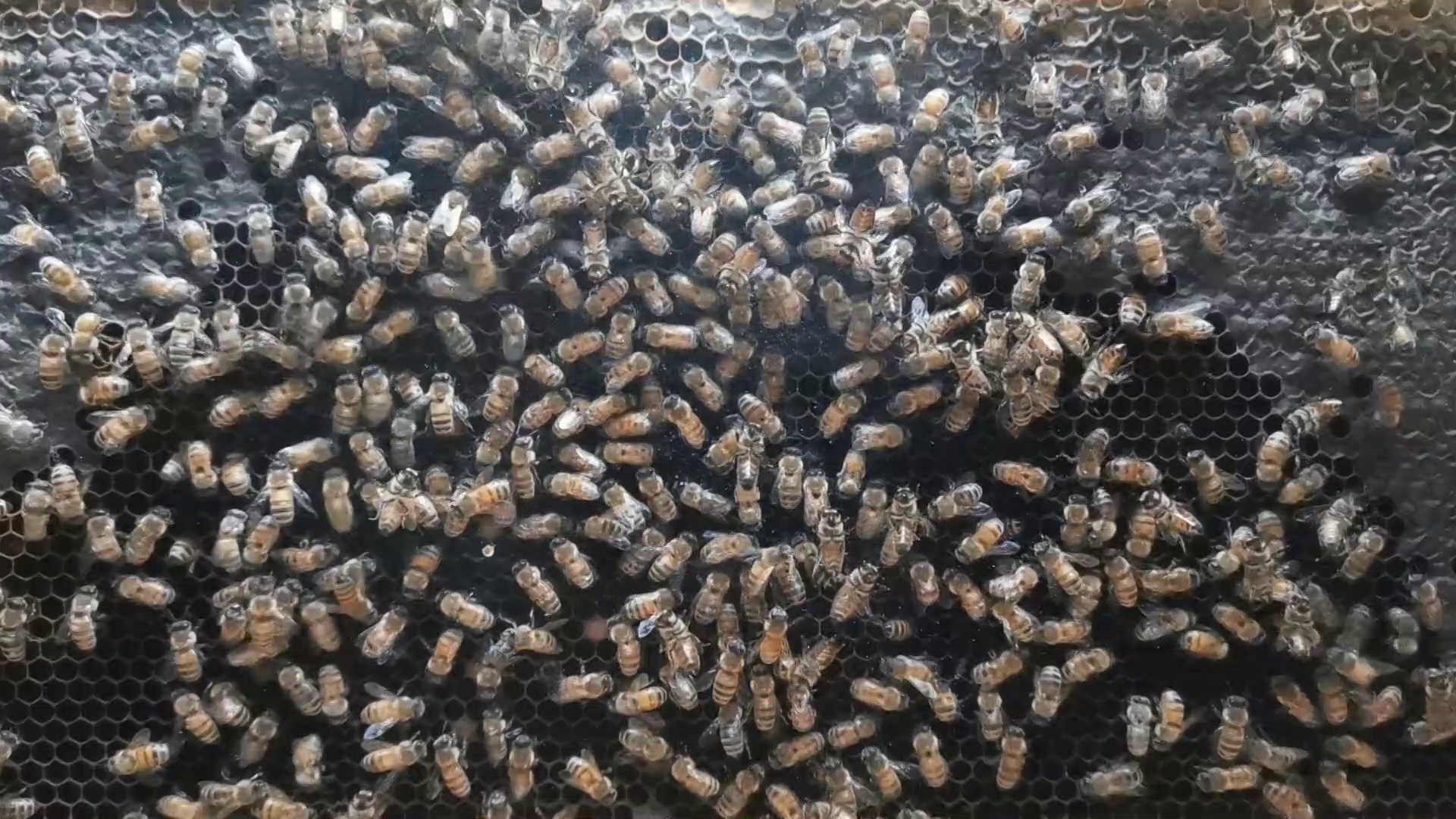

Supplement: Supplementary file 1 — Supplementary Information. [file 41598_2023_44718_MOESM1_ESM.zip › Dataset/test set-system_evaluation/test_set_15fps/033.jpg]

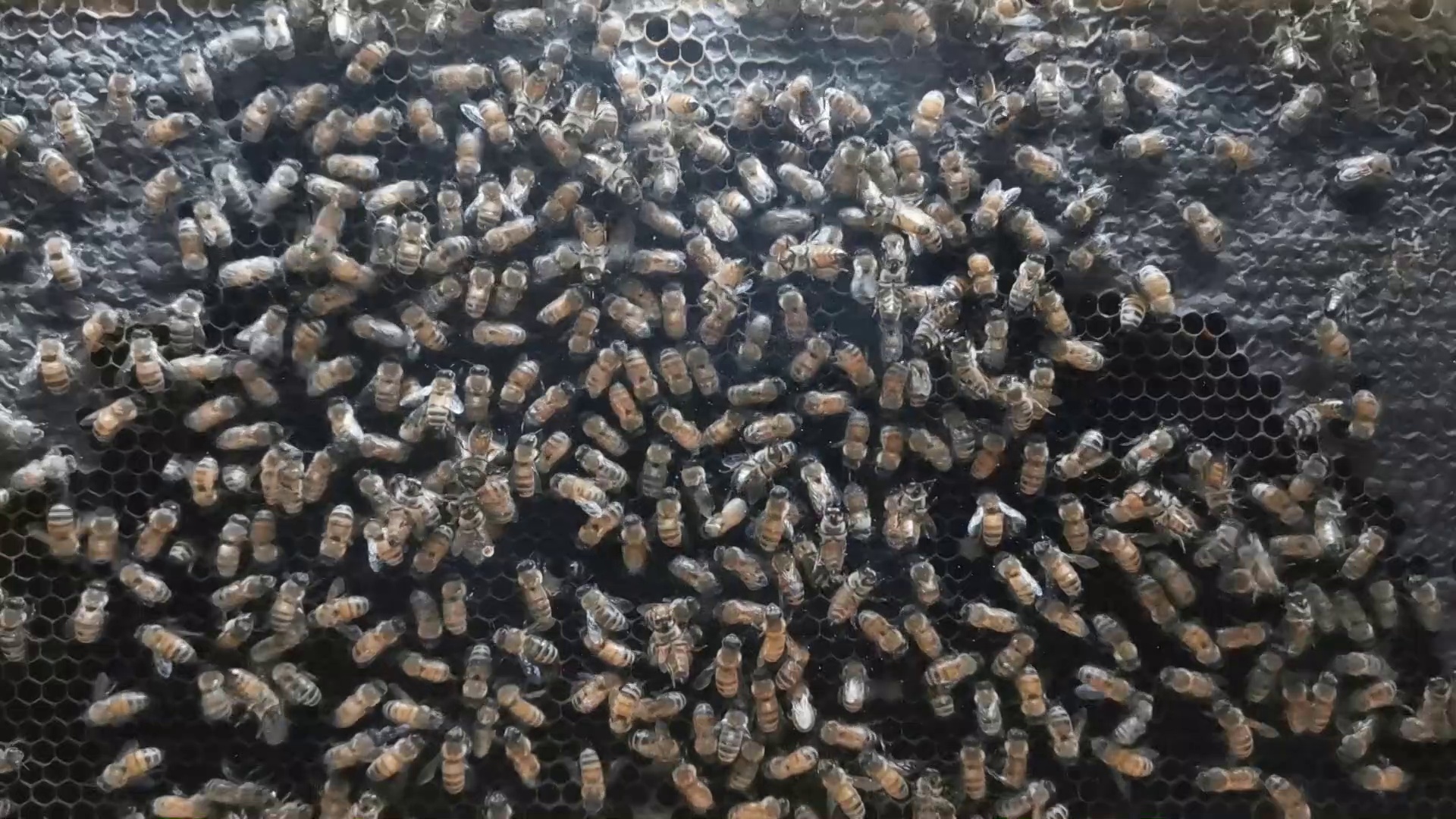

Supplement: Supplementary file 1 — Supplementary Information. [file 41598_2023_44718_MOESM1_ESM.zip › Dataset/test set-system_evaluation/test_set_15fps/103.jpg]

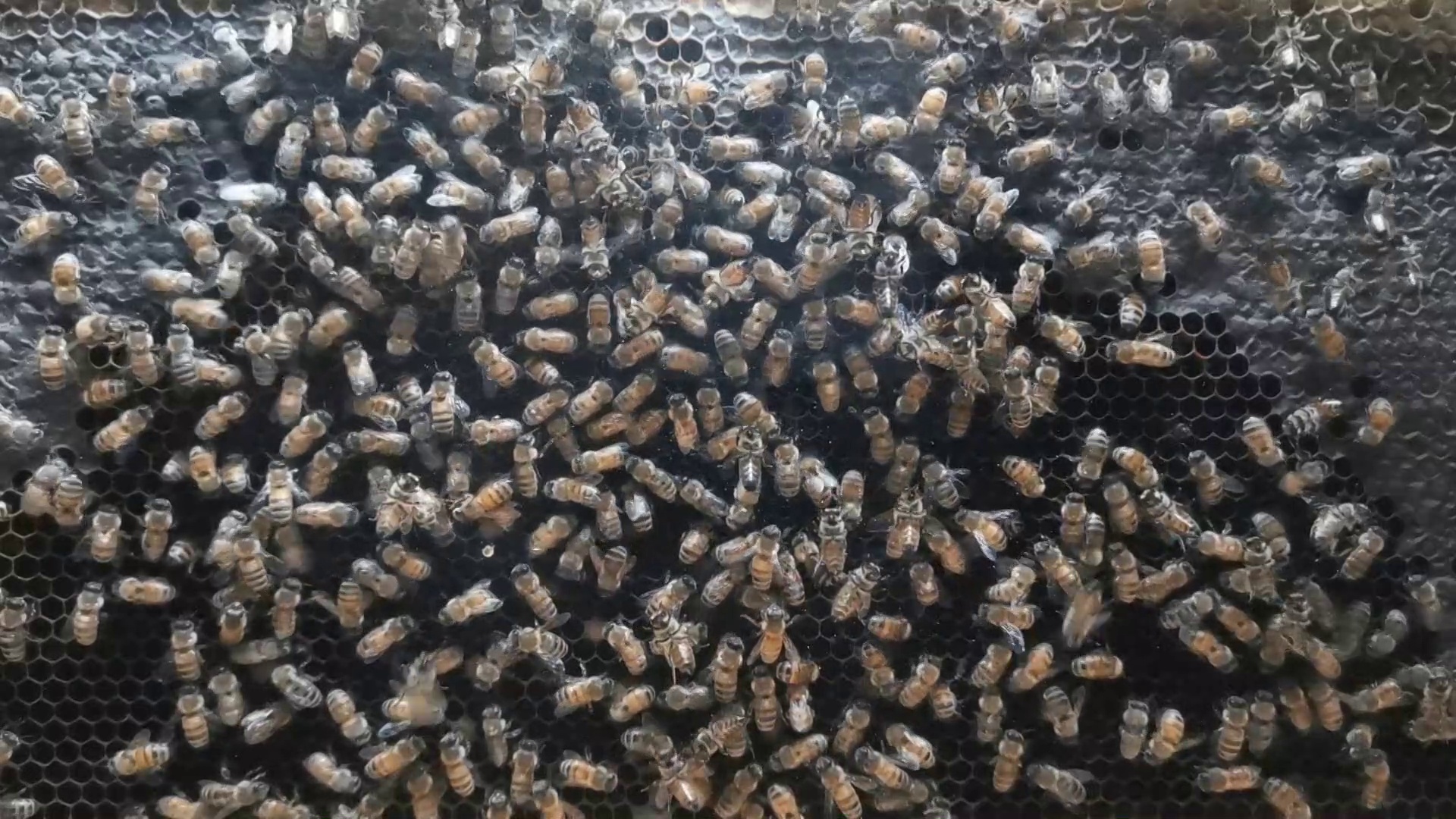

Supplement: Supplementary file 1 — Supplementary Information. [file 41598_2023_44718_MOESM1_ESM.zip › Dataset/test set-system_evaluation/test_set_15fps/054.jpg]

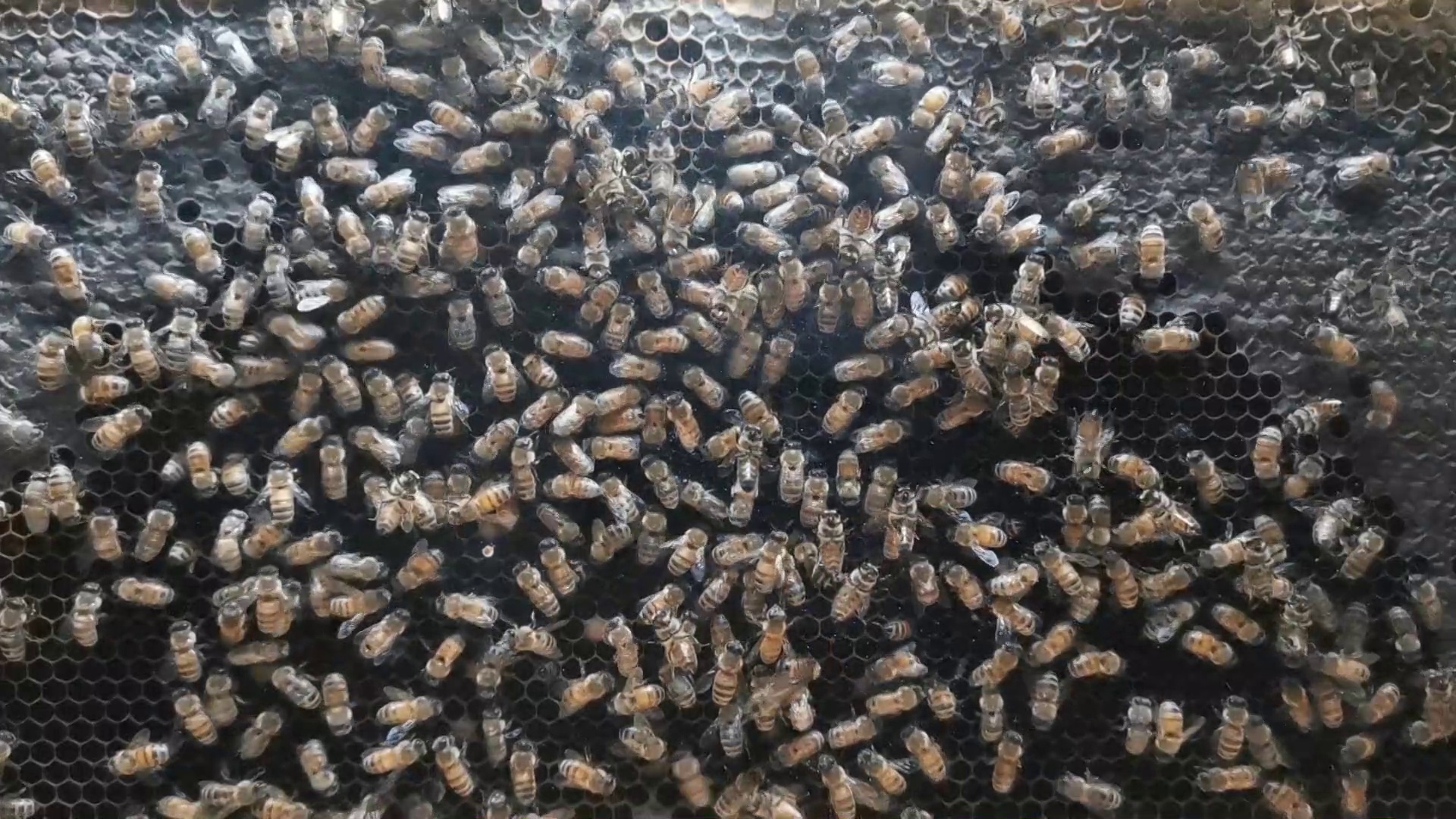

Supplement: Supplementary file 1 — Supplementary Information. [file 41598_2023_44718_MOESM1_ESM.zip › Dataset/test set-system_evaluation/test_set_15fps/040.jpg]

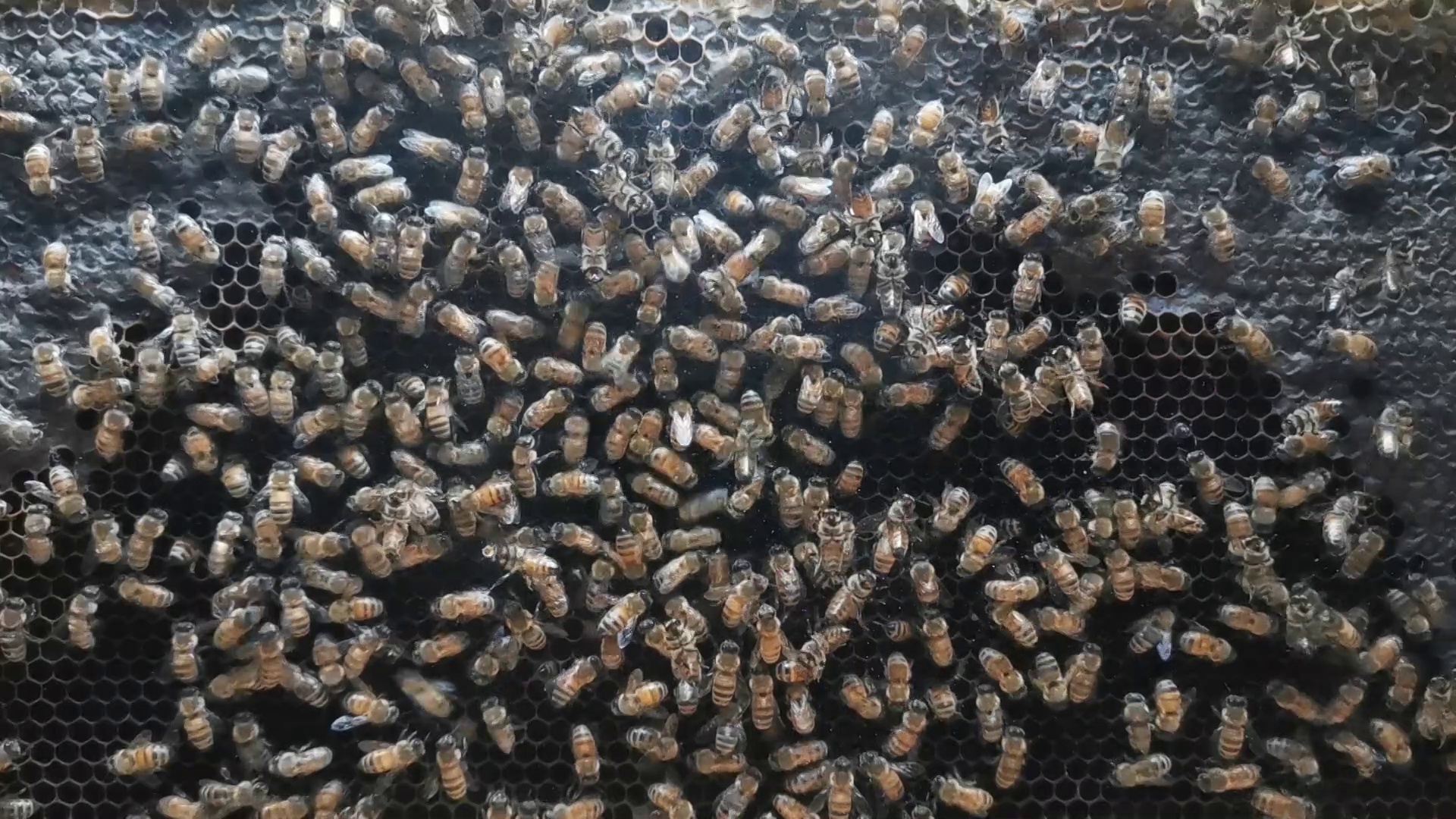

Supplement: Supplementary file 1 — Supplementary Information. [file 41598_2023_44718_MOESM1_ESM.zip › Dataset/test set-system_evaluation/test_set_15fps/003.jpg]

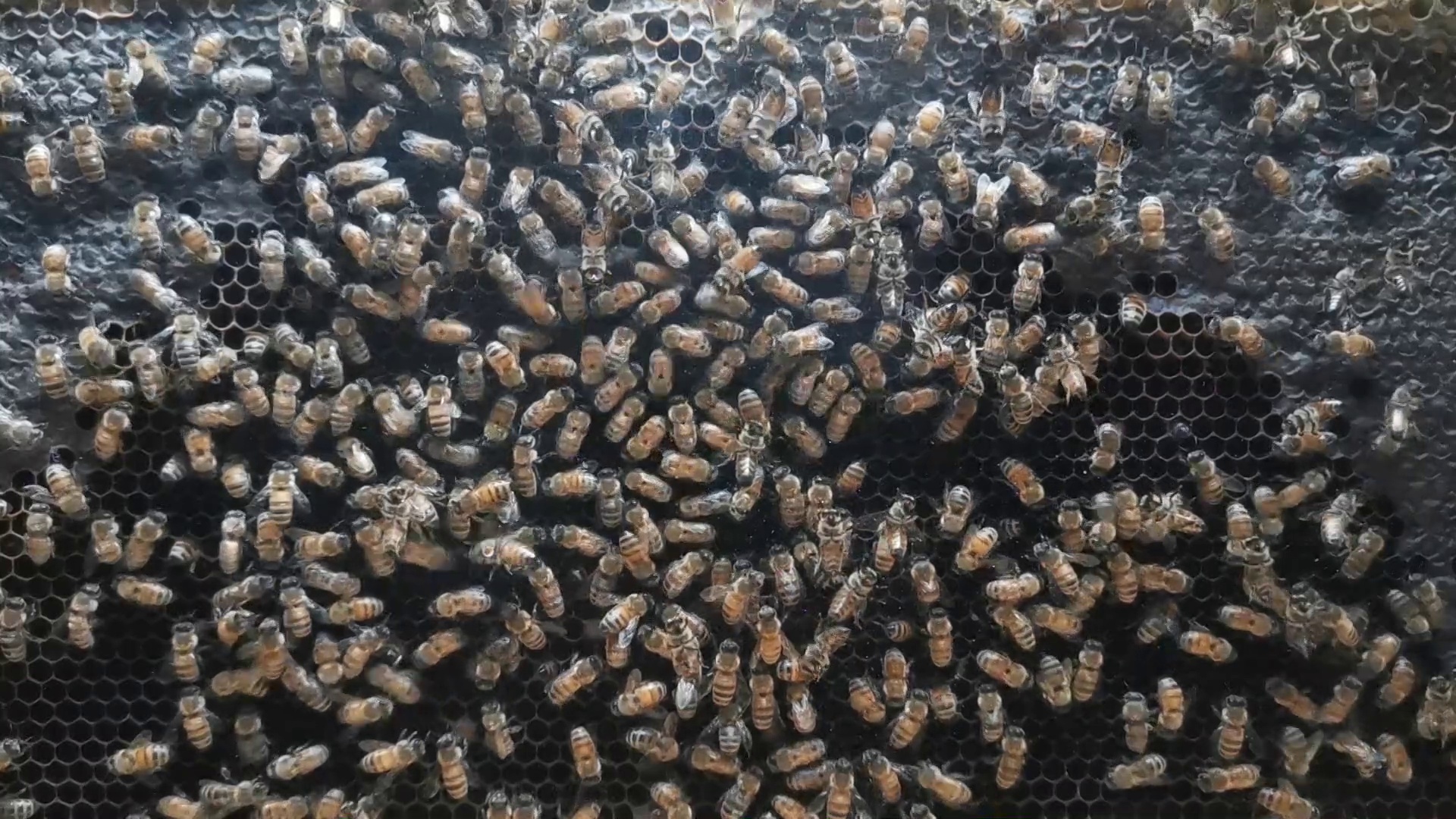

Supplement: Supplementary file 1 — Supplementary Information. [file 41598_2023_44718_MOESM1_ESM.zip › Dataset/test set-system_evaluation/test_set_15fps/007.jpg]

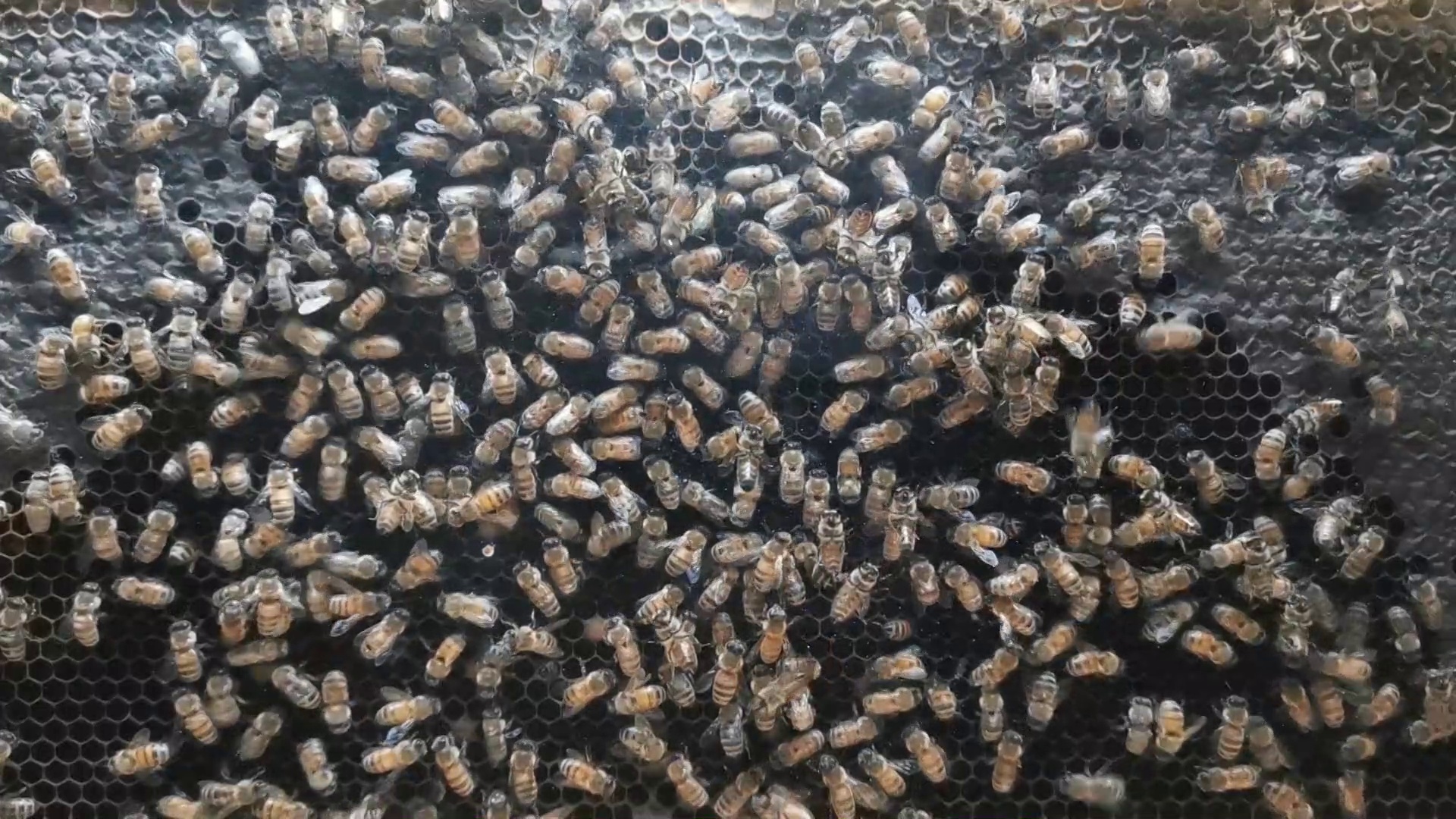

Supplement: Supplementary file 1 — Supplementary Information. [file 41598_2023_44718_MOESM1_ESM.zip › Dataset/test set-system_evaluation/test_set_15fps/039.jpg]

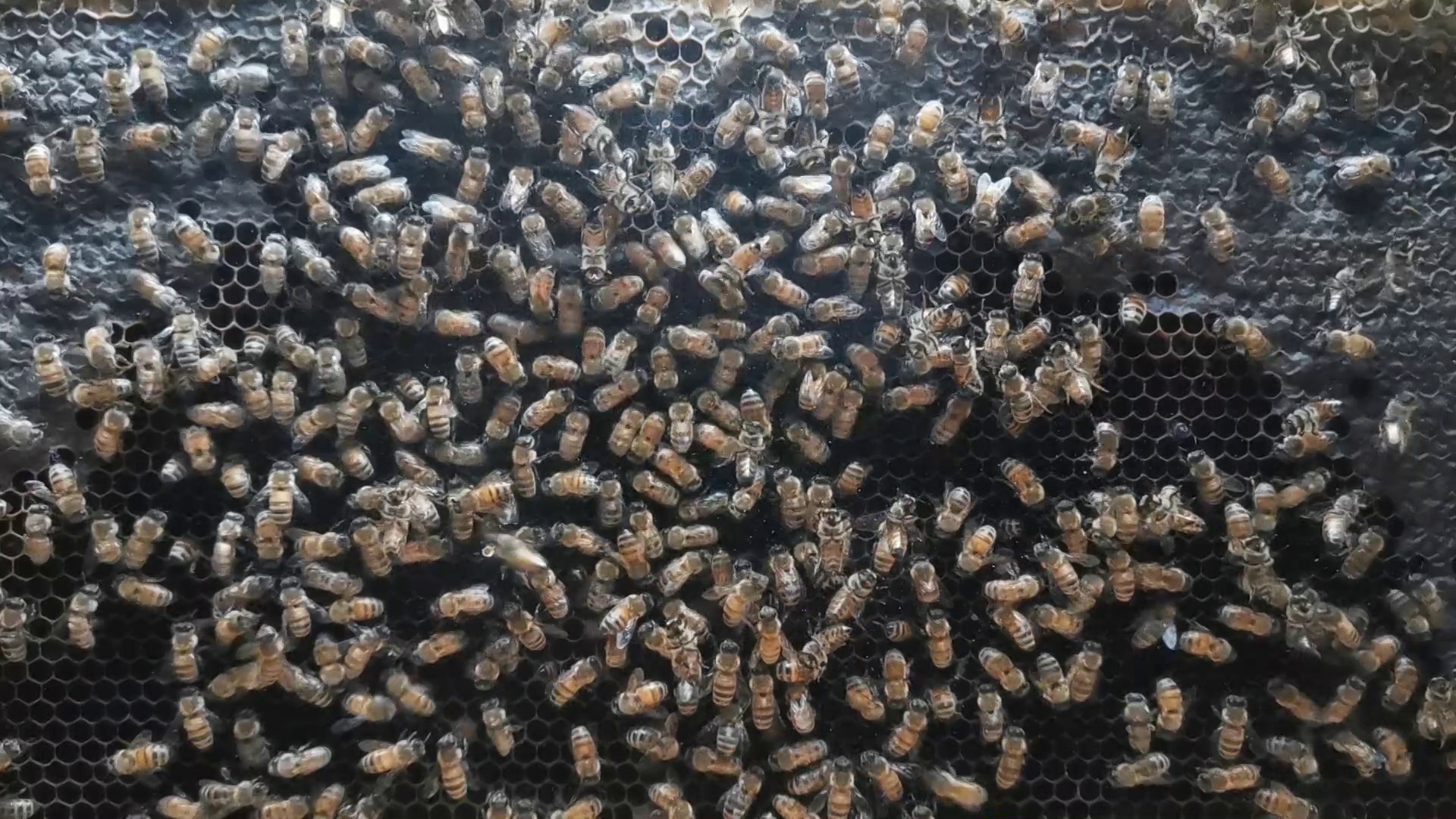

Supplement: Supplementary file 1 — Supplementary Information. [file 41598_2023_44718_MOESM1_ESM.zip › Dataset/test set-system_evaluation/test_set_15fps/005.jpg]

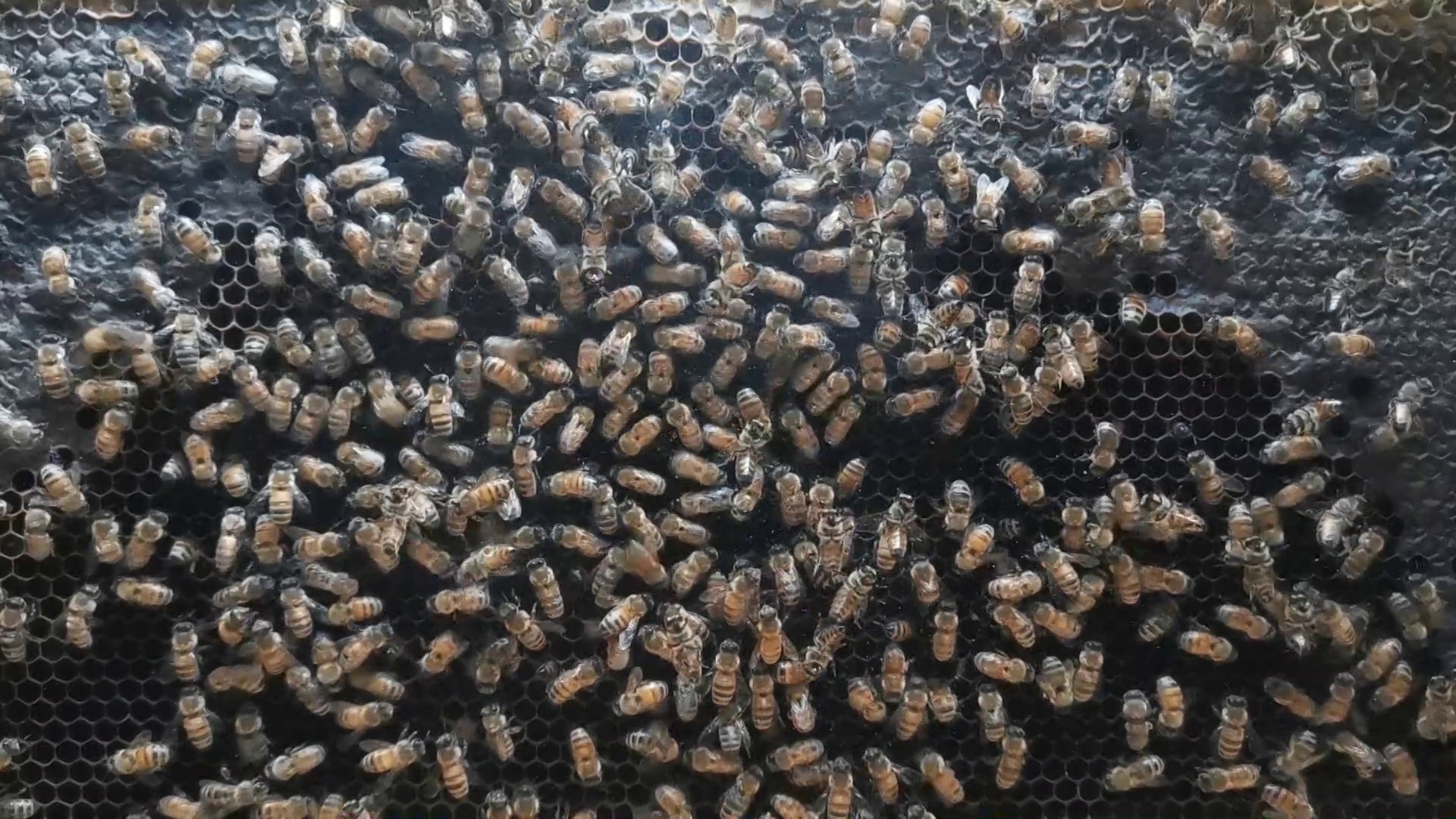

Supplement: Supplementary file 1 — Supplementary Information. [file 41598_2023_44718_MOESM1_ESM.zip › Dataset/test set-system_evaluation/test_set_15fps/010.jpg]

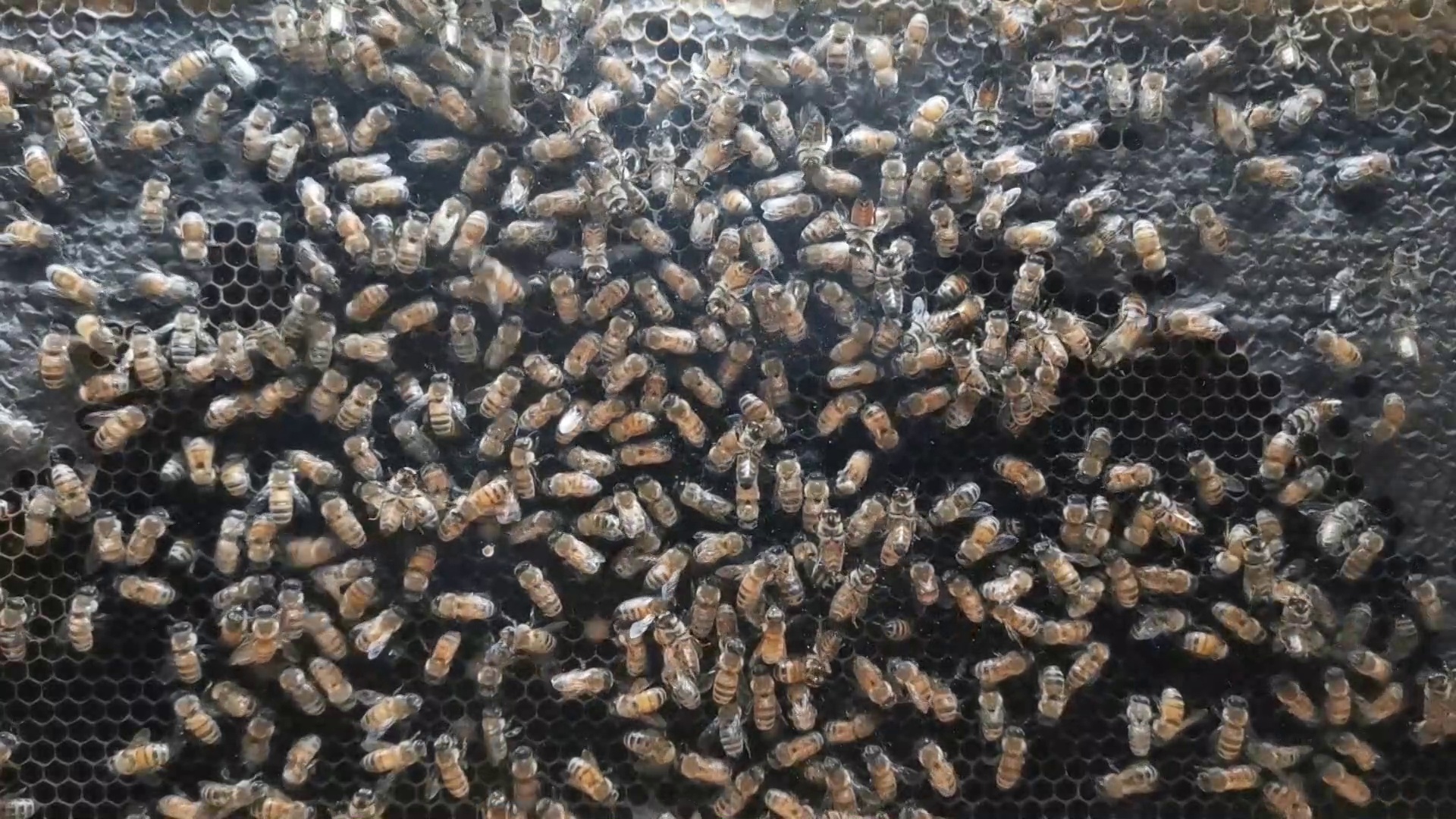

Supplement: Supplementary file 1 — Supplementary Information. [file 41598_2023_44718_MOESM1_ESM.zip › Dataset/test set-system_evaluation/test_set_15fps/028.jpg]

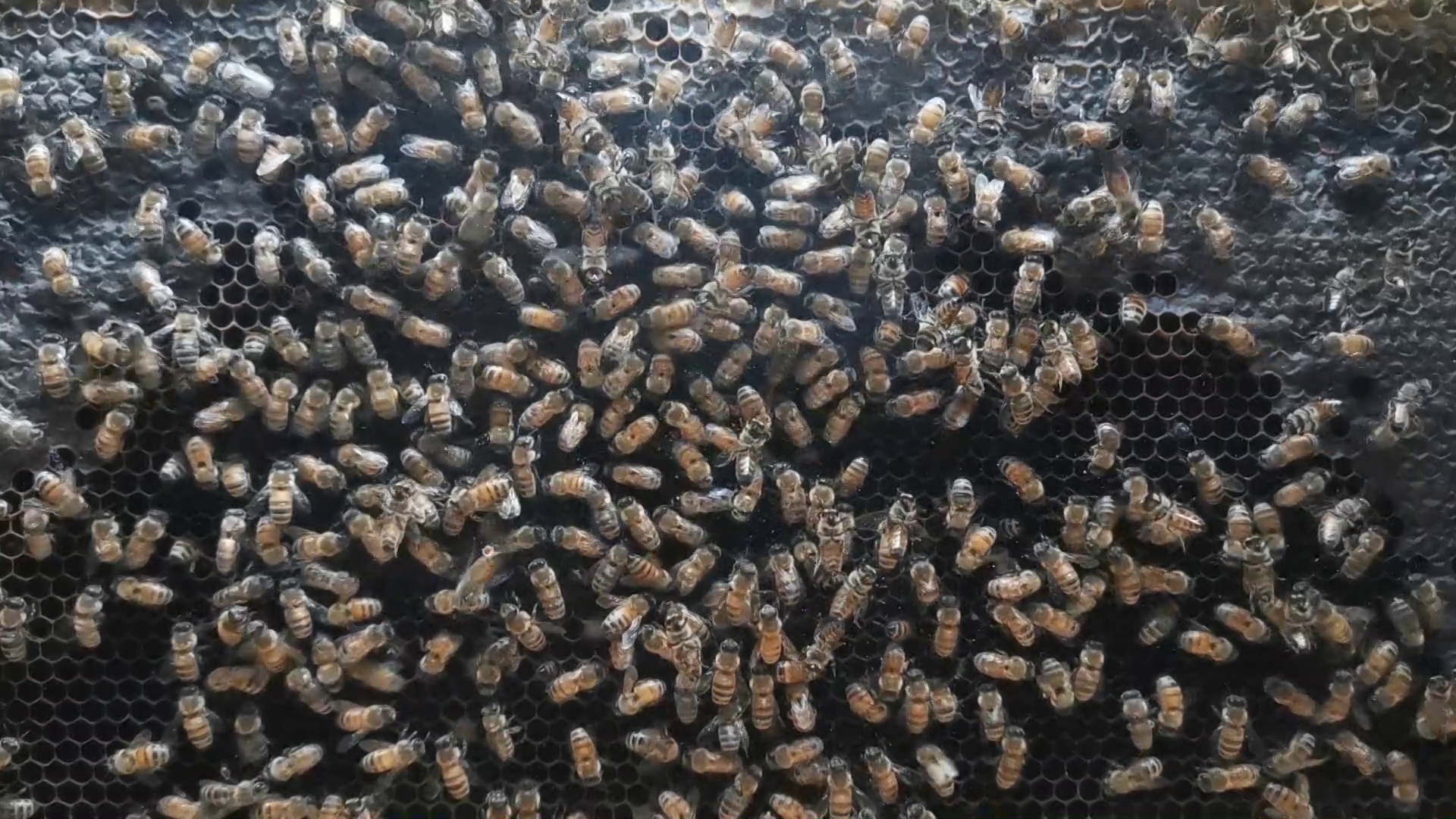

Supplement: Supplementary file 1 — Supplementary Information. [file 41598_2023_44718_MOESM1_ESM.zip › Dataset/test set-system_evaluation/test_set_15fps/012.jpg]

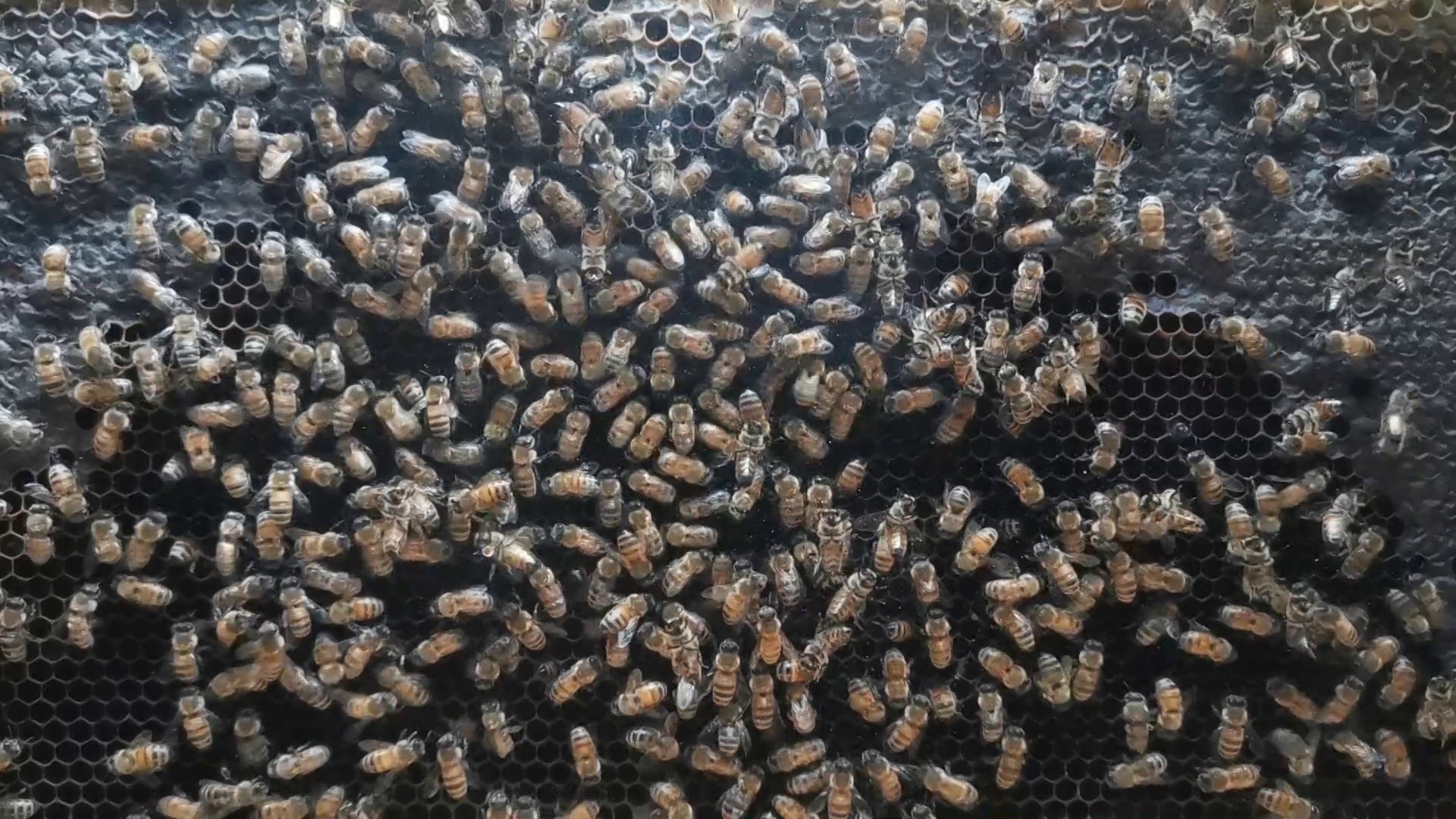

Supplement: Supplementary file 1 — Supplementary Information. [file 41598_2023_44718_MOESM1_ESM.zip › Dataset/test set-system_evaluation/test_set_15fps/006.jpg]

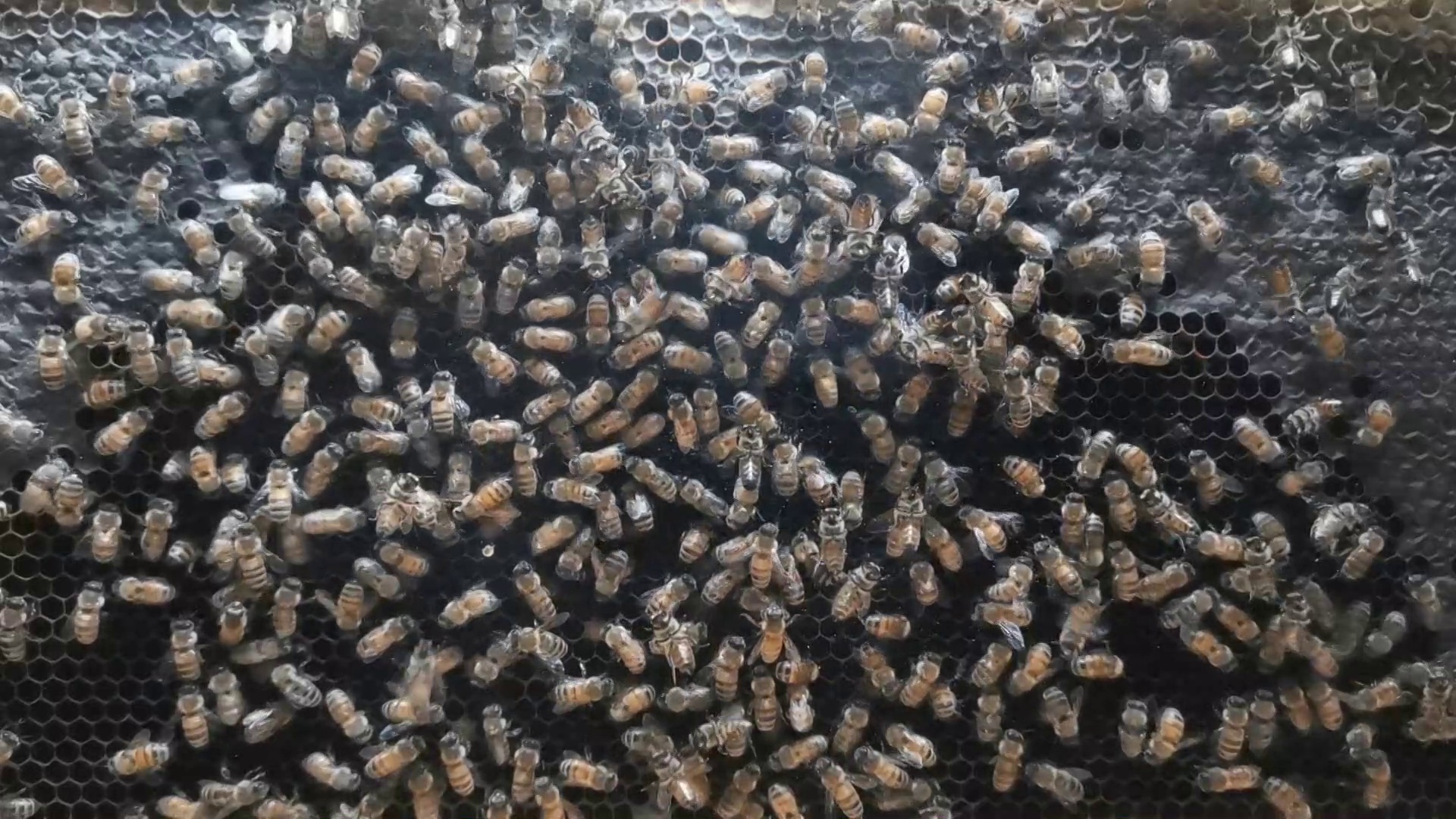

Supplement: Supplementary file 1 — Supplementary Information. [file 41598_2023_44718_MOESM1_ESM.zip › Dataset/test set-system_evaluation/test_set_15fps/055.jpg]

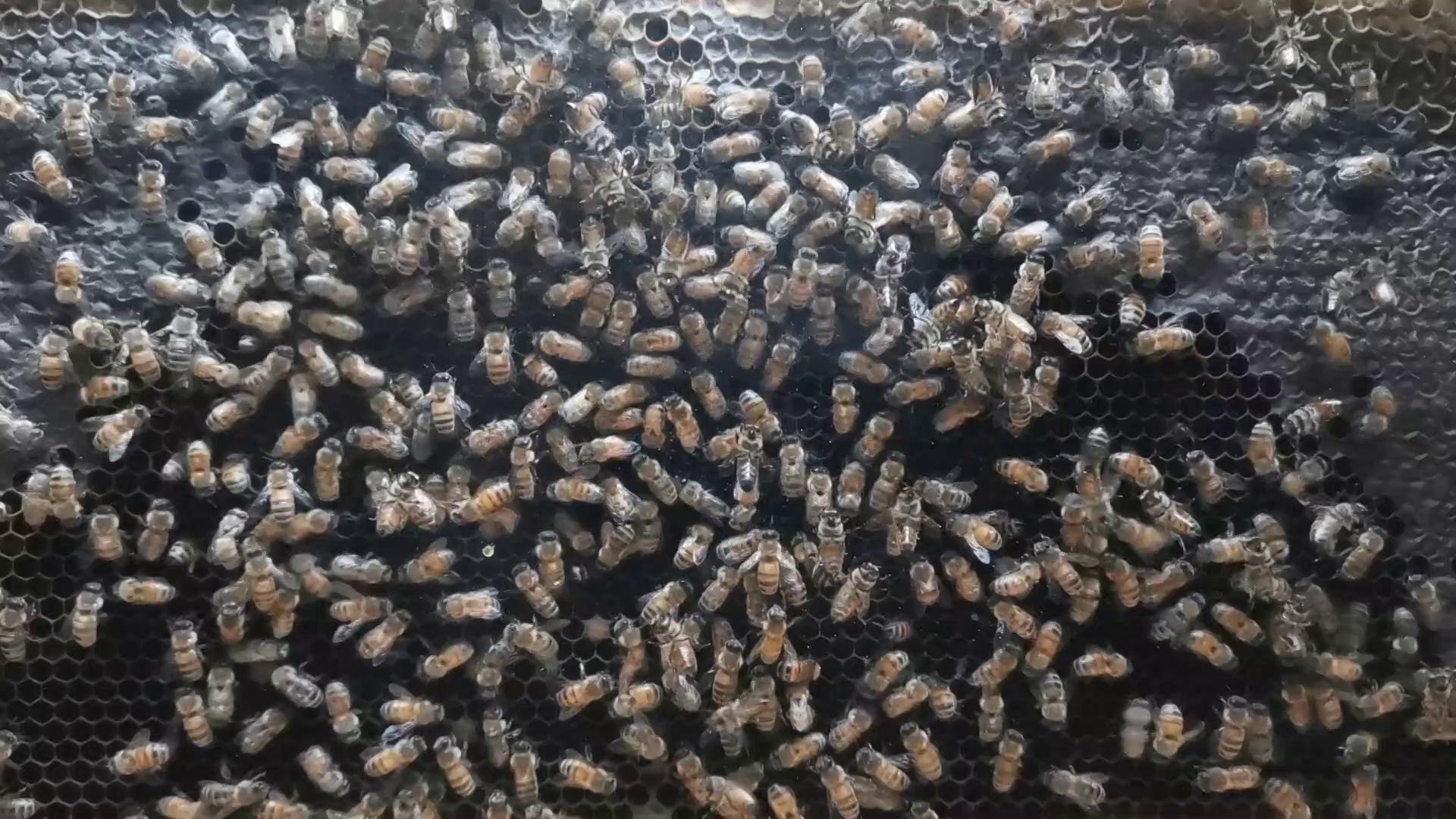

Supplement: Supplementary file 1 — Supplementary Information. [file 41598_2023_44718_MOESM1_ESM.zip › Dataset/test set-system_evaluation/test_set_15fps/045.jpg]

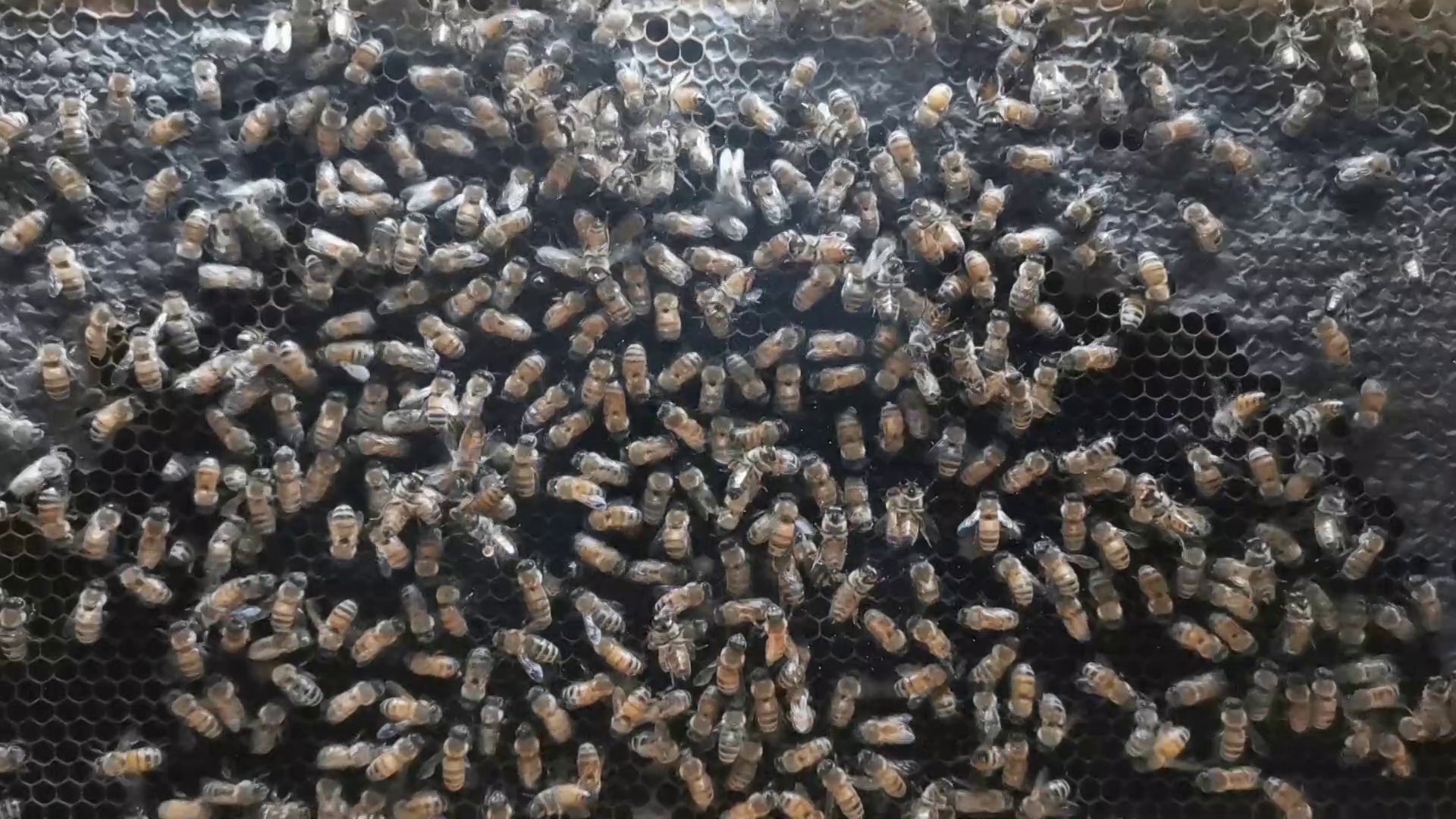

Supplement: Supplementary file 1 — Supplementary Information. [file 41598_2023_44718_MOESM1_ESM.zip › Dataset/test set-system_evaluation/test_set_15fps/089.jpg]

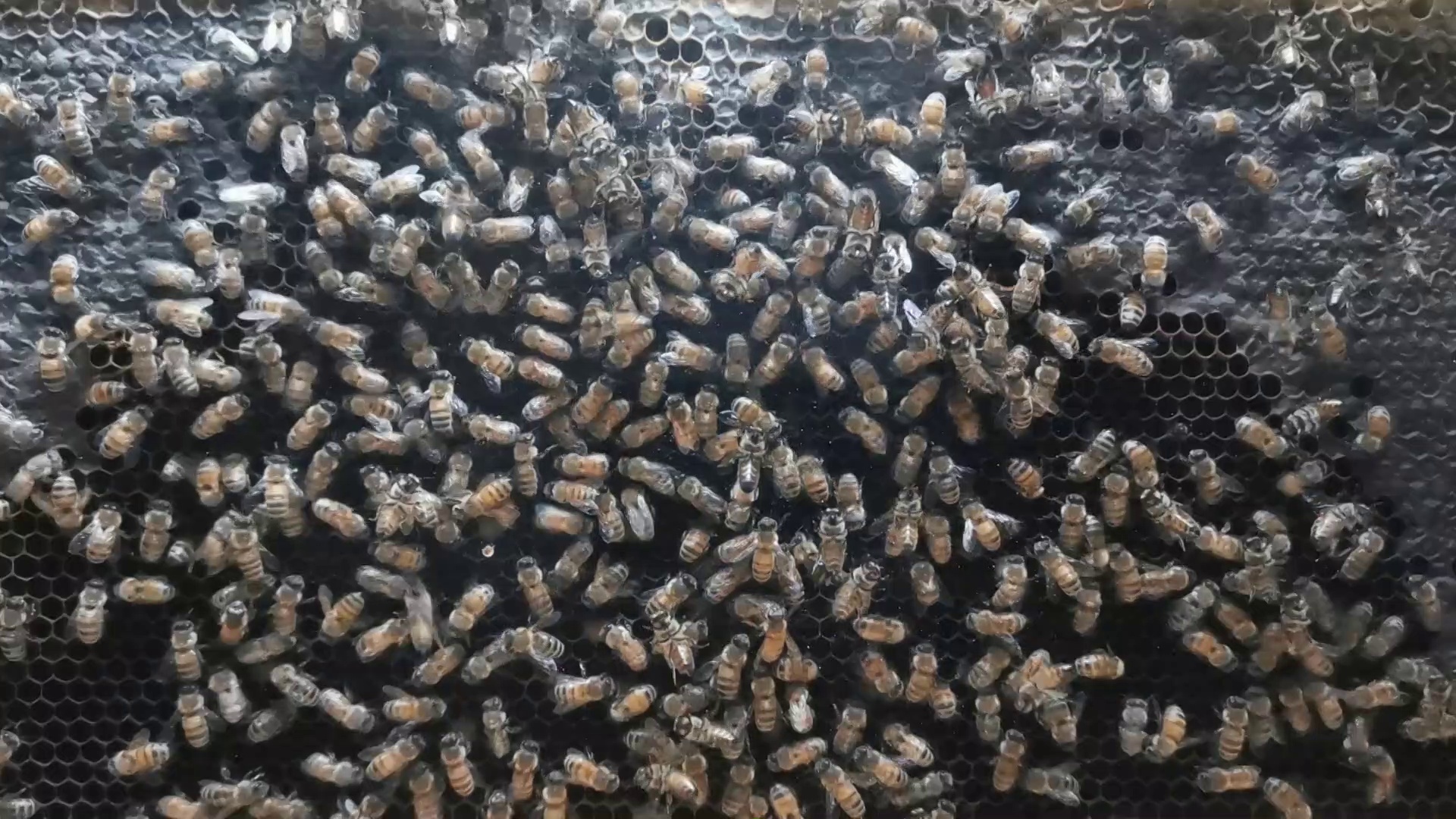

Supplement: Supplementary file 1 — Supplementary Information. [file 41598_2023_44718_MOESM1_ESM.zip › Dataset/test set-system_evaluation/test_set_15fps/059.jpg]

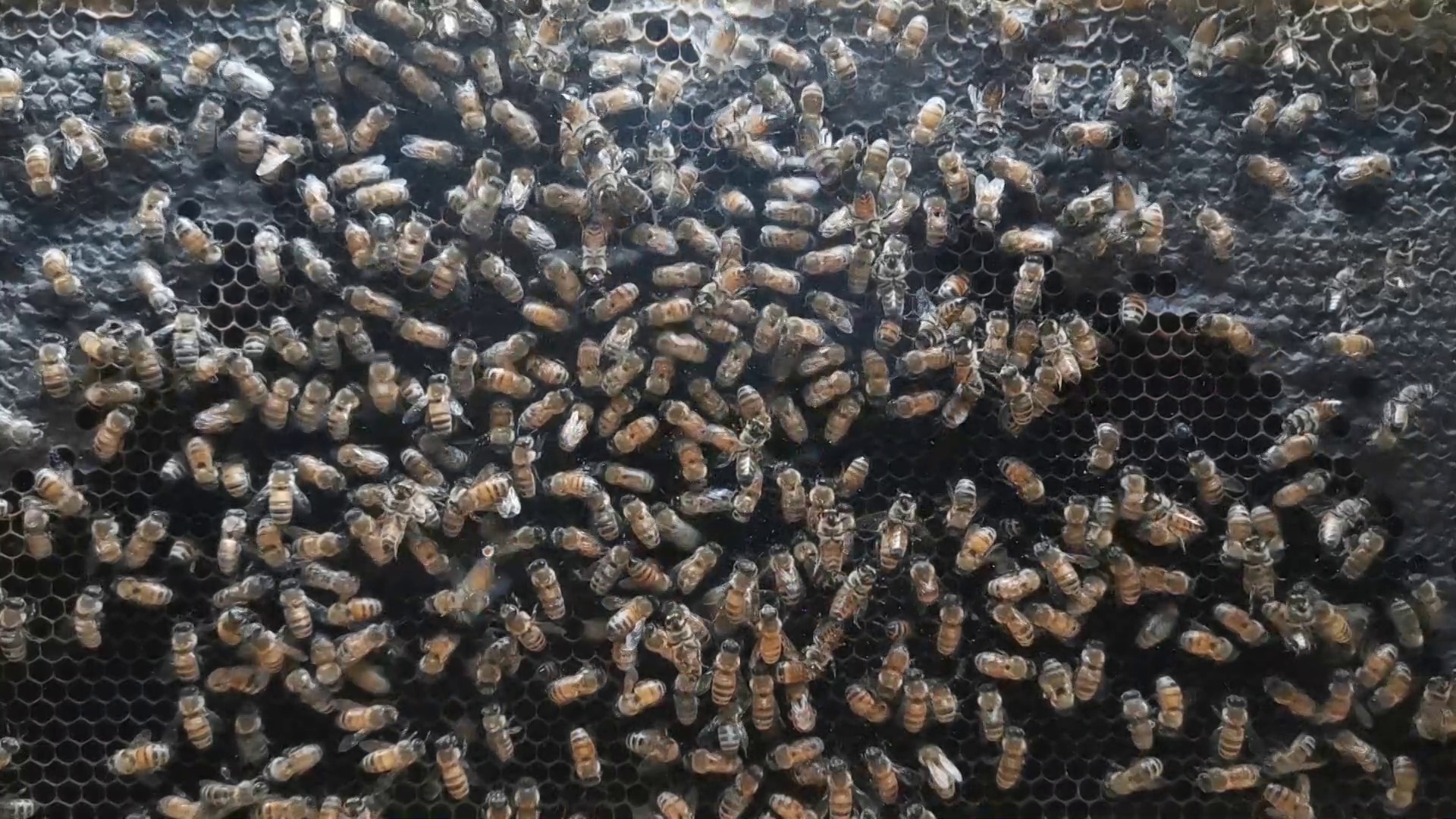

Supplement: Supplementary file 1 — Supplementary Information. [file 41598_2023_44718_MOESM1_ESM.zip › Dataset/test set-system_evaluation/test_set_15fps/013.jpg]

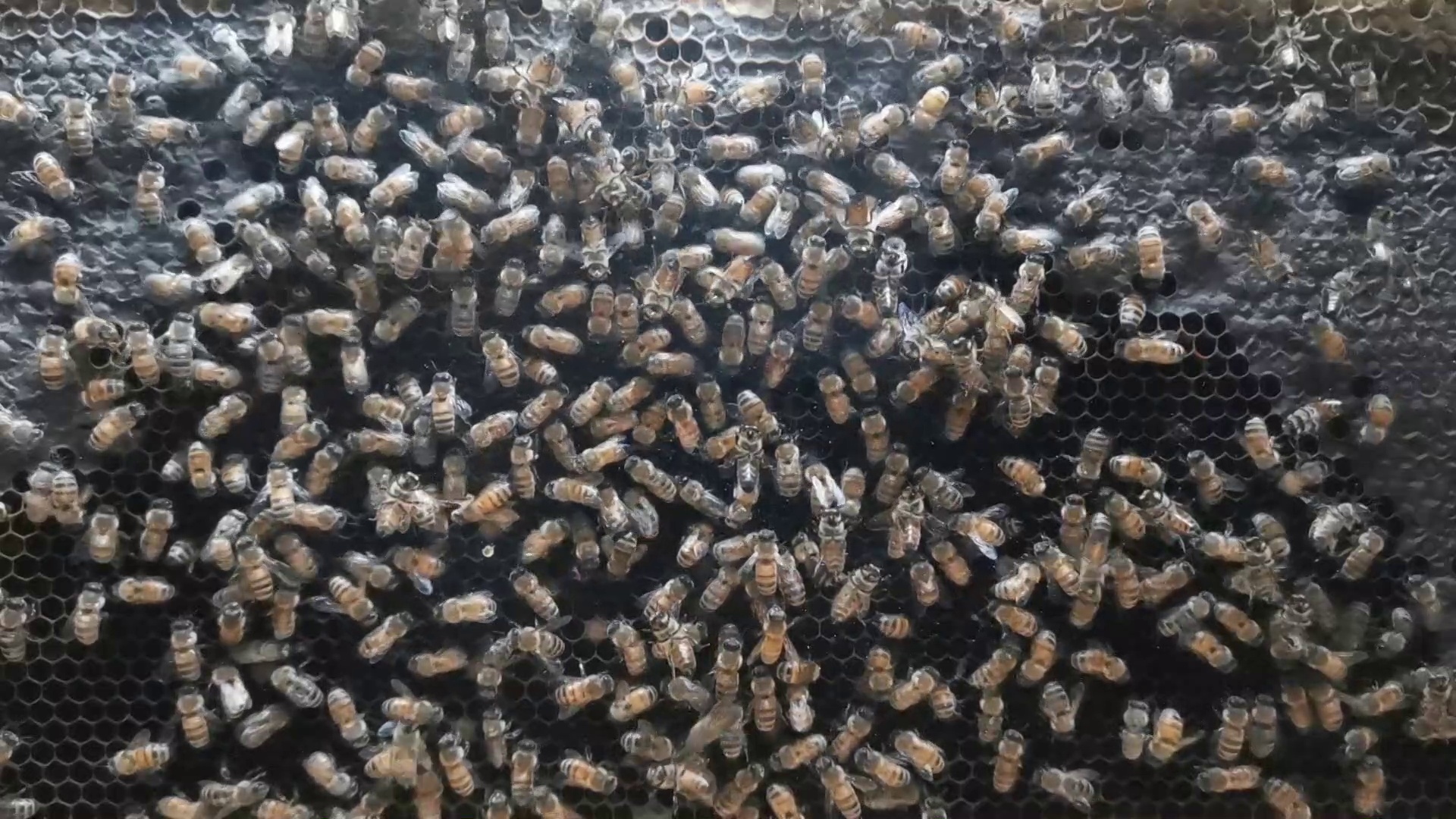

Supplement: Supplementary file 1 — Supplementary Information. [file 41598_2023_44718_MOESM1_ESM.zip › Dataset/test set-system_evaluation/test_set_15fps/050.jpg]

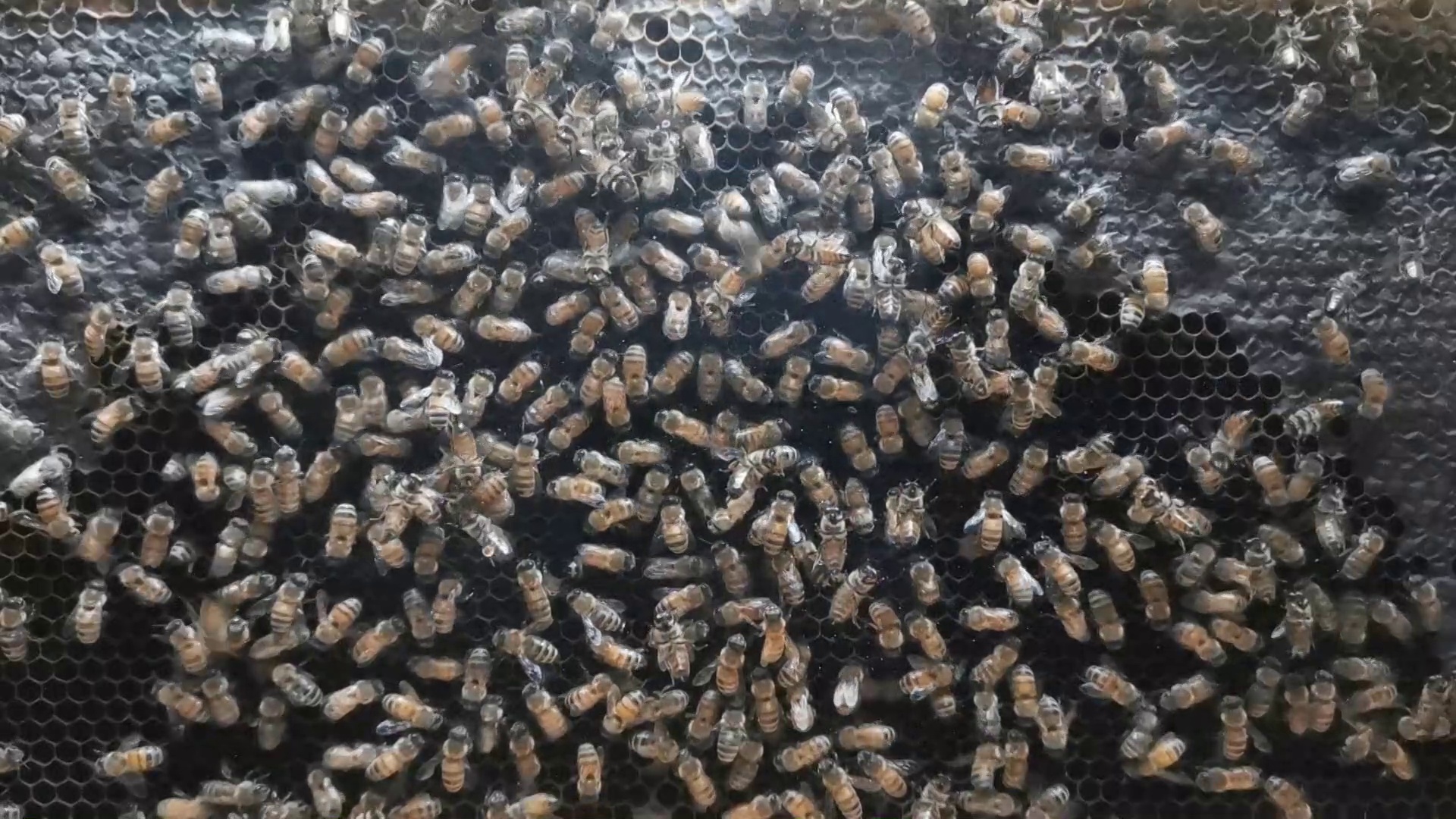

Supplement: Supplementary file 1 — Supplementary Information. [file 41598_2023_44718_MOESM1_ESM.zip › Dataset/test set-system_evaluation/test_set_15fps/093.jpg]

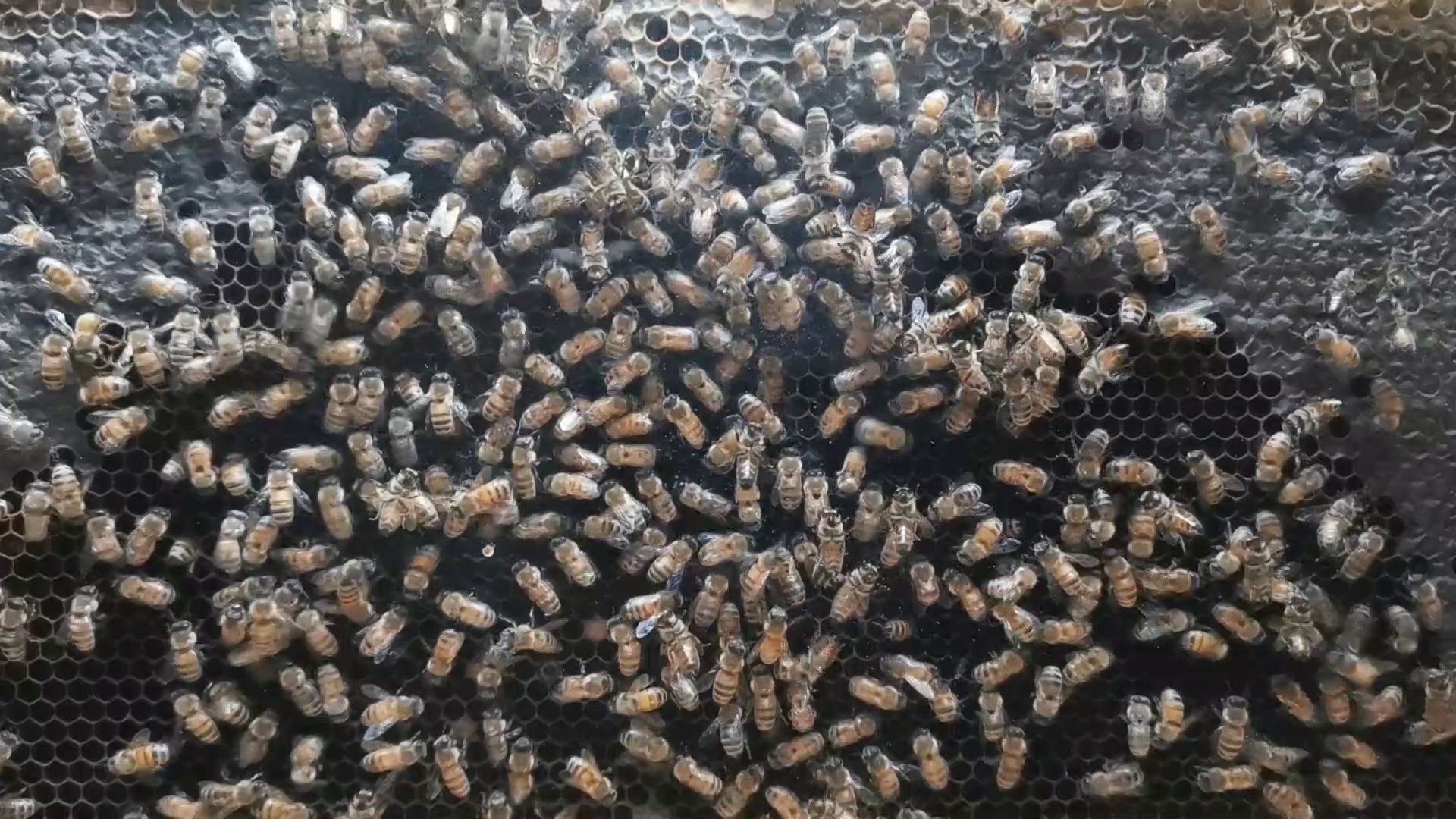

Supplement: Supplementary file 1 — Supplementary Information. [file 41598_2023_44718_MOESM1_ESM.zip › Dataset/test set-system_evaluation/test_set_15fps/032.jpg]

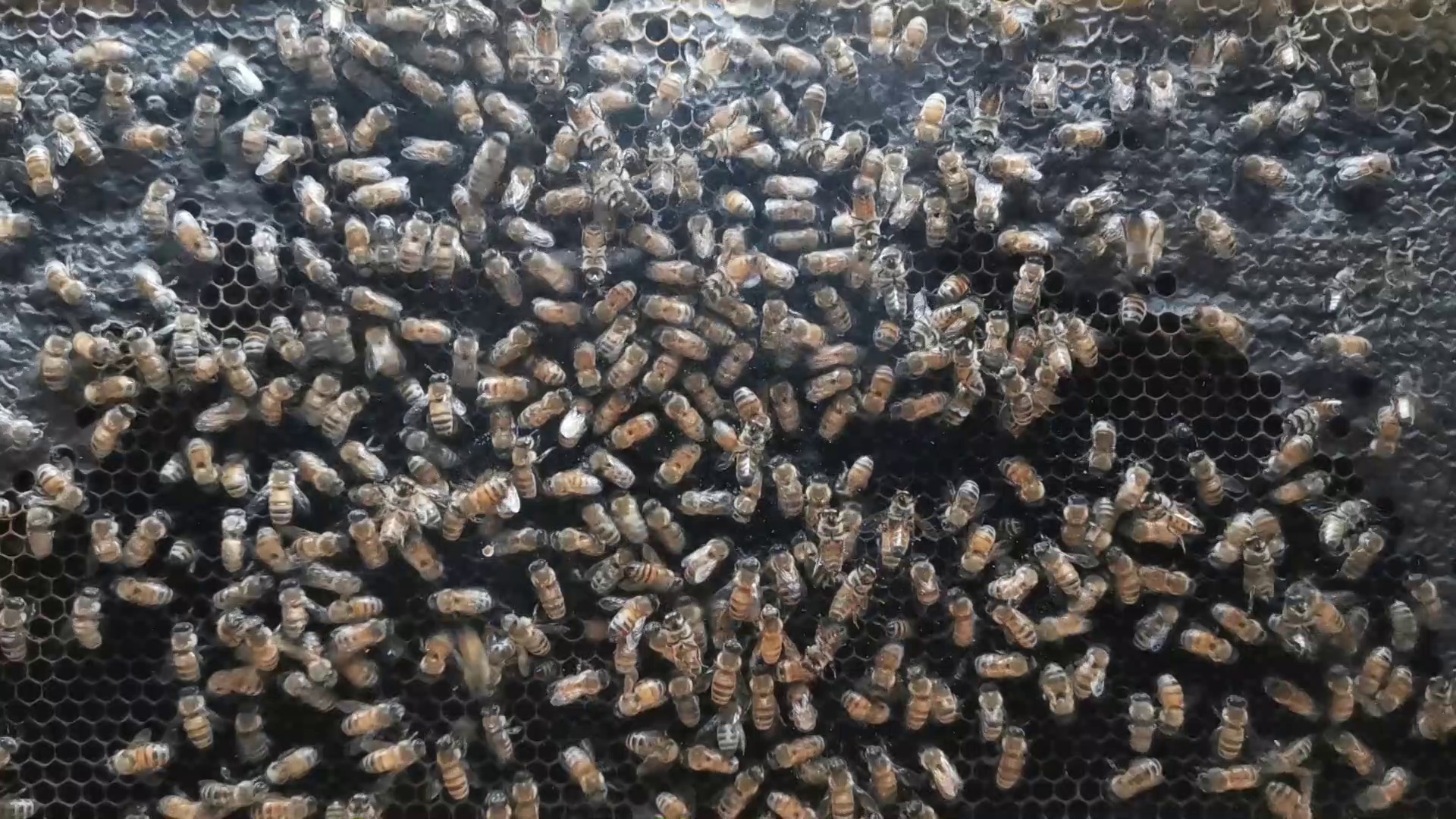

Supplement: Supplementary file 1 — Supplementary Information. [file 41598_2023_44718_MOESM1_ESM.zip › Dataset/test set-system_evaluation/test_set_15fps/017.jpg]

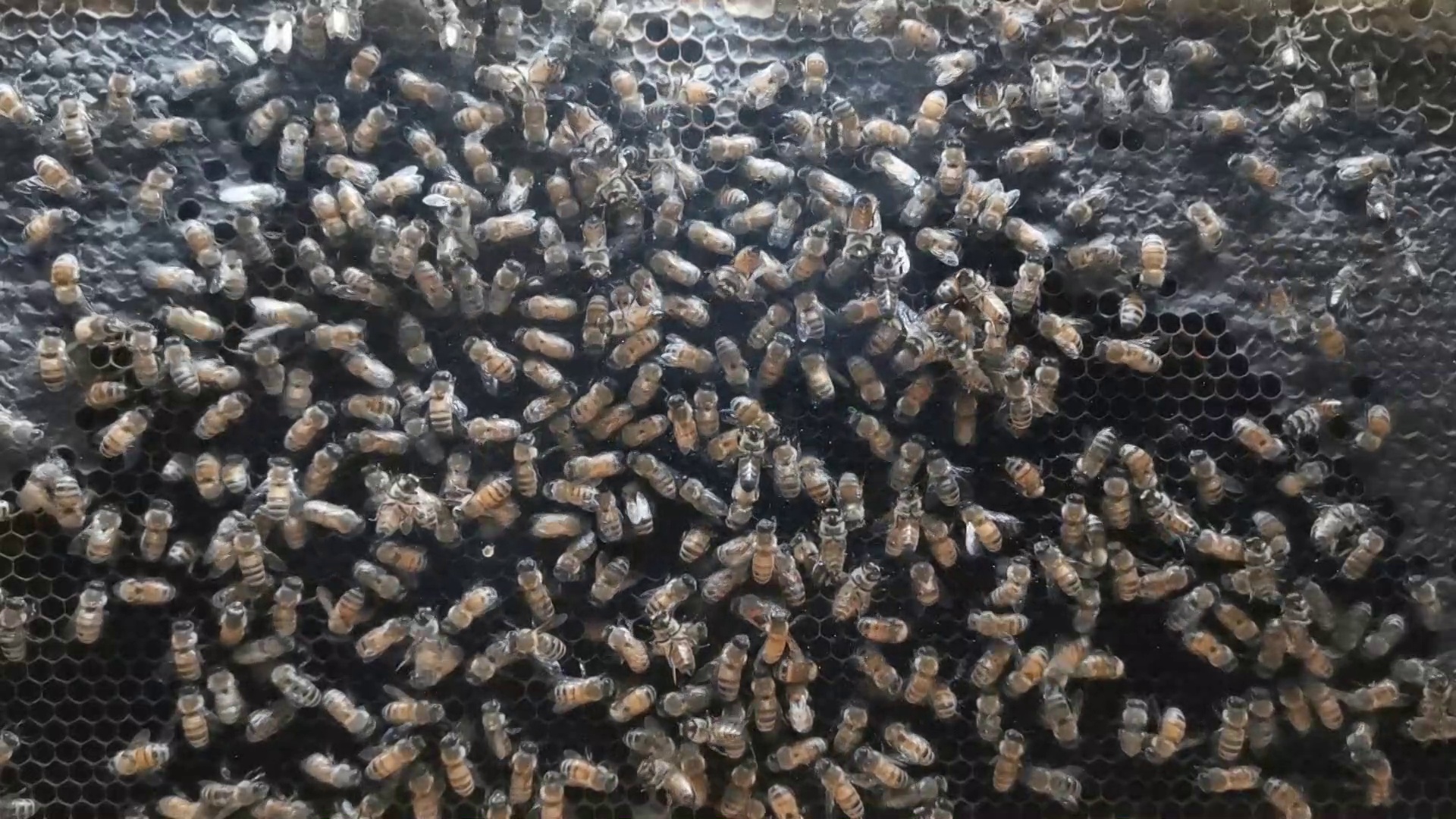

Supplement: Supplementary file 1 — Supplementary Information. [file 41598_2023_44718_MOESM1_ESM.zip › Dataset/test set-system_evaluation/test_set_15fps/057.jpg]

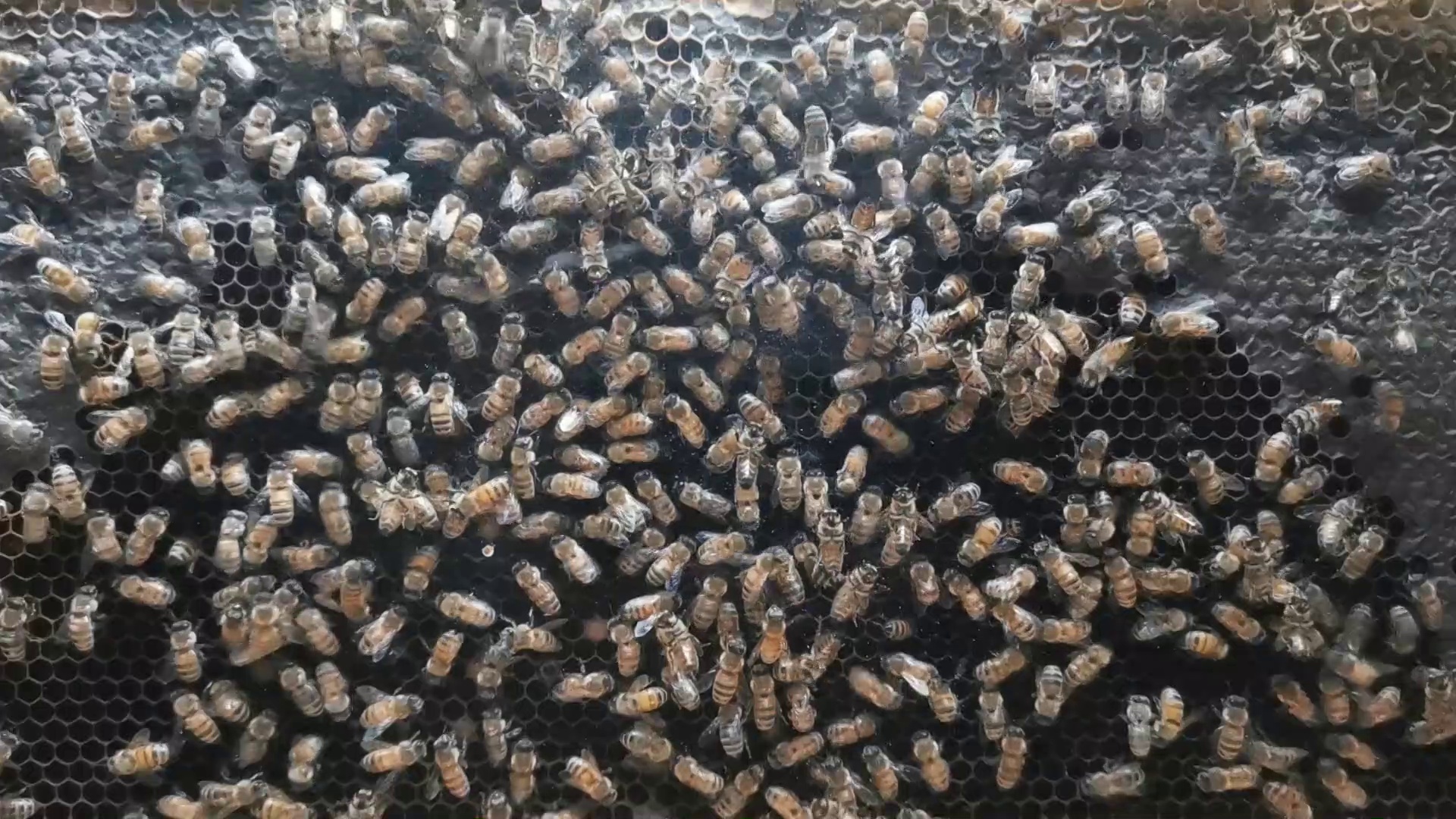

Supplement: Supplementary file 1 — Supplementary Information. [file 41598_2023_44718_MOESM1_ESM.zip › Dataset/test set-system_evaluation/test_set_15fps/031.jpg]

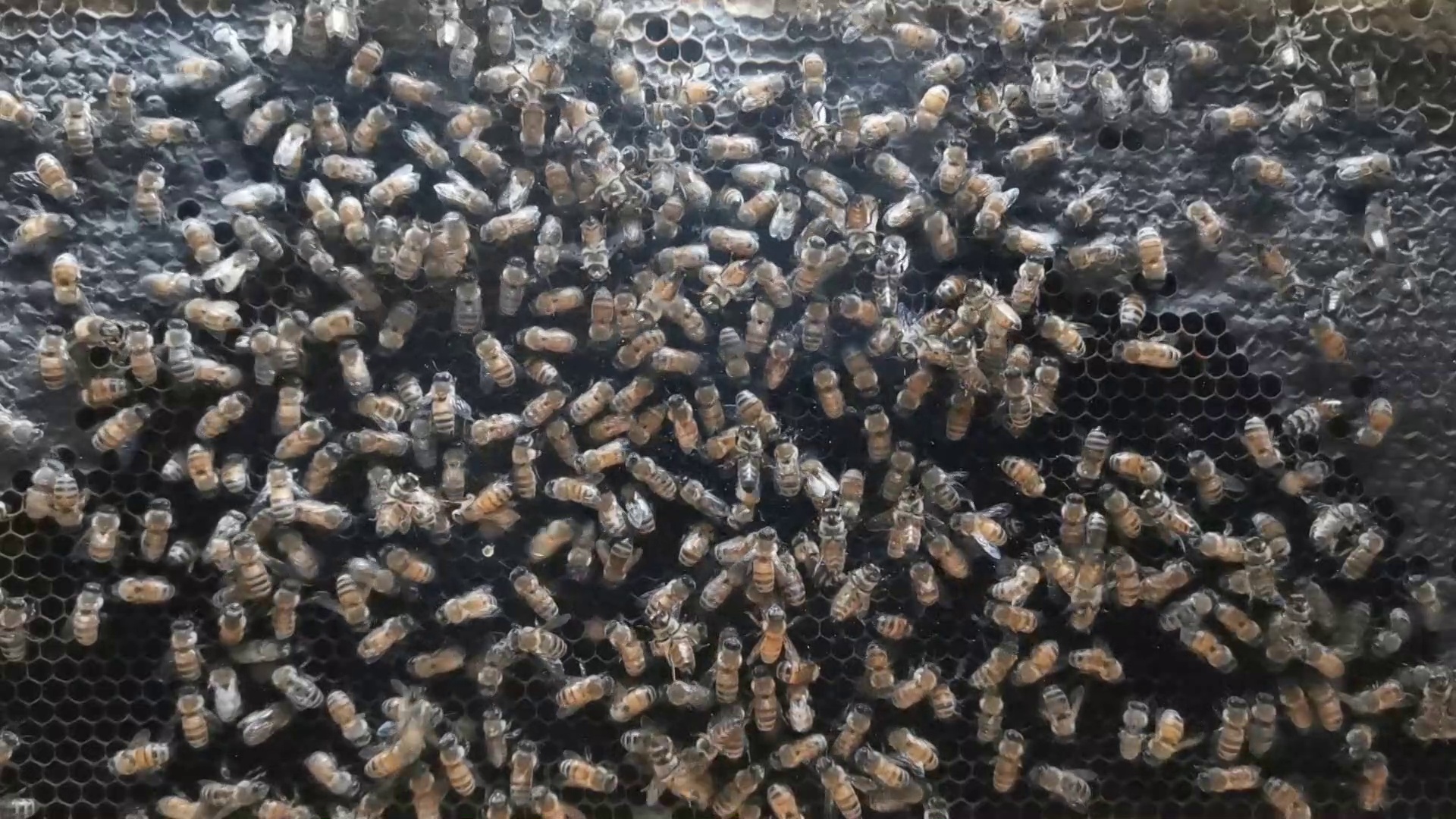

Supplement: Supplementary file 1 — Supplementary Information. [file 41598_2023_44718_MOESM1_ESM.zip › Dataset/test set-system_evaluation/test_set_15fps/052.jpg]

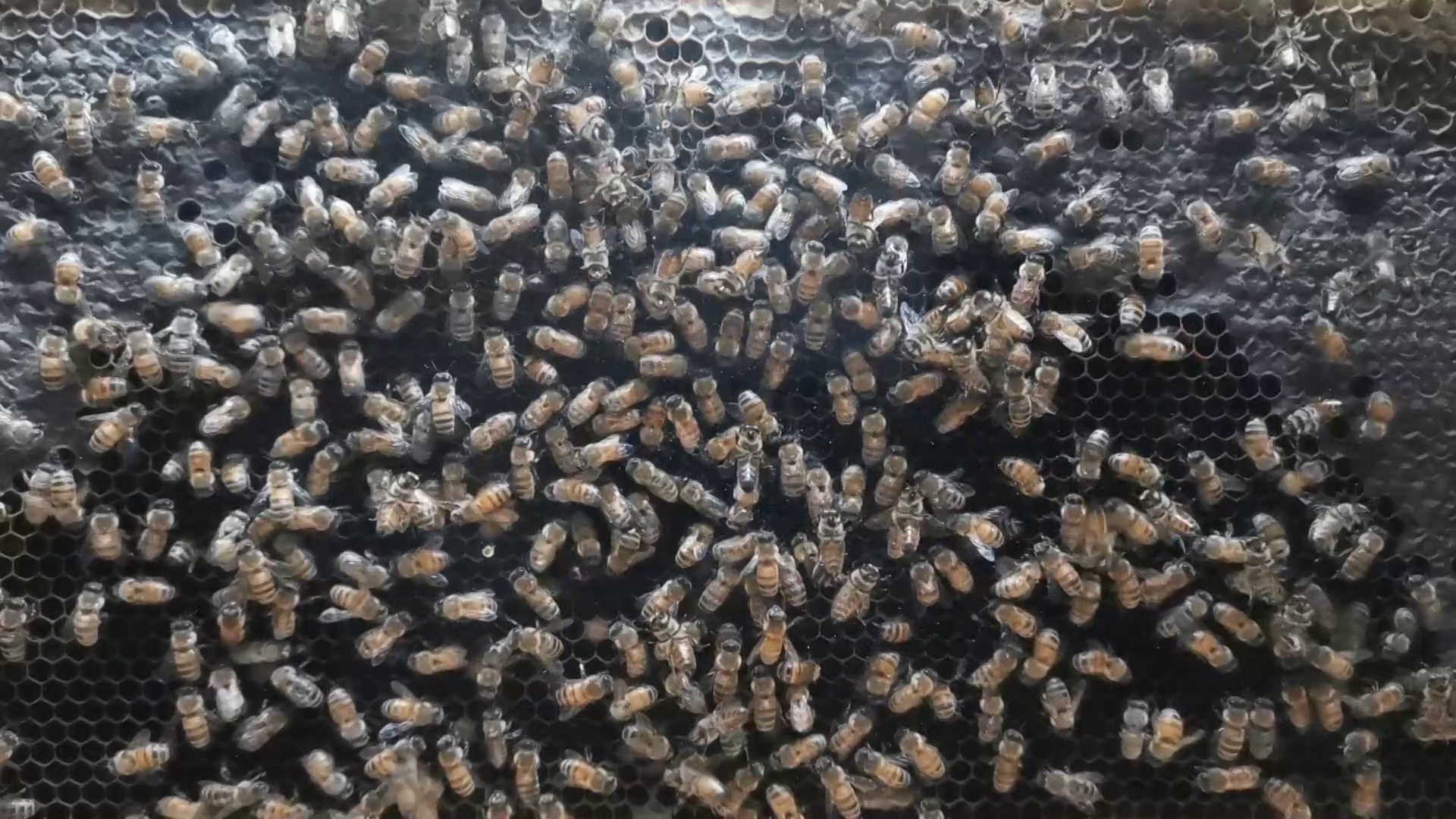

Supplement: Supplementary file 1 — Supplementary Information. [file 41598_2023_44718_MOESM1_ESM.zip › Dataset/test set-system_evaluation/test_set_15fps/048.jpg]

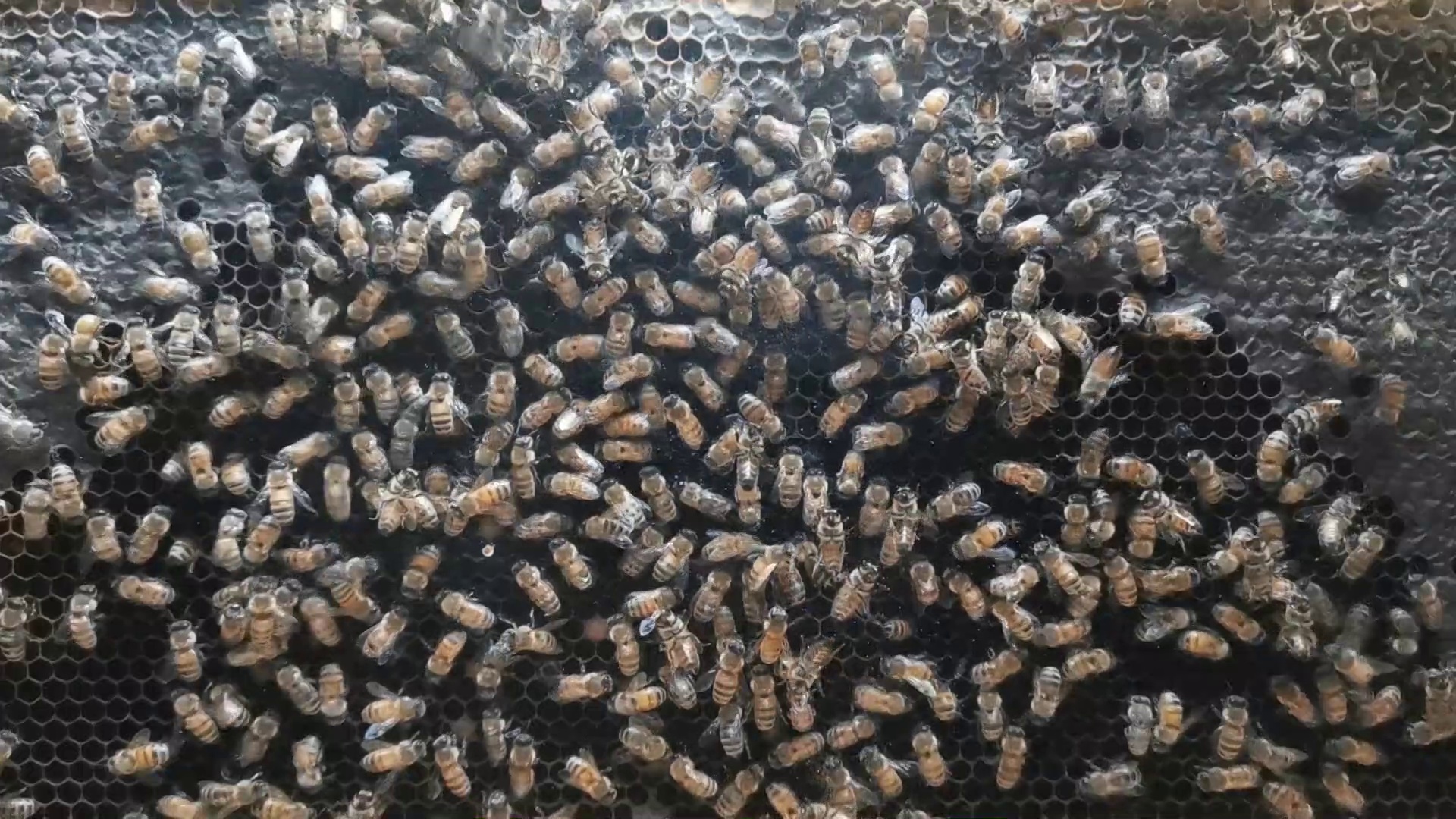

Supplement: Supplementary file 1 — Supplementary Information. [file 41598_2023_44718_MOESM1_ESM.zip › Dataset/test set-system_evaluation/test_set_15fps/034.jpg]

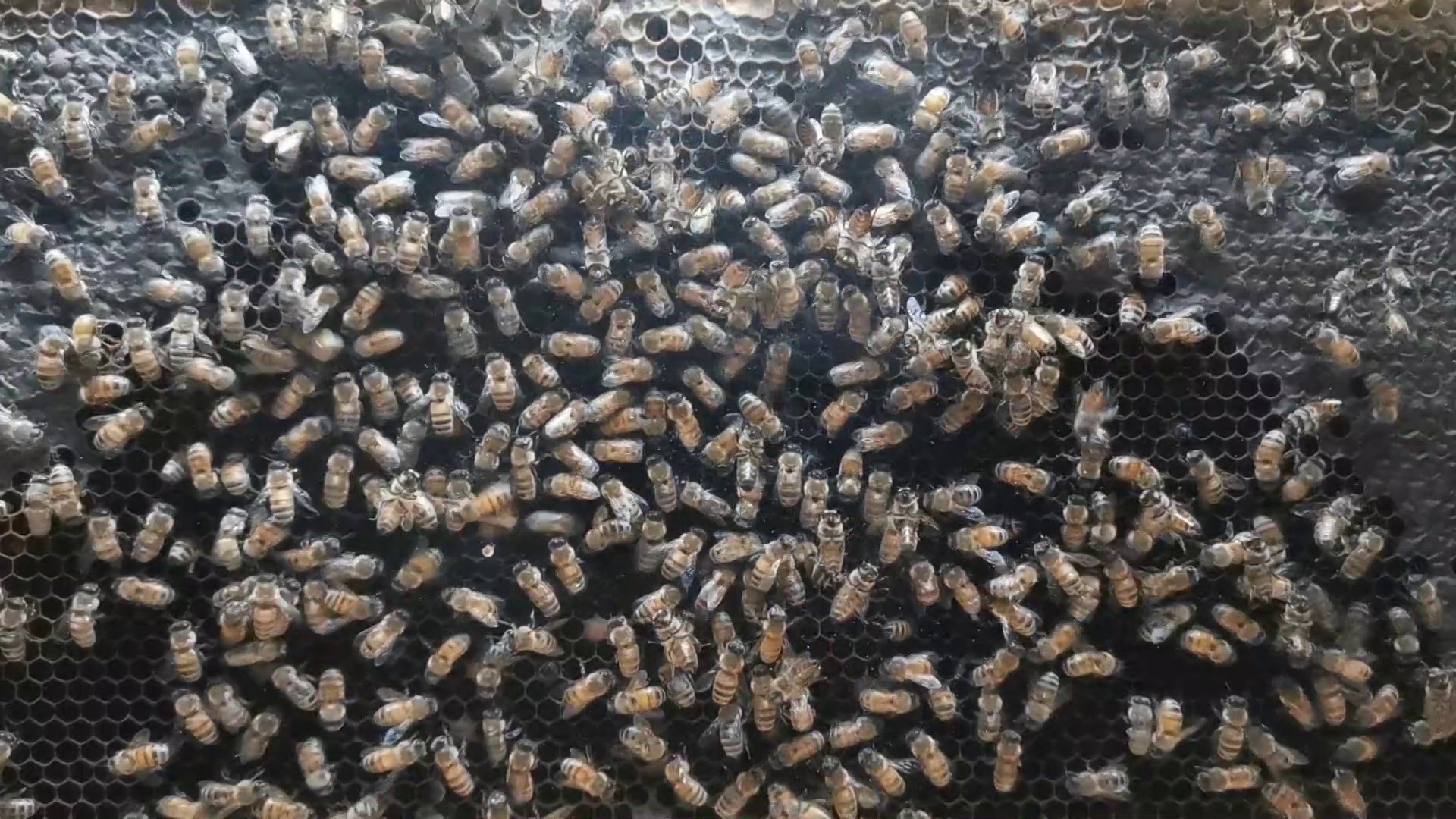

Supplement: Supplementary file 1 — Supplementary Information. [file 41598_2023_44718_MOESM1_ESM.zip › Dataset/test set-system_evaluation/test_set_15fps/037.jpg]

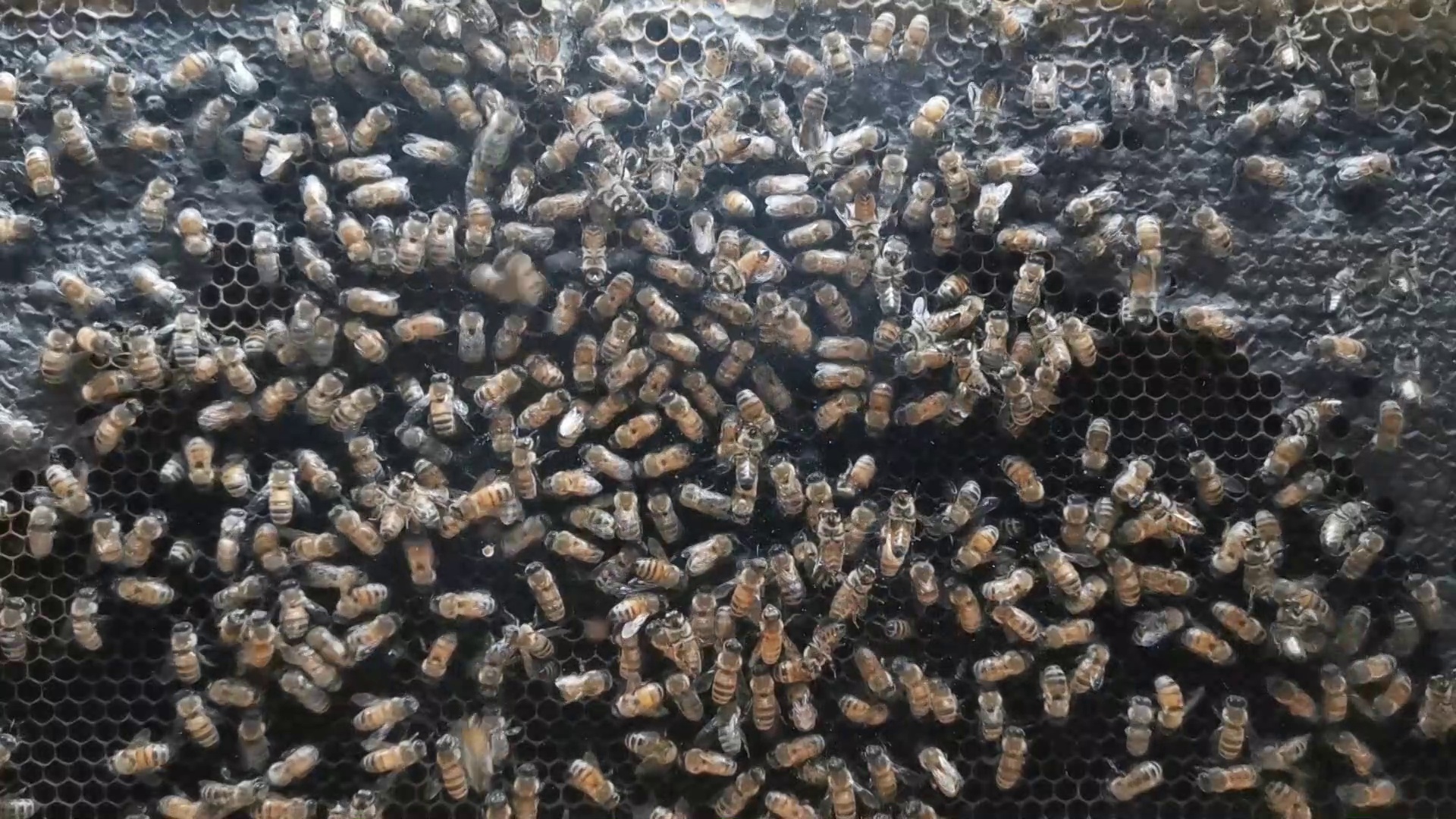

Supplement: Supplementary file 1 — Supplementary Information. [file 41598_2023_44718_MOESM1_ESM.zip › Dataset/test set-system_evaluation/test_set_15fps/022.jpg]

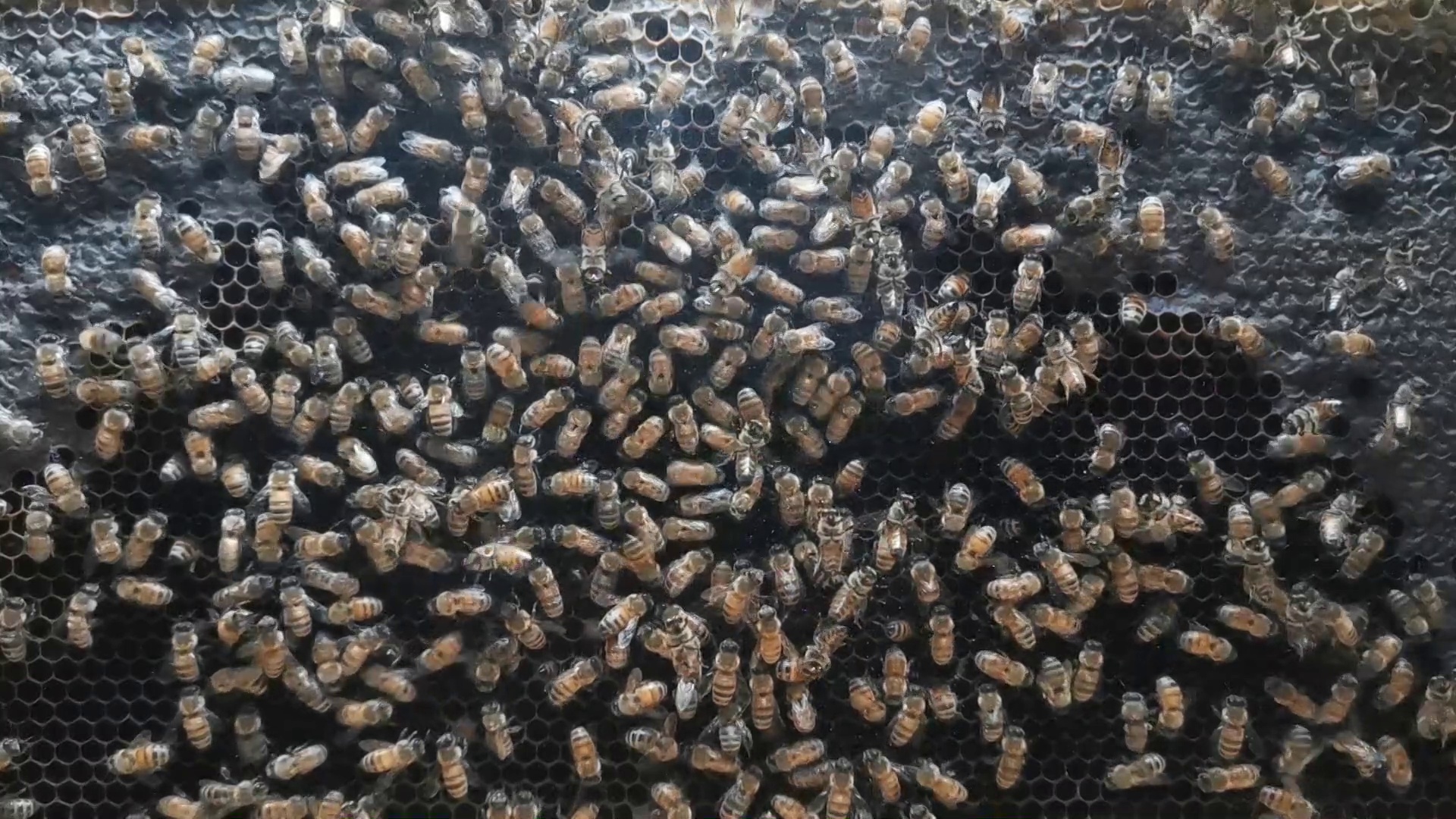

Supplement: Supplementary file 1 — Supplementary Information. [file 41598_2023_44718_MOESM1_ESM.zip › Dataset/test set-system_evaluation/test_set_15fps/008.jpg]

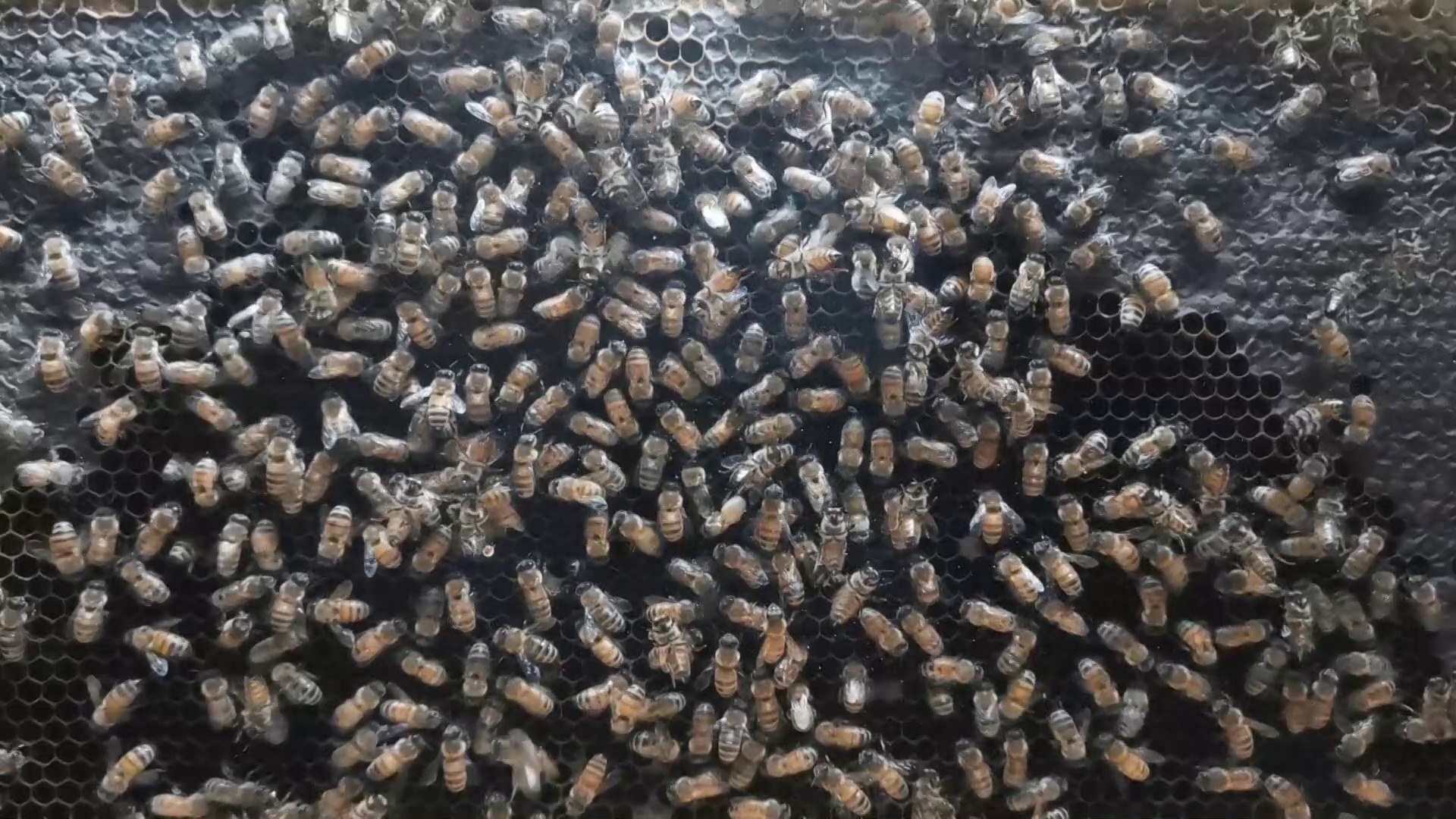

Supplement: Supplementary file 1 — Supplementary Information. [file 41598_2023_44718_MOESM1_ESM.zip › Dataset/test set-system_evaluation/test_set_5fps/038.jpg]

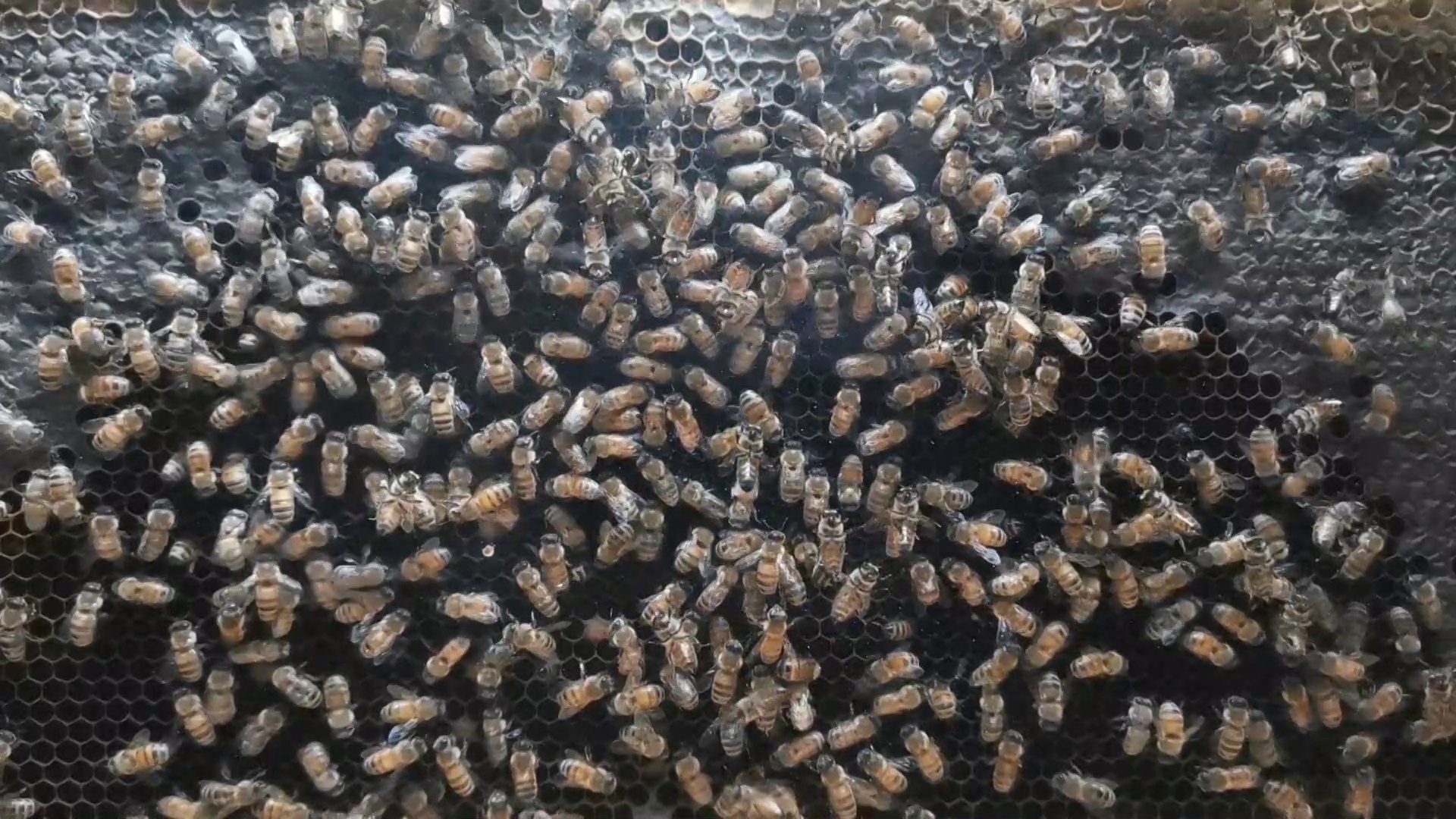

Supplement: Supplementary file 1 — Supplementary Information. [file 41598_2023_44718_MOESM1_ESM.zip › Dataset/test set-system_evaluation/test_set_15fps/042.jpg]

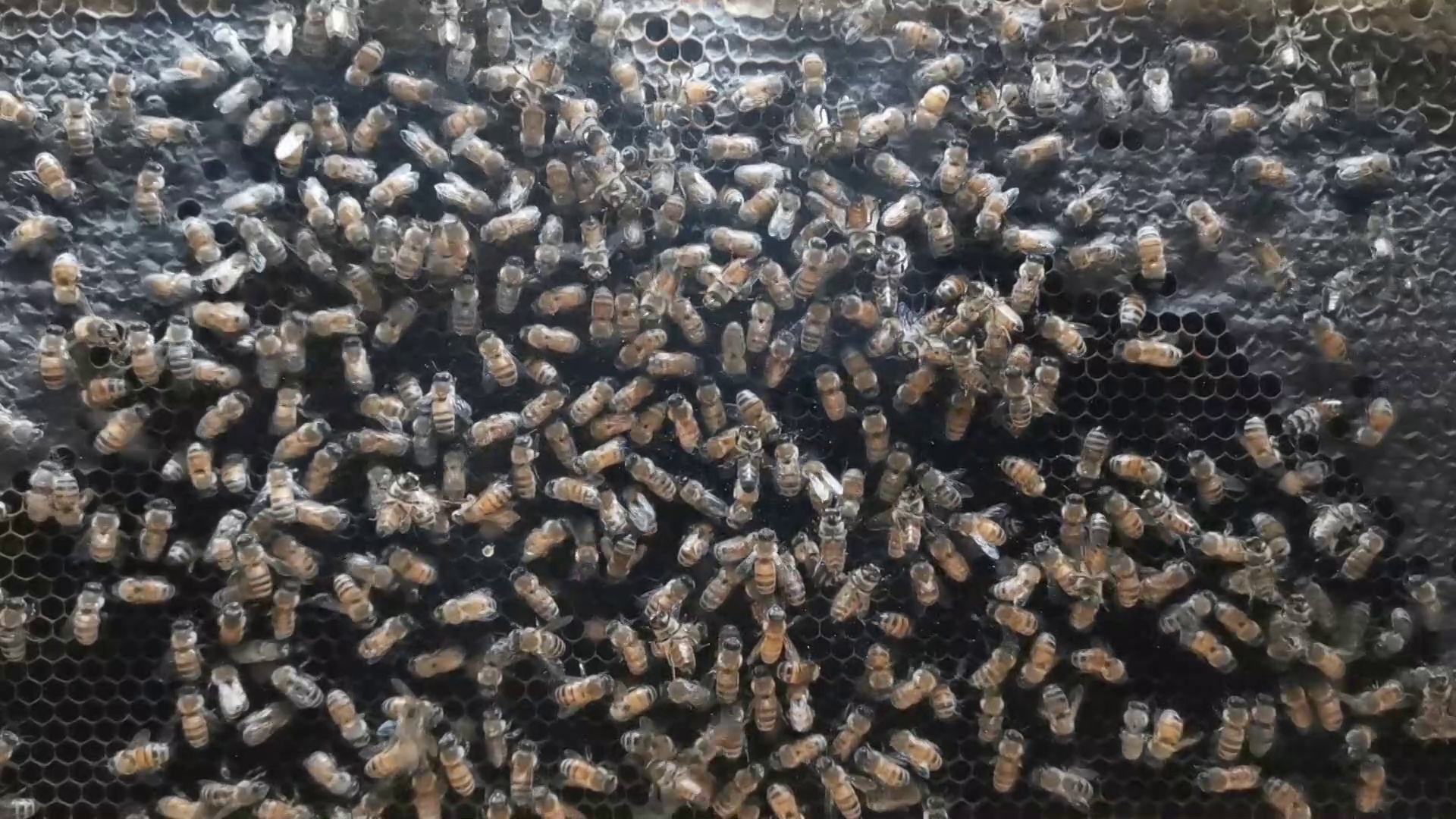

Supplement: Supplementary file 1 — Supplementary Information. [file 41598_2023_44718_MOESM1_ESM.zip › Dataset/test set-system_evaluation/test_set_15fps/051.jpg]

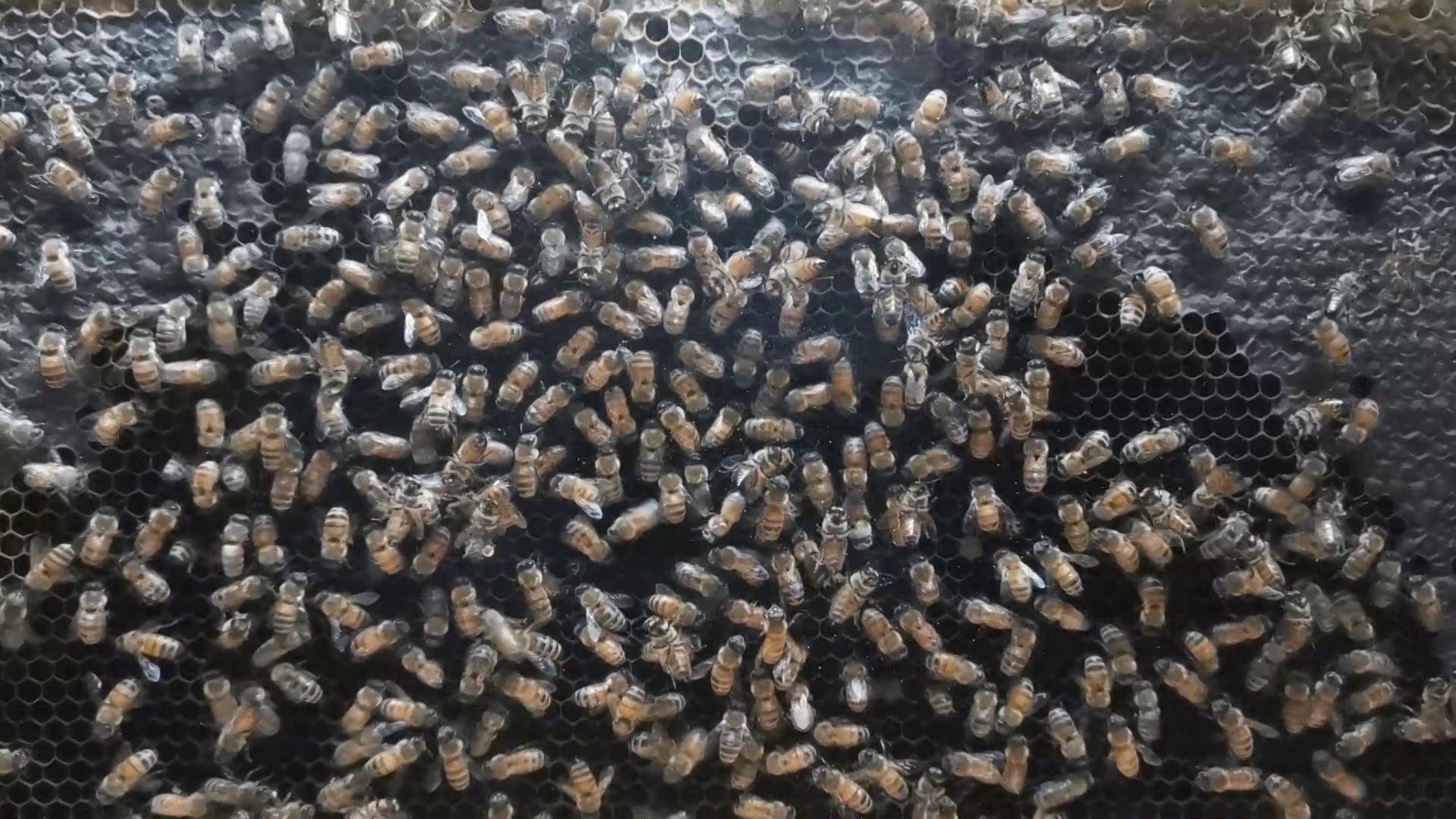

Supplement: Supplementary file 1 — Supplementary Information. [file 41598_2023_44718_MOESM1_ESM.zip › Dataset/test set-system_evaluation/test_set_5fps/040.jpg]

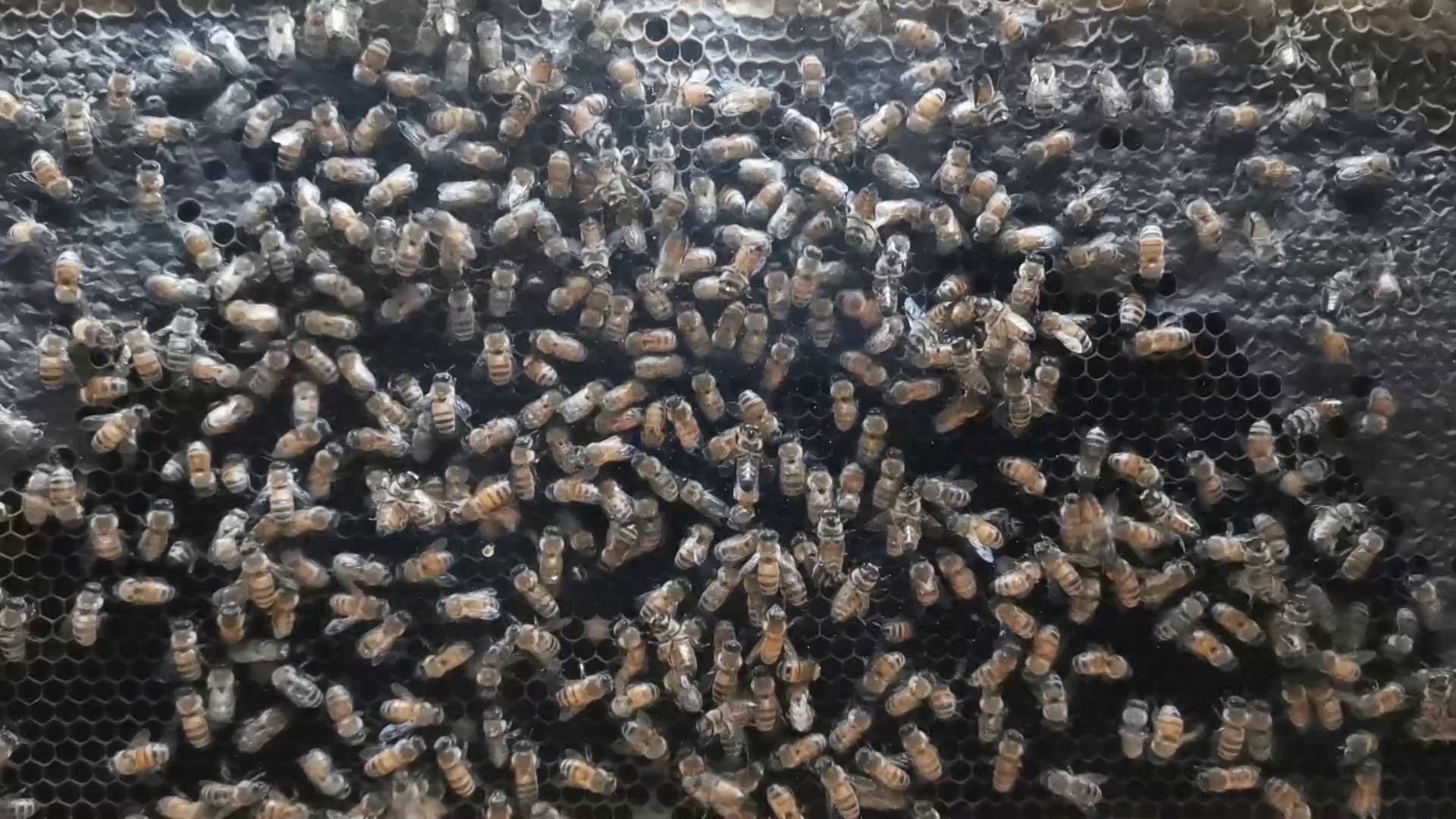

Supplement: Supplementary file 1 — Supplementary Information. [file 41598_2023_44718_MOESM1_ESM.zip › Dataset/test set-system_evaluation/test_set_15fps/046.jpg]
