# Supplementary figures and images for: Individual honey bee tracking in a beehive environment using deep learning and Kalman filter (part 1 of 2)
Source: Sci Rep. 2024 Jan 11;14:1061. doi: 10.1038/s41598-023-44718-y (PMC10784501; doi:10.1038/s41598-023-44718-y)

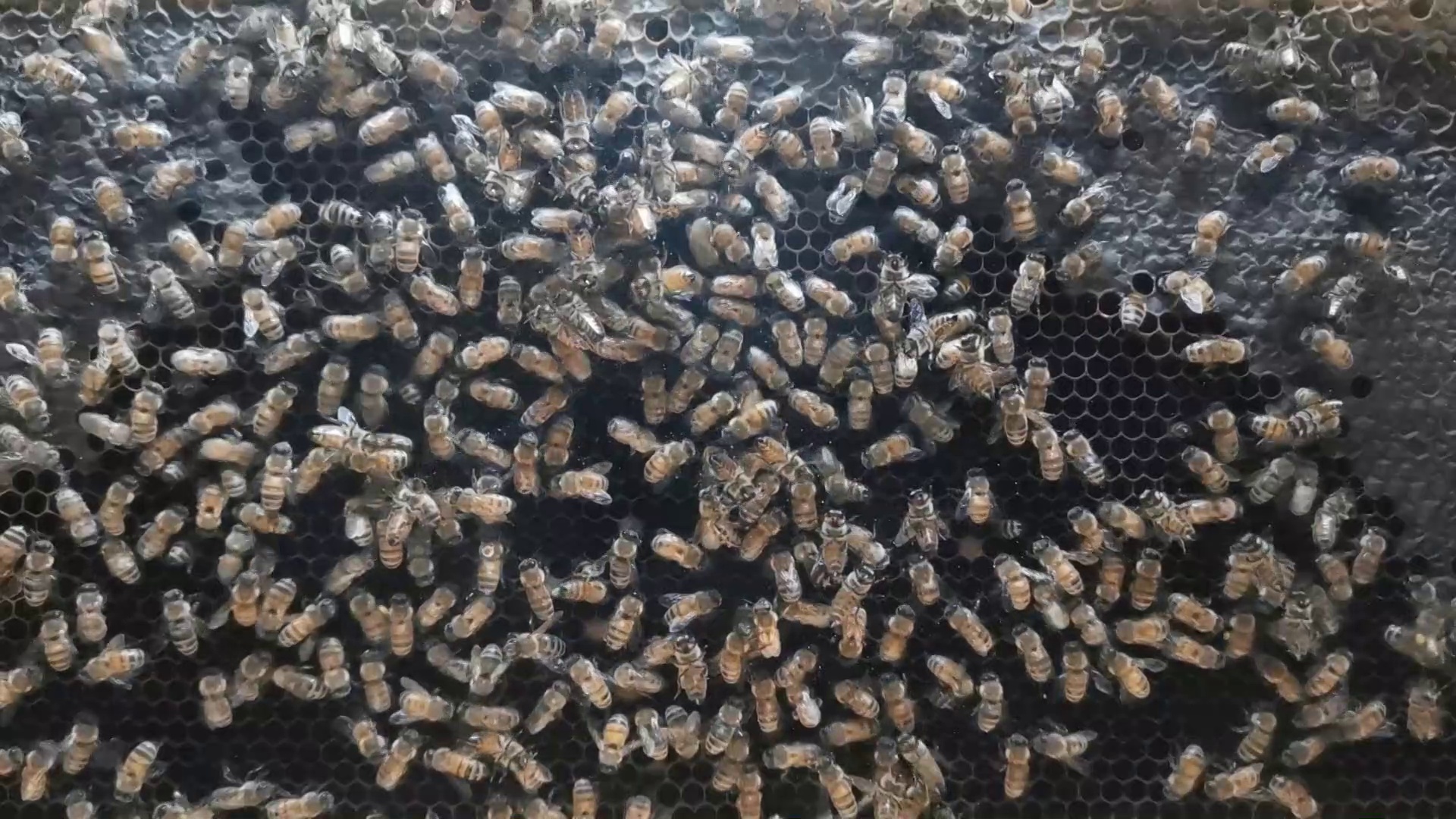

Supplement: Supplementary file 1 — Supplementary Information. [file 41598_2023_44718_MOESM1_ESM.zip › Dataset/dataset-Mask_RCNN_Training/dataset/val/002.jpg]

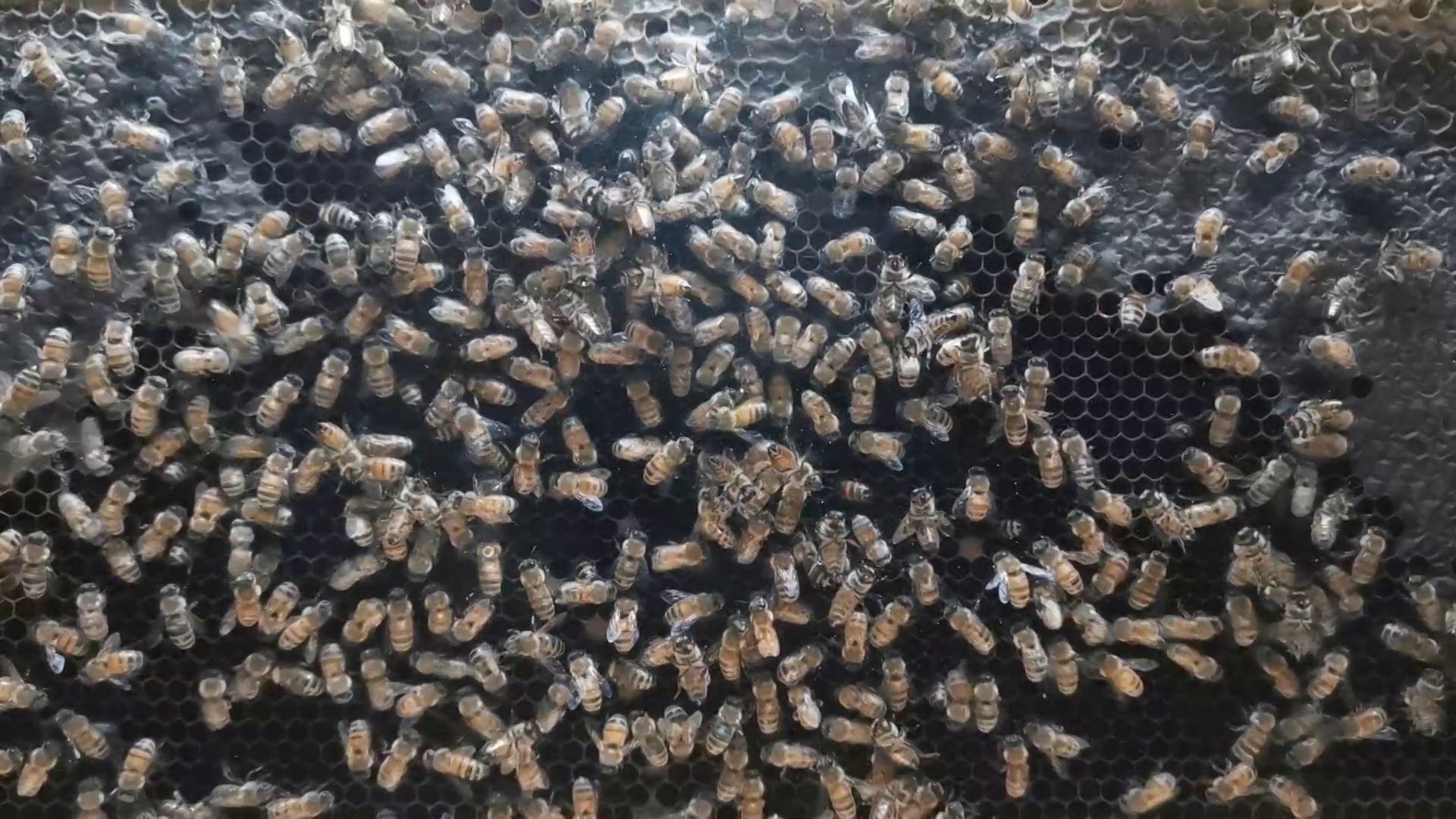

Supplement: Supplementary file 1 — Supplementary Information. [file 41598_2023_44718_MOESM1_ESM.zip › Dataset/dataset-Mask_RCNN_Training/dataset/val/005.jpg]

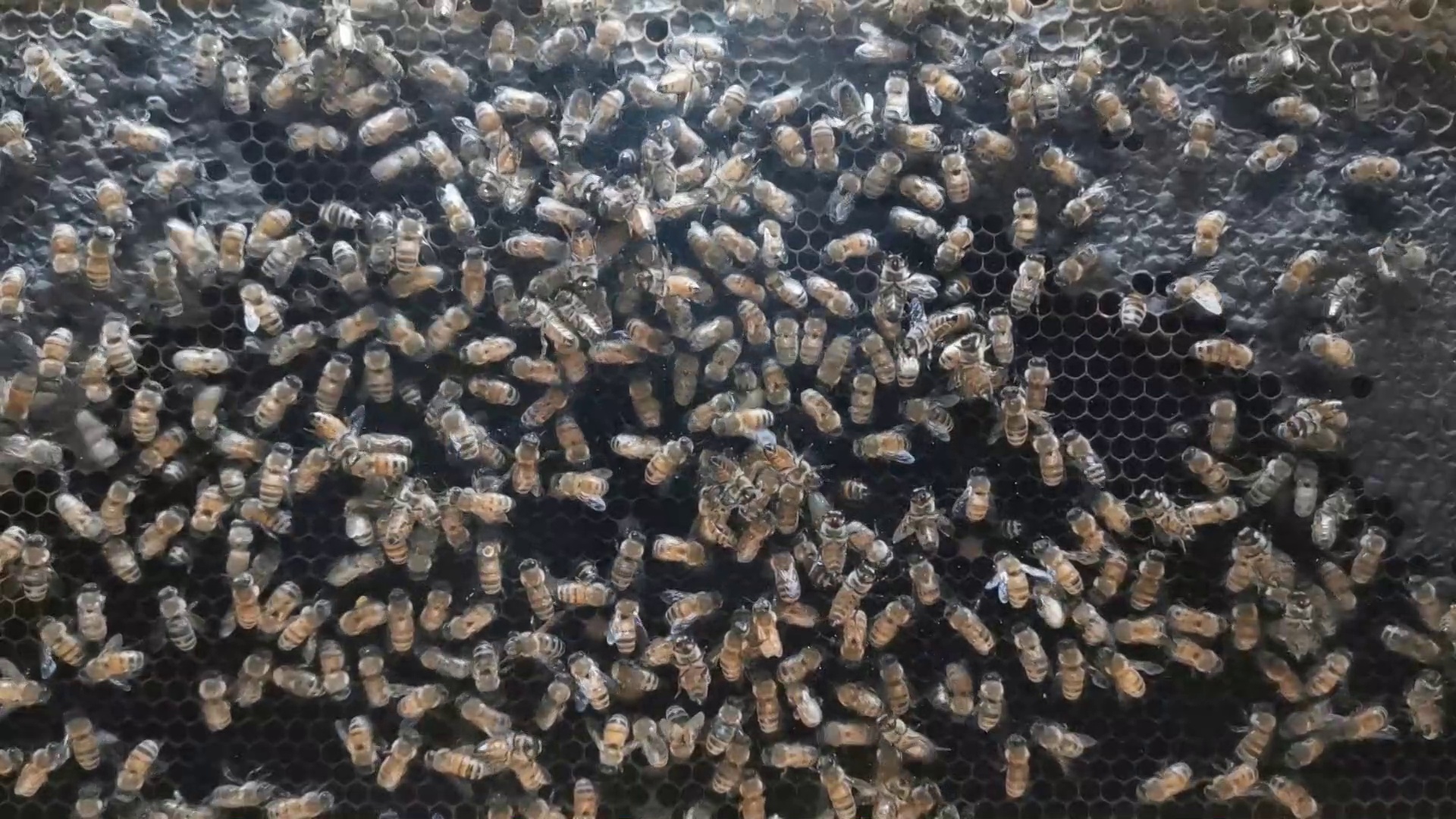

Supplement: Supplementary file 1 — Supplementary Information. [file 41598_2023_44718_MOESM1_ESM.zip › Dataset/dataset-Mask_RCNN_Training/dataset/val/004.jpg]

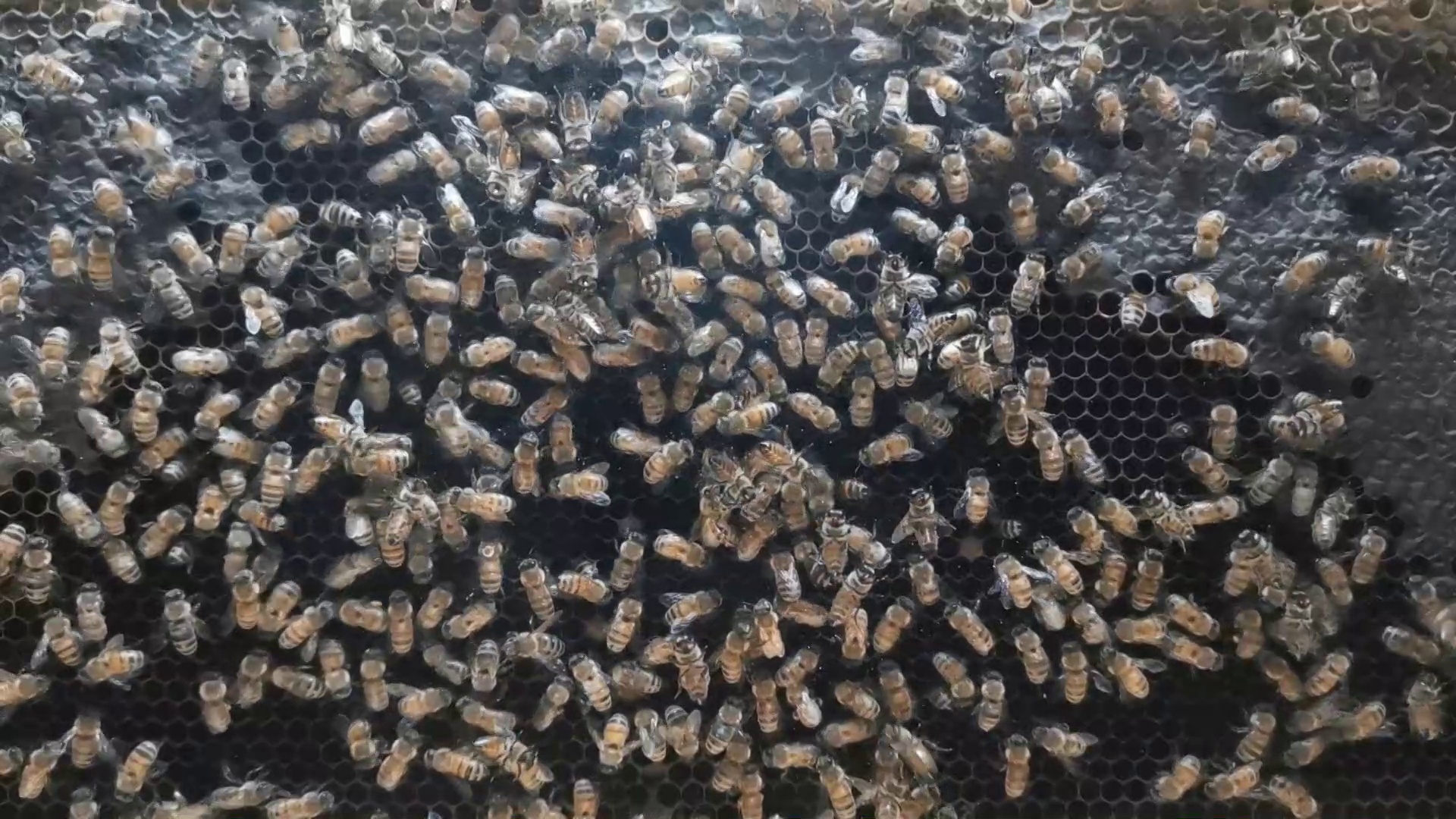

Supplement: Supplementary file 1 — Supplementary Information. [file 41598_2023_44718_MOESM1_ESM.zip › Dataset/dataset-Mask_RCNN_Training/dataset/val/003.jpg]

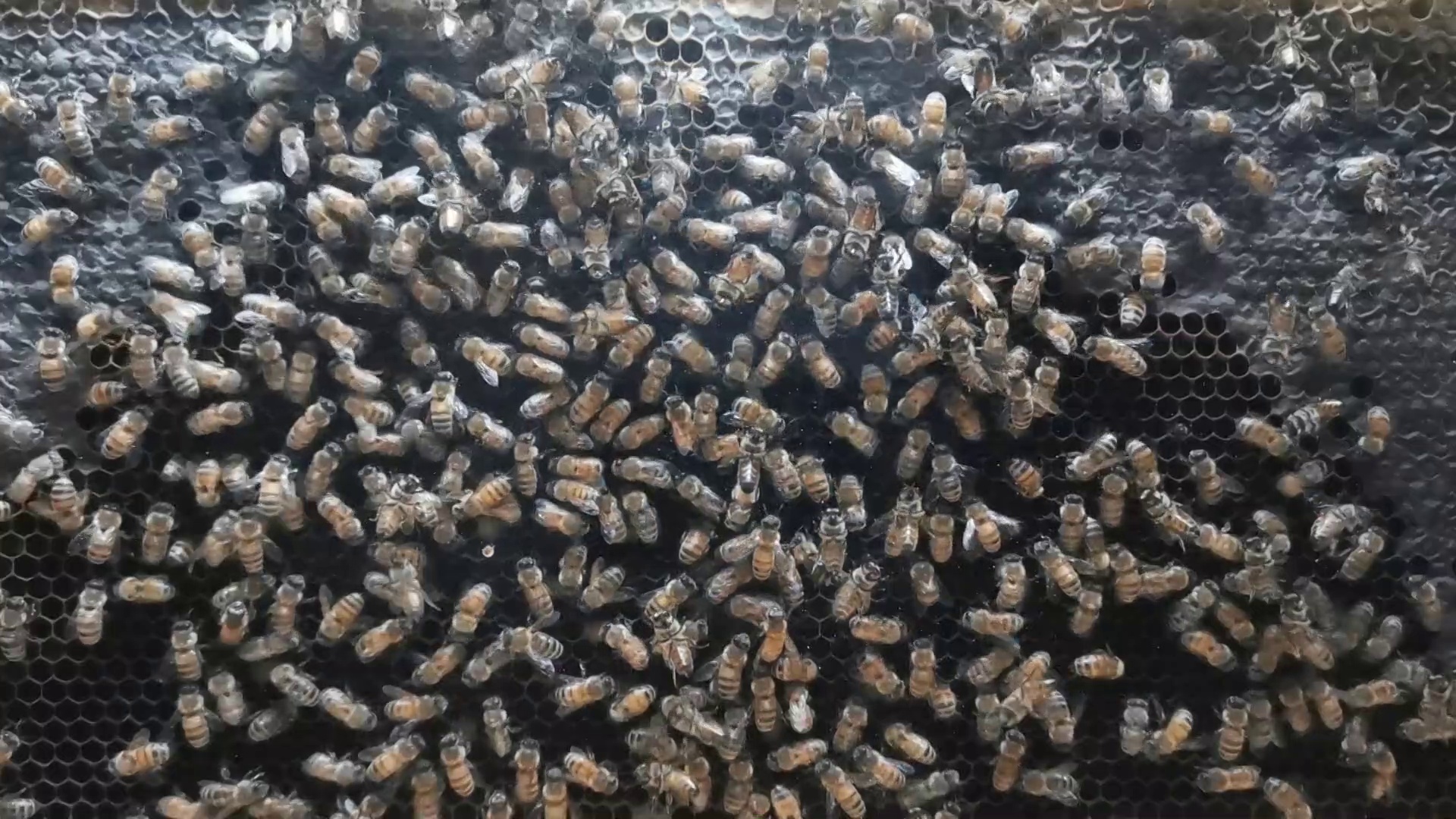

Supplement: Supplementary file 1 — Supplementary Information. [file 41598_2023_44718_MOESM1_ESM.zip › Dataset/dataset-Mask_RCNN_Training/dataset/train/022.jpg]

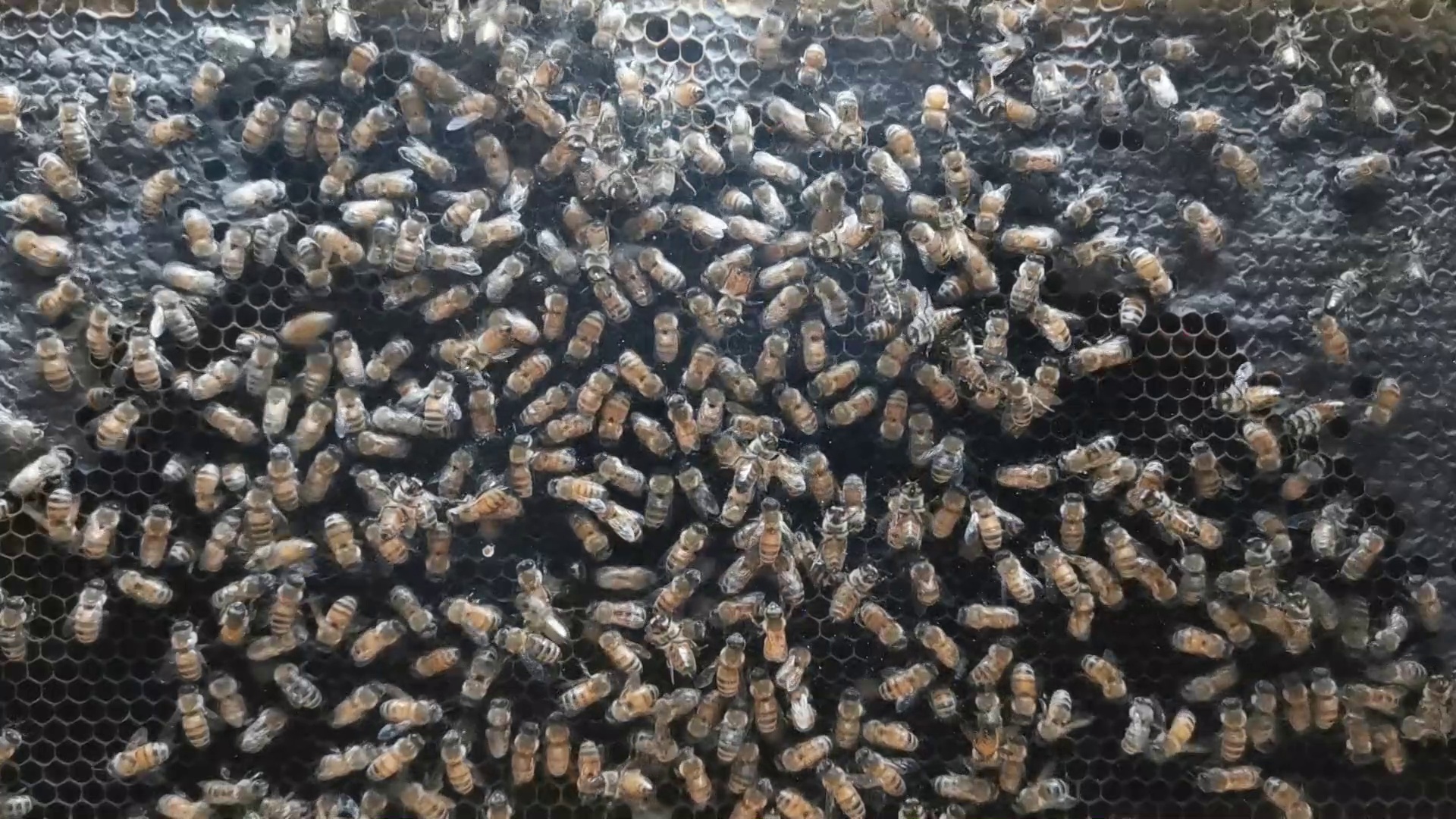

Supplement: Supplementary file 1 — Supplementary Information. [file 41598_2023_44718_MOESM1_ESM.zip › Dataset/dataset-Mask_RCNN_Training/dataset/train/028.jpg]

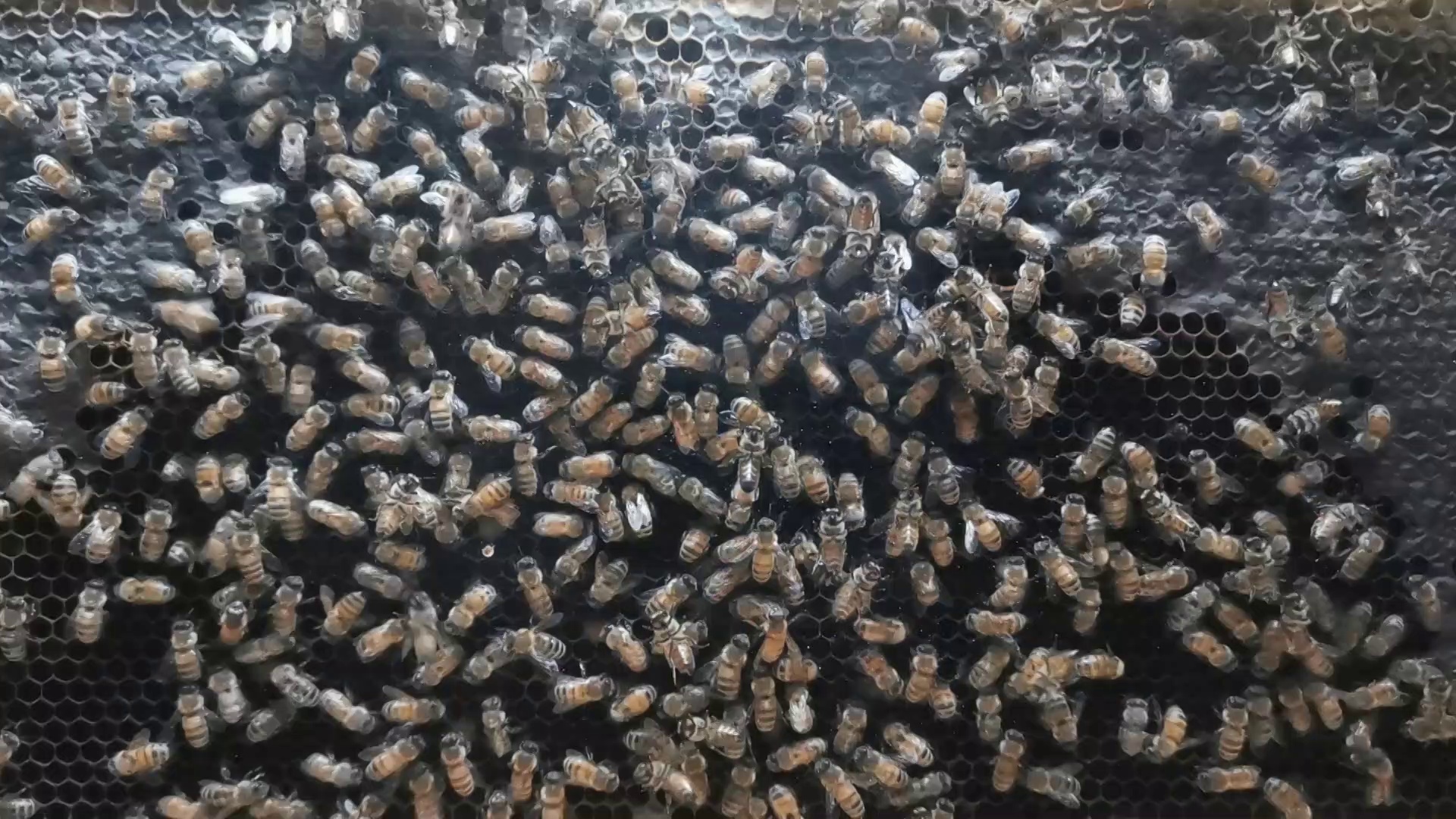

Supplement: Supplementary file 1 — Supplementary Information. [file 41598_2023_44718_MOESM1_ESM.zip › Dataset/dataset-Mask_RCNN_Training/dataset/train/021.jpg]

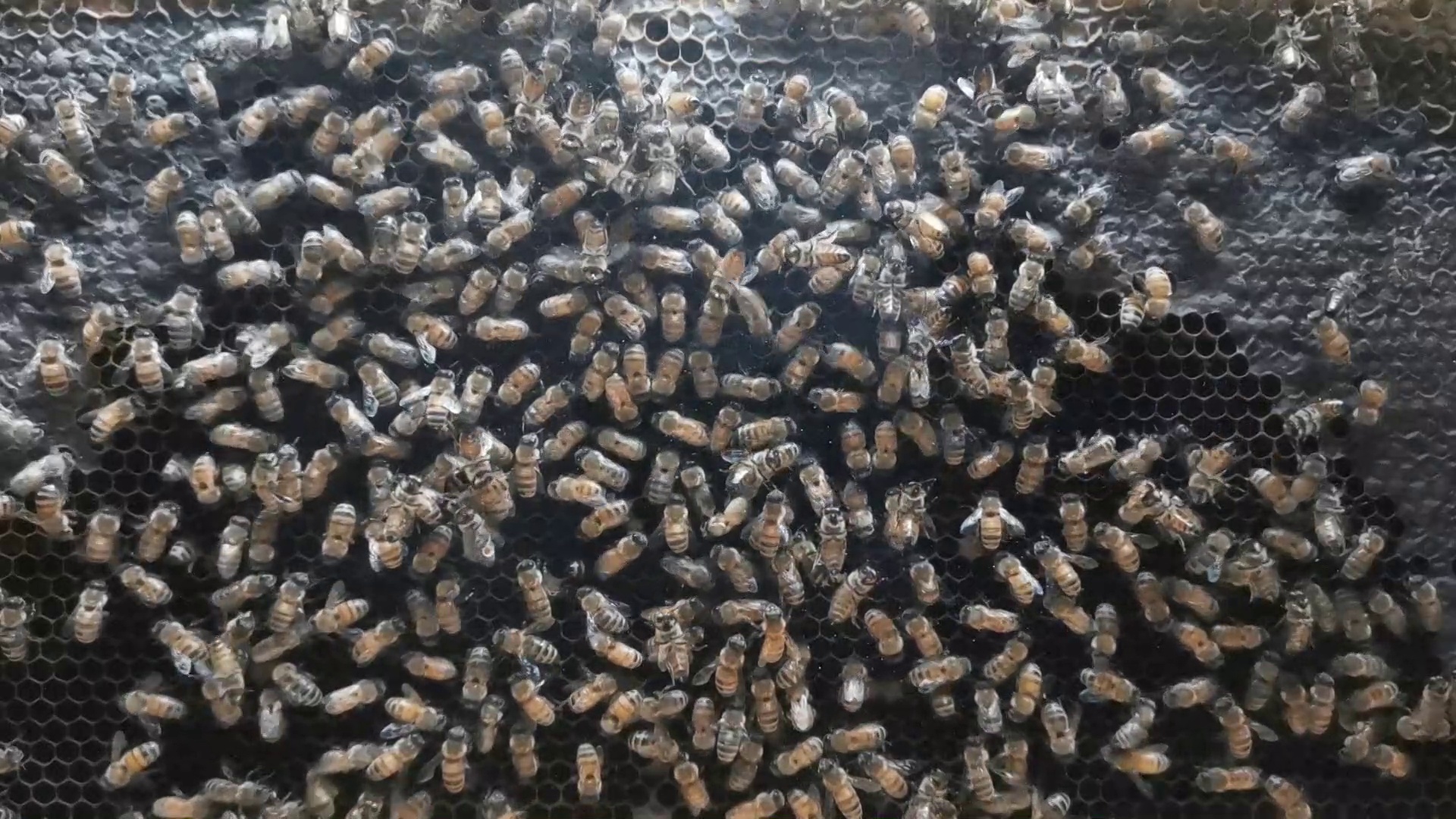

Supplement: Supplementary file 1 — Supplementary Information. [file 41598_2023_44718_MOESM1_ESM.zip › Dataset/dataset-Mask_RCNN_Training/dataset/train/035.jpg]

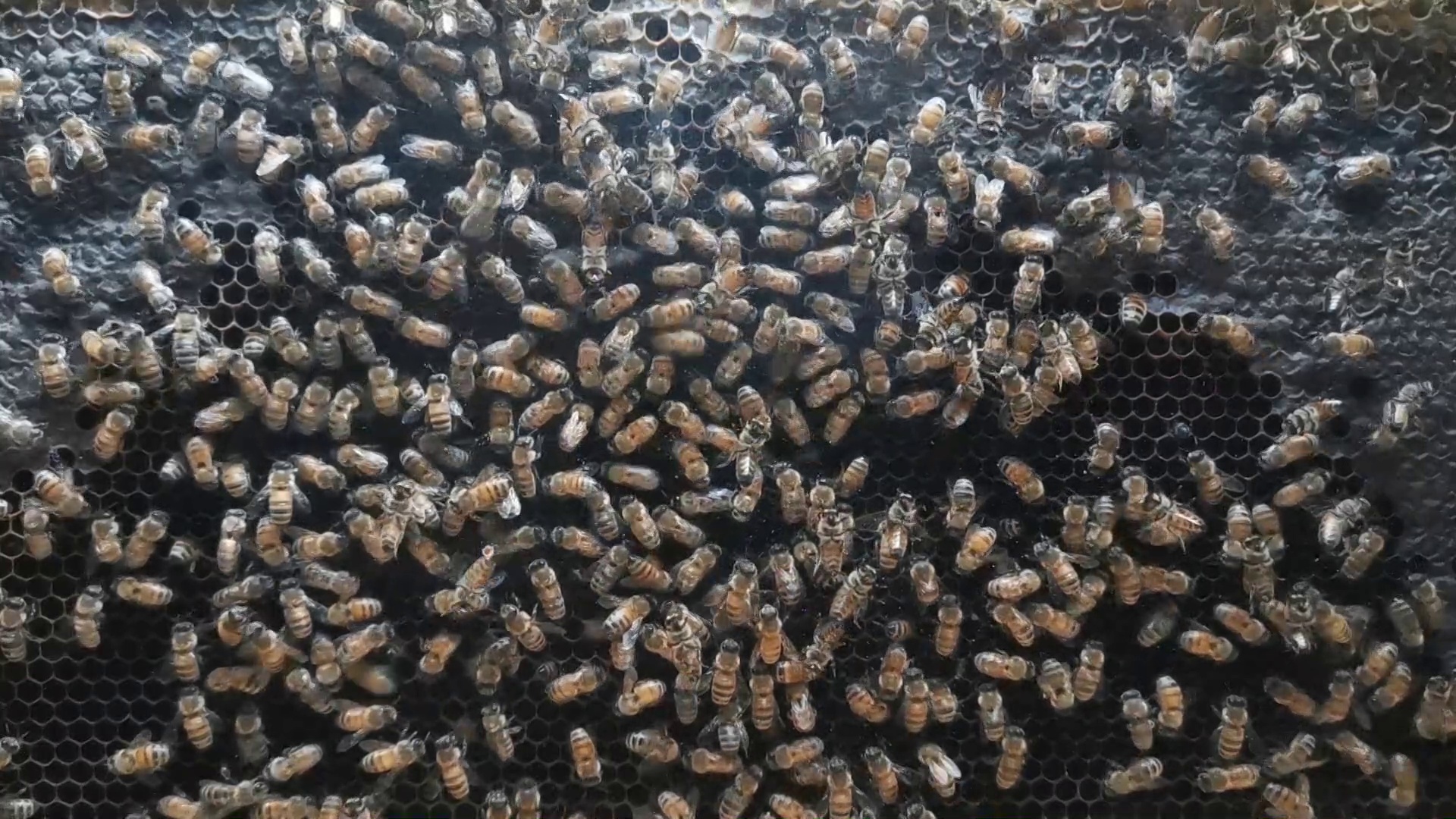

Supplement: Supplementary file 1 — Supplementary Information. [file 41598_2023_44718_MOESM1_ESM.zip › Dataset/dataset-Mask_RCNN_Training/dataset/train/005.jpg]

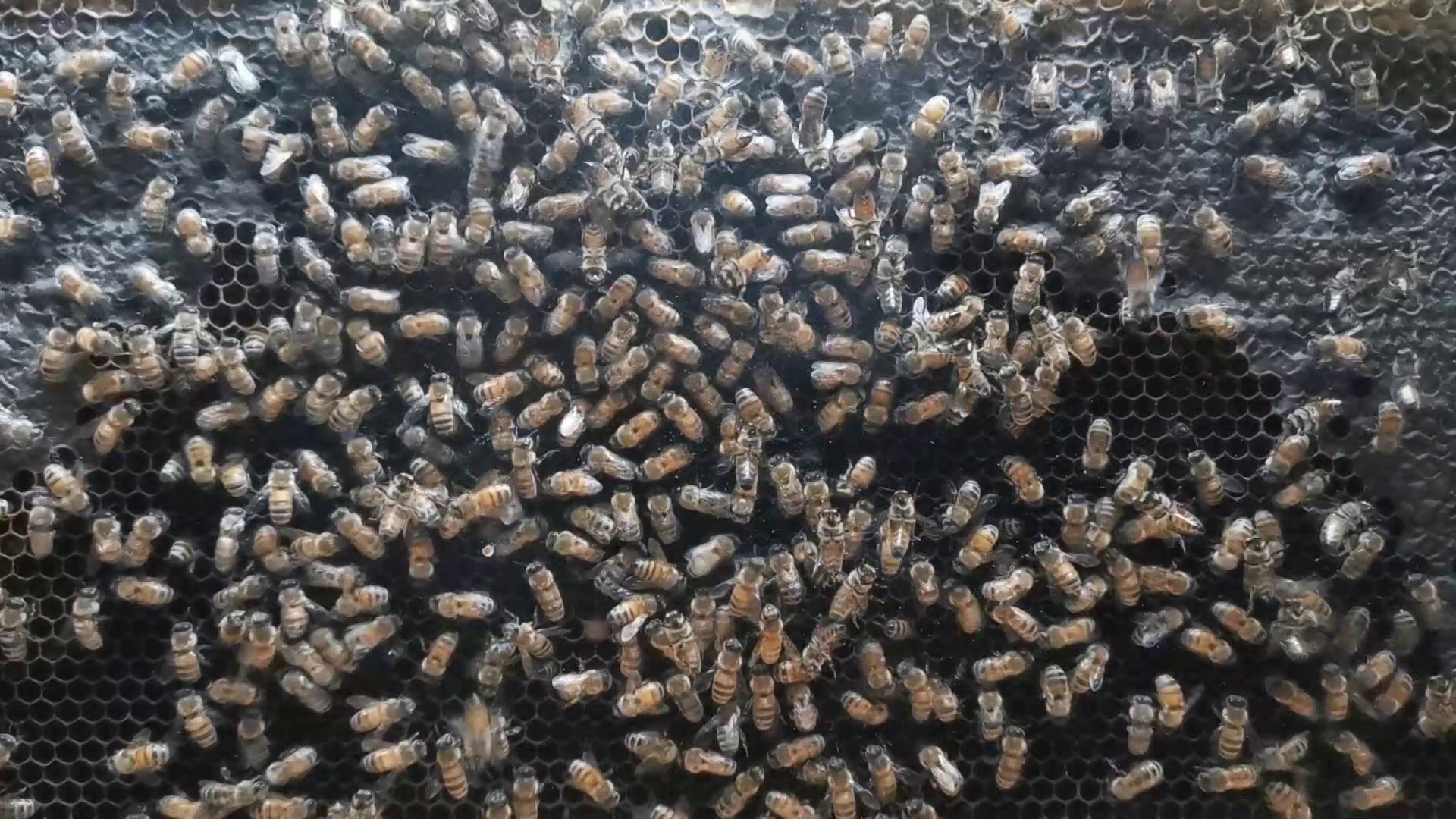

Supplement: Supplementary file 1 — Supplementary Information. [file 41598_2023_44718_MOESM1_ESM.zip › Dataset/dataset-Mask_RCNN_Training/dataset/train/008.jpg]

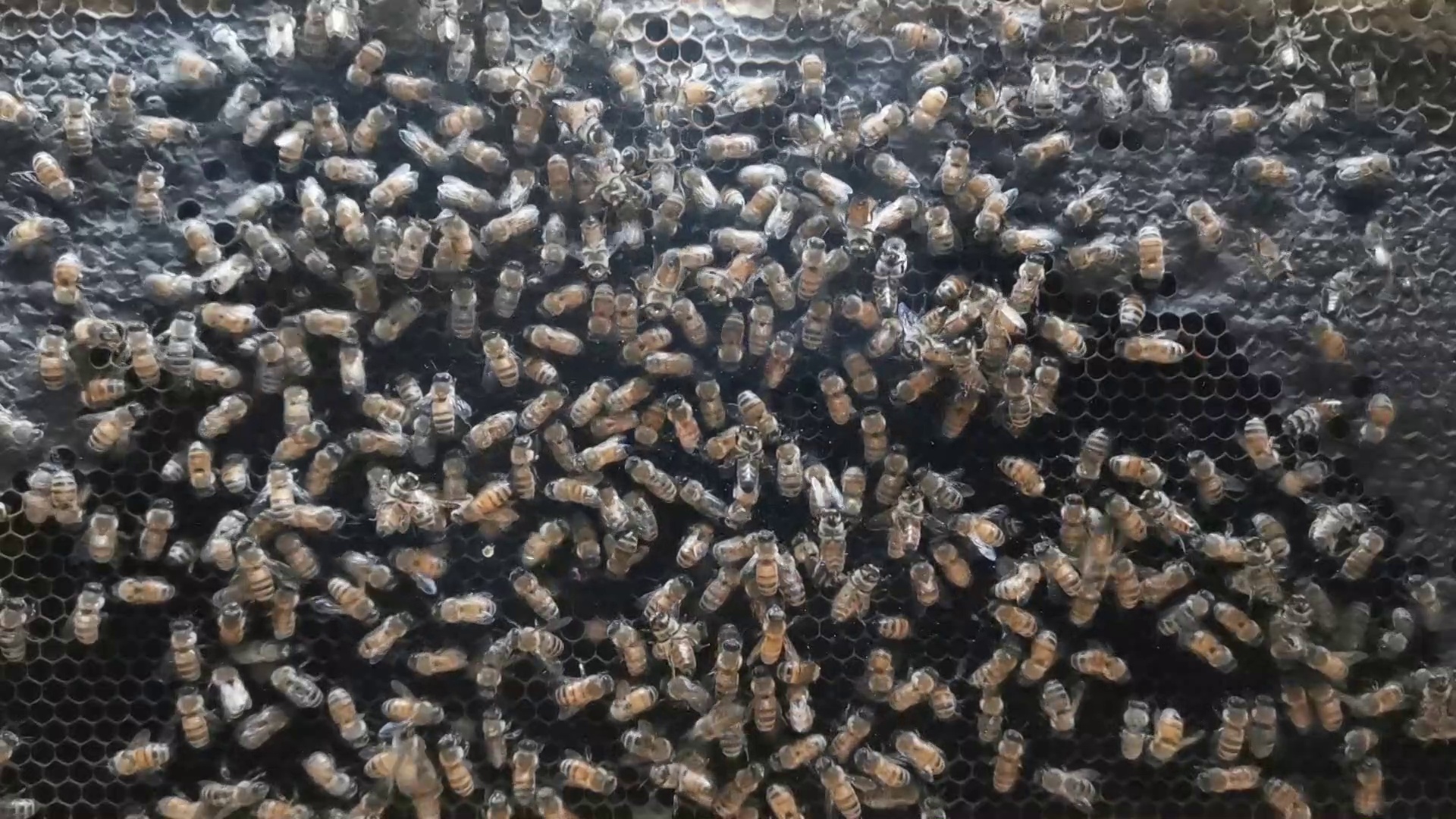

Supplement: Supplementary file 1 — Supplementary Information. [file 41598_2023_44718_MOESM1_ESM.zip › Dataset/dataset-Mask_RCNN_Training/dataset/train/018.jpg]

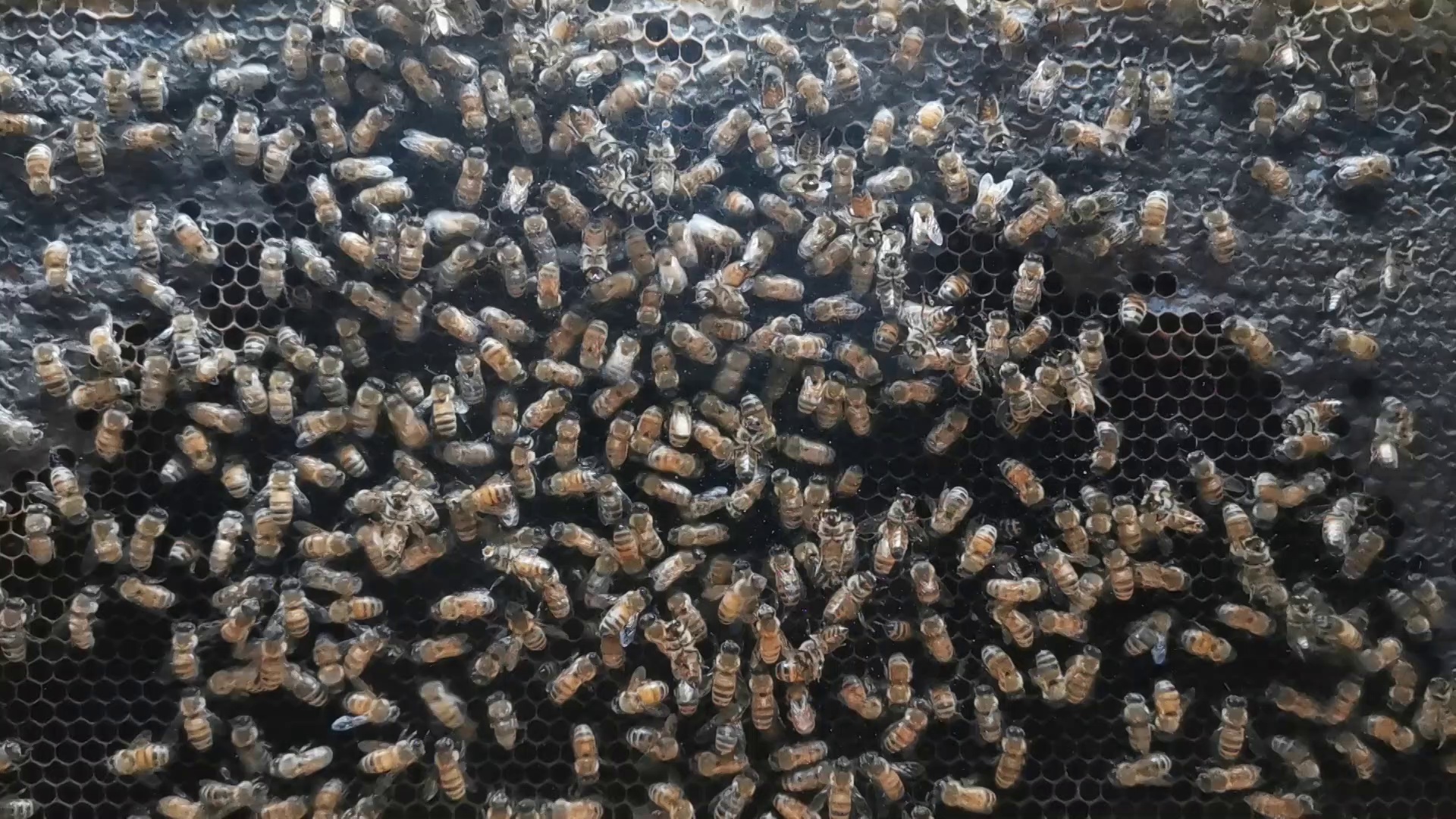

Supplement: Supplementary file 1 — Supplementary Information. [file 41598_2023_44718_MOESM1_ESM.zip › Dataset/dataset-Mask_RCNN_Training/dataset/train/001.jpg]

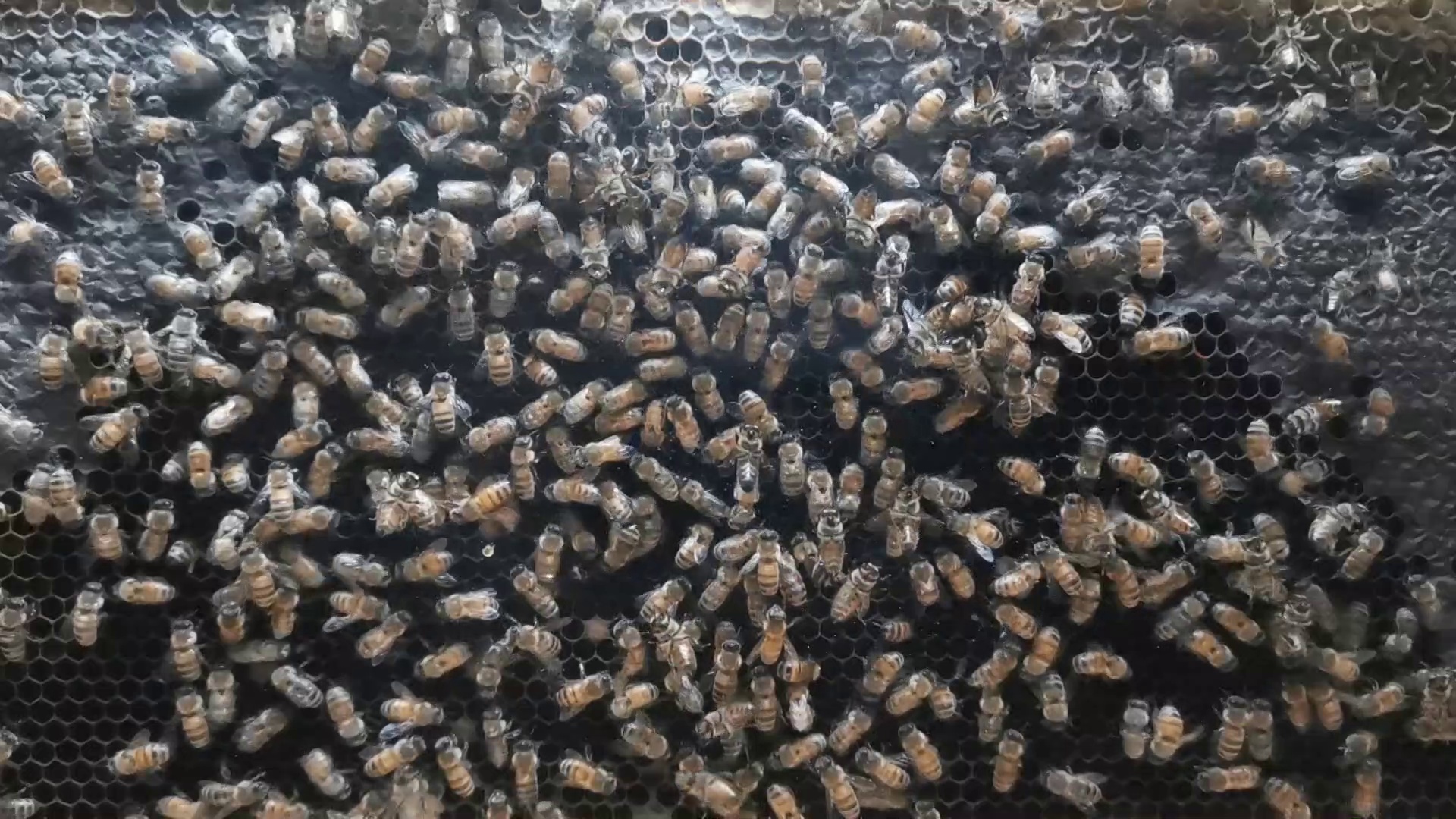

Supplement: Supplementary file 1 — Supplementary Information. [file 41598_2023_44718_MOESM1_ESM.zip › Dataset/dataset-Mask_RCNN_Training/dataset/train/017.jpg]

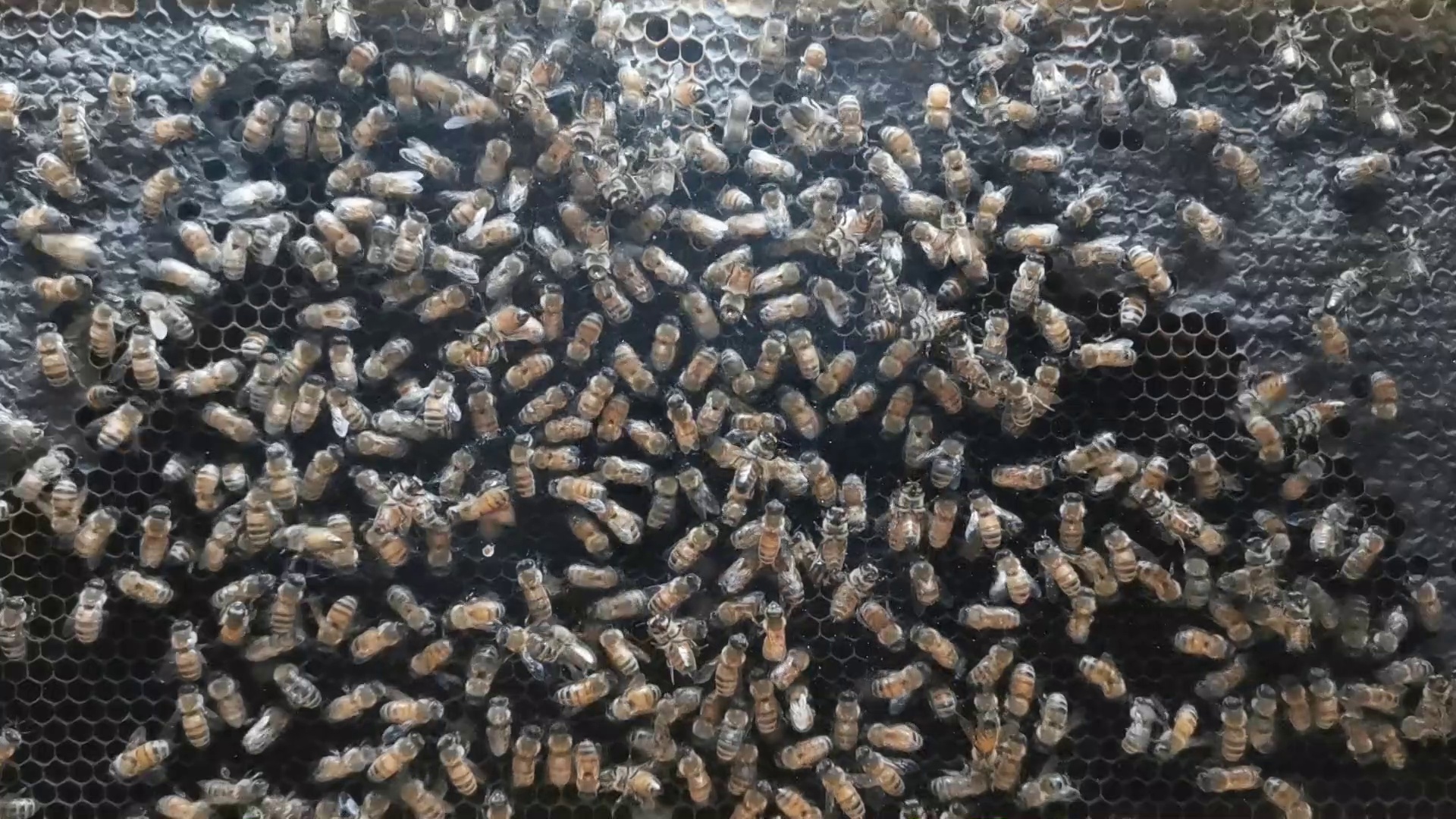

Supplement: Supplementary file 1 — Supplementary Information. [file 41598_2023_44718_MOESM1_ESM.zip › Dataset/dataset-Mask_RCNN_Training/dataset/train/027.jpg]

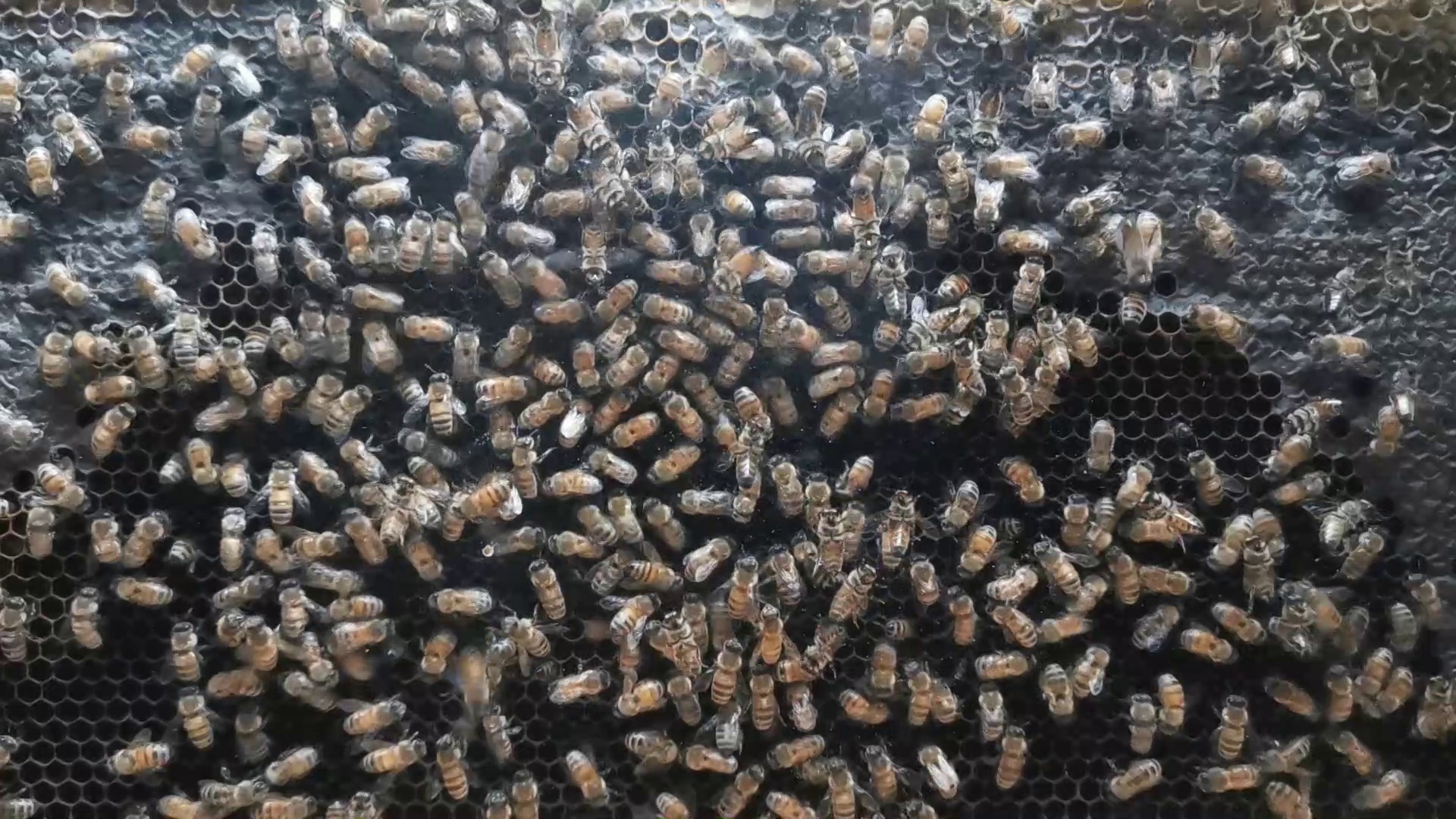

Supplement: Supplementary file 1 — Supplementary Information. [file 41598_2023_44718_MOESM1_ESM.zip › Dataset/dataset-Mask_RCNN_Training/dataset/train/007.jpg]

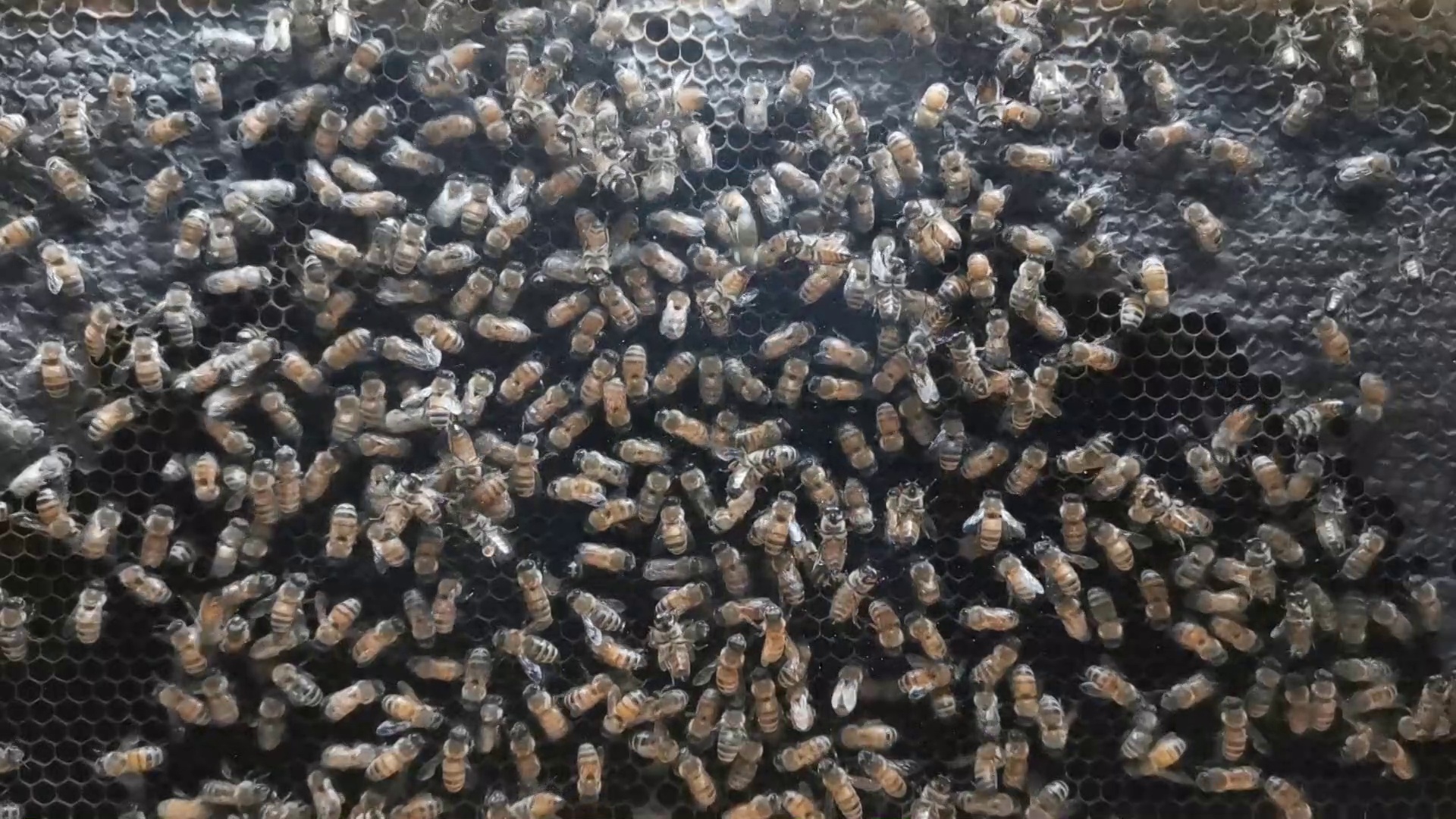

Supplement: Supplementary file 1 — Supplementary Information. [file 41598_2023_44718_MOESM1_ESM.zip › Dataset/dataset-Mask_RCNN_Training/dataset/train/033.jpg]

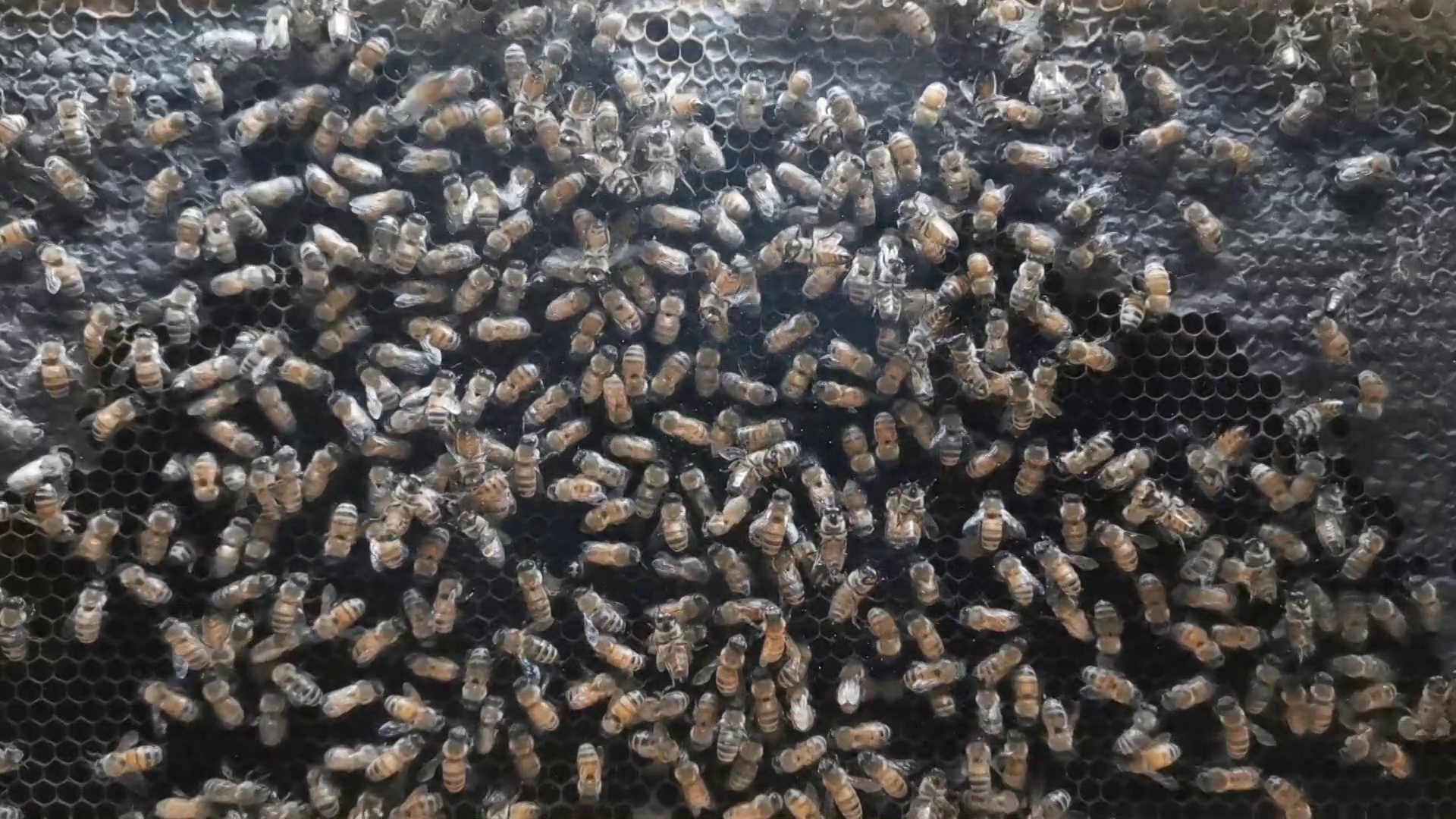

Supplement: Supplementary file 1 — Supplementary Information. [file 41598_2023_44718_MOESM1_ESM.zip › Dataset/dataset-Mask_RCNN_Training/dataset/train/034.jpg]

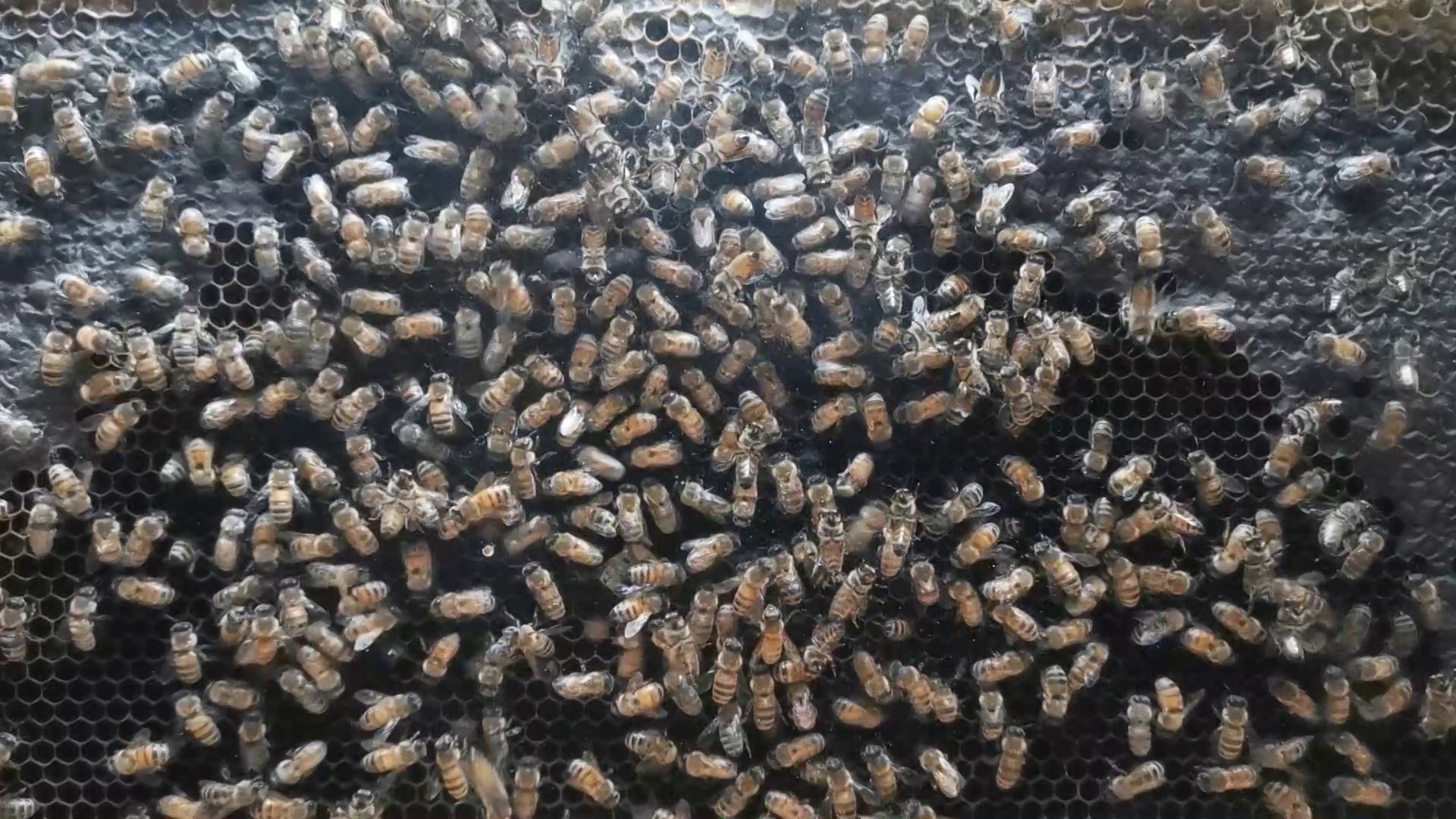

Supplement: Supplementary file 1 — Supplementary Information. [file 41598_2023_44718_MOESM1_ESM.zip › Dataset/dataset-Mask_RCNN_Training/dataset/train/009.jpg]

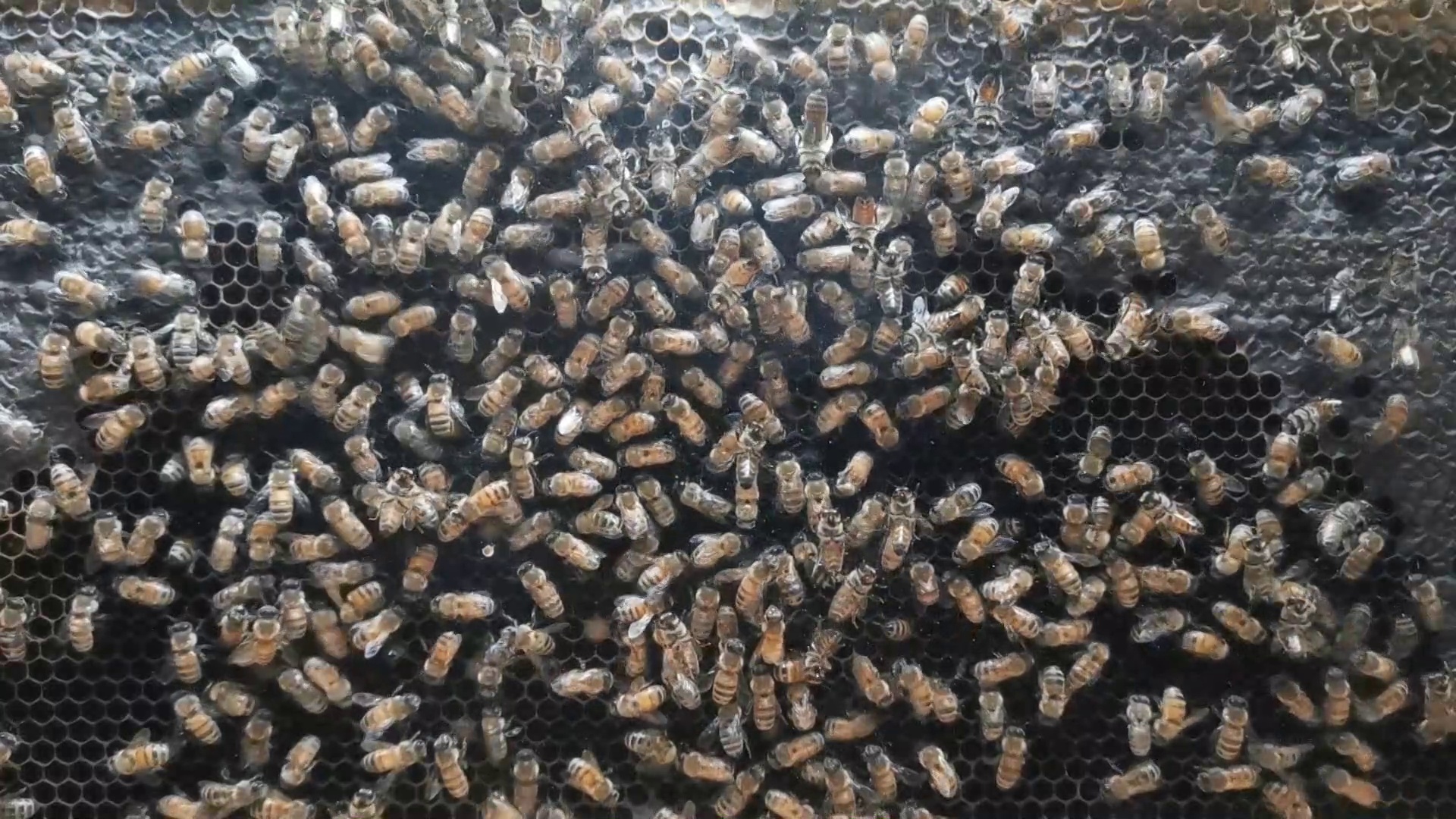

Supplement: Supplementary file 1 — Supplementary Information. [file 41598_2023_44718_MOESM1_ESM.zip › Dataset/dataset-Mask_RCNN_Training/dataset/train/010.jpg]

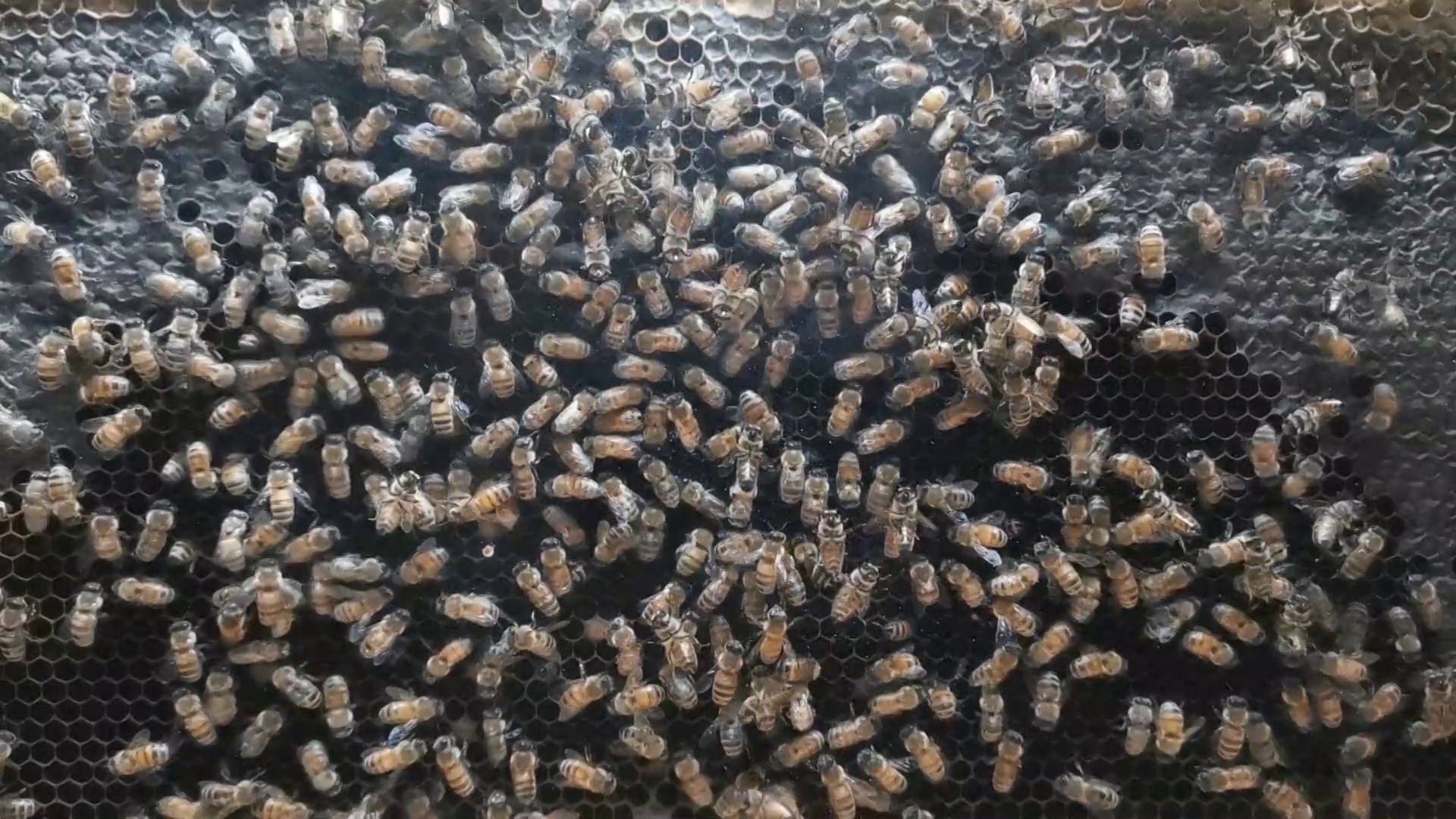

Supplement: Supplementary file 1 — Supplementary Information. [file 41598_2023_44718_MOESM1_ESM.zip › Dataset/dataset-Mask_RCNN_Training/dataset/train/015.jpg]

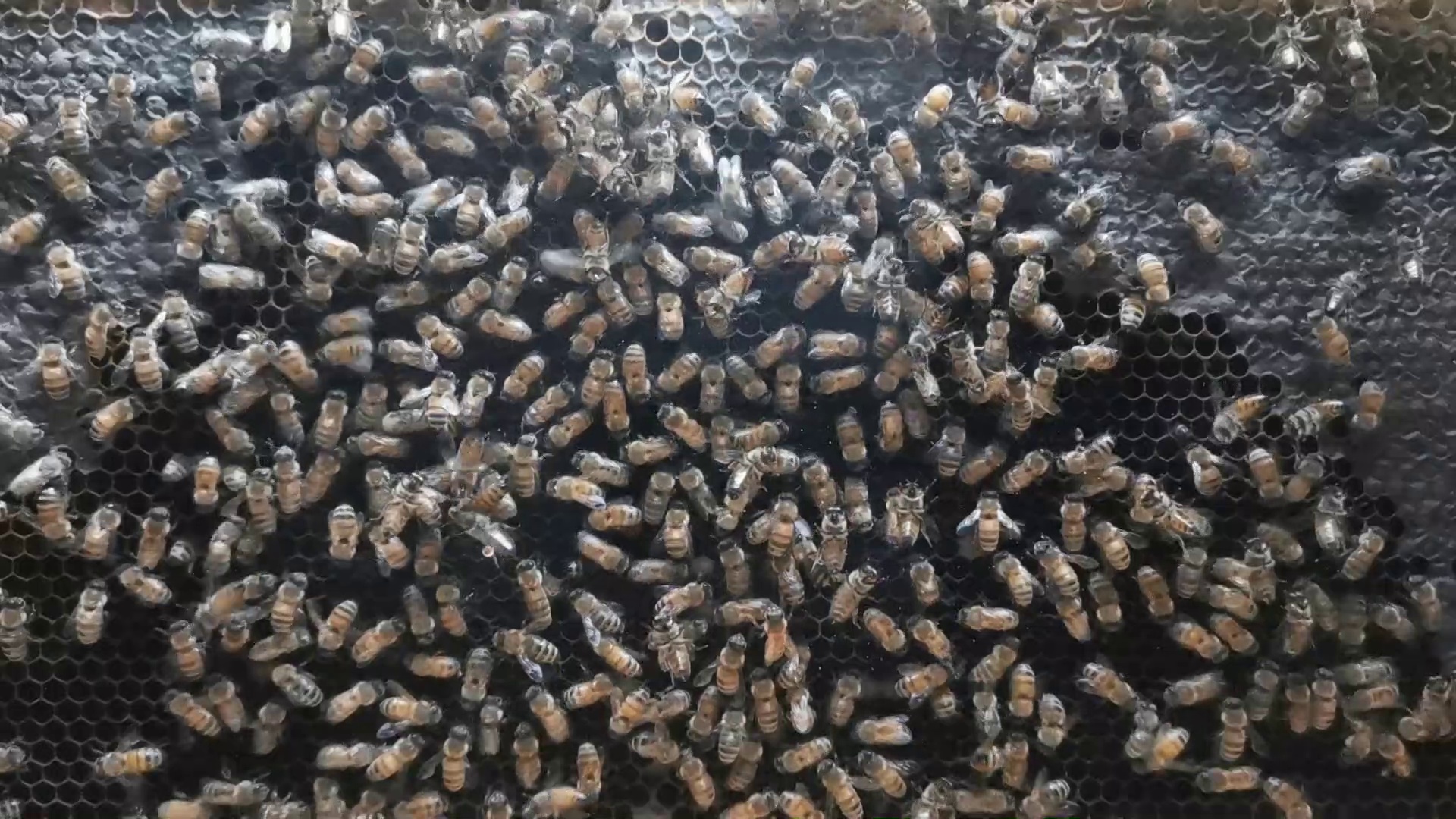

Supplement: Supplementary file 1 — Supplementary Information. [file 41598_2023_44718_MOESM1_ESM.zip › Dataset/dataset-Mask_RCNN_Training/dataset/train/032.jpg]

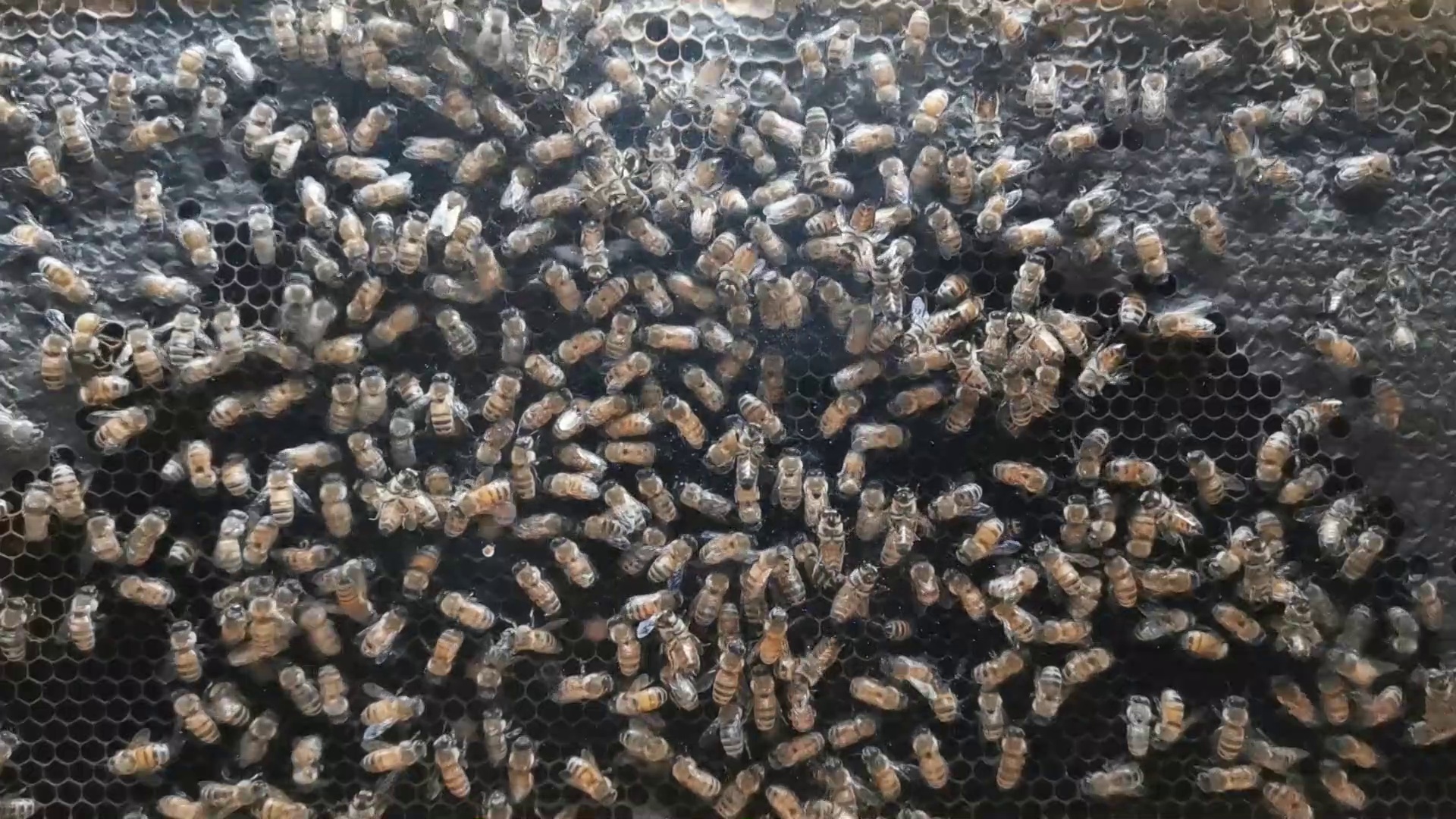

Supplement: Supplementary file 1 — Supplementary Information. [file 41598_2023_44718_MOESM1_ESM.zip › Dataset/dataset-Mask_RCNN_Training/dataset/train/012.jpg]

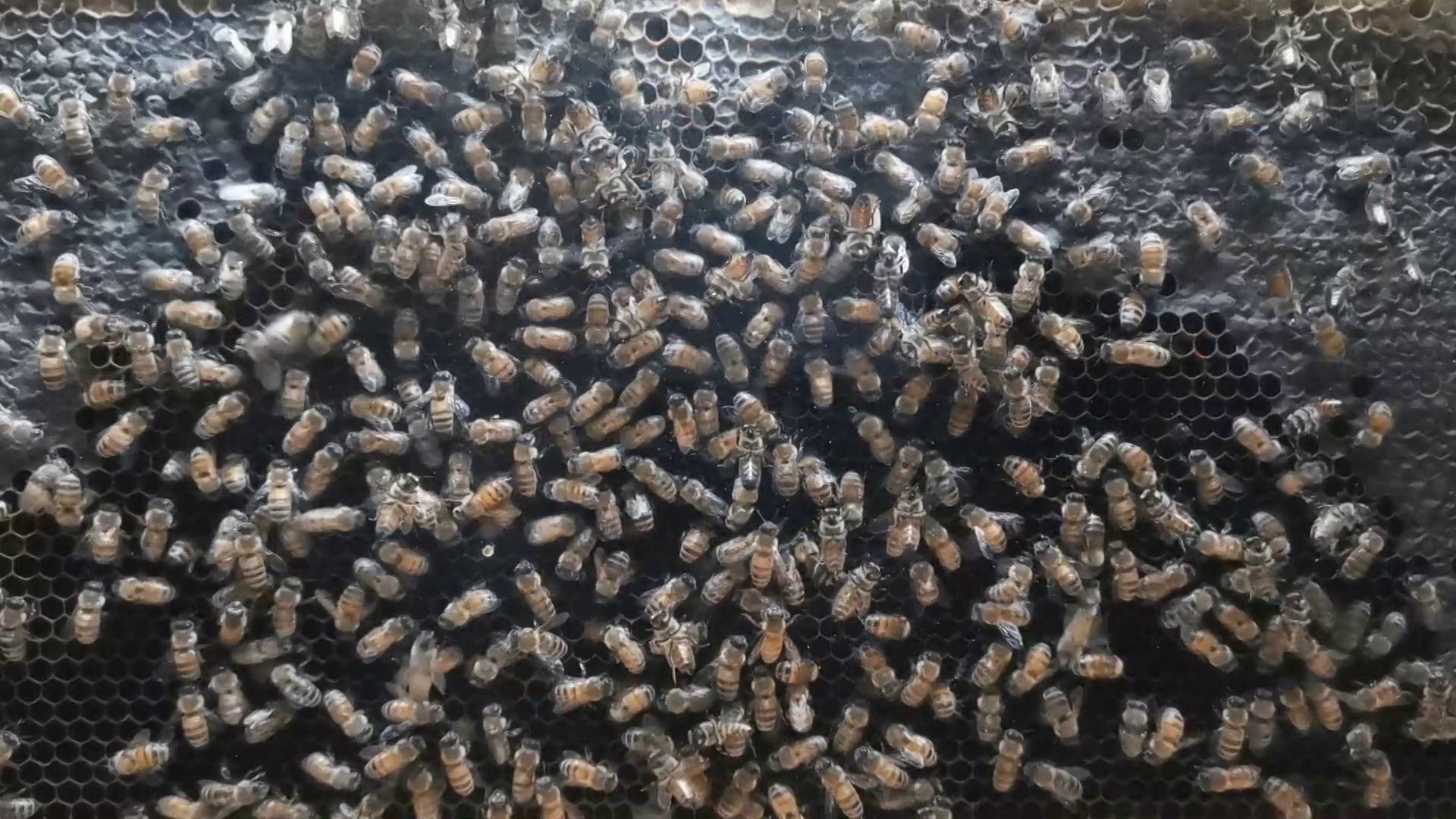

Supplement: Supplementary file 1 — Supplementary Information. [file 41598_2023_44718_MOESM1_ESM.zip › Dataset/dataset-Mask_RCNN_Training/dataset/train/020.jpg]

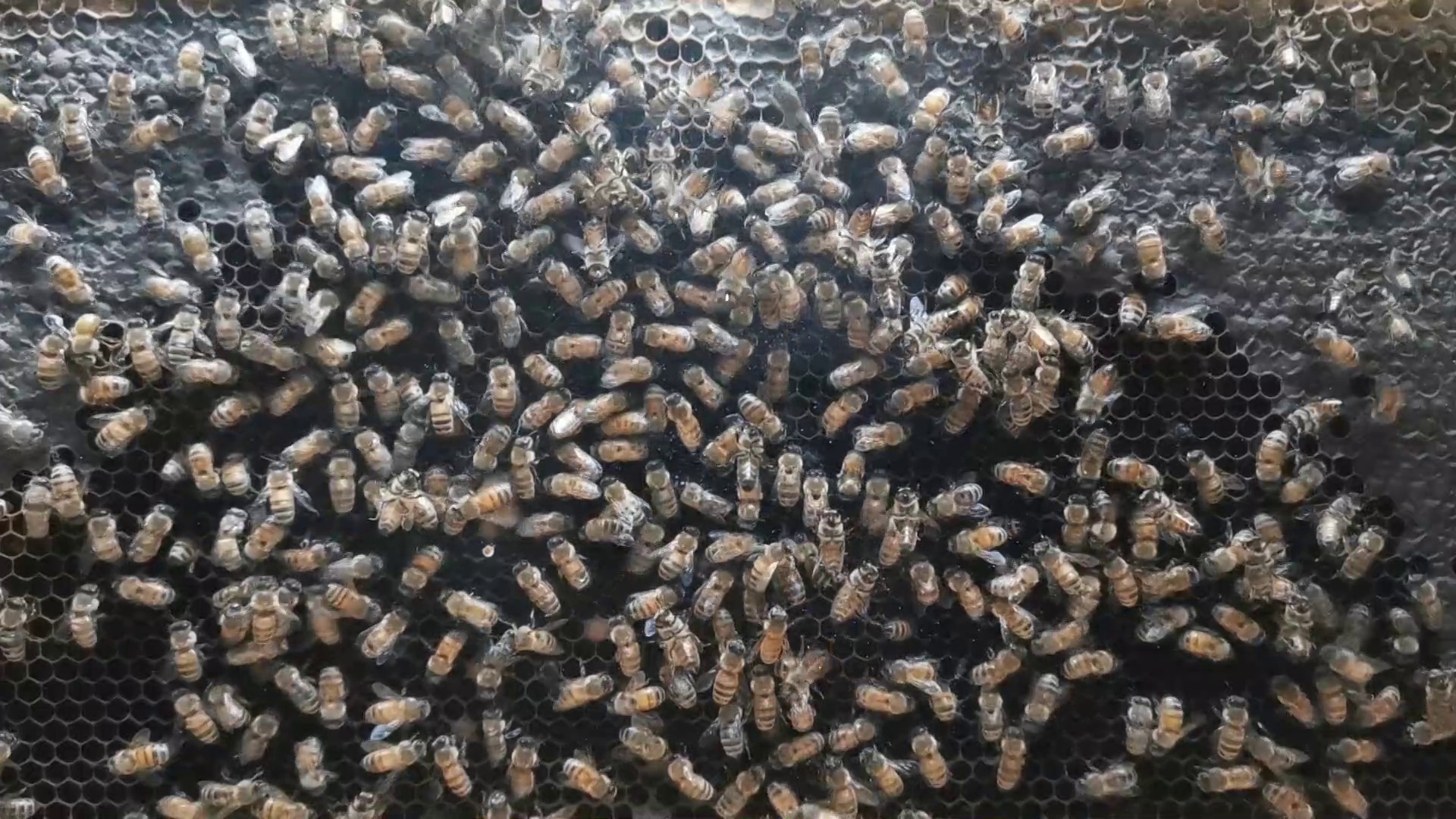

Supplement: Supplementary file 1 — Supplementary Information. [file 41598_2023_44718_MOESM1_ESM.zip › Dataset/dataset-Mask_RCNN_Training/dataset/train/013.jpg]

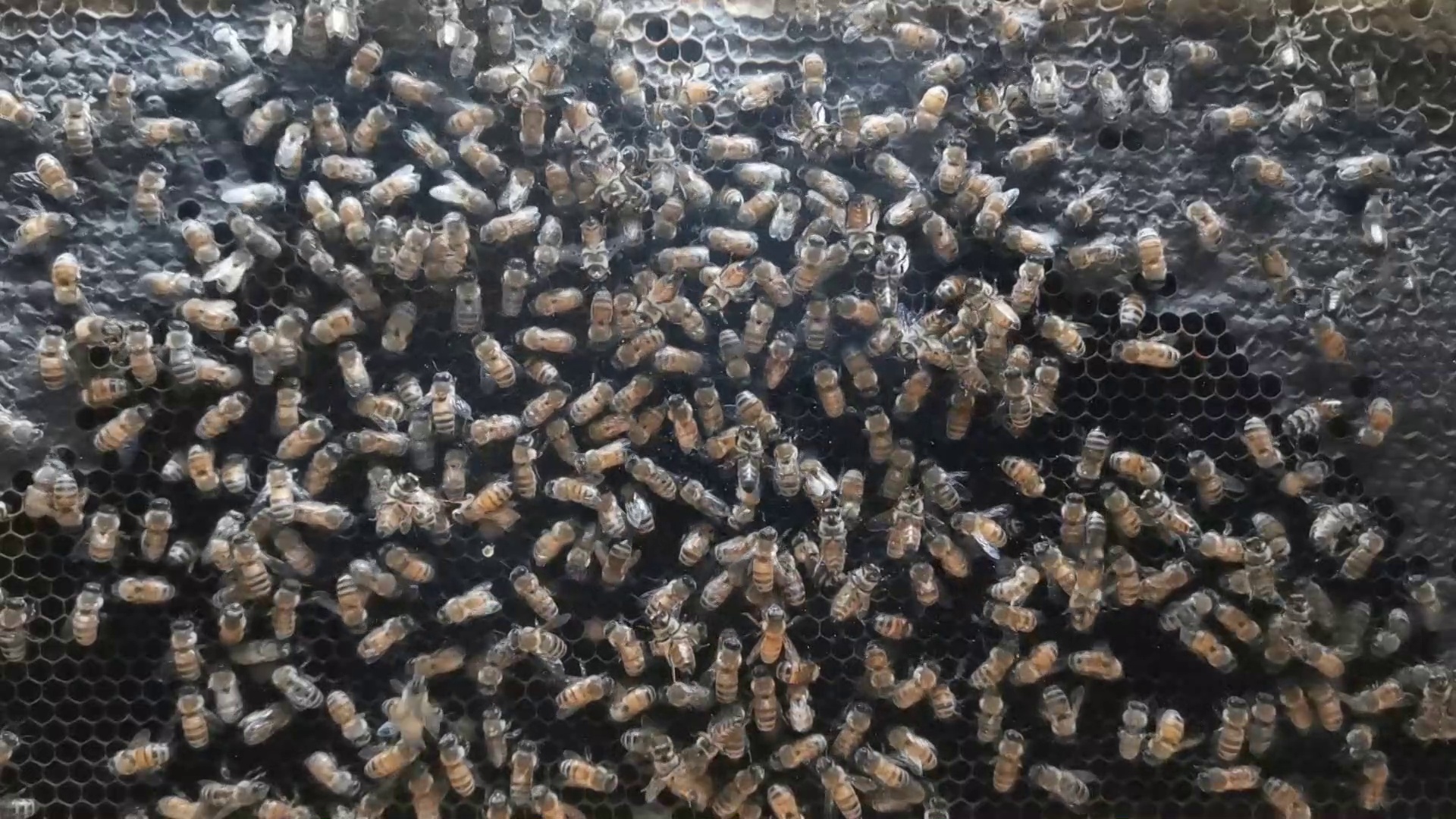

Supplement: Supplementary file 1 — Supplementary Information. [file 41598_2023_44718_MOESM1_ESM.zip › Dataset/dataset-Mask_RCNN_Training/dataset/train/019.jpg]

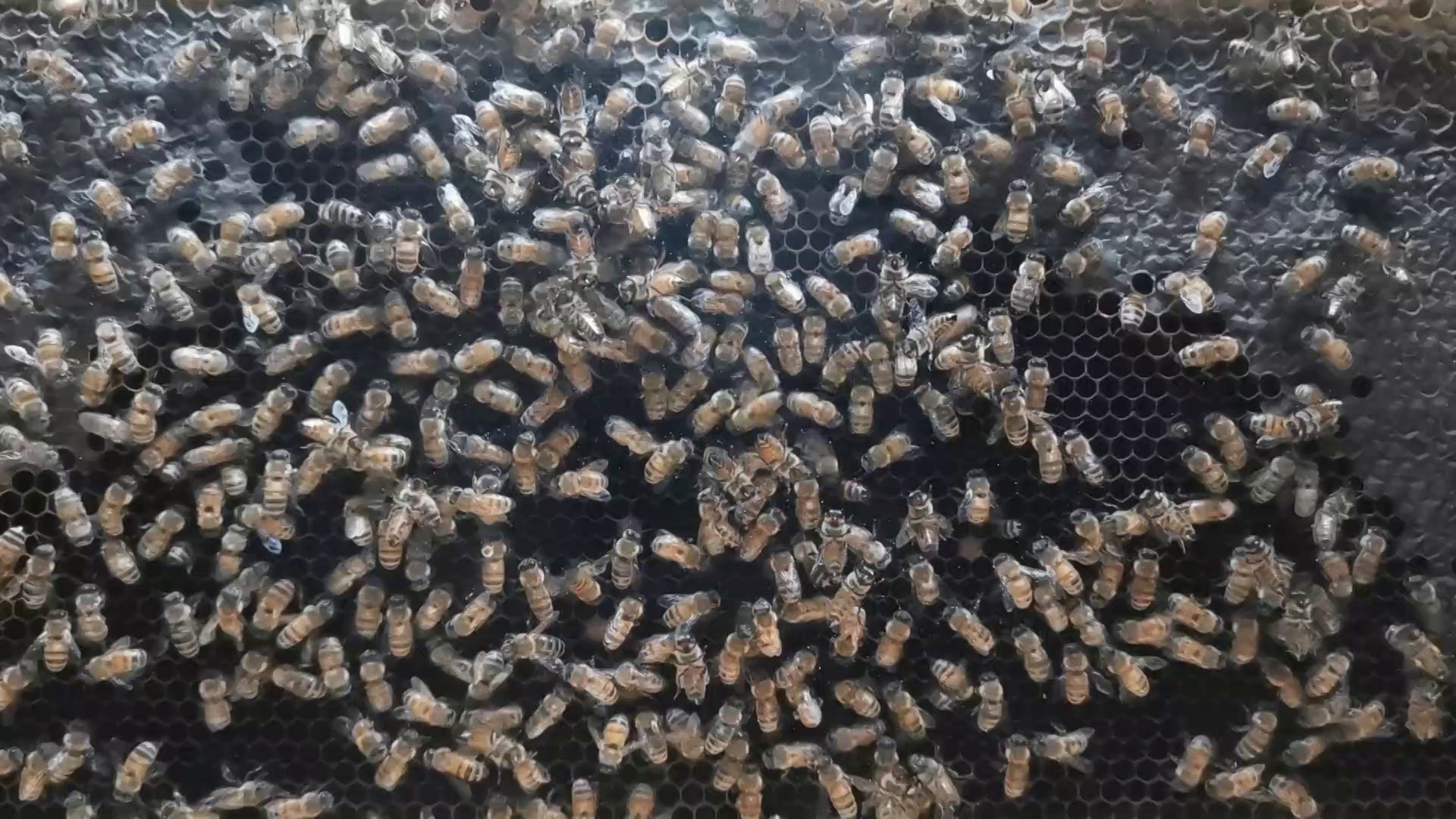

Supplement: Supplementary file 1 — Supplementary Information. [file 41598_2023_44718_MOESM1_ESM.zip › Dataset/dataset-Mask_RCNN_Training/dataset/val/001.jpg]

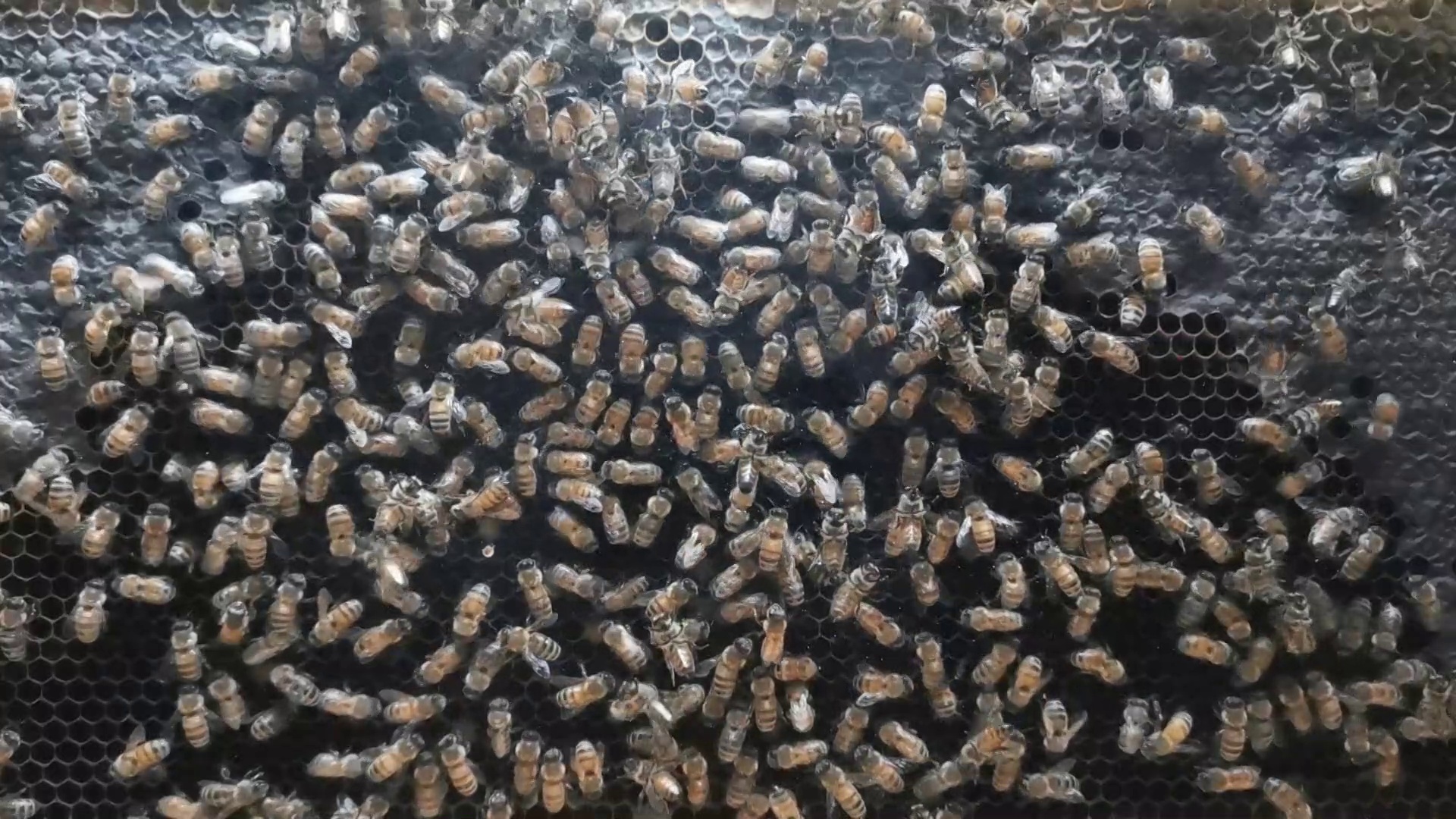

Supplement: Supplementary file 1 — Supplementary Information. [file 41598_2023_44718_MOESM1_ESM.zip › Dataset/dataset-Mask_RCNN_Training/dataset/train/024.jpg]

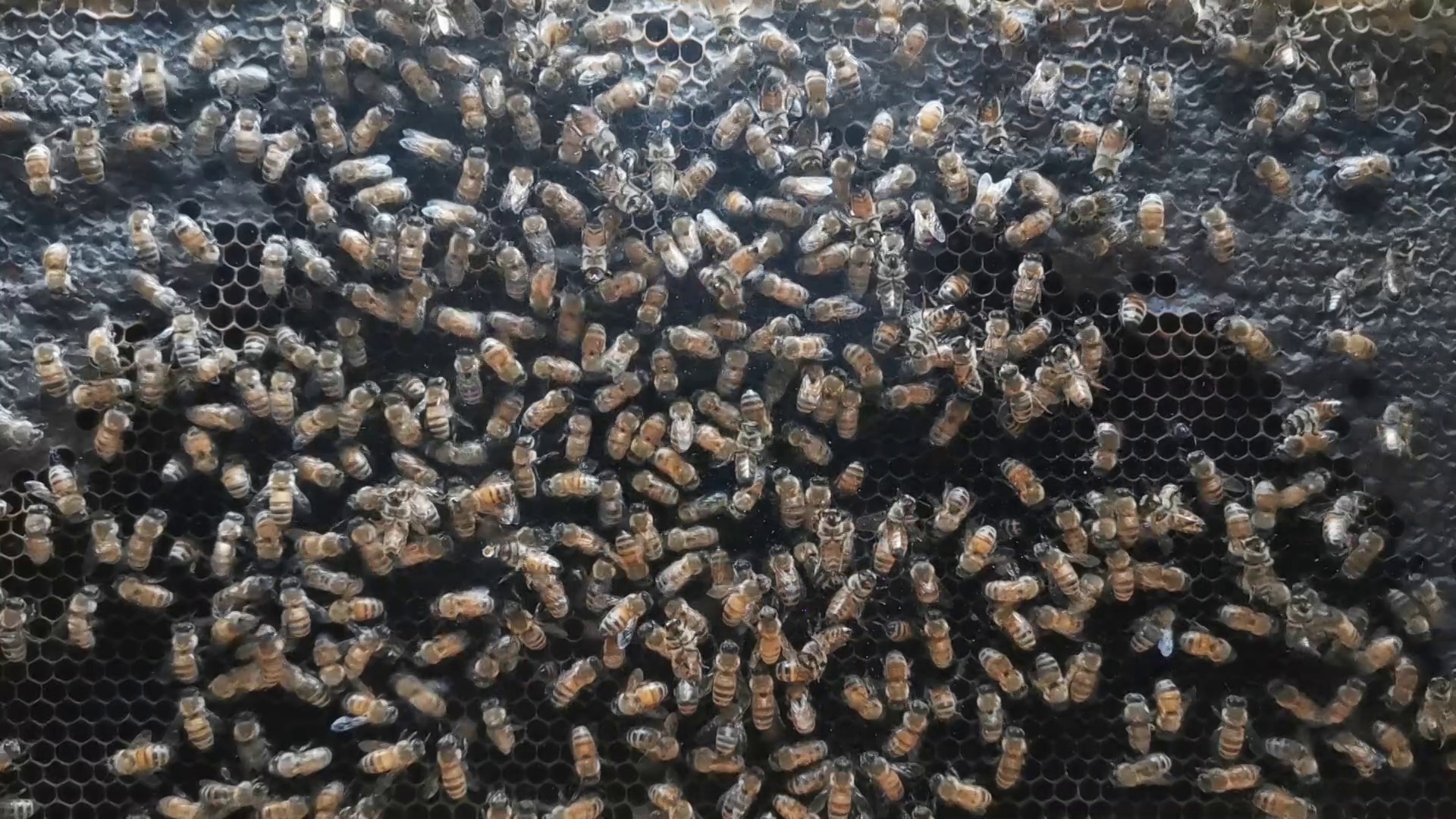

Supplement: Supplementary file 1 — Supplementary Information. [file 41598_2023_44718_MOESM1_ESM.zip › Dataset/dataset-Mask_RCNN_Training/dataset/train/002.jpg]

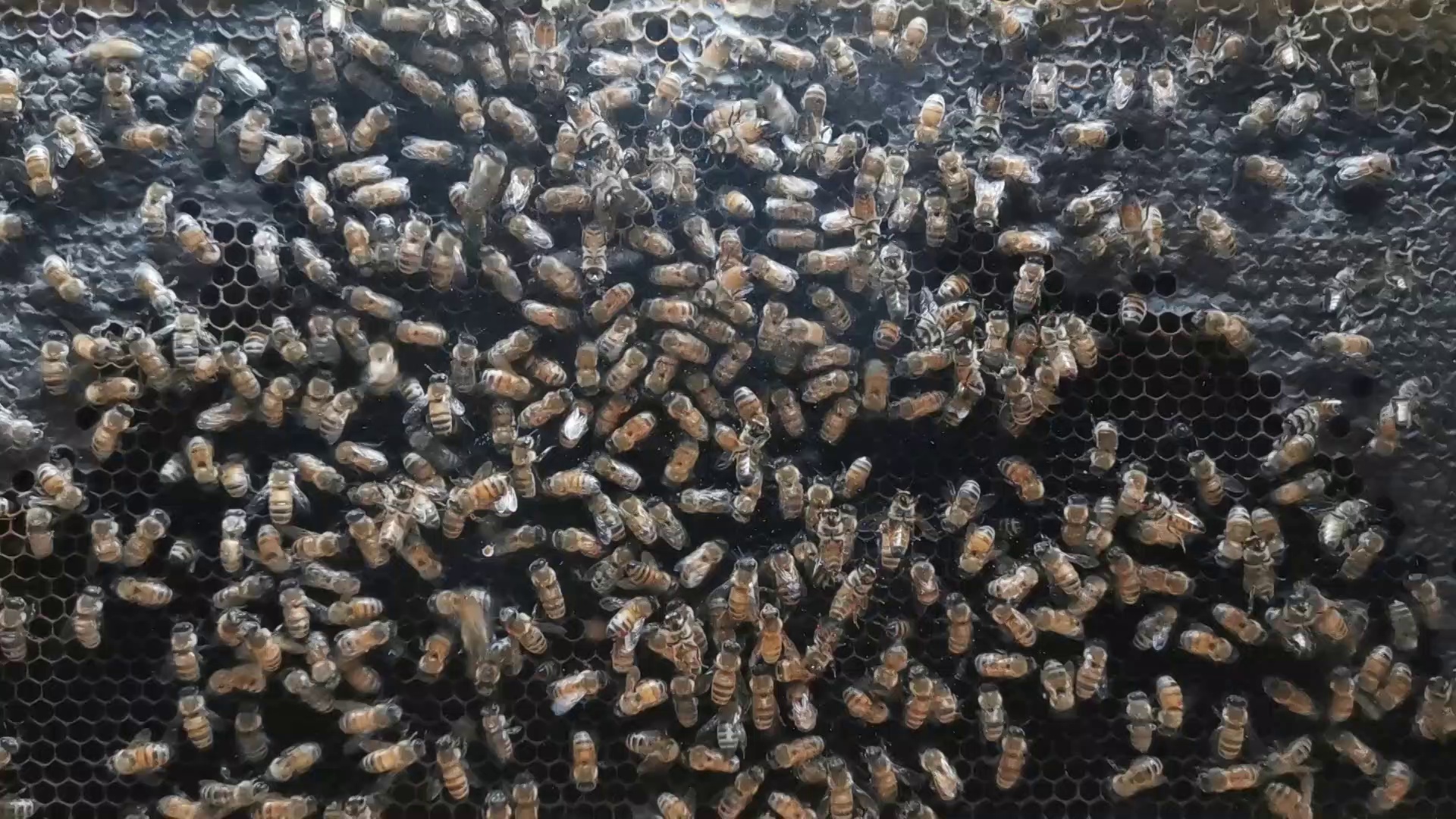

Supplement: Supplementary file 1 — Supplementary Information. [file 41598_2023_44718_MOESM1_ESM.zip › Dataset/dataset-Mask_RCNN_Training/dataset/train/006.jpg]

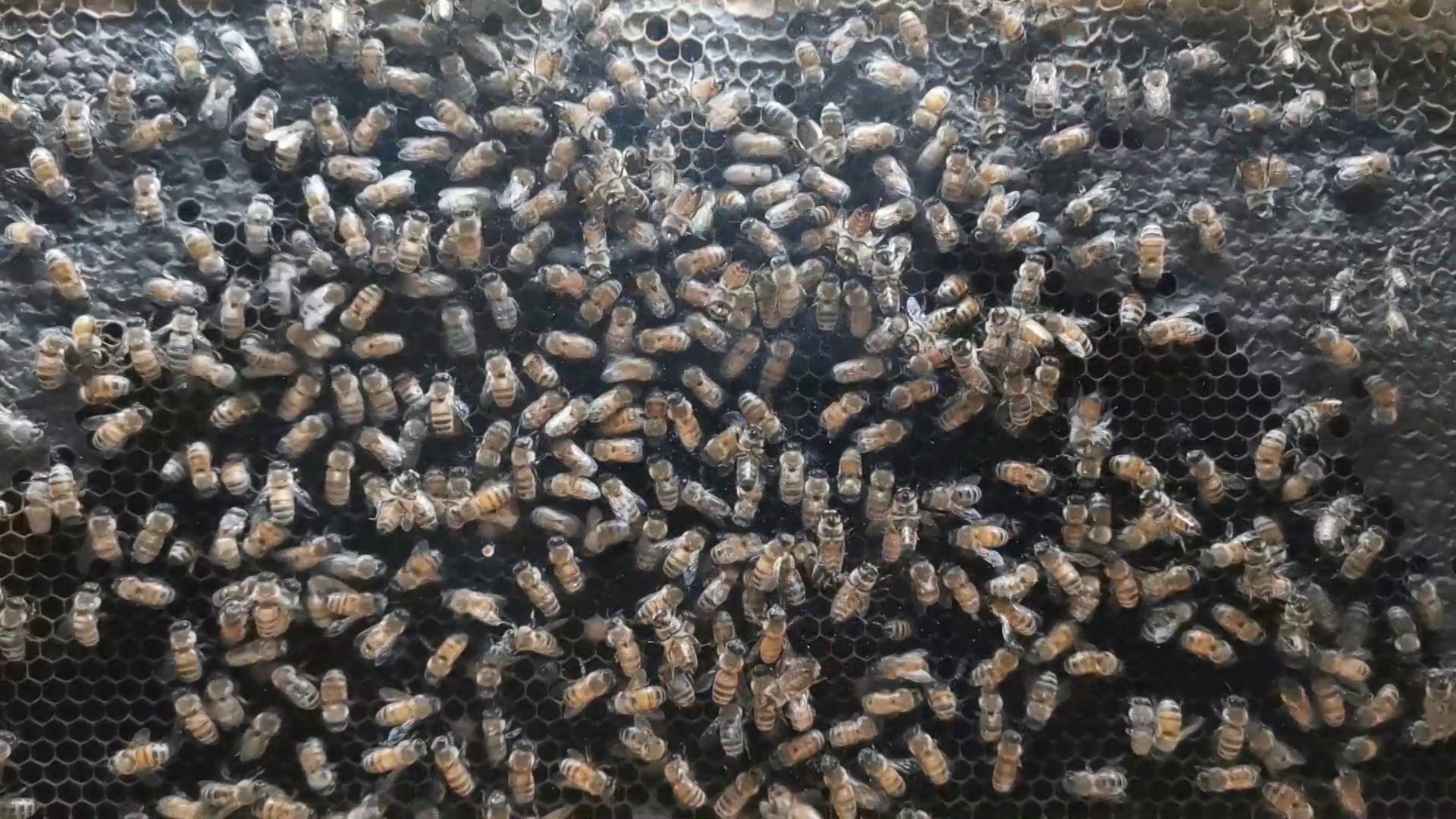

Supplement: Supplementary file 1 — Supplementary Information. [file 41598_2023_44718_MOESM1_ESM.zip › Dataset/dataset-Mask_RCNN_Training/dataset/train/014.jpg]

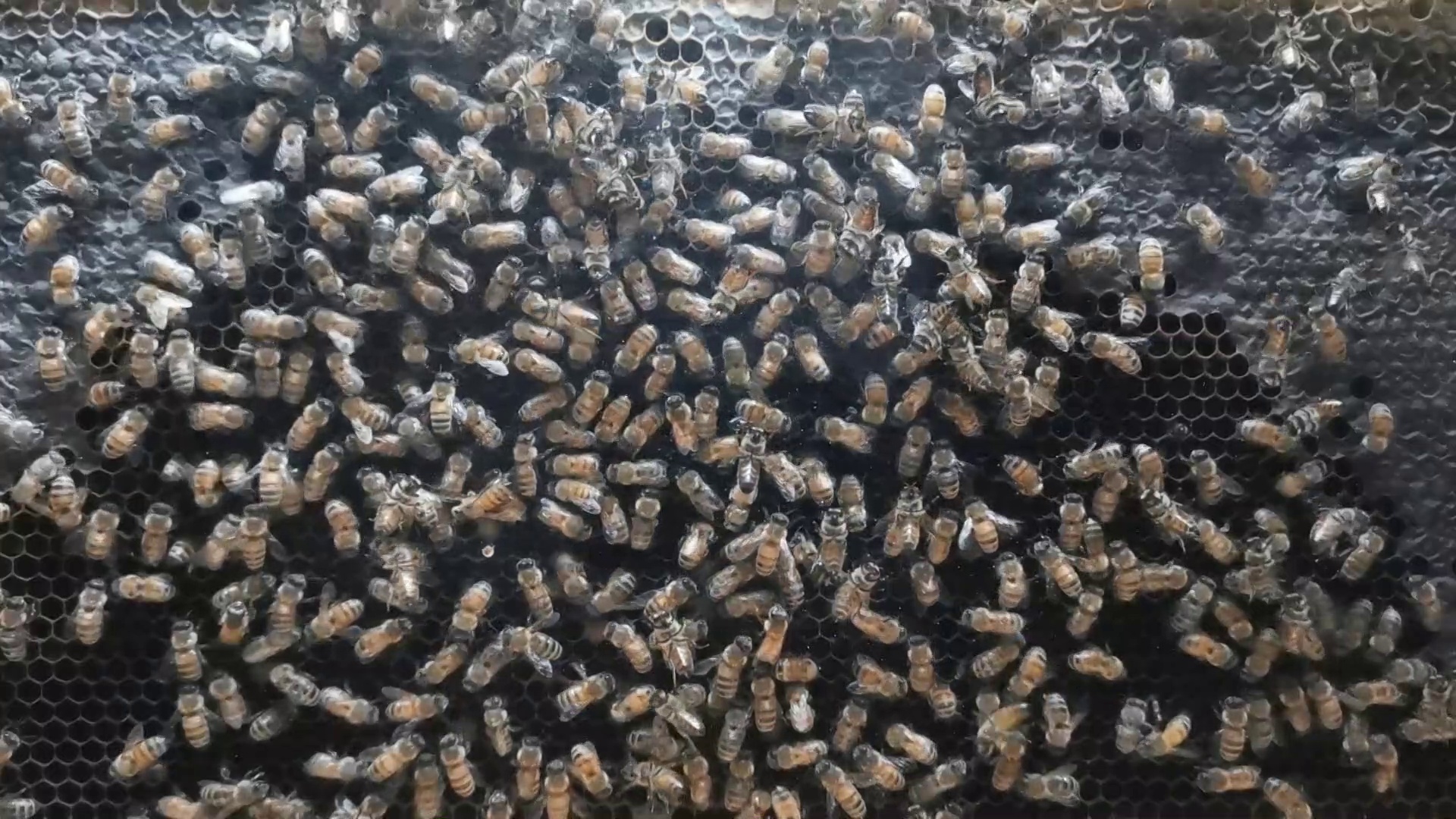

Supplement: Supplementary file 1 — Supplementary Information. [file 41598_2023_44718_MOESM1_ESM.zip › Dataset/dataset-Mask_RCNN_Training/dataset/train/023.jpg]

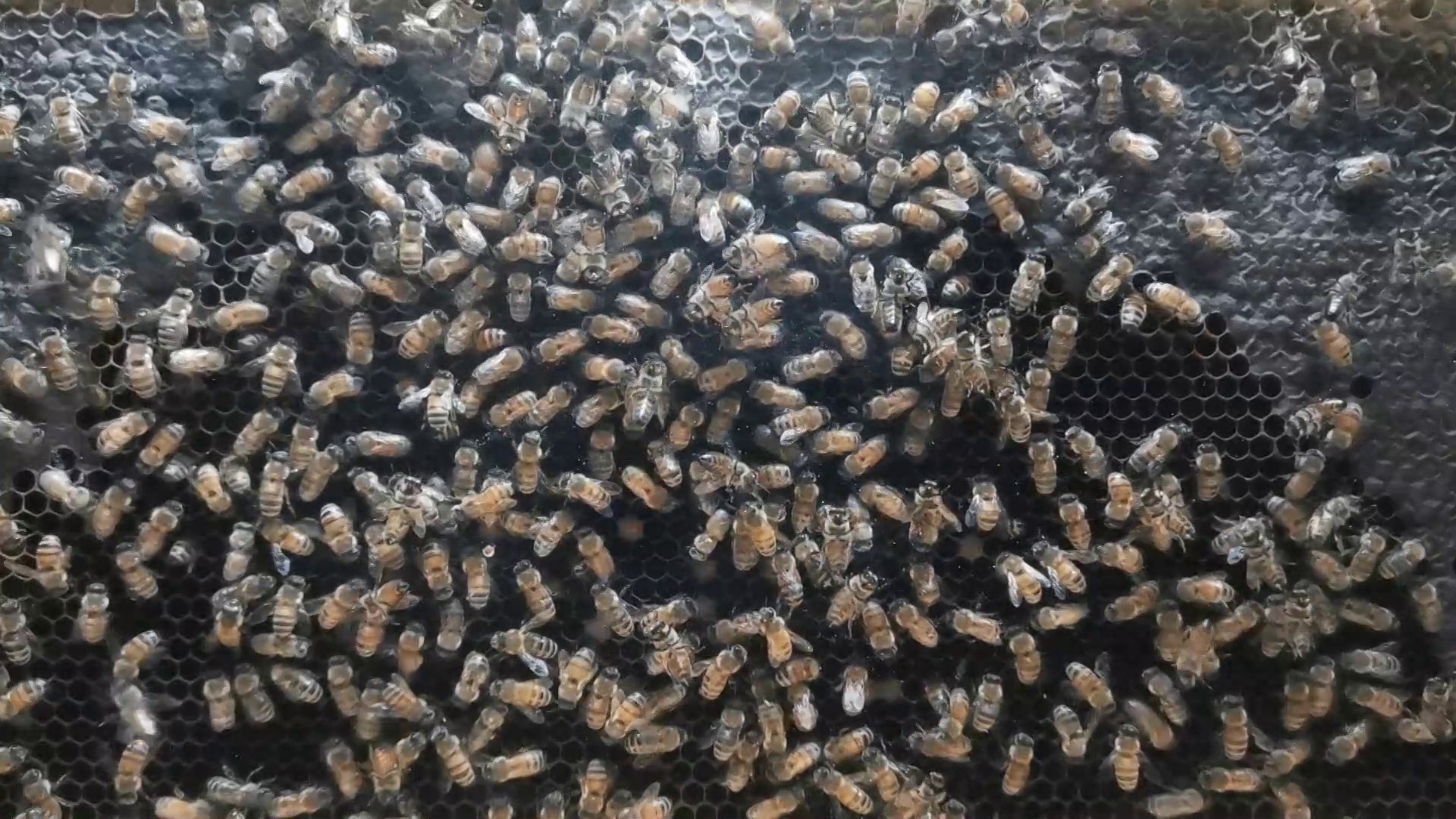

Supplement: Supplementary file 1 — Supplementary Information. [file 41598_2023_44718_MOESM1_ESM.zip › Dataset/test set-system_evaluation/test_set_15fps/143.jpg]

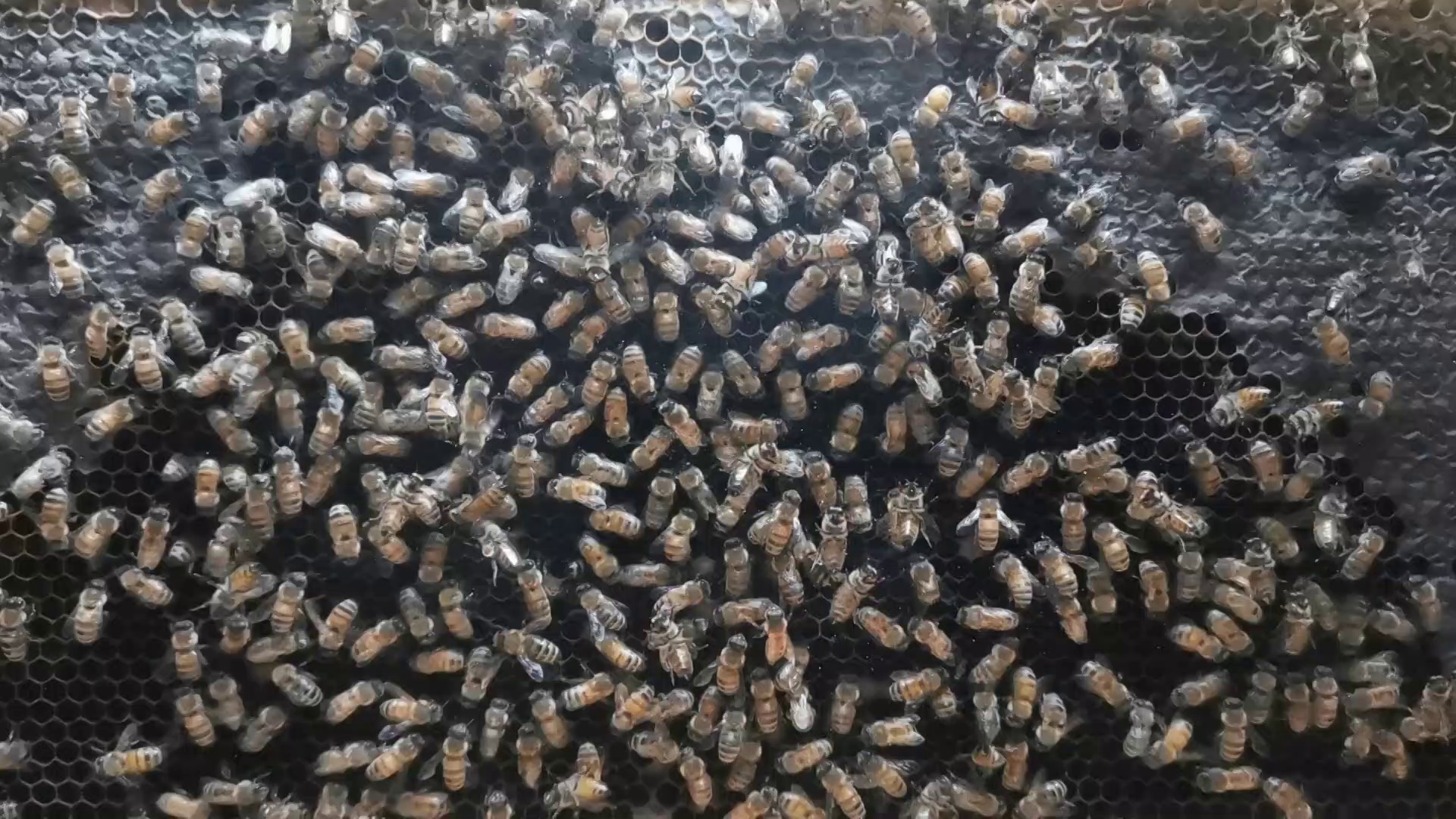

Supplement: Supplementary file 1 — Supplementary Information. [file 41598_2023_44718_MOESM1_ESM.zip › Dataset/dataset-Mask_RCNN_Training/dataset/train/031.jpg]

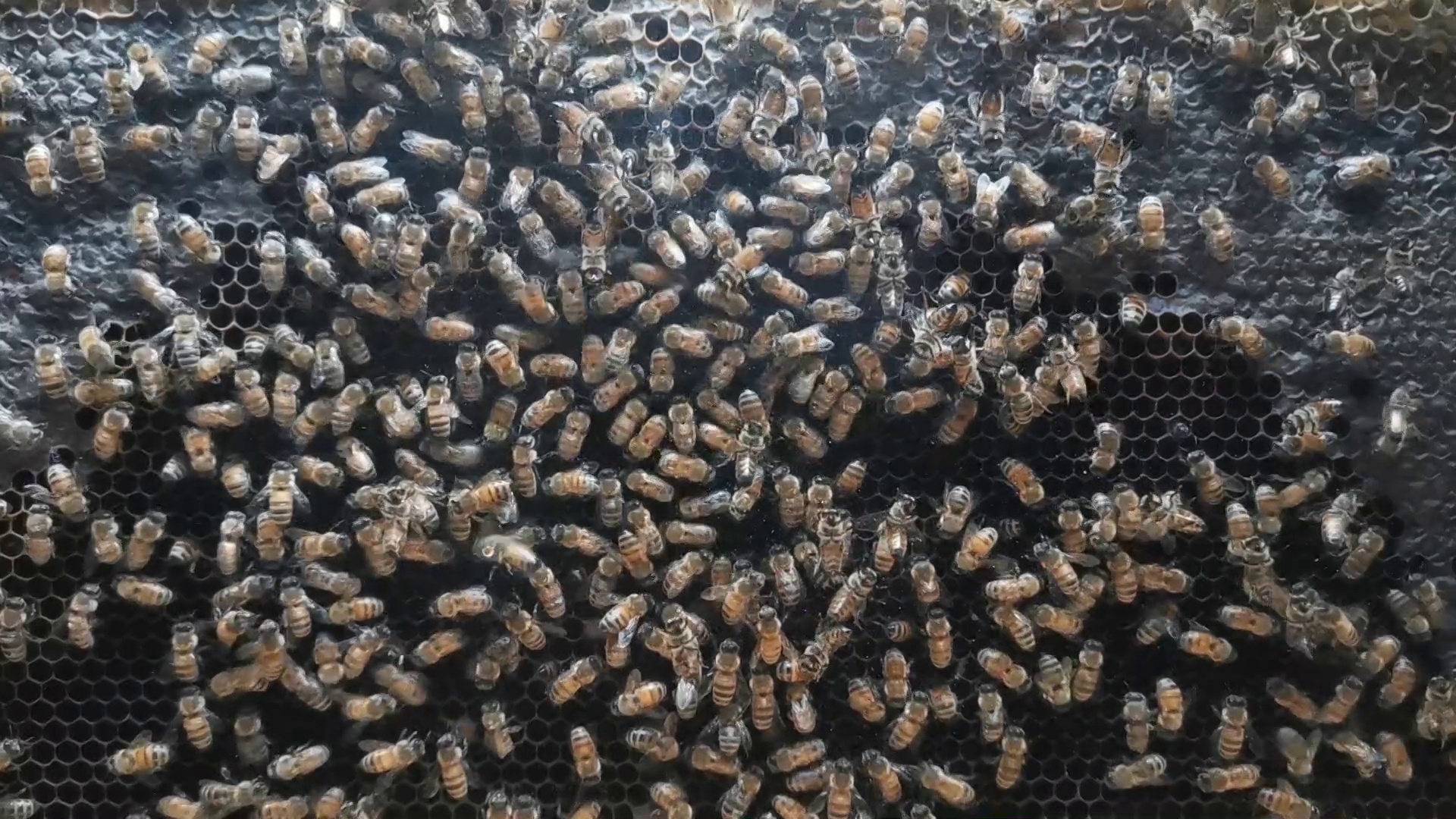

Supplement: Supplementary file 1 — Supplementary Information. [file 41598_2023_44718_MOESM1_ESM.zip › Dataset/dataset-Mask_RCNN_Training/dataset/train/003.jpg]

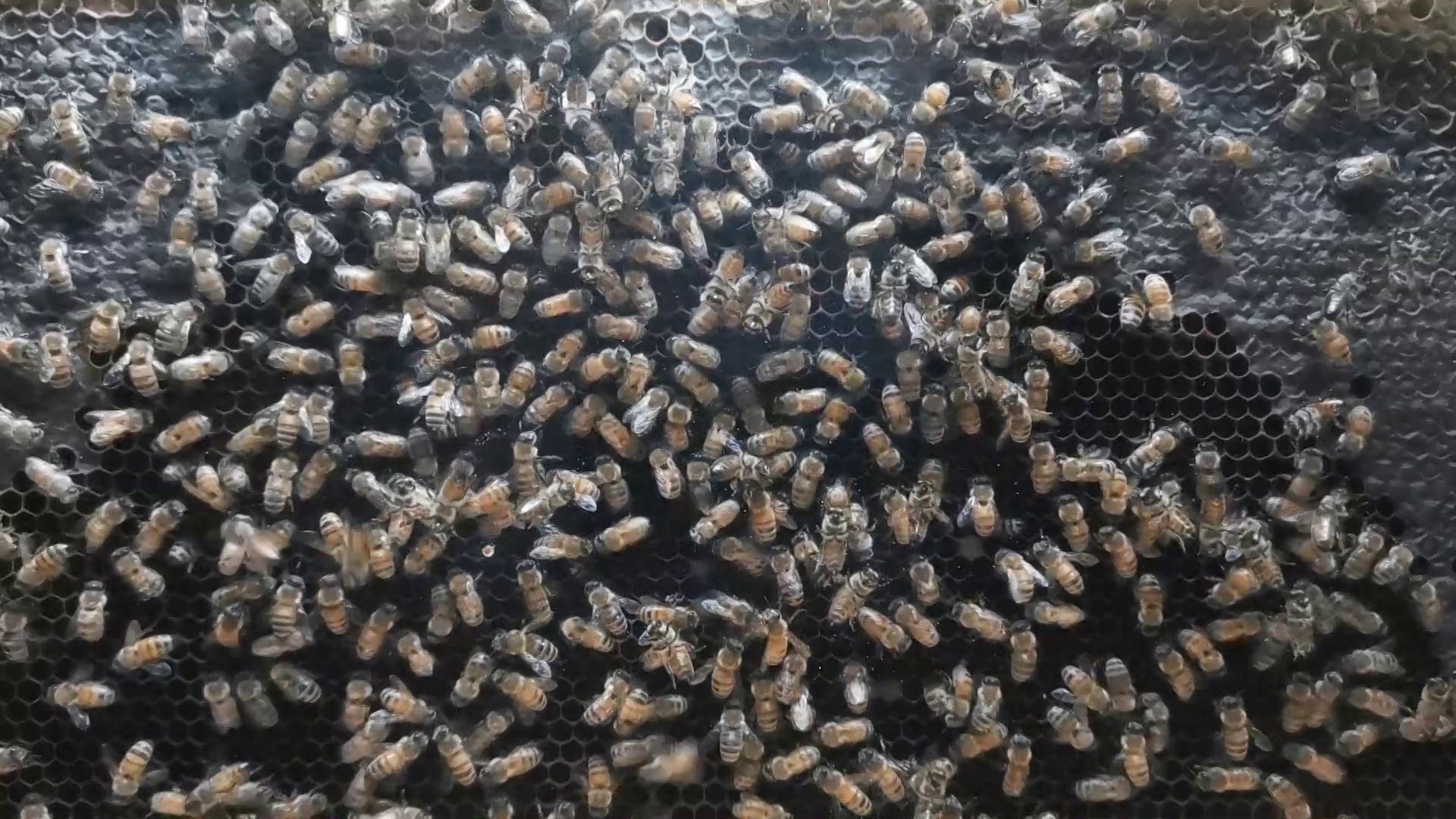

Supplement: Supplementary file 1 — Supplementary Information. [file 41598_2023_44718_MOESM1_ESM.zip › Dataset/test set-system_evaluation/test_set_15fps/125.jpg]

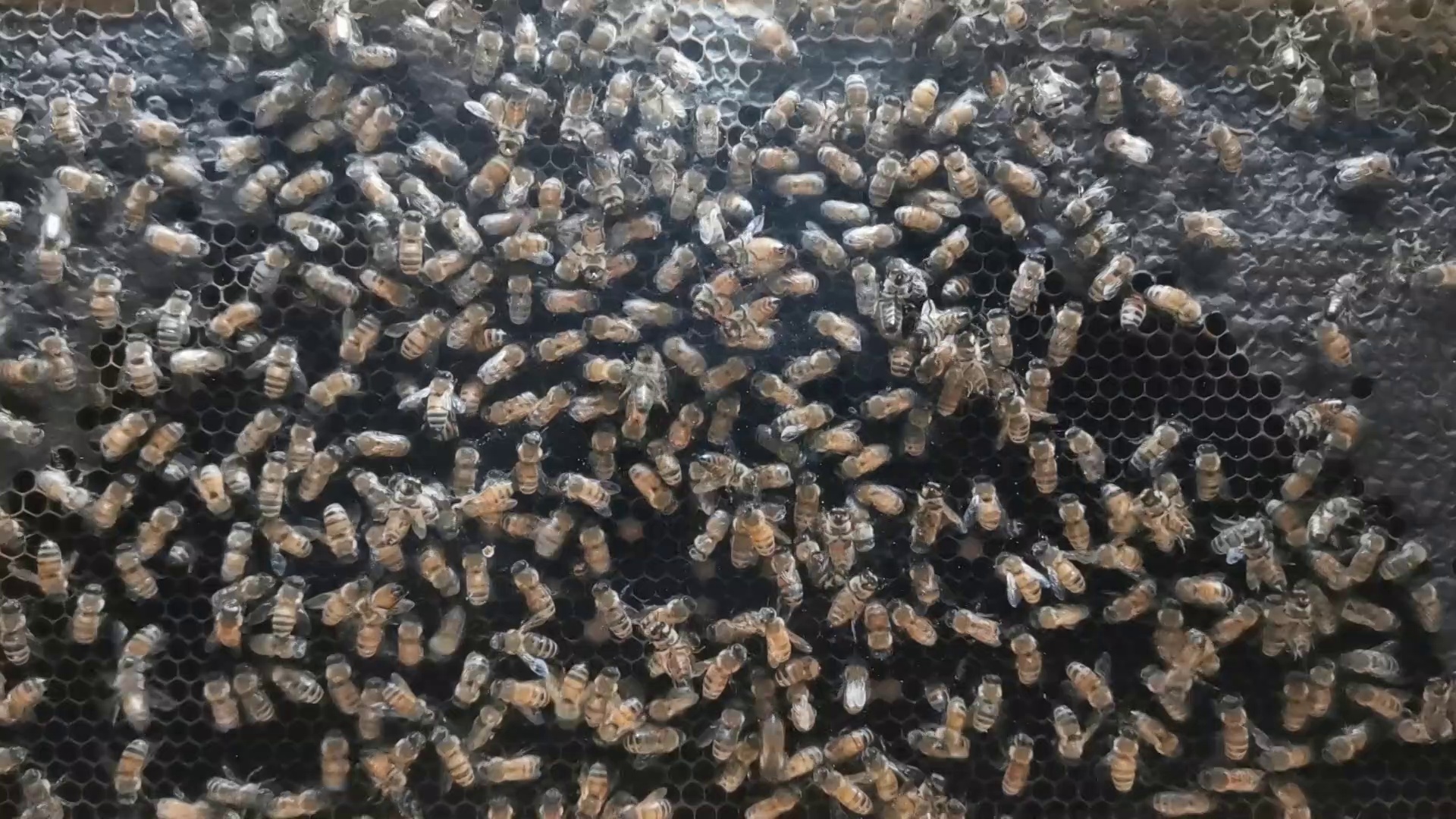

Supplement: Supplementary file 1 — Supplementary Information. [file 41598_2023_44718_MOESM1_ESM.zip › Dataset/test set-system_evaluation/test_set_15fps/145.jpg]

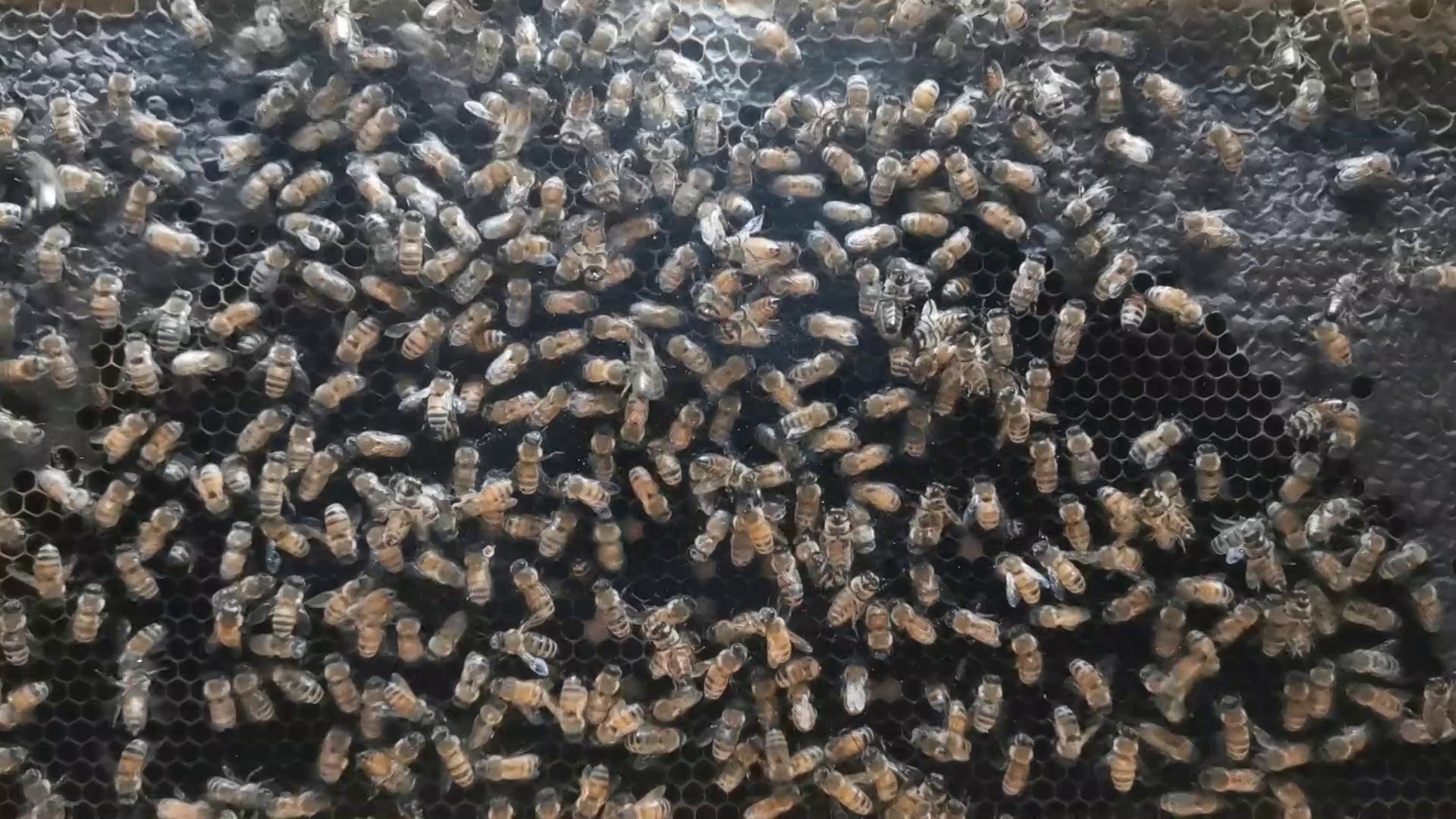

Supplement: Supplementary file 1 — Supplementary Information. [file 41598_2023_44718_MOESM1_ESM.zip › Dataset/test set-system_evaluation/test_set_15fps/146.jpg]

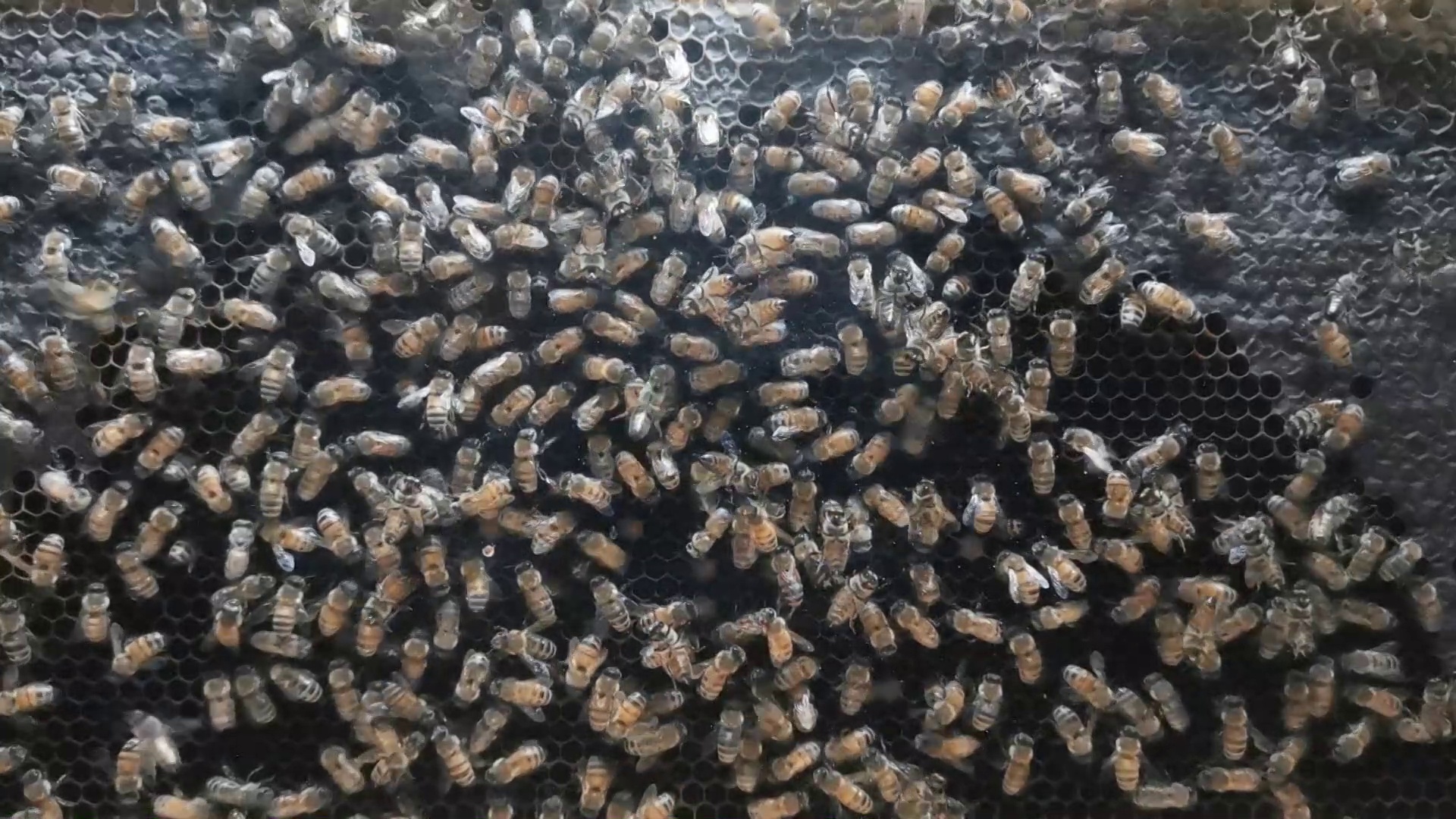

Supplement: Supplementary file 1 — Supplementary Information. [file 41598_2023_44718_MOESM1_ESM.zip › Dataset/test set-system_evaluation/test_set_15fps/140.jpg]

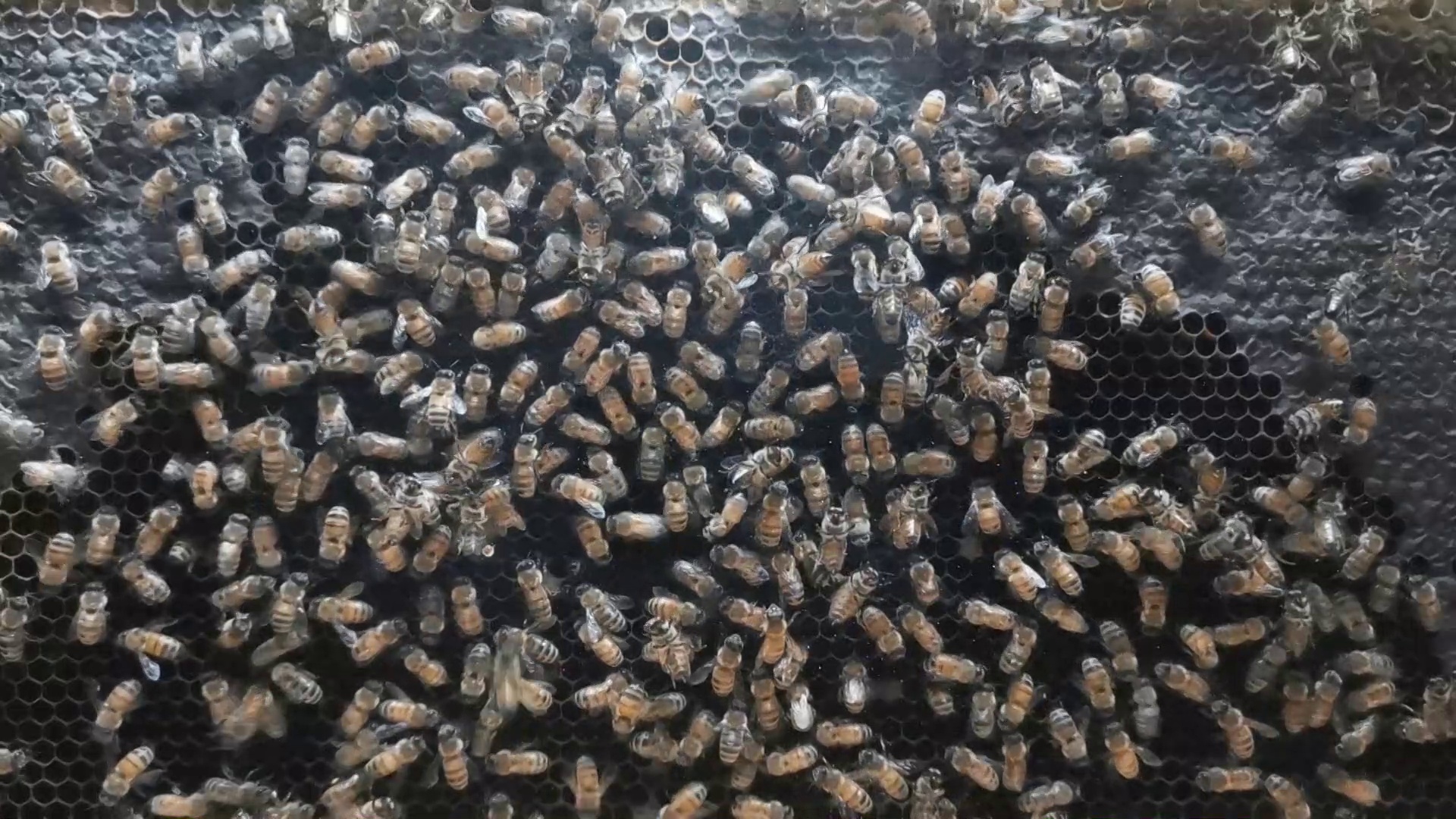

Supplement: Supplementary file 1 — Supplementary Information. [file 41598_2023_44718_MOESM1_ESM.zip › Dataset/test set-system_evaluation/test_set_15fps/110.jpg]

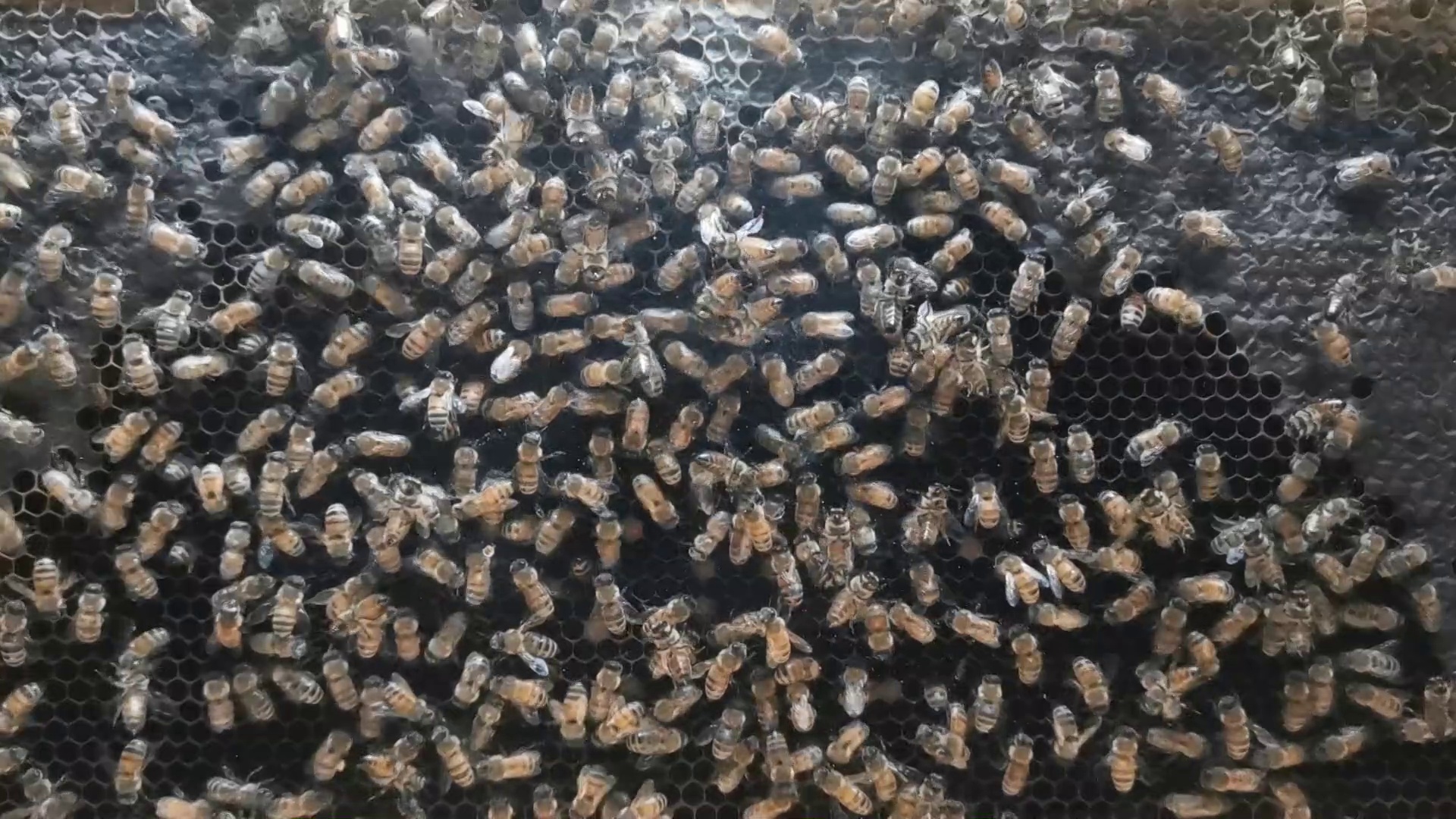

Supplement: Supplementary file 1 — Supplementary Information. [file 41598_2023_44718_MOESM1_ESM.zip › Dataset/test set-system_evaluation/test_set_15fps/148.jpg]

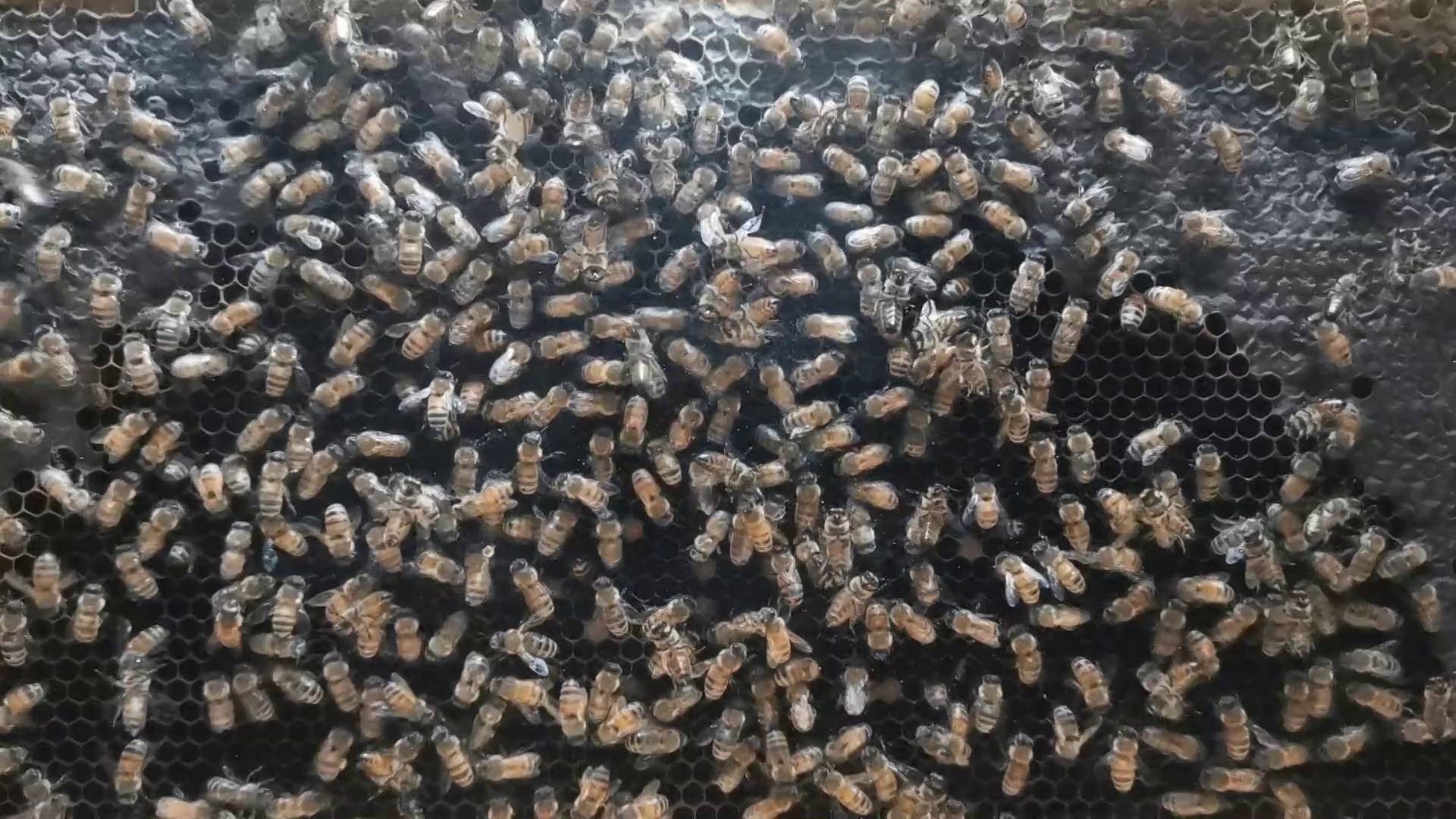

Supplement: Supplementary file 1 — Supplementary Information. [file 41598_2023_44718_MOESM1_ESM.zip › Dataset/test set-system_evaluation/test_set_15fps/147.jpg]

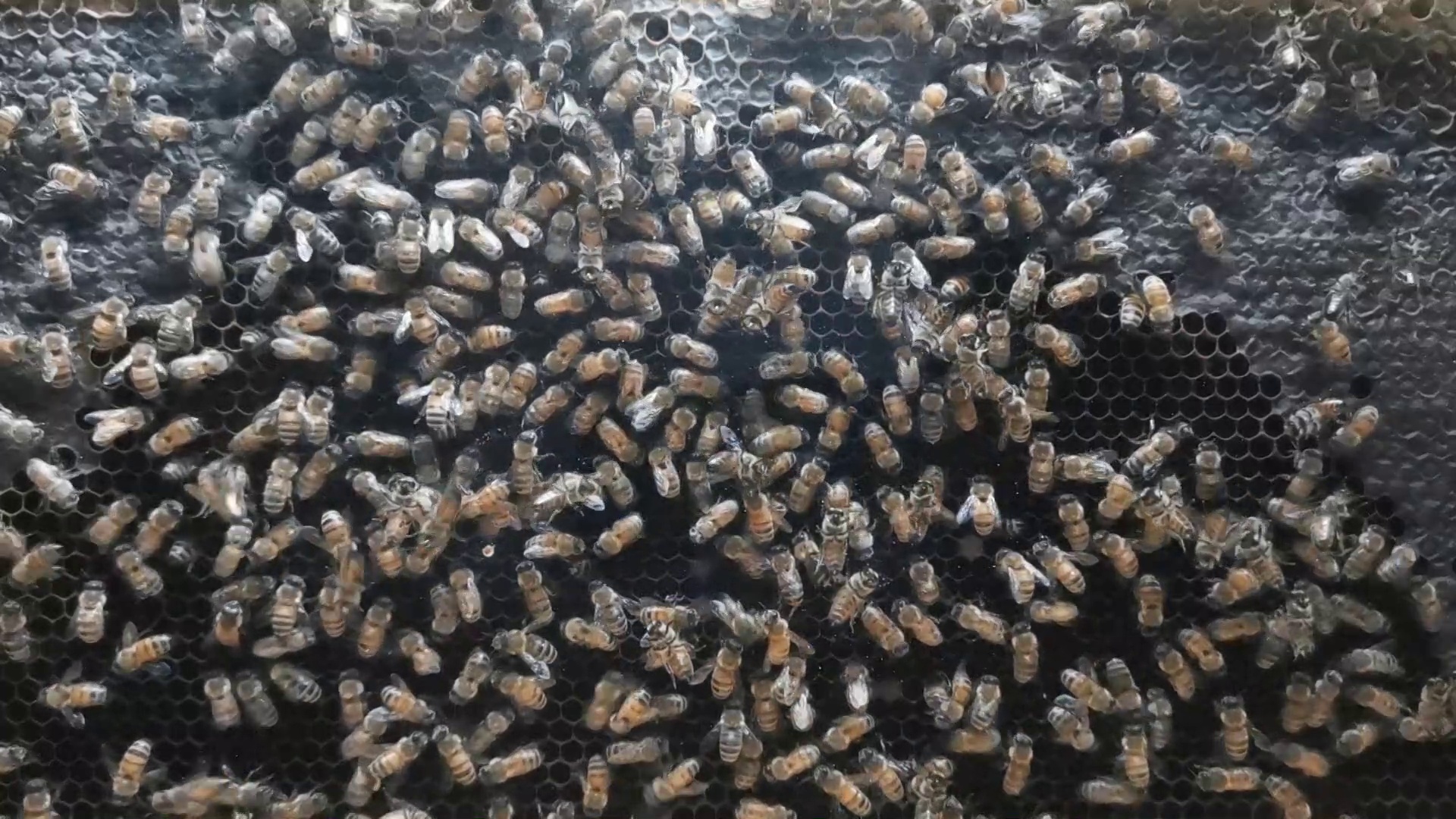

Supplement: Supplementary file 1 — Supplementary Information. [file 41598_2023_44718_MOESM1_ESM.zip › Dataset/test set-system_evaluation/test_set_15fps/127.jpg]

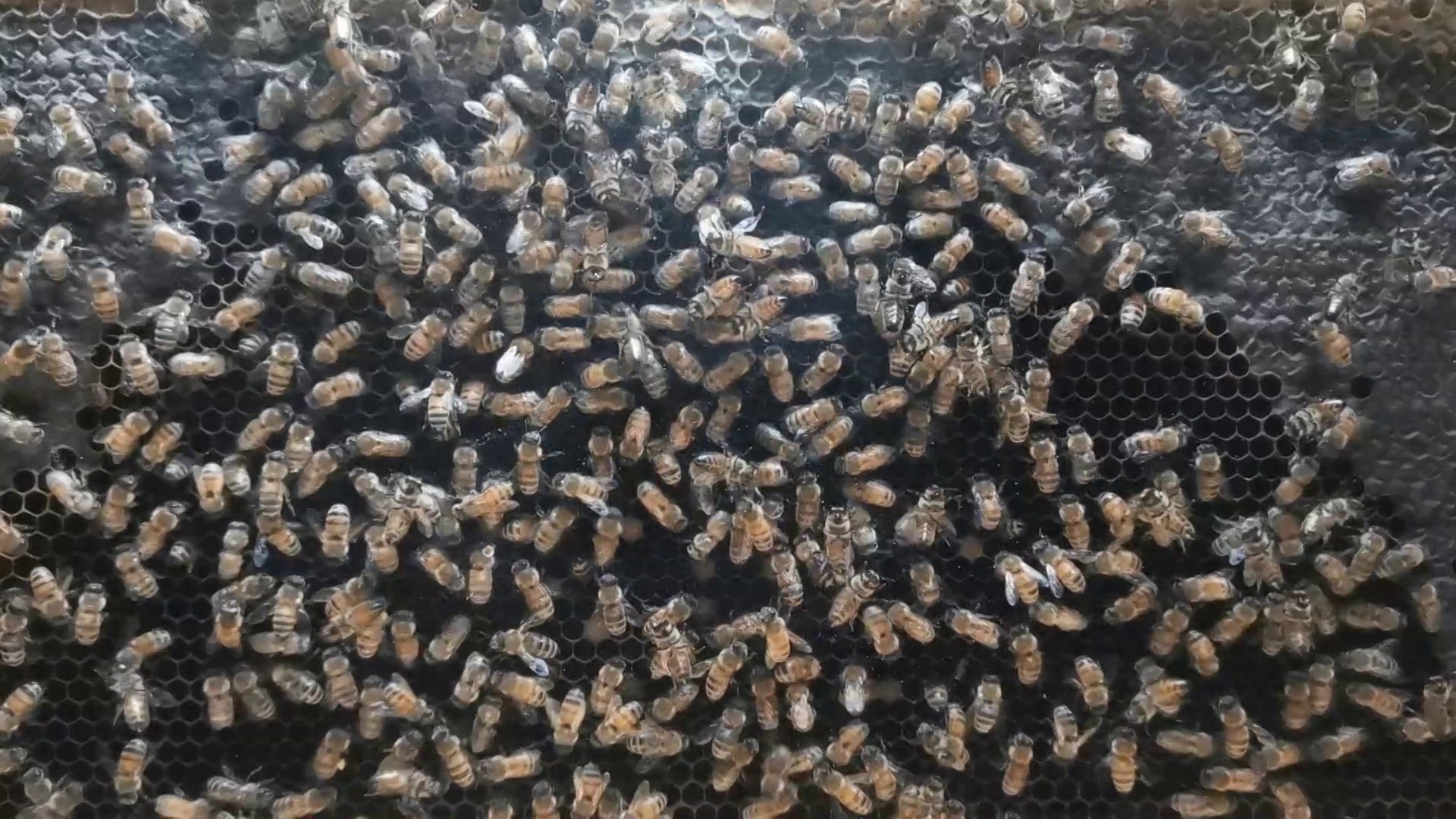

Supplement: Supplementary file 1 — Supplementary Information. [file 41598_2023_44718_MOESM1_ESM.zip › Dataset/test set-system_evaluation/test_set_15fps/150.jpg]

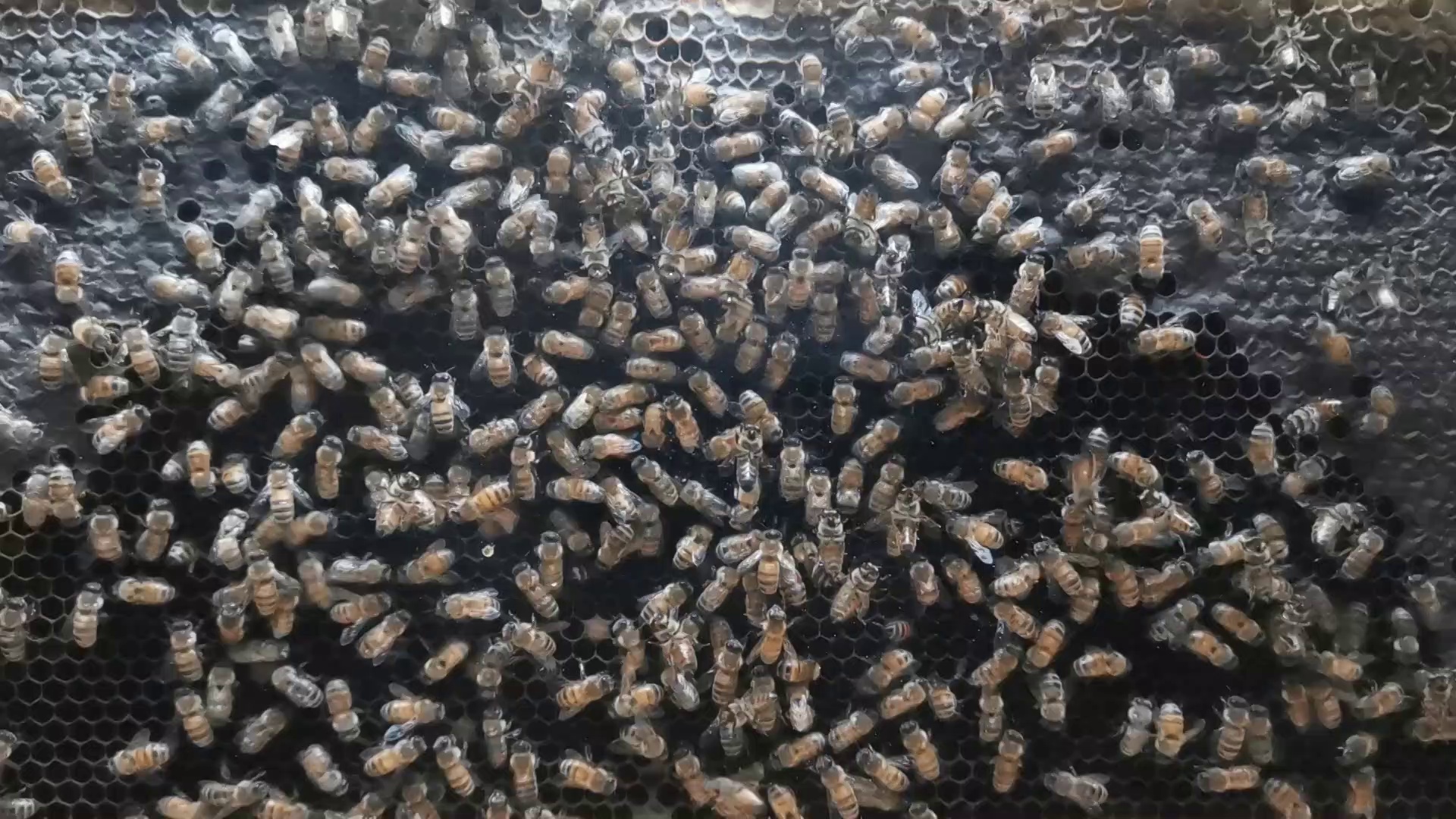

Supplement: Supplementary file 1 — Supplementary Information. [file 41598_2023_44718_MOESM1_ESM.zip › Dataset/dataset-Mask_RCNN_Training/dataset/train/016.jpg]

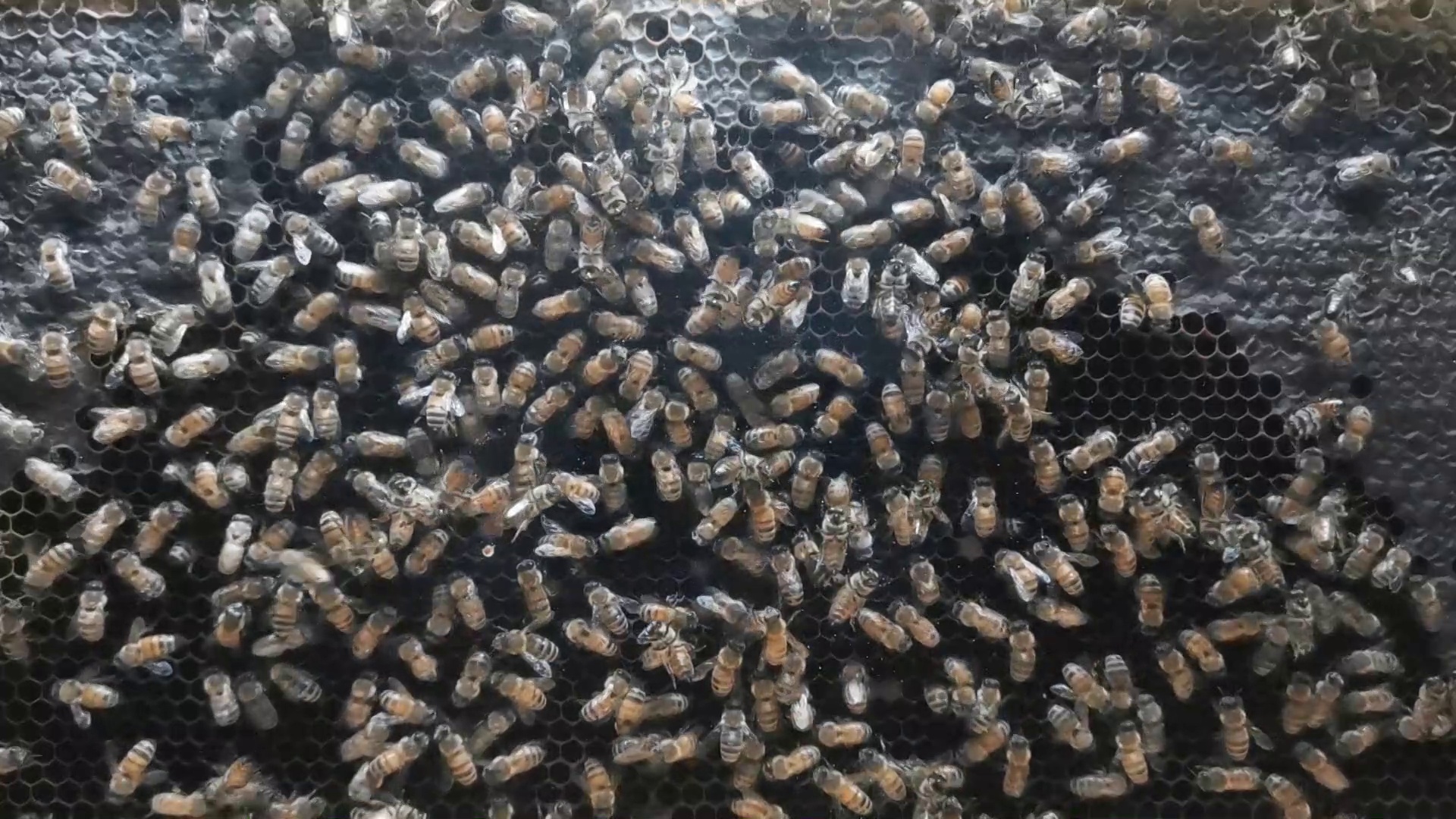

Supplement: Supplementary file 1 — Supplementary Information. [file 41598_2023_44718_MOESM1_ESM.zip › Dataset/test set-system_evaluation/test_set_15fps/123.jpg]

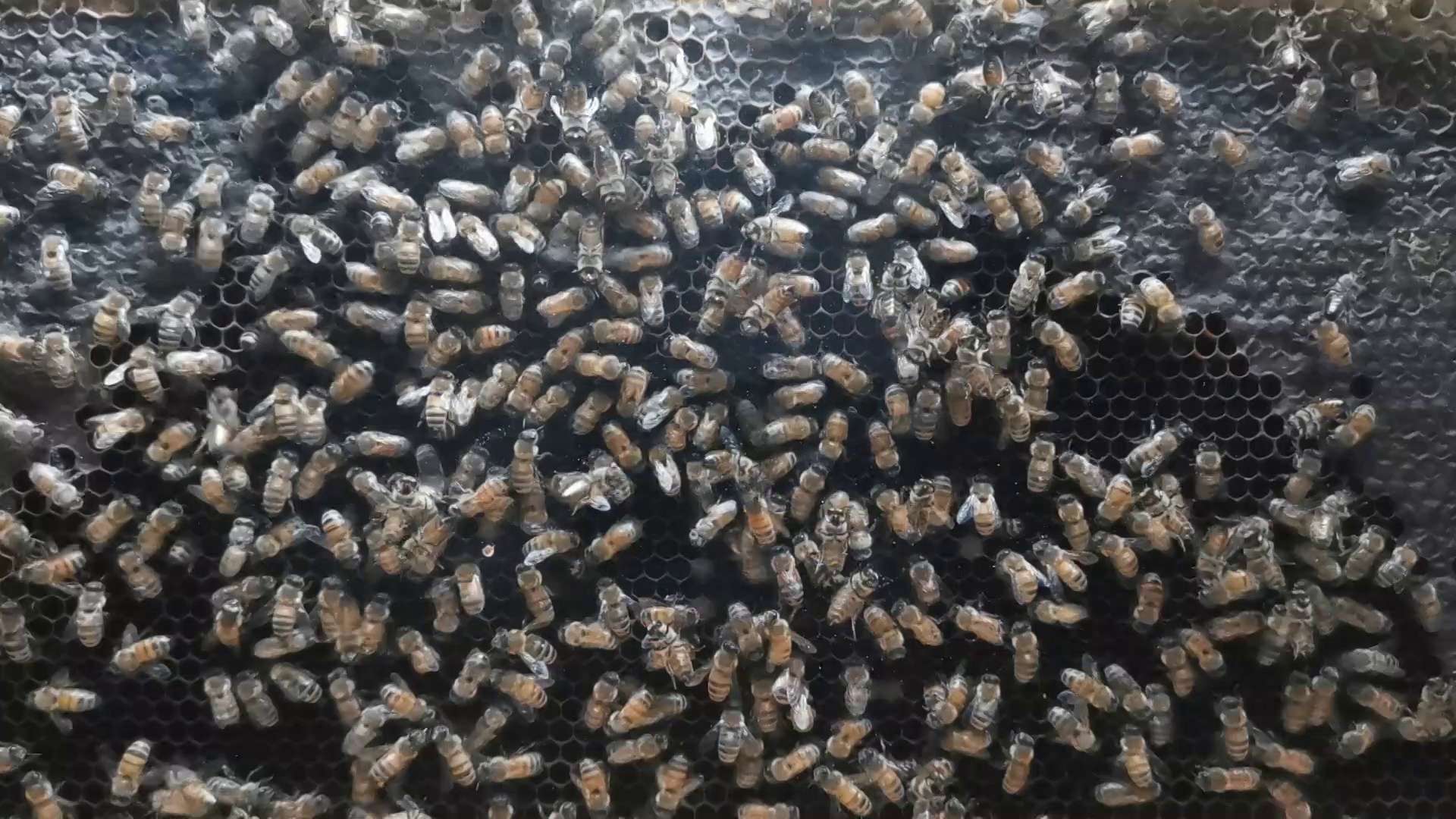

Supplement: Supplementary file 1 — Supplementary Information. [file 41598_2023_44718_MOESM1_ESM.zip › Dataset/test set-system_evaluation/test_set_15fps/130.jpg]

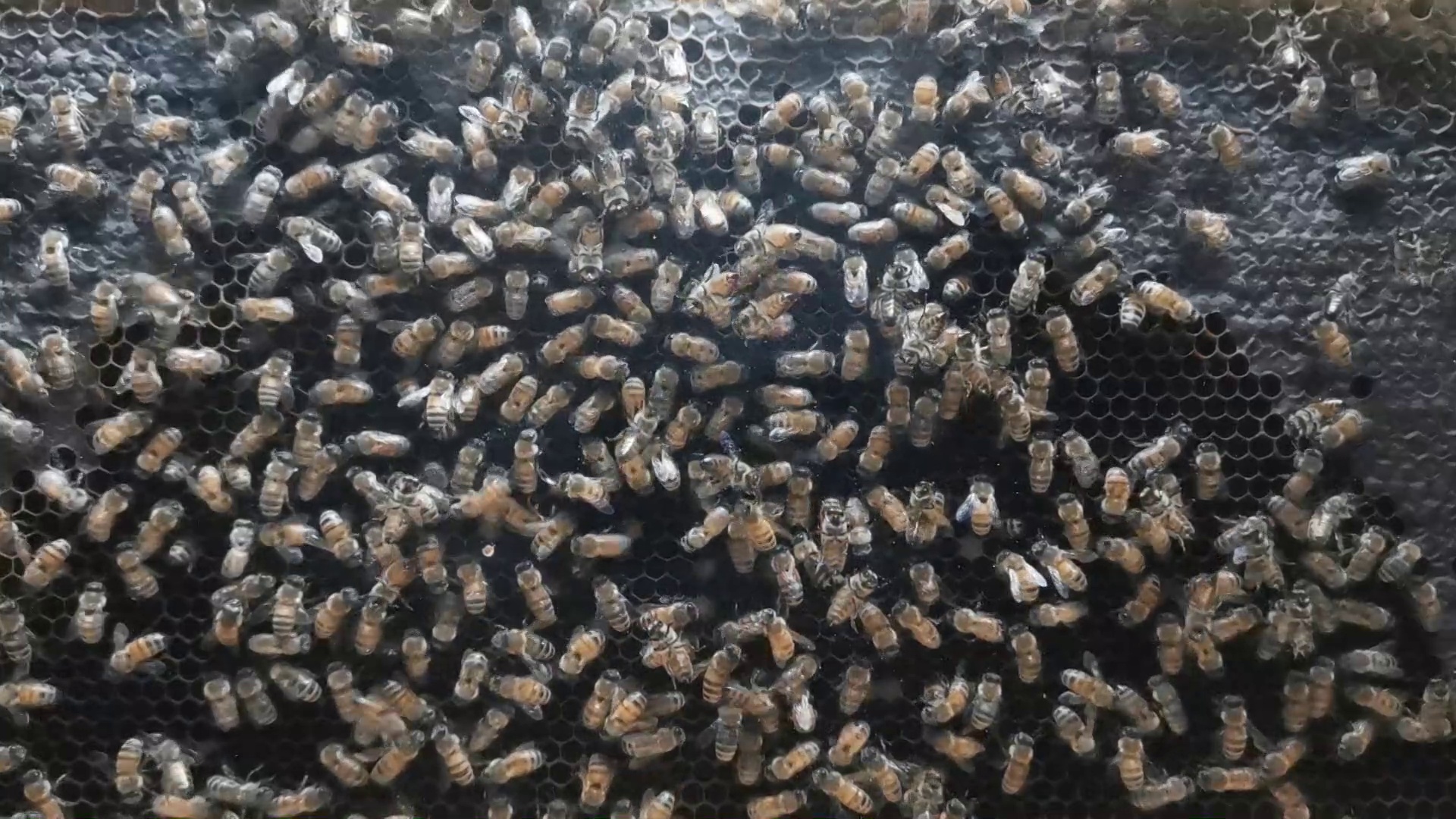

Supplement: Supplementary file 1 — Supplementary Information. [file 41598_2023_44718_MOESM1_ESM.zip › Dataset/test set-system_evaluation/test_set_15fps/137.jpg]

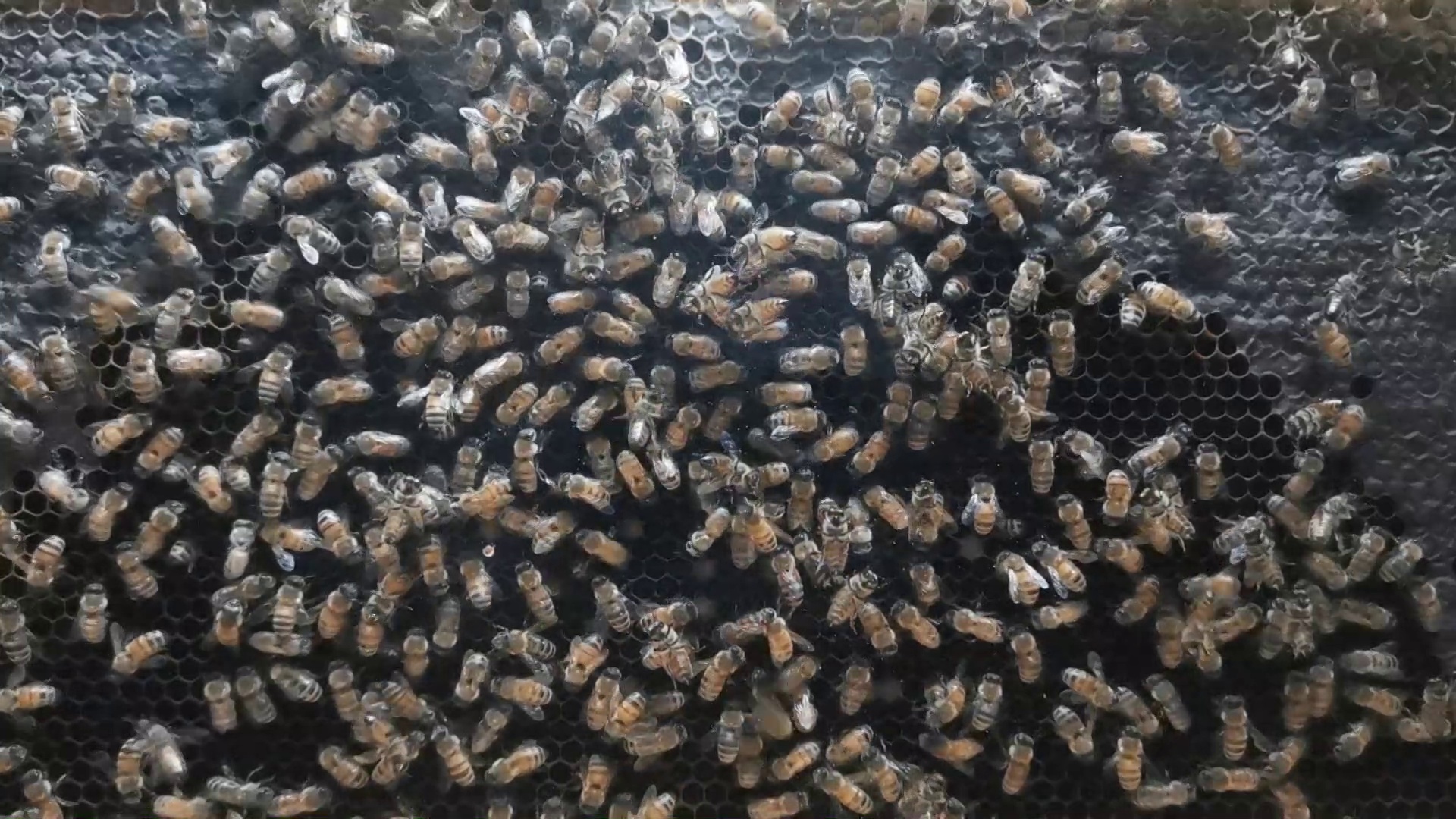

Supplement: Supplementary file 1 — Supplementary Information. [file 41598_2023_44718_MOESM1_ESM.zip › Dataset/test set-system_evaluation/test_set_15fps/139.jpg]

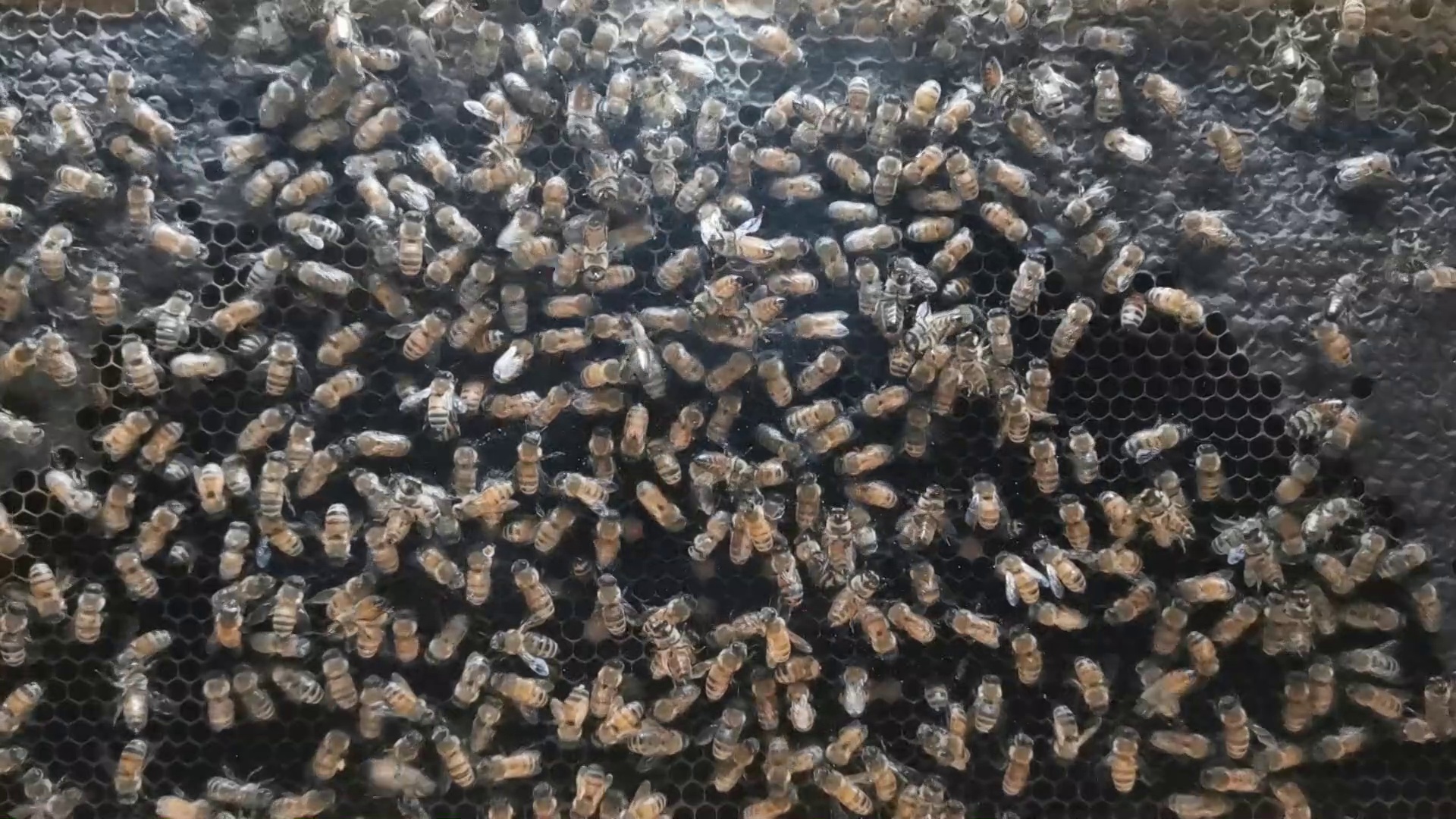

Supplement: Supplementary file 1 — Supplementary Information. [file 41598_2023_44718_MOESM1_ESM.zip › Dataset/test set-system_evaluation/test_set_15fps/149.jpg]

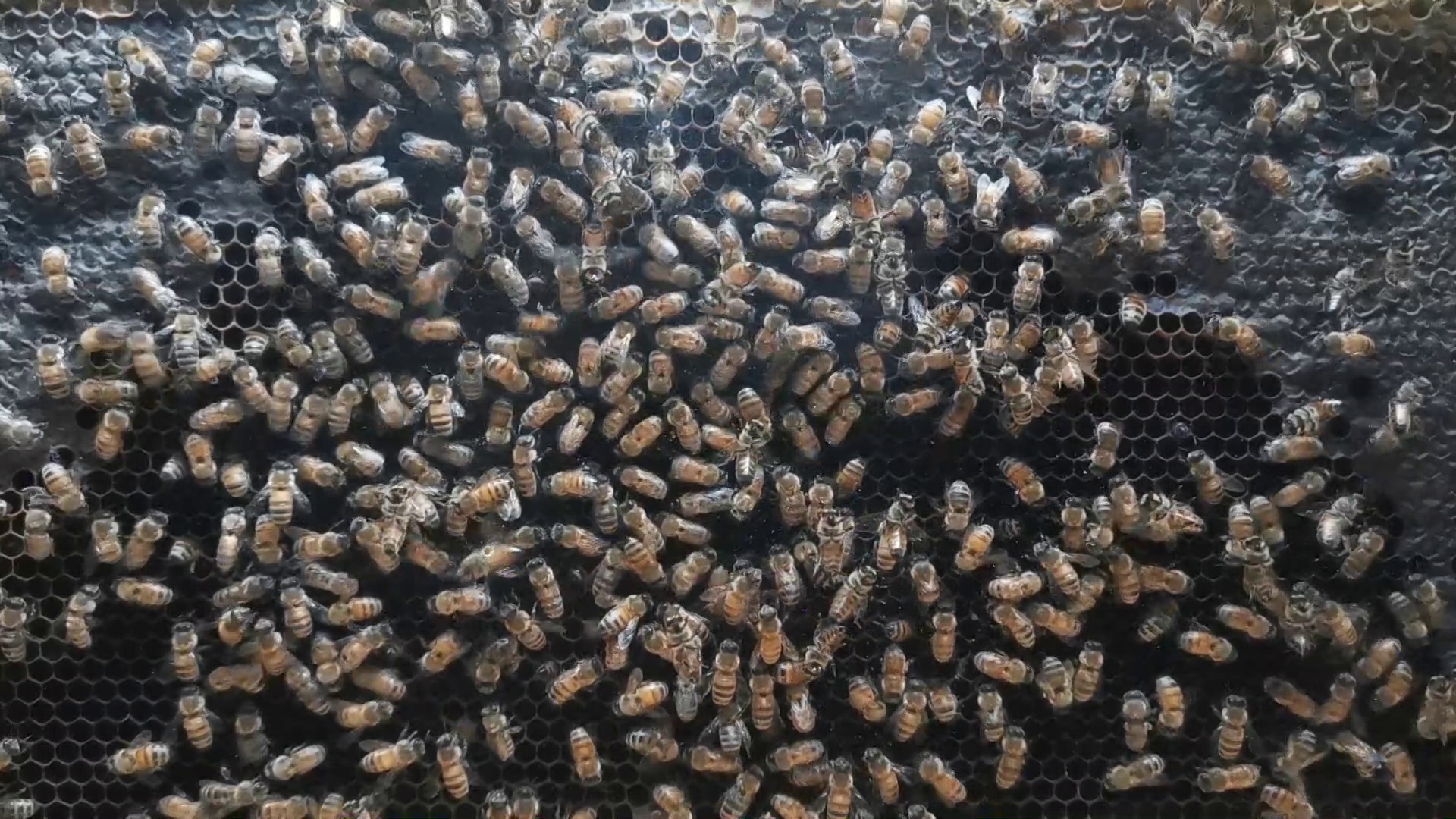

Supplement: Supplementary file 1 — Supplementary Information. [file 41598_2023_44718_MOESM1_ESM.zip › Dataset/dataset-Mask_RCNN_Training/dataset/train/004.jpg]

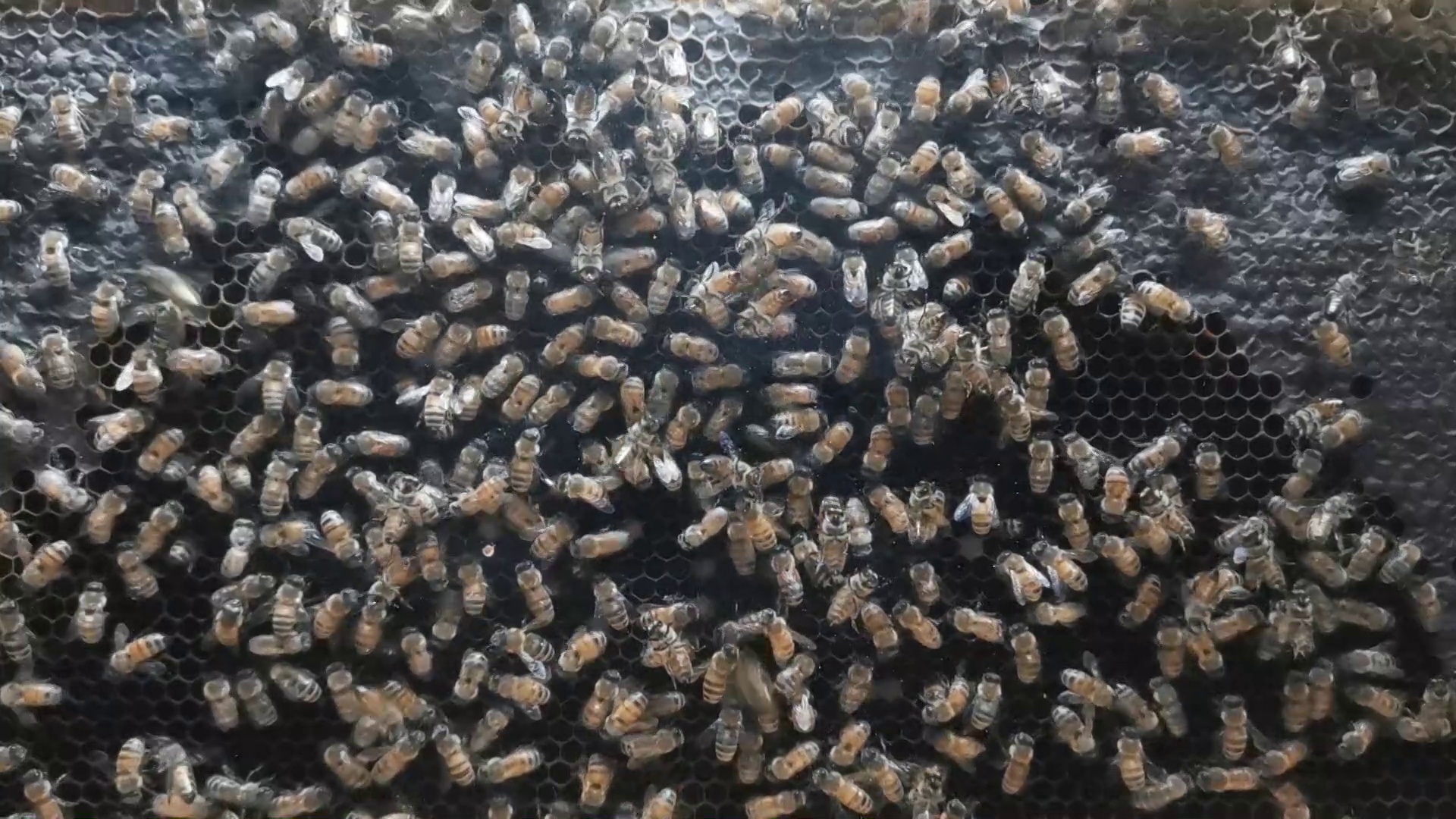

Supplement: Supplementary file 1 — Supplementary Information. [file 41598_2023_44718_MOESM1_ESM.zip › Dataset/test set-system_evaluation/test_set_15fps/136.jpg]

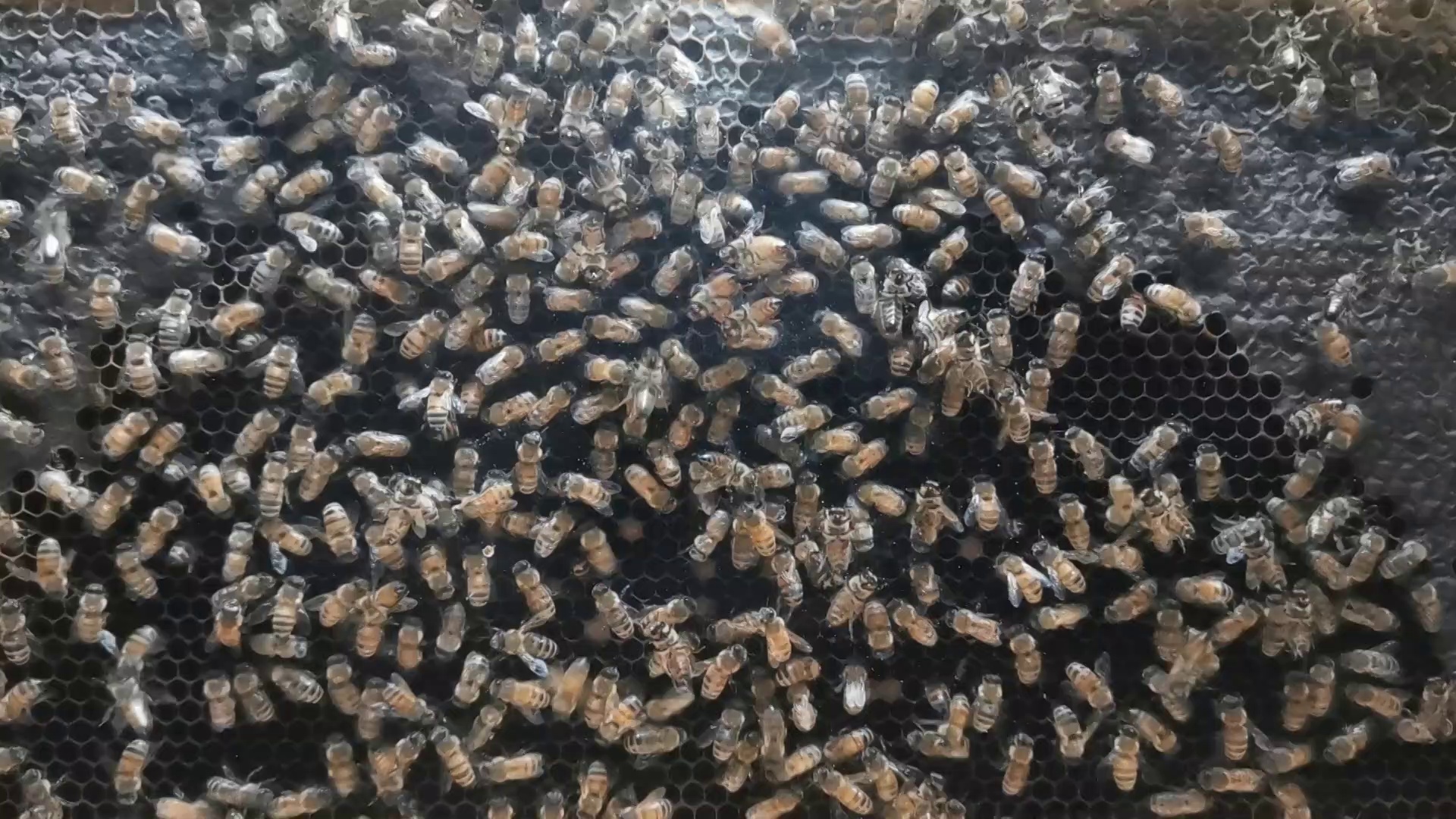

Supplement: Supplementary file 1 — Supplementary Information. [file 41598_2023_44718_MOESM1_ESM.zip › Dataset/test set-system_evaluation/test_set_15fps/144.jpg]

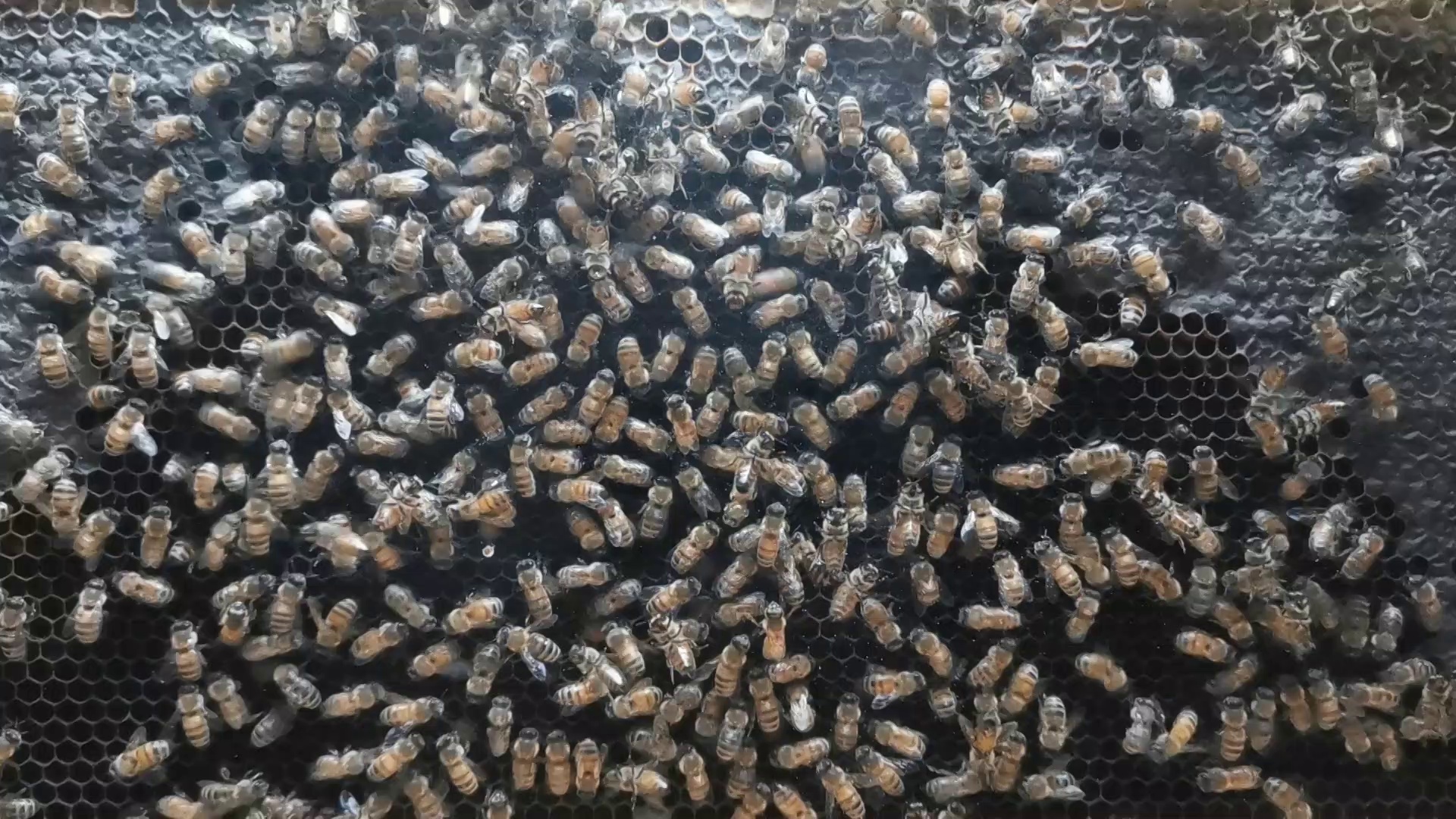

Supplement: Supplementary file 1 — Supplementary Information. [file 41598_2023_44718_MOESM1_ESM.zip › Dataset/dataset-Mask_RCNN_Training/dataset/train/026.jpg]

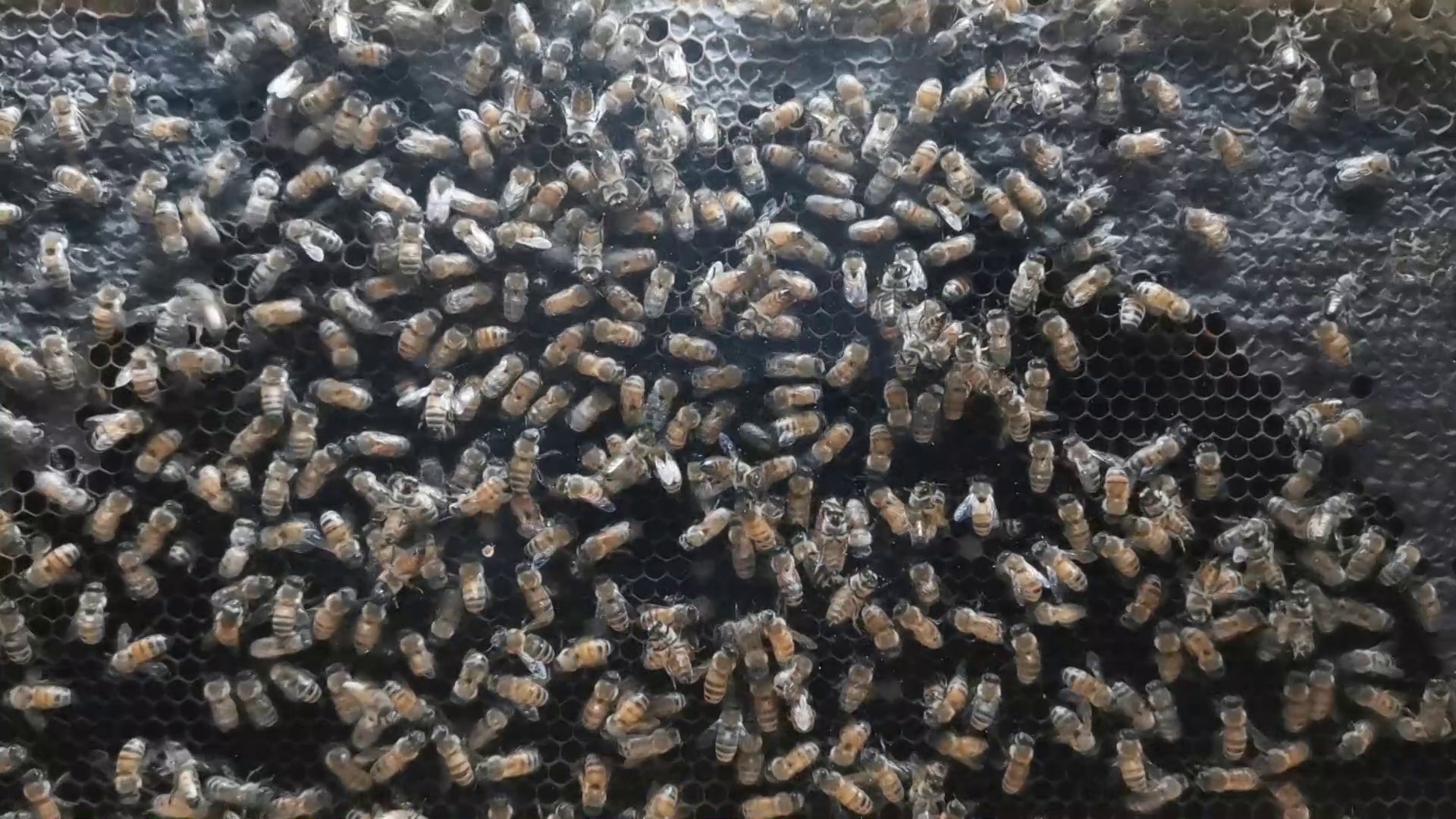

Supplement: Supplementary file 1 — Supplementary Information. [file 41598_2023_44718_MOESM1_ESM.zip › Dataset/test set-system_evaluation/test_set_15fps/135.jpg]

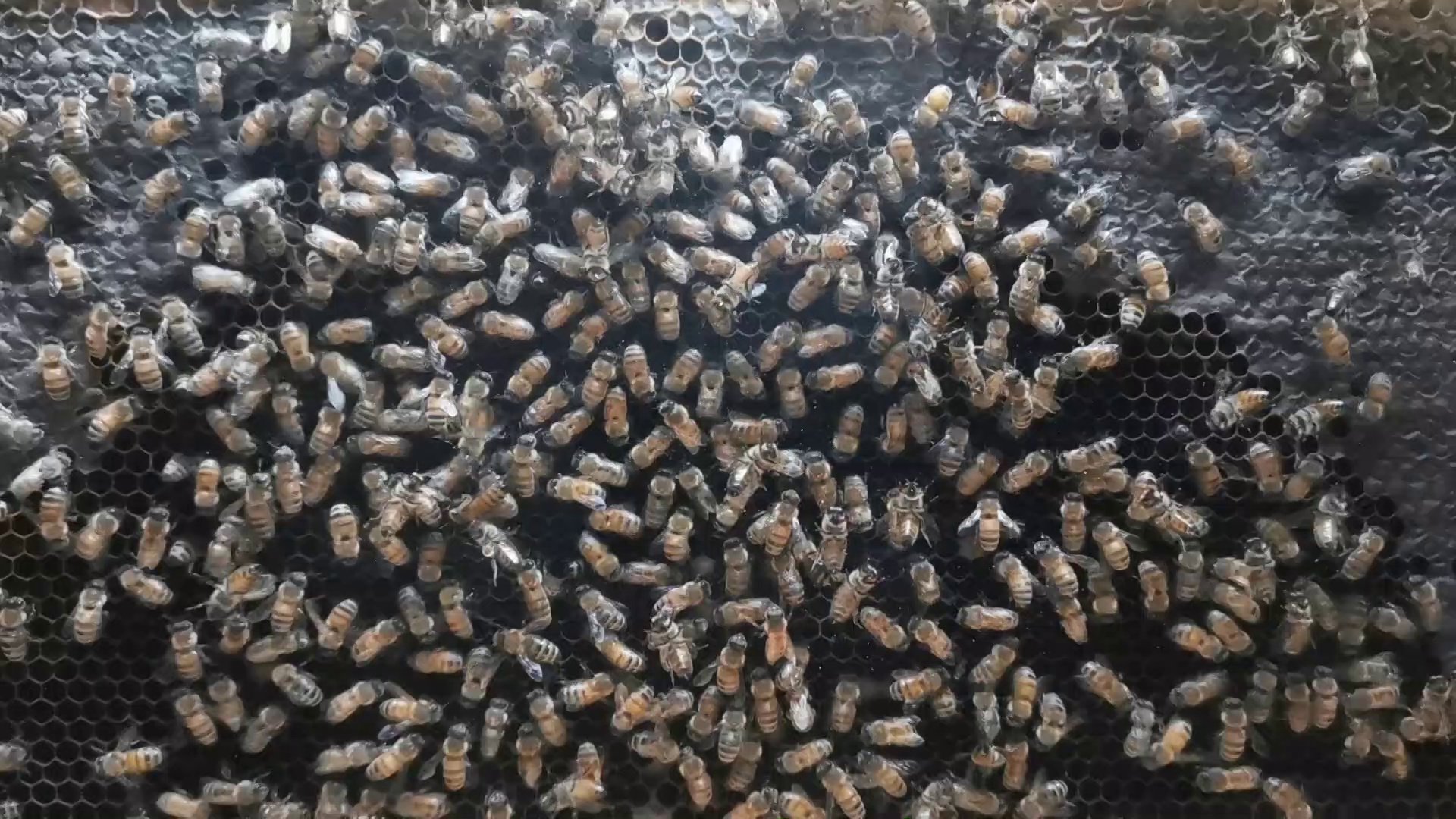

Supplement: Supplementary file 1 — Supplementary Information. [file 41598_2023_44718_MOESM1_ESM.zip › Dataset/test set-system_evaluation/test_set_15fps/087.jpg]

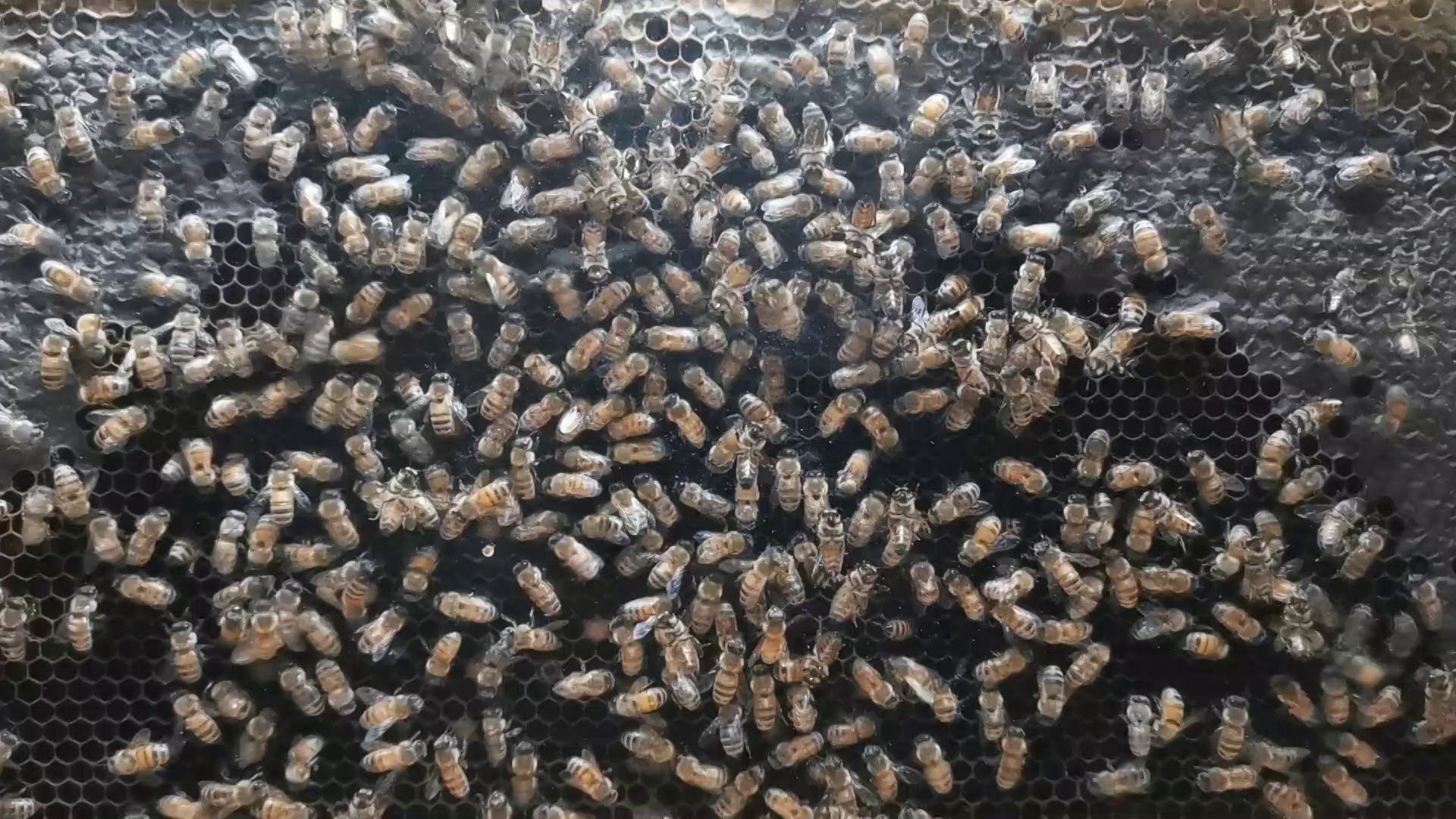

Supplement: Supplementary file 1 — Supplementary Information. [file 41598_2023_44718_MOESM1_ESM.zip › Dataset/dataset-Mask_RCNN_Training/dataset/train/011.jpg]

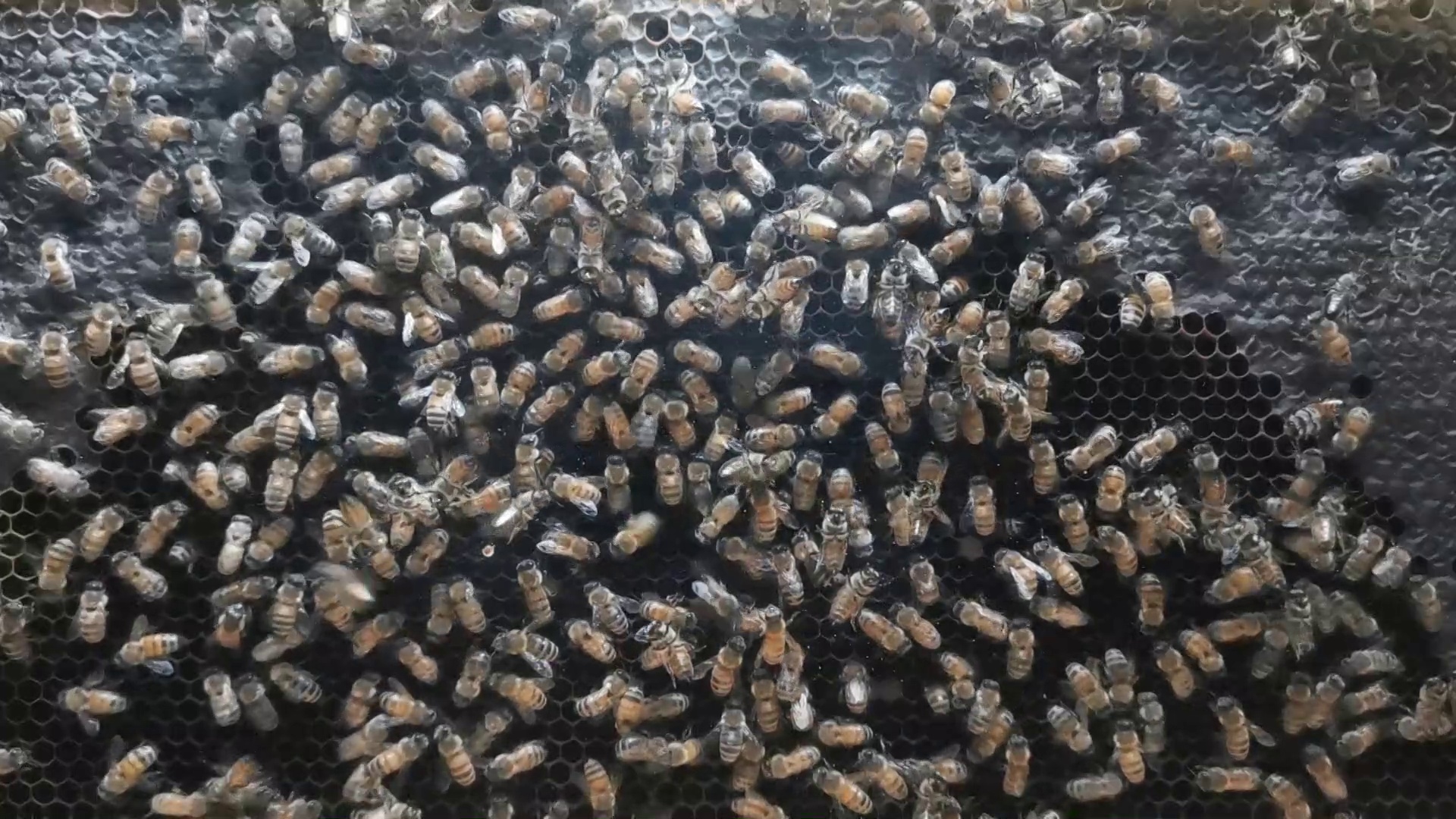

Supplement: Supplementary file 1 — Supplementary Information. [file 41598_2023_44718_MOESM1_ESM.zip › Dataset/test set-system_evaluation/test_set_15fps/121.jpg]

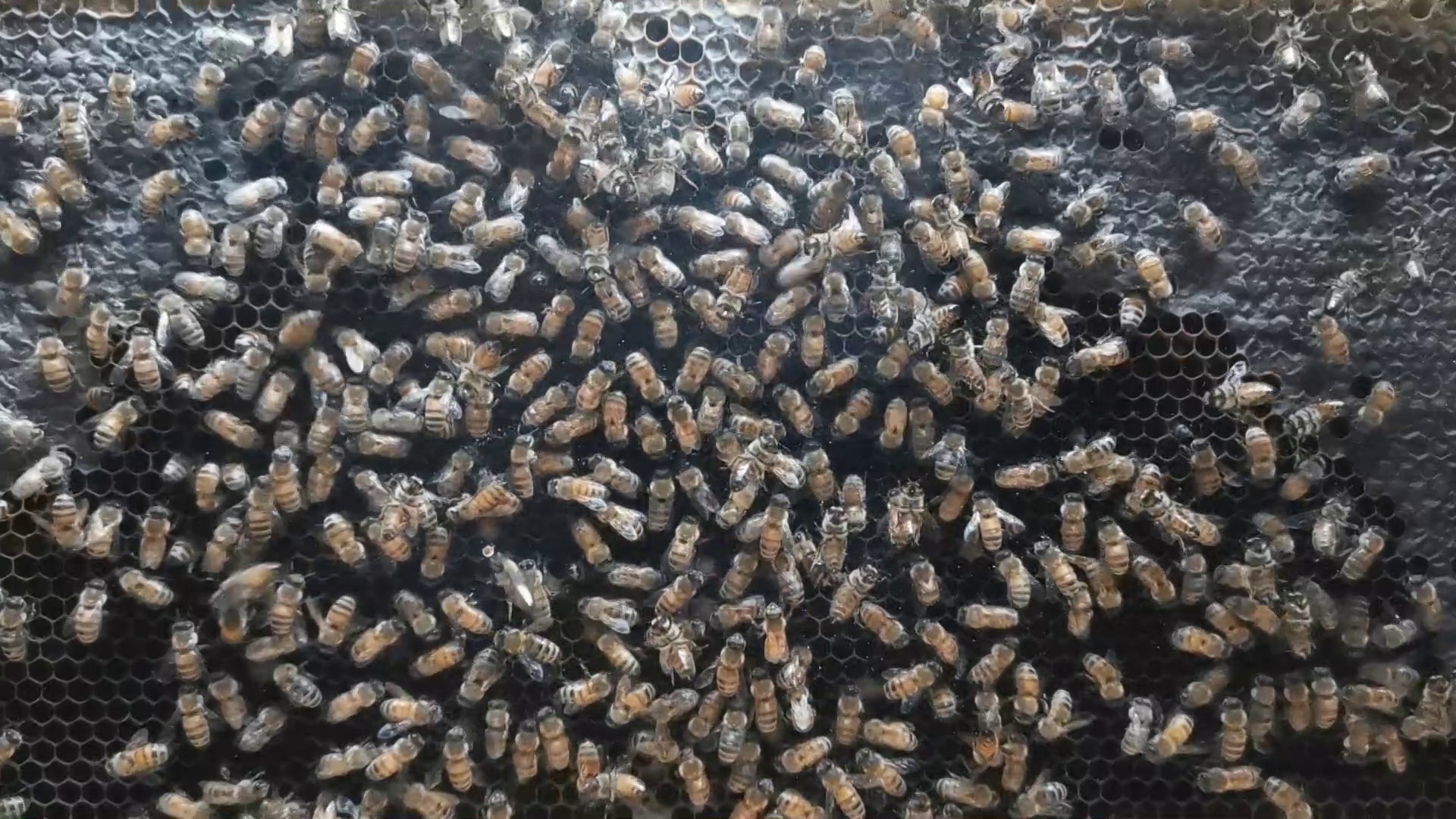

Supplement: Supplementary file 1 — Supplementary Information. [file 41598_2023_44718_MOESM1_ESM.zip › Dataset/dataset-Mask_RCNN_Training/dataset/train/029.jpg]

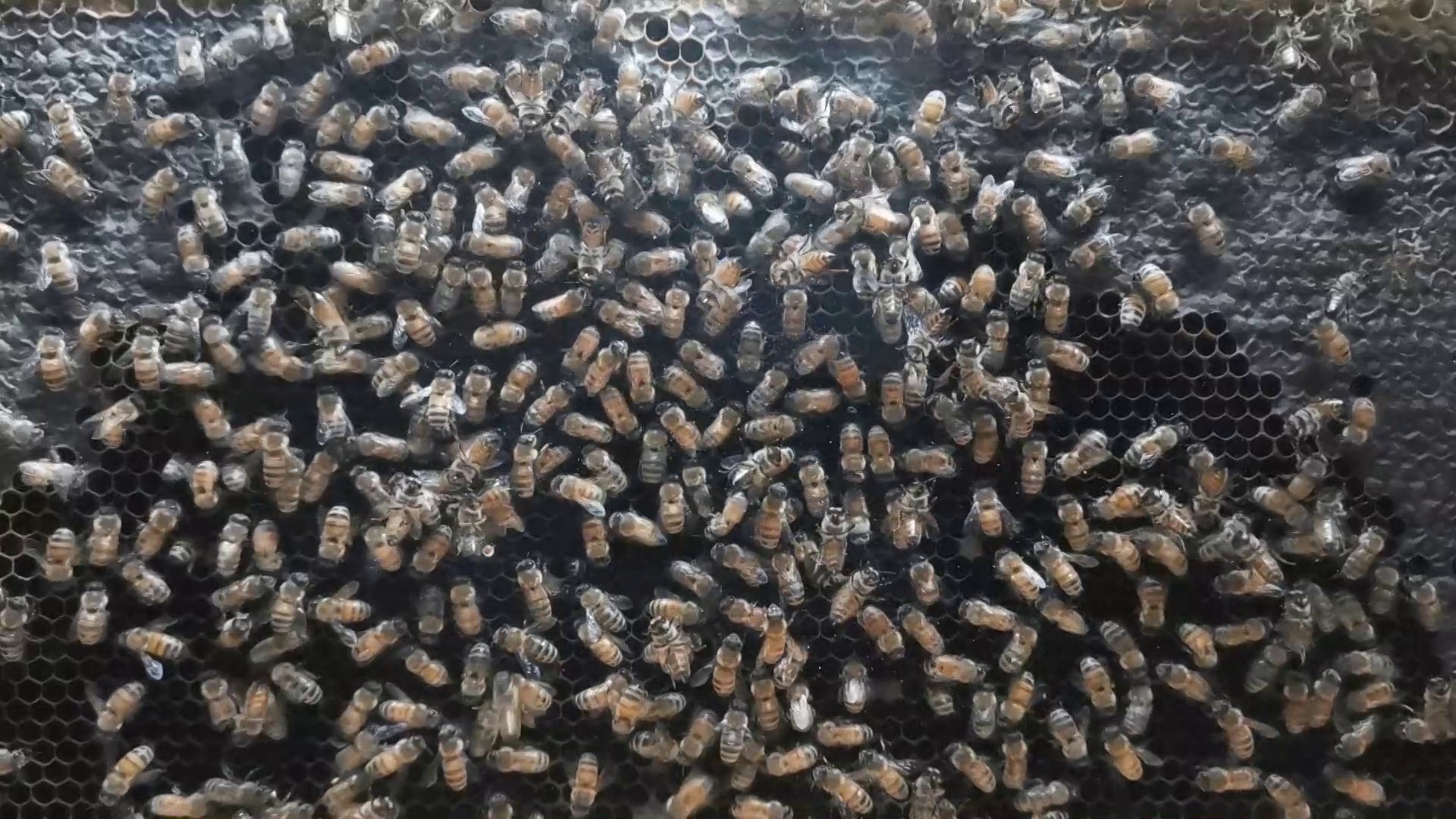

Supplement: Supplementary file 1 — Supplementary Information. [file 41598_2023_44718_MOESM1_ESM.zip › Dataset/test set-system_evaluation/test_set_15fps/109.jpg]

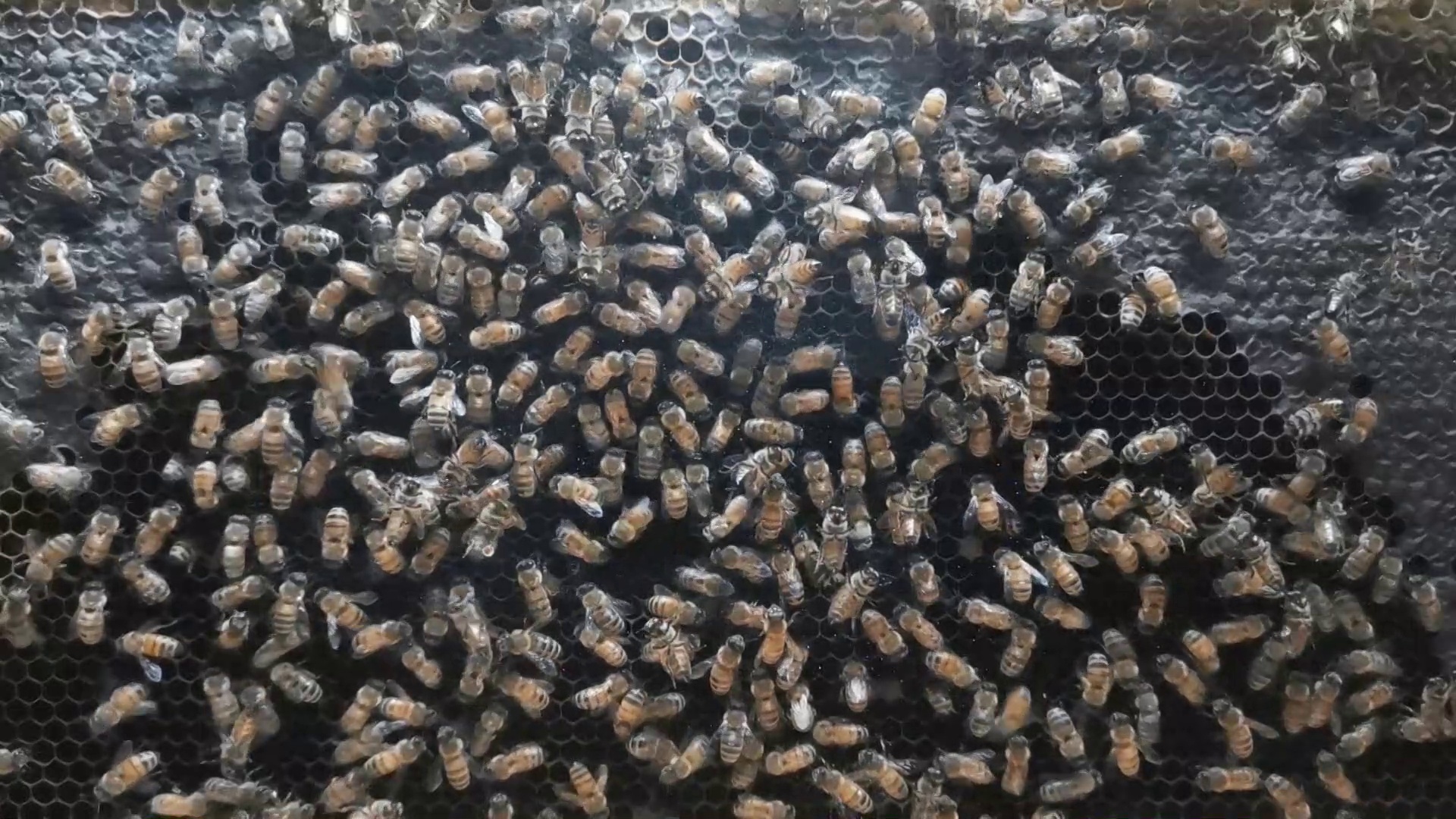

Supplement: Supplementary file 1 — Supplementary Information. [file 41598_2023_44718_MOESM1_ESM.zip › Dataset/test set-system_evaluation/test_set_15fps/114.jpg]

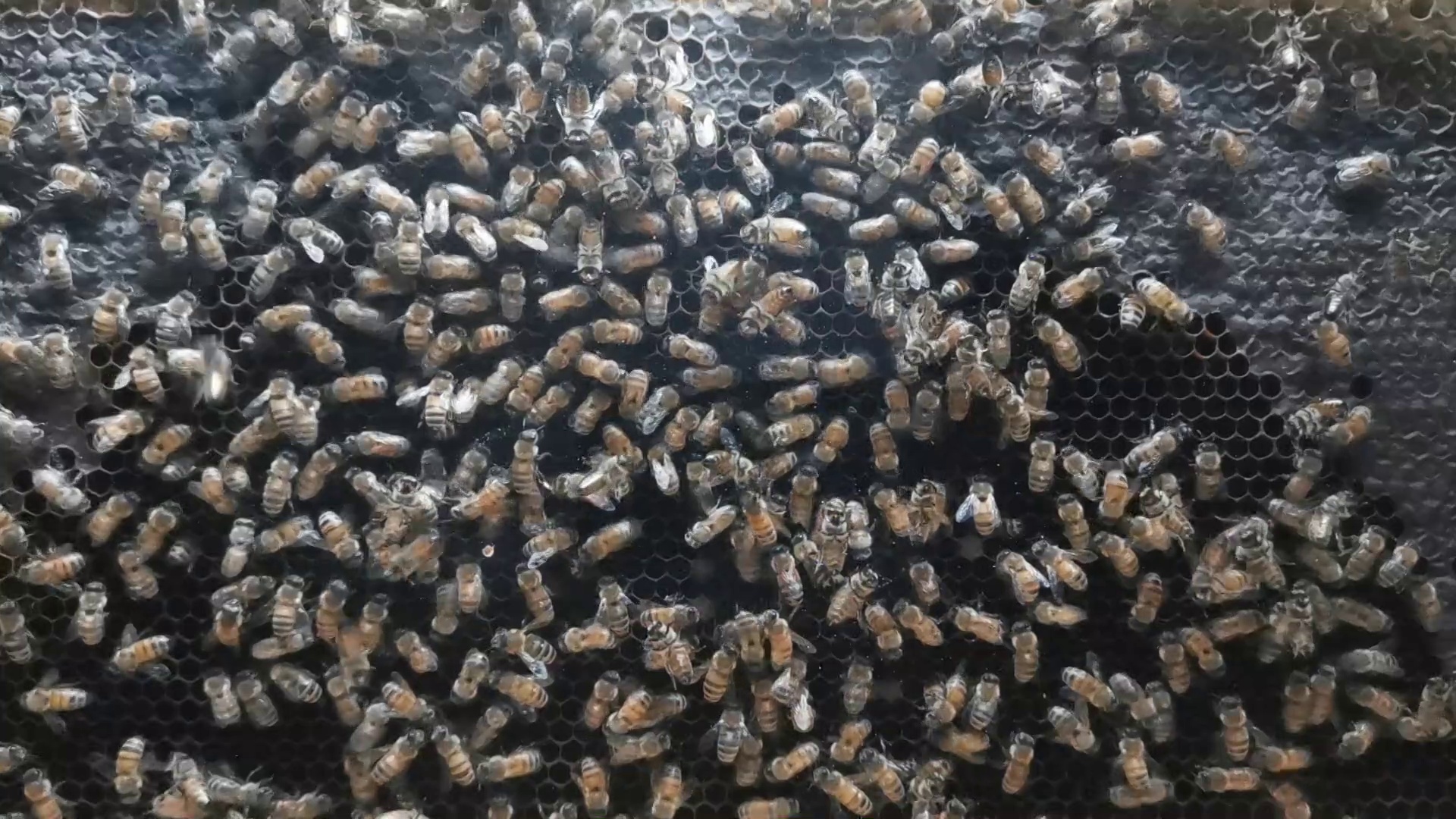

Supplement: Supplementary file 1 — Supplementary Information. [file 41598_2023_44718_MOESM1_ESM.zip › Dataset/test set-system_evaluation/test_set_15fps/132.jpg]

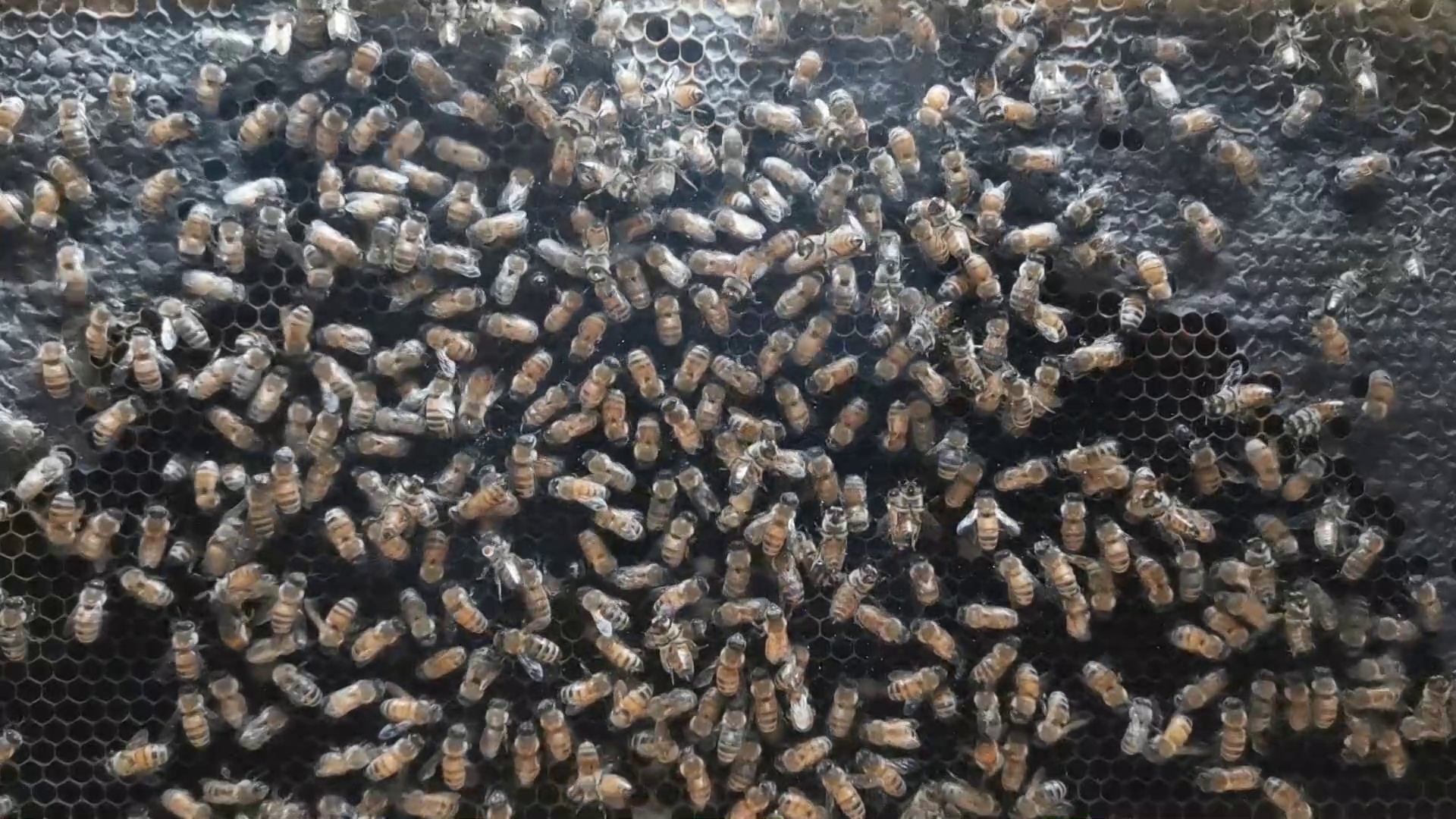

Supplement: Supplementary file 1 — Supplementary Information. [file 41598_2023_44718_MOESM1_ESM.zip › Dataset/dataset-Mask_RCNN_Training/dataset/train/030.jpg]

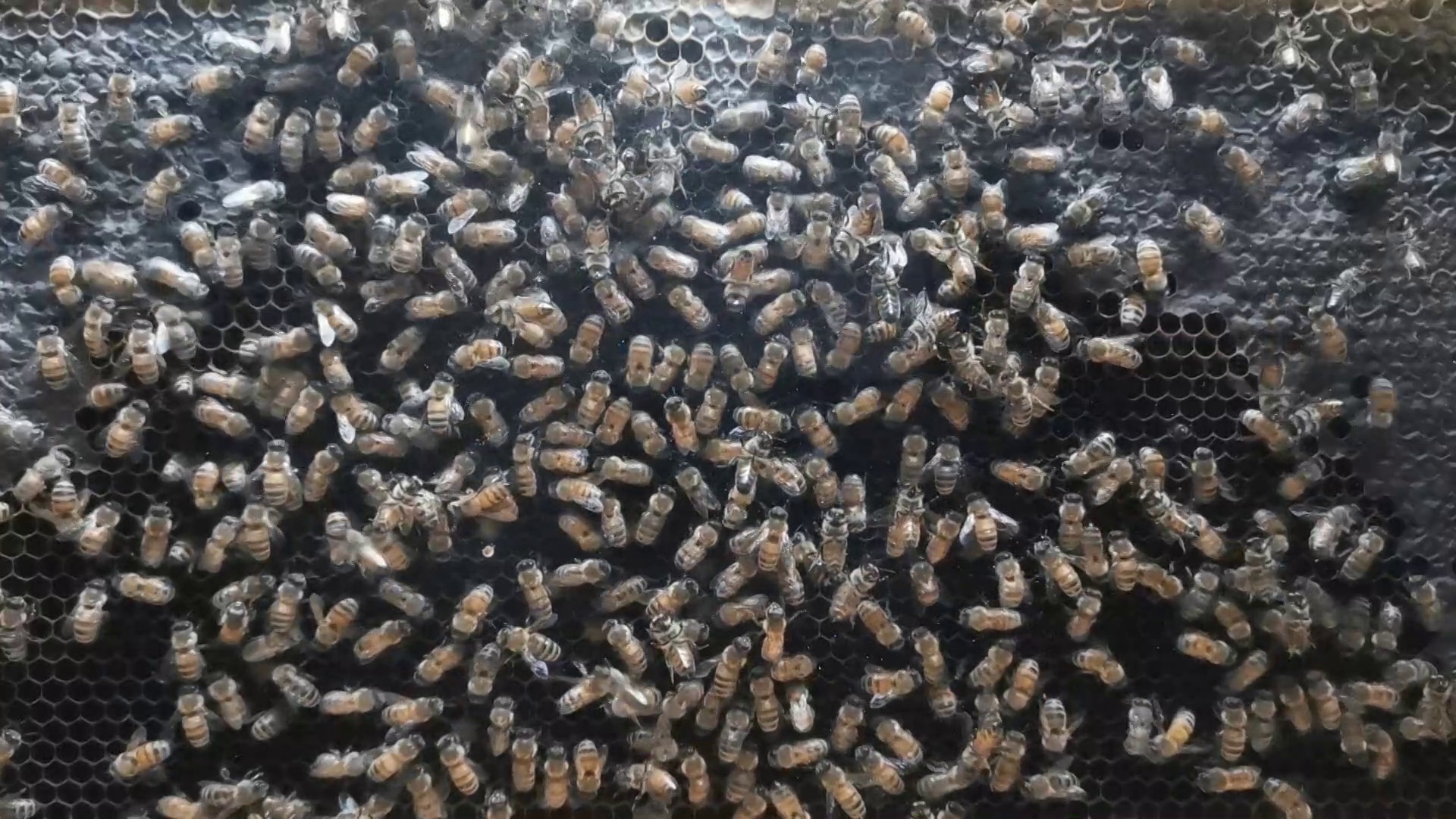

Supplement: Supplementary file 1 — Supplementary Information. [file 41598_2023_44718_MOESM1_ESM.zip › Dataset/dataset-Mask_RCNN_Training/dataset/train/025.jpg]

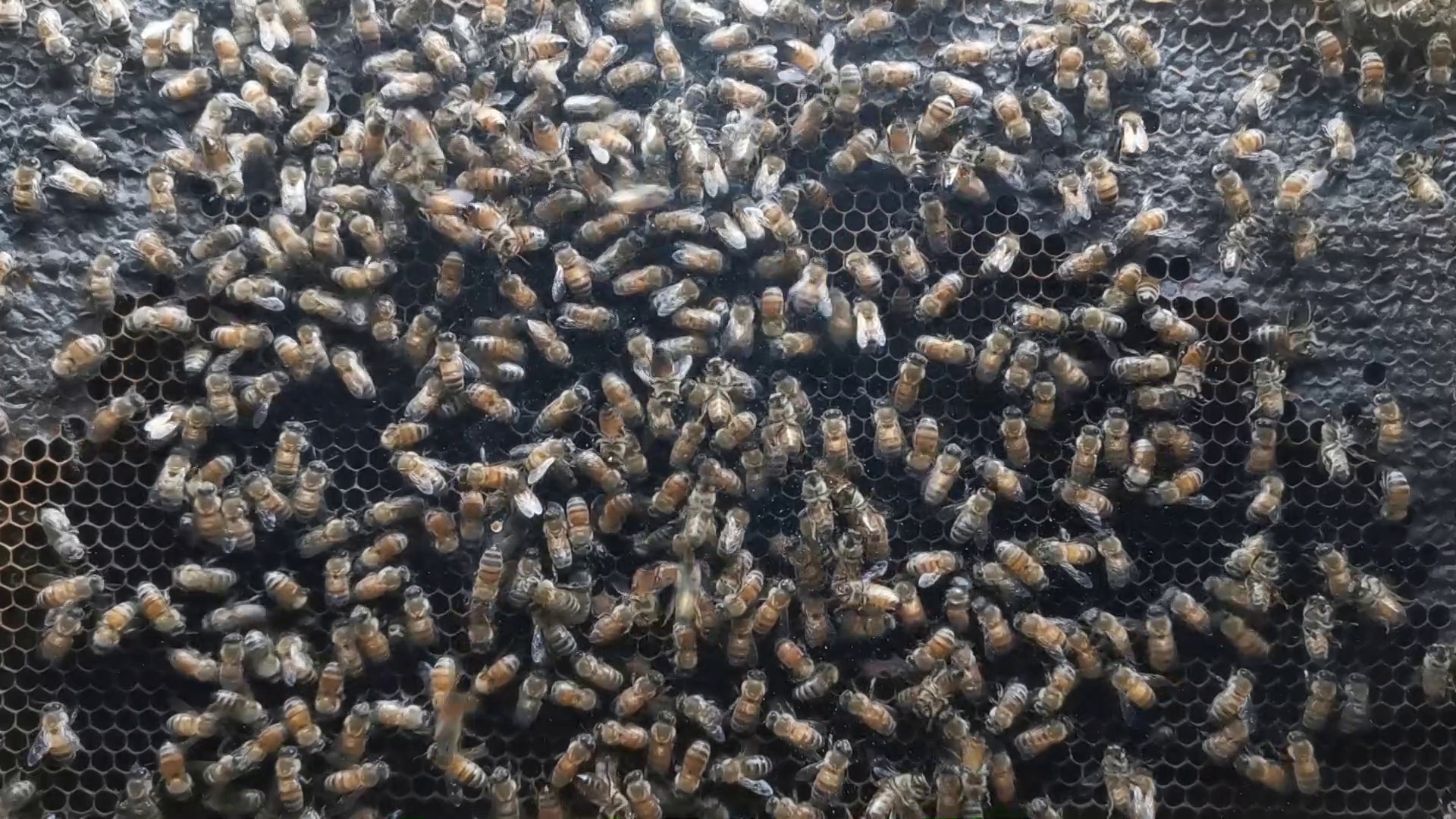

Supplement: Supplementary file 1 — Supplementary Information. [file 41598_2023_44718_MOESM1_ESM.zip › Dataset/dataset-Mask_RCNN_Training/dataset/test/003.jpg]

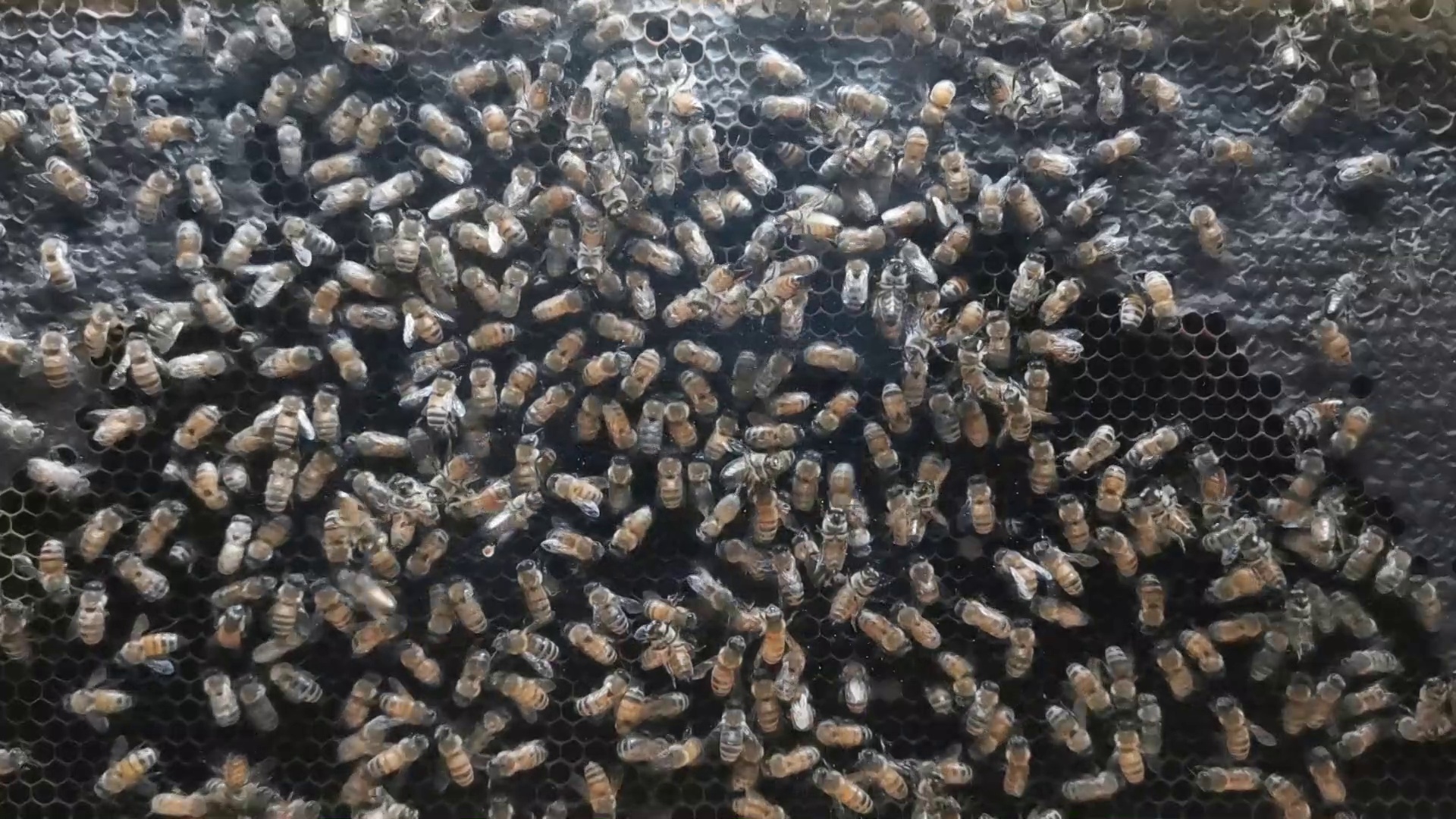

Supplement: Supplementary file 1 — Supplementary Information. [file 41598_2023_44718_MOESM1_ESM.zip › Dataset/test set-system_evaluation/test_set_15fps/120.jpg]

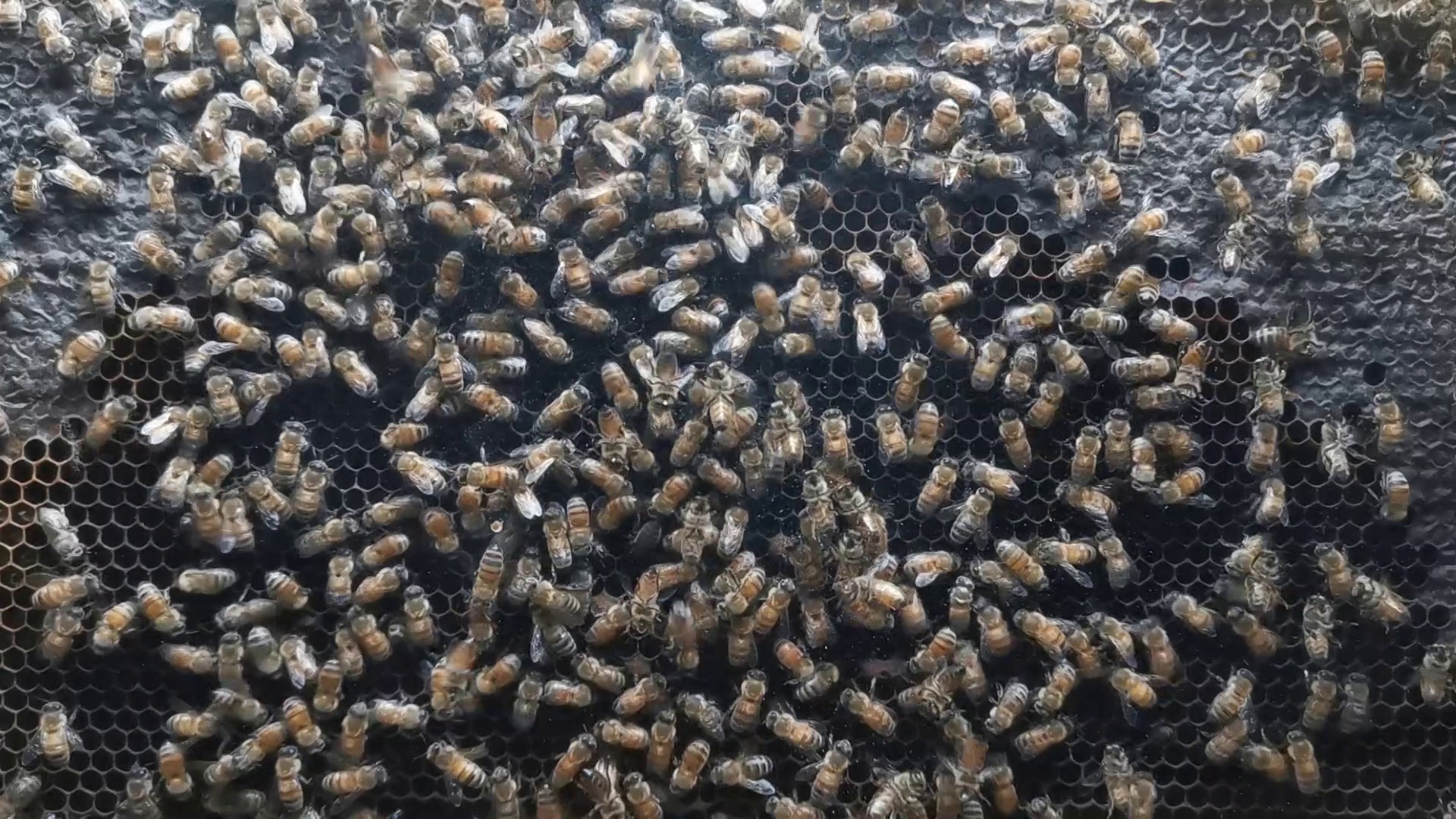

Supplement: Supplementary file 1 — Supplementary Information. [file 41598_2023_44718_MOESM1_ESM.zip › Dataset/dataset-Mask_RCNN_Training/dataset/test/002.jpg]

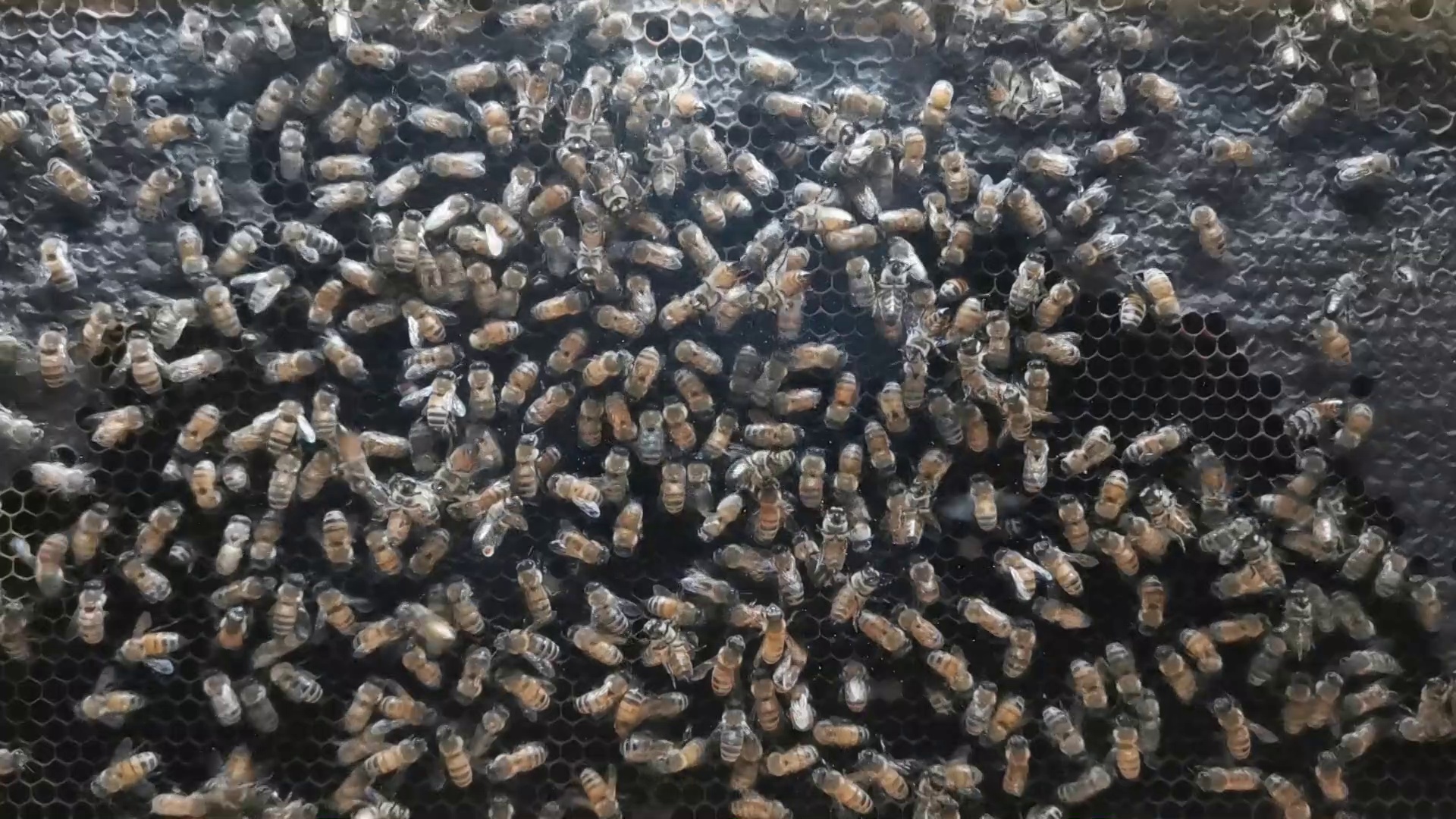

Supplement: Supplementary file 1 — Supplementary Information. [file 41598_2023_44718_MOESM1_ESM.zip › Dataset/test set-system_evaluation/test_set_15fps/117.jpg]

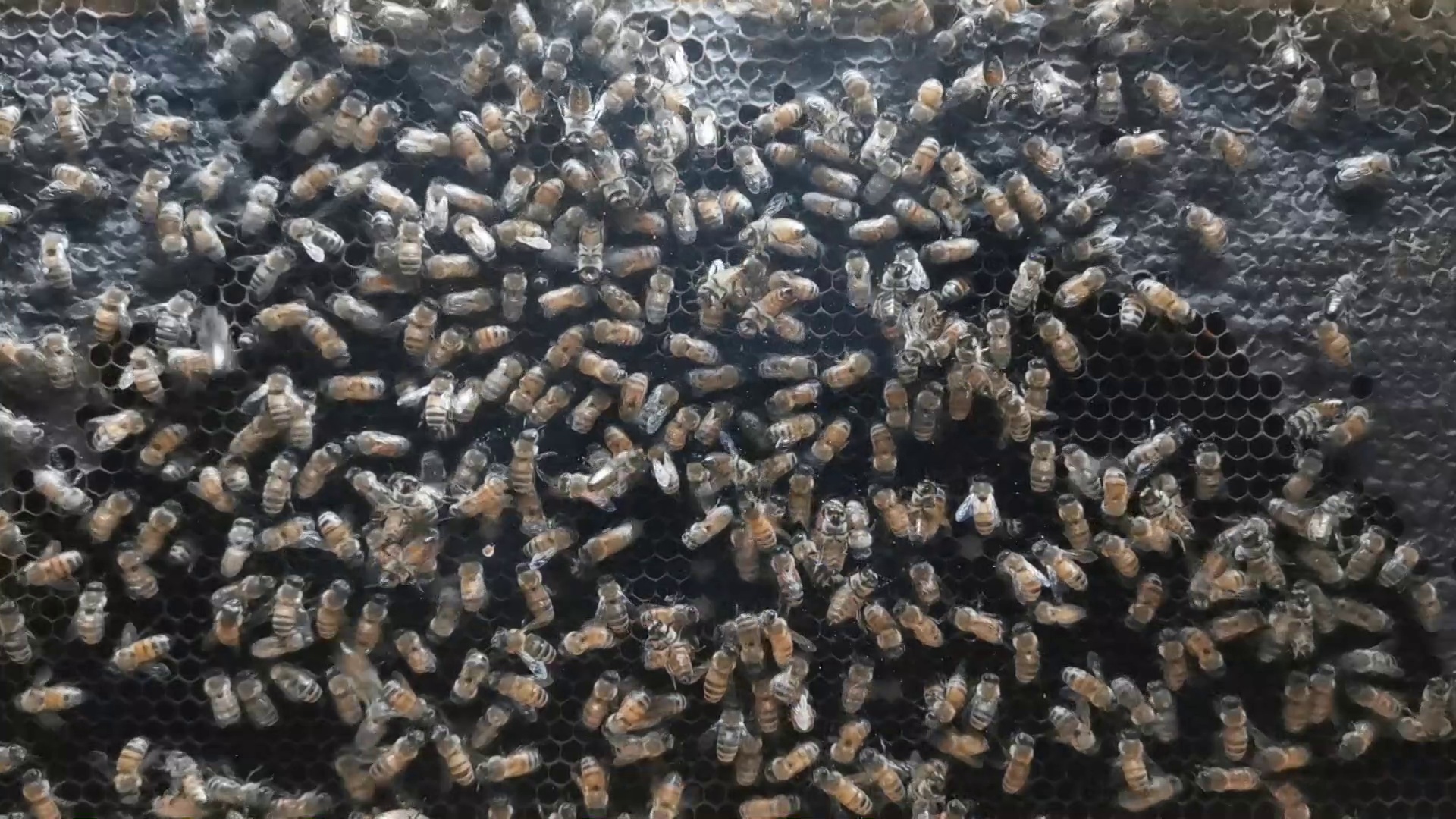

Supplement: Supplementary file 1 — Supplementary Information. [file 41598_2023_44718_MOESM1_ESM.zip › Dataset/test set-system_evaluation/test_set_15fps/133.jpg]

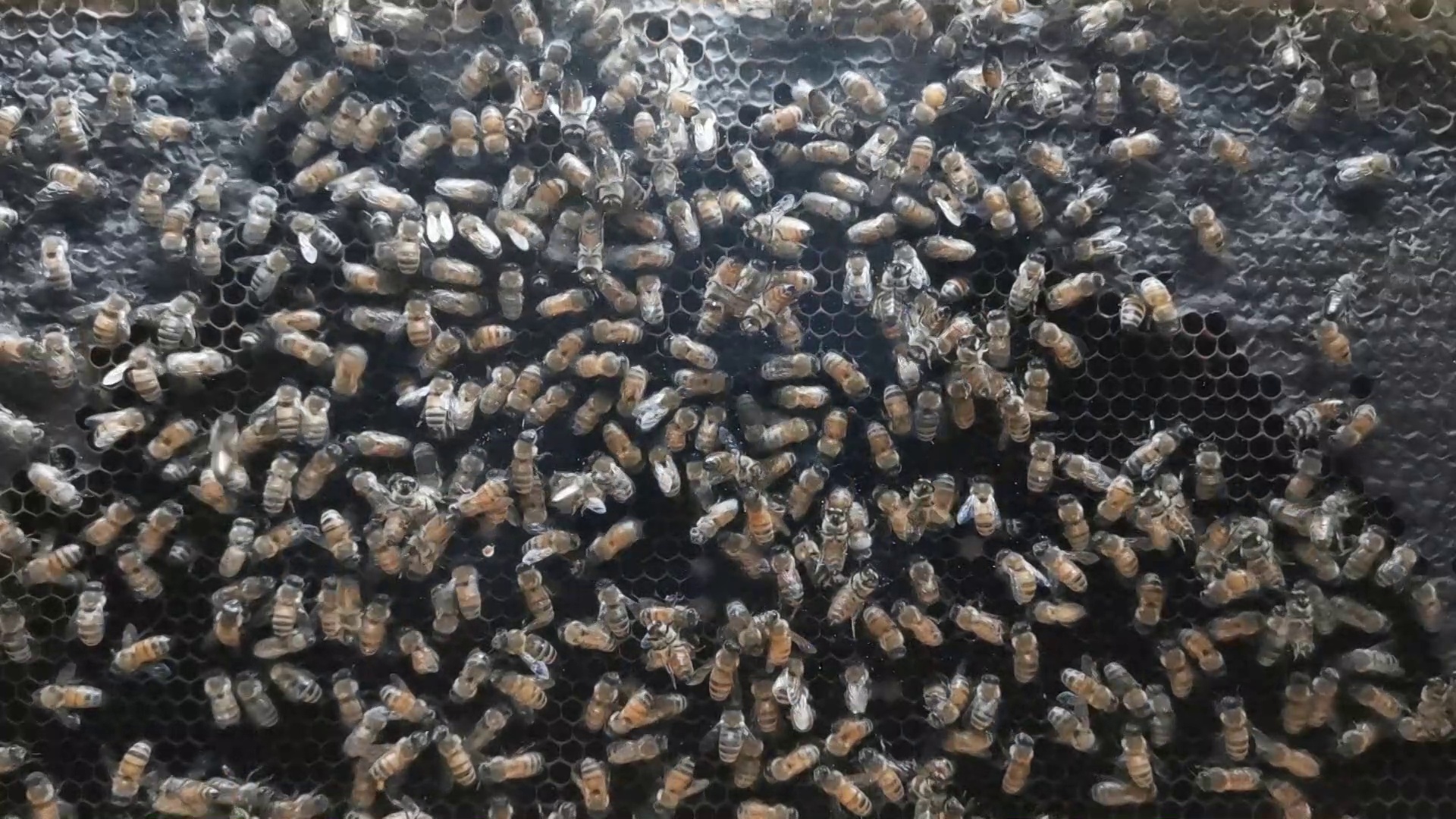

Supplement: Supplementary file 1 — Supplementary Information. [file 41598_2023_44718_MOESM1_ESM.zip › Dataset/test set-system_evaluation/test_set_15fps/129.jpg]

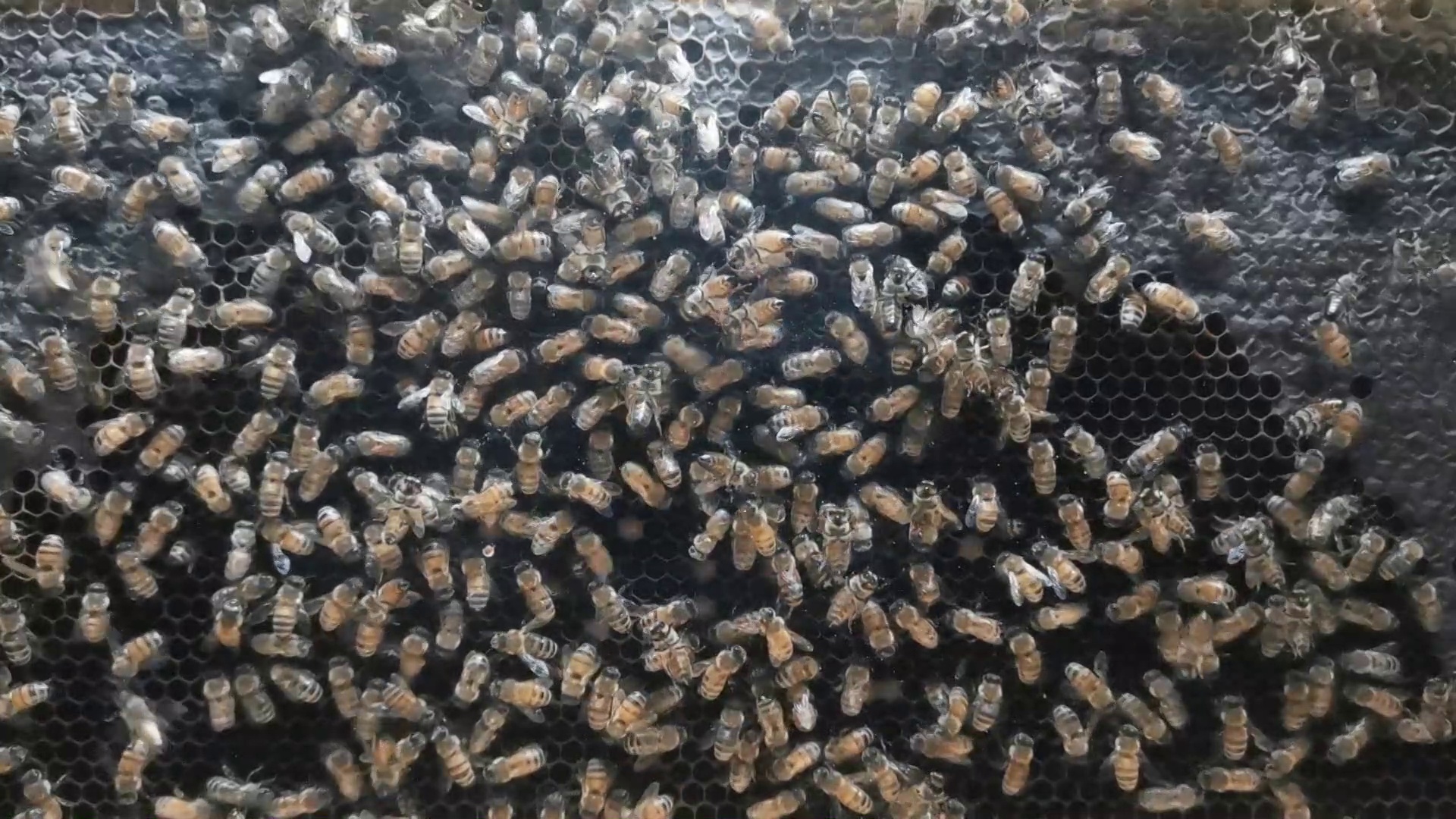

Supplement: Supplementary file 1 — Supplementary Information. [file 41598_2023_44718_MOESM1_ESM.zip › Dataset/test set-system_evaluation/test_set_15fps/142.jpg]

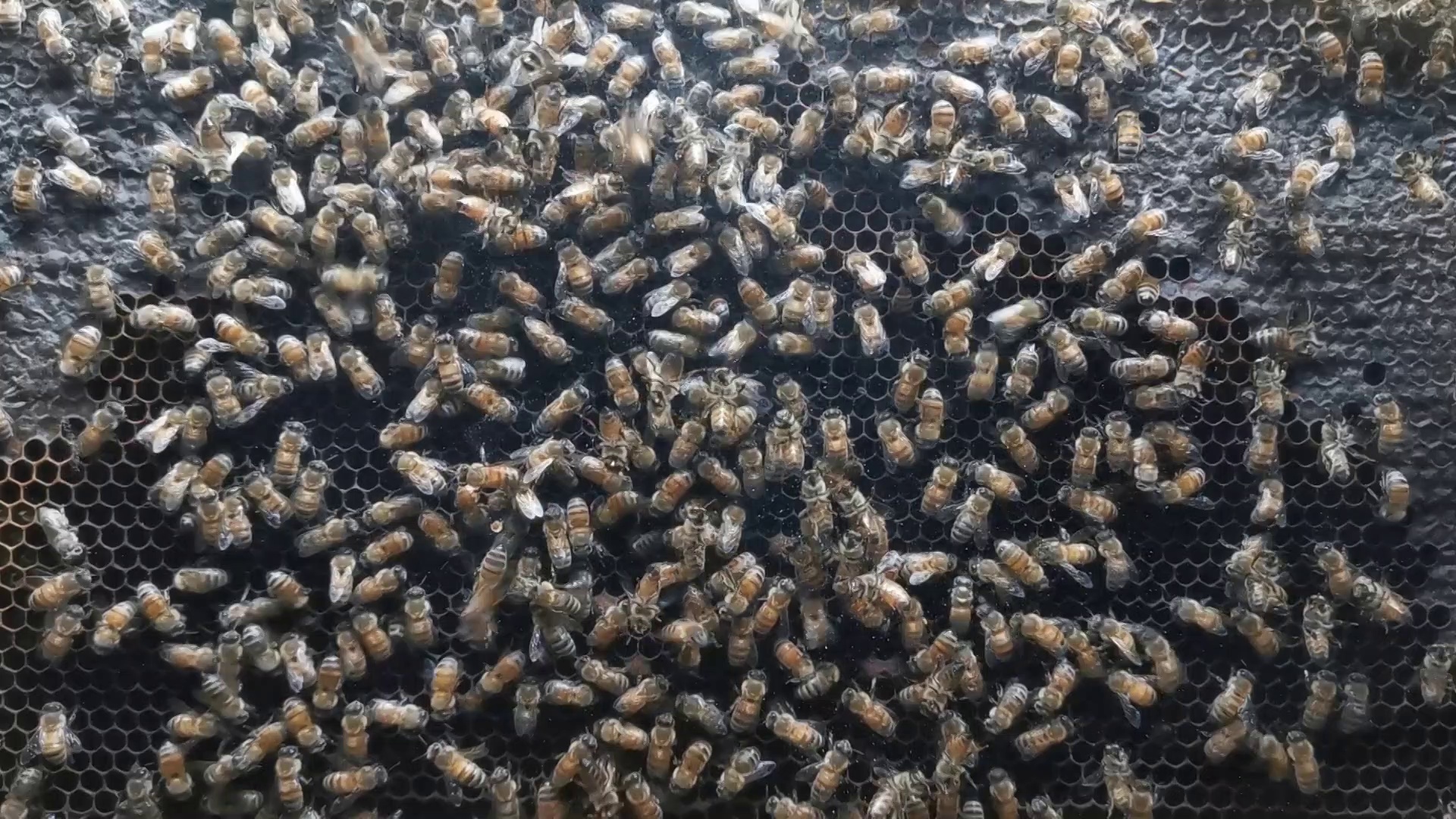

Supplement: Supplementary file 1 — Supplementary Information. [file 41598_2023_44718_MOESM1_ESM.zip › Dataset/dataset-Mask_RCNN_Training/dataset/test/001.jpg]

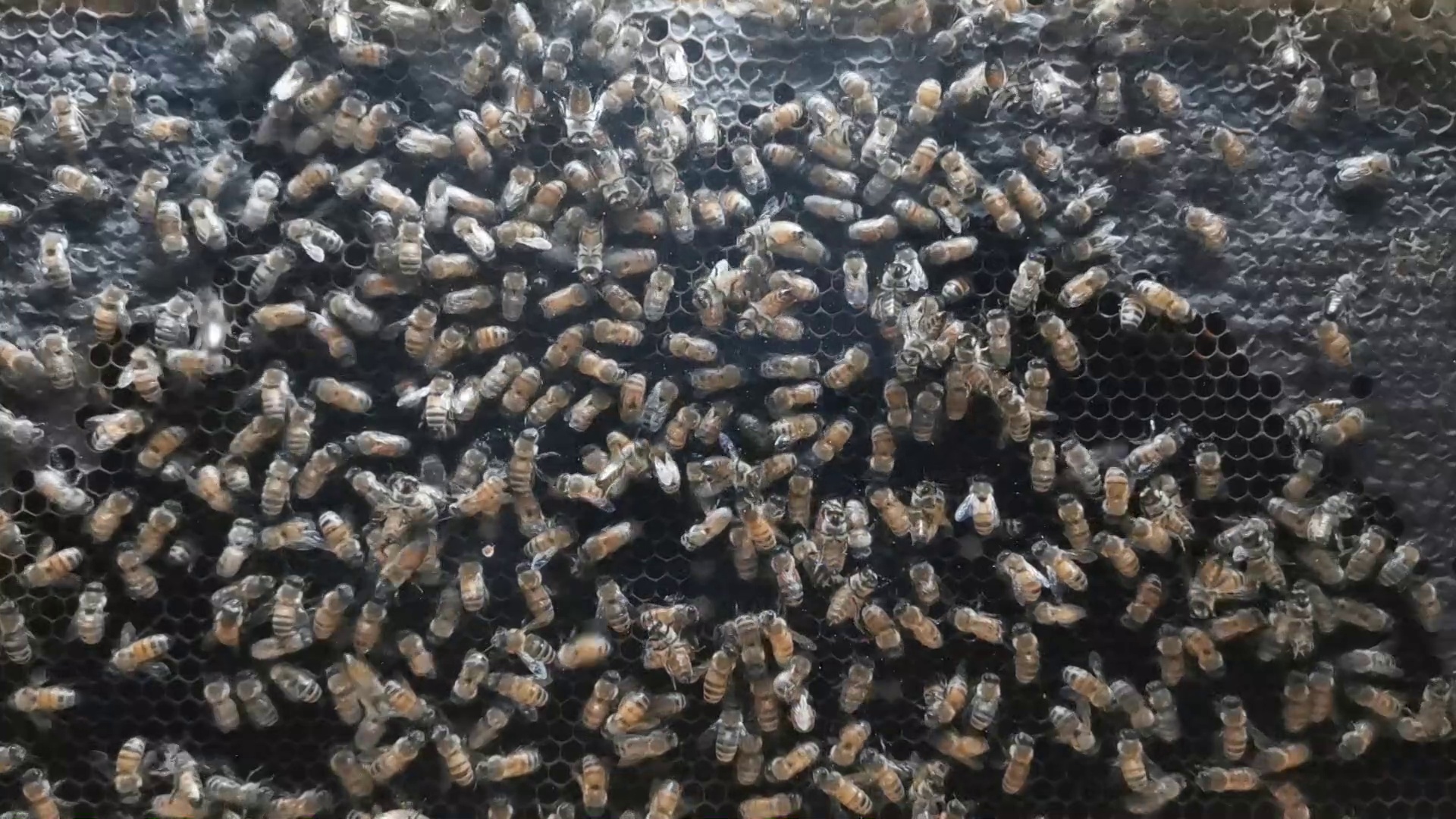

Supplement: Supplementary file 1 — Supplementary Information. [file 41598_2023_44718_MOESM1_ESM.zip › Dataset/test set-system_evaluation/test_set_15fps/134.jpg]

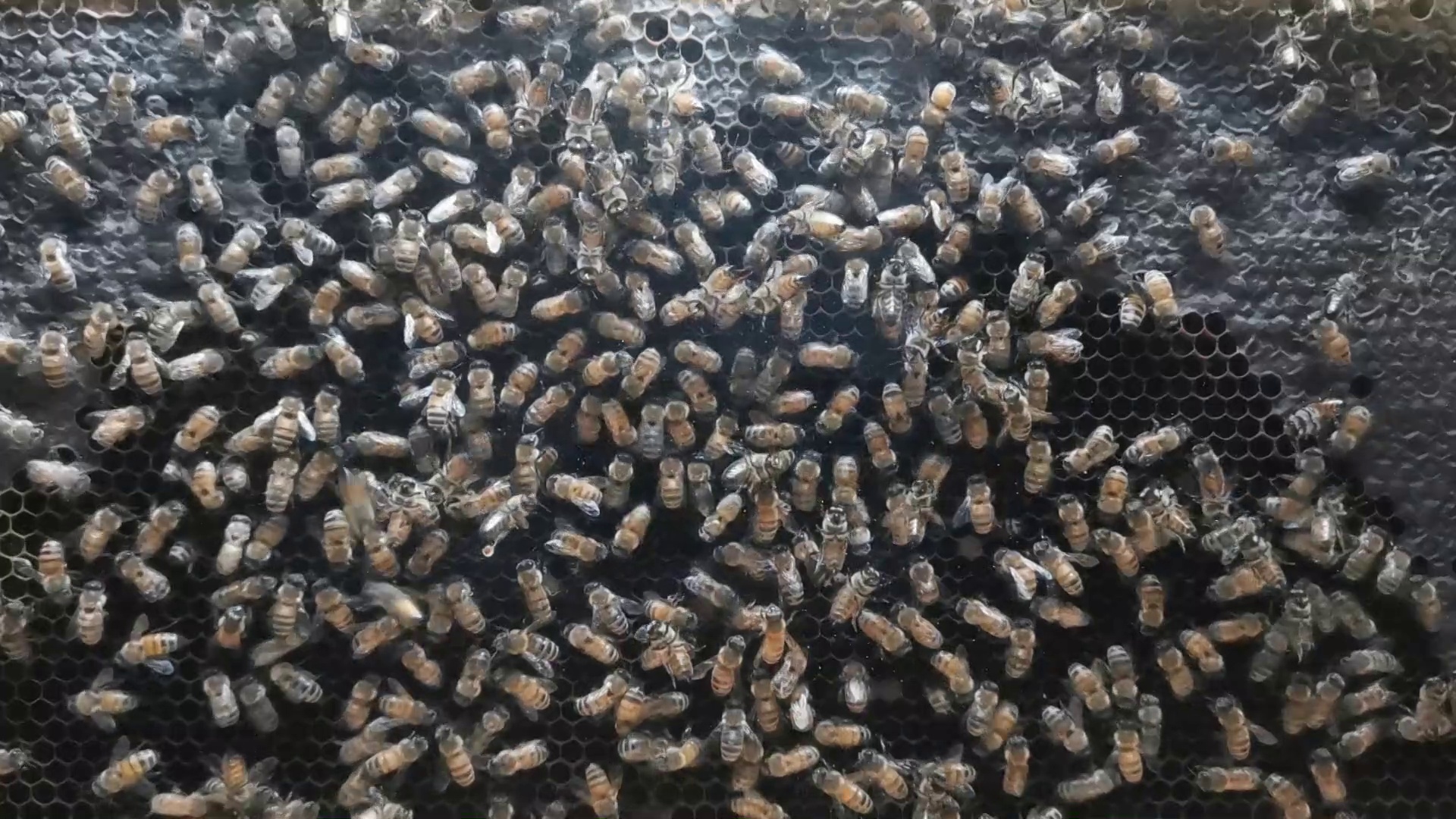

Supplement: Supplementary file 1 — Supplementary Information. [file 41598_2023_44718_MOESM1_ESM.zip › Dataset/test set-system_evaluation/test_set_15fps/119.jpg]

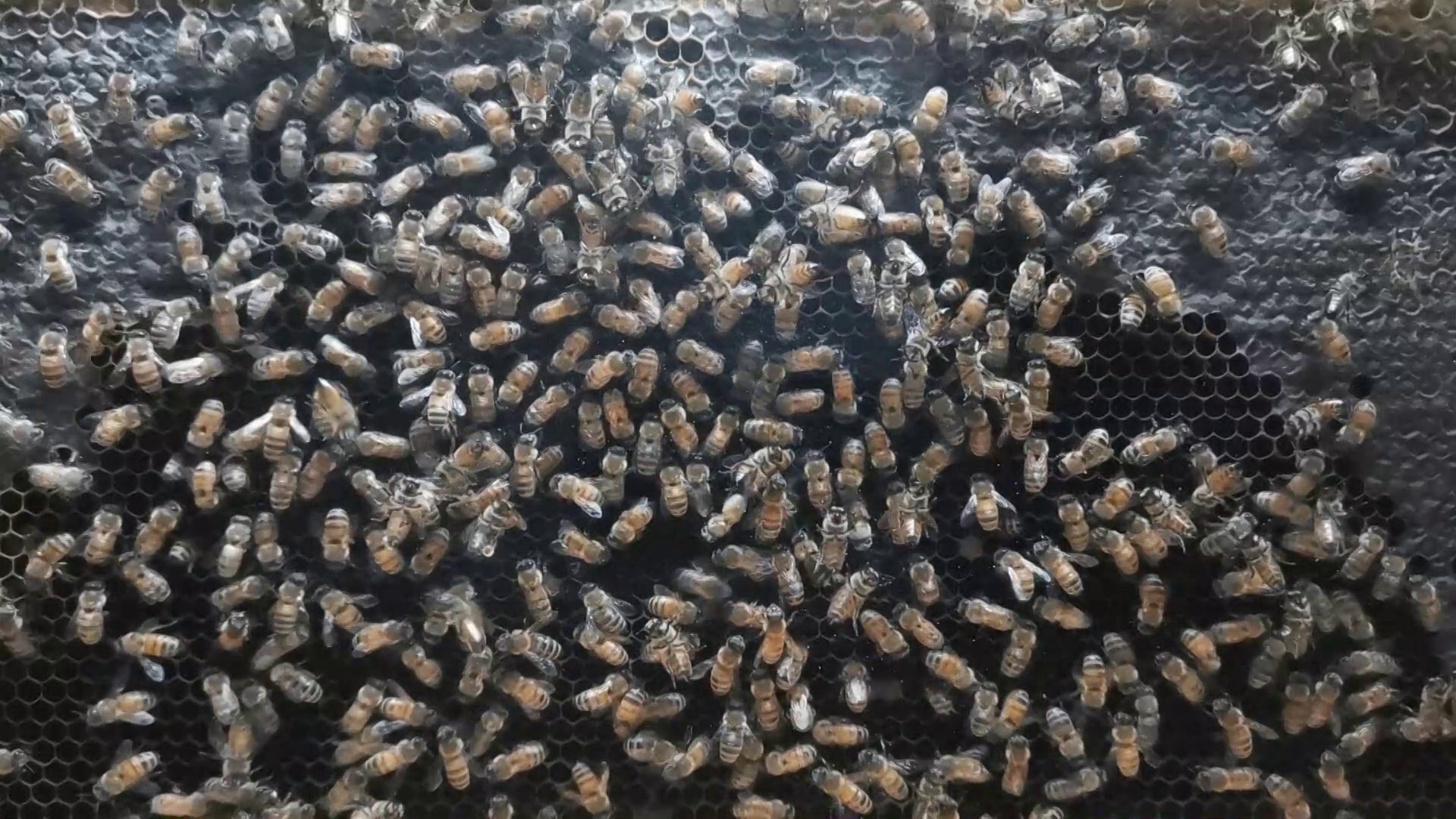

Supplement: Supplementary file 1 — Supplementary Information. [file 41598_2023_44718_MOESM1_ESM.zip › Dataset/test set-system_evaluation/test_set_15fps/115.jpg]

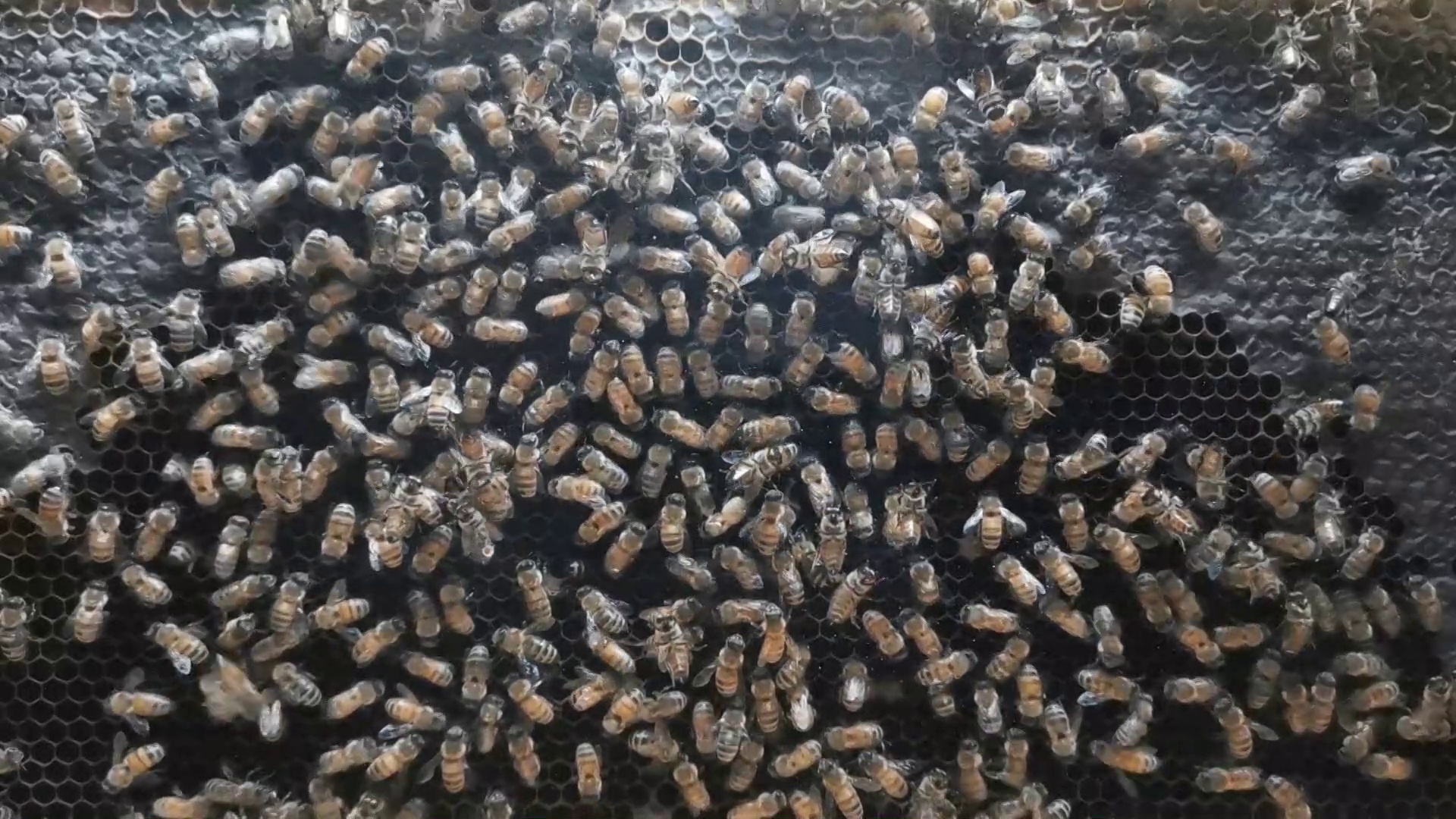

Supplement: Supplementary file 1 — Supplementary Information. [file 41598_2023_44718_MOESM1_ESM.zip › Dataset/test set-system_evaluation/test_set_15fps/100.jpg]

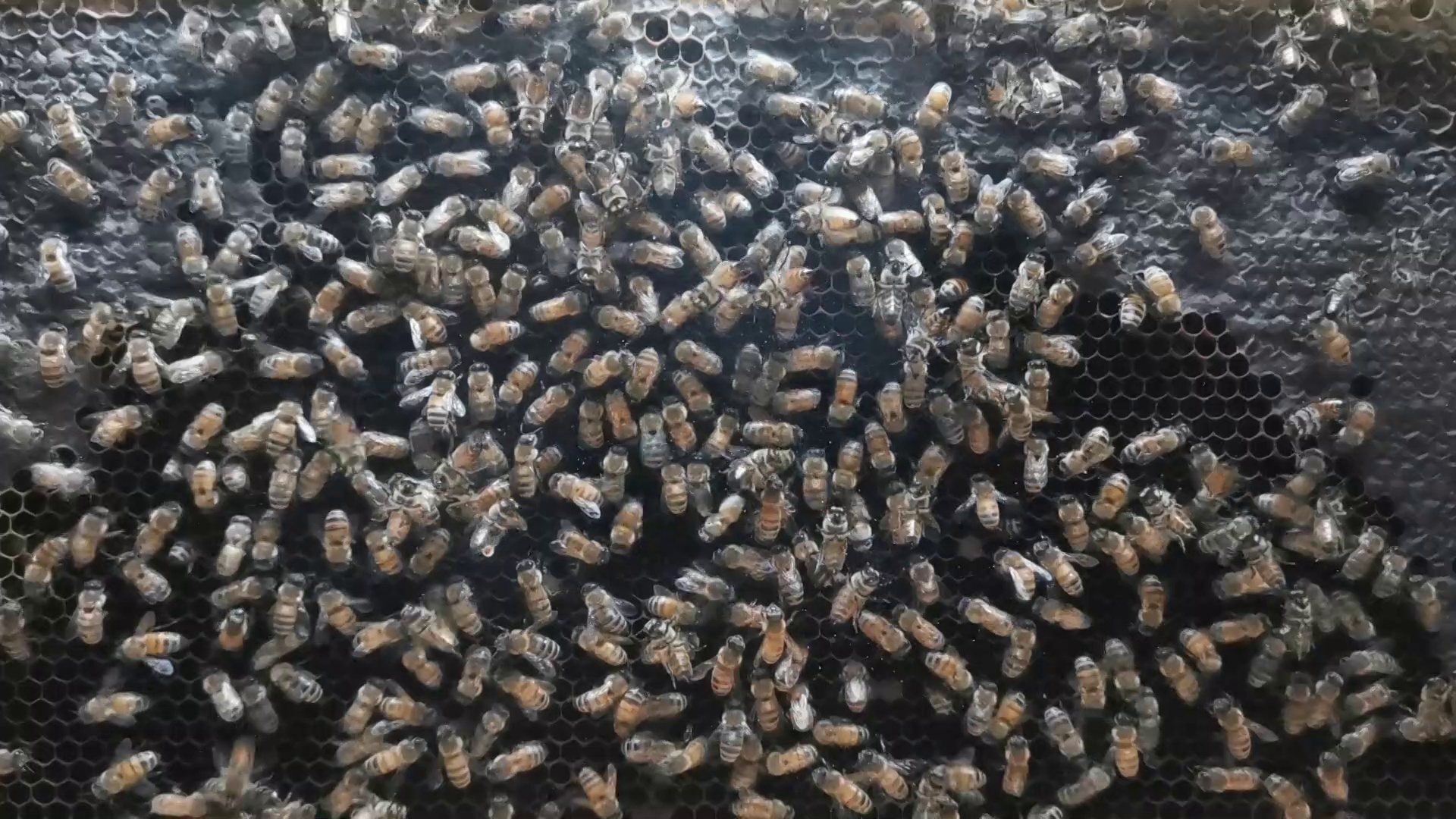

Supplement: Supplementary file 1 — Supplementary Information. [file 41598_2023_44718_MOESM1_ESM.zip › Dataset/test set-system_evaluation/test_set_15fps/116.jpg]

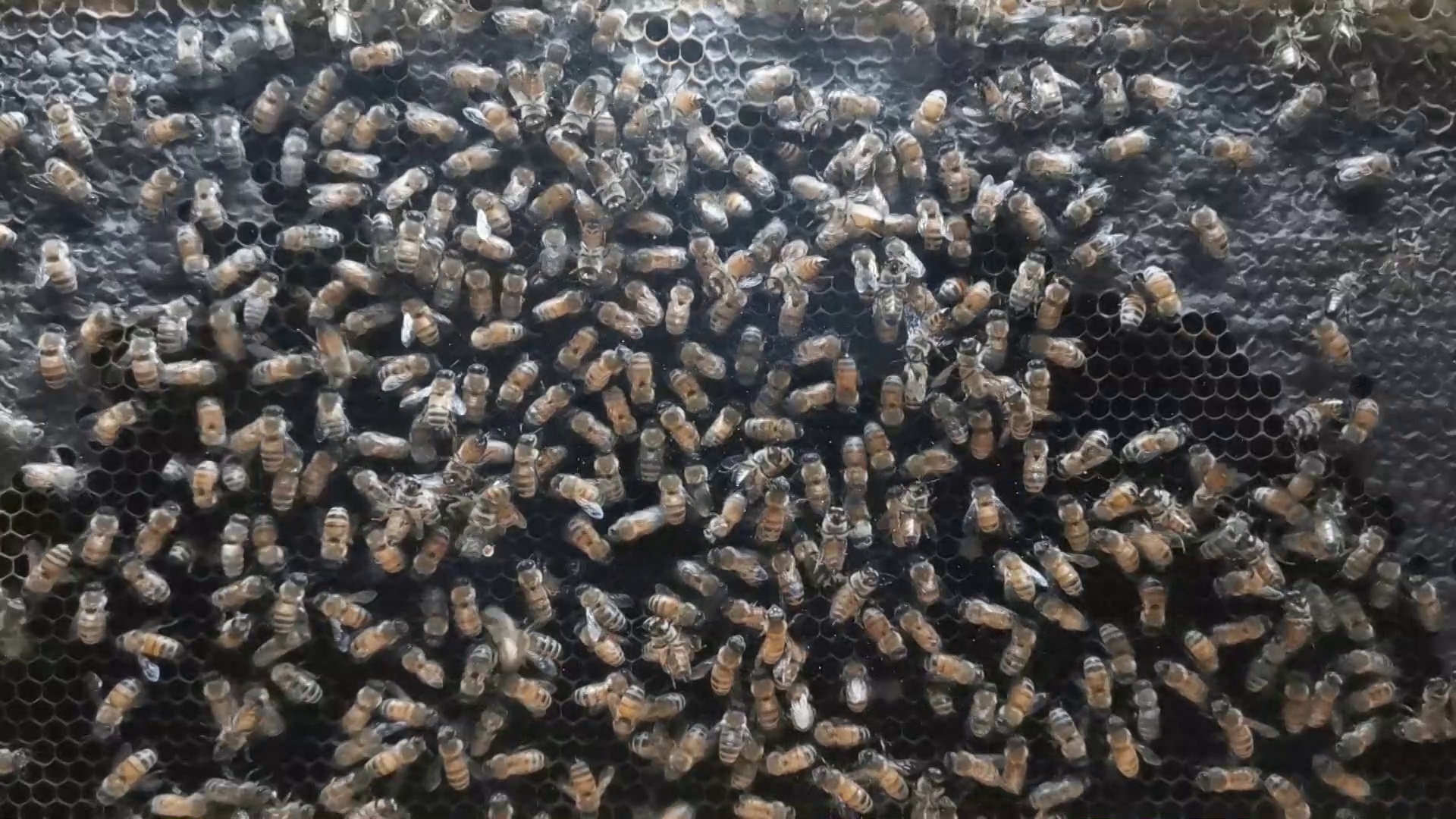

Supplement: Supplementary file 1 — Supplementary Information. [file 41598_2023_44718_MOESM1_ESM.zip › Dataset/test set-system_evaluation/test_set_15fps/112.jpg]

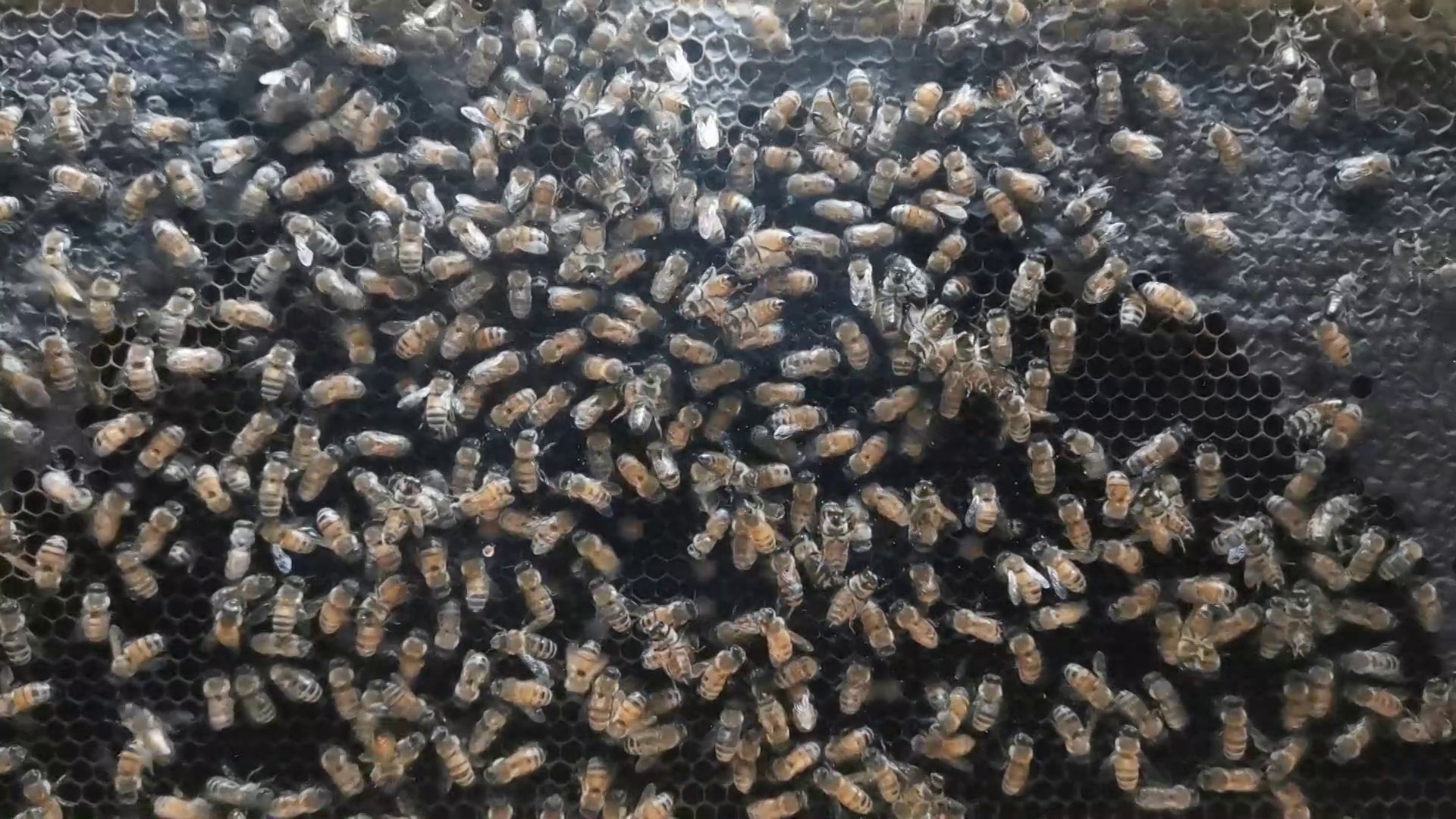

Supplement: Supplementary file 1 — Supplementary Information. [file 41598_2023_44718_MOESM1_ESM.zip › Dataset/test set-system_evaluation/test_set_15fps/141.jpg]

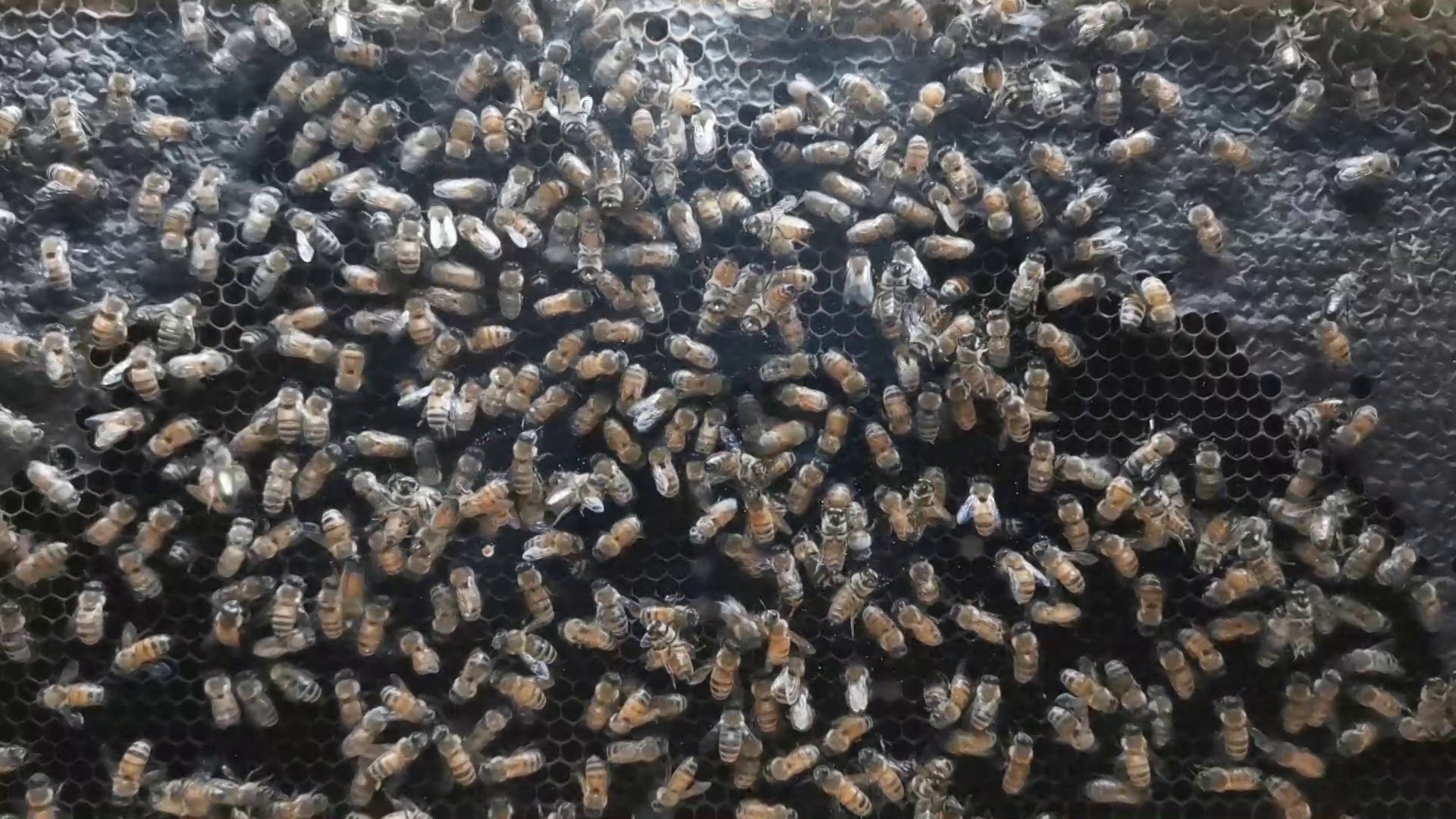

Supplement: Supplementary file 1 — Supplementary Information. [file 41598_2023_44718_MOESM1_ESM.zip › Dataset/test set-system_evaluation/test_set_15fps/128.jpg]

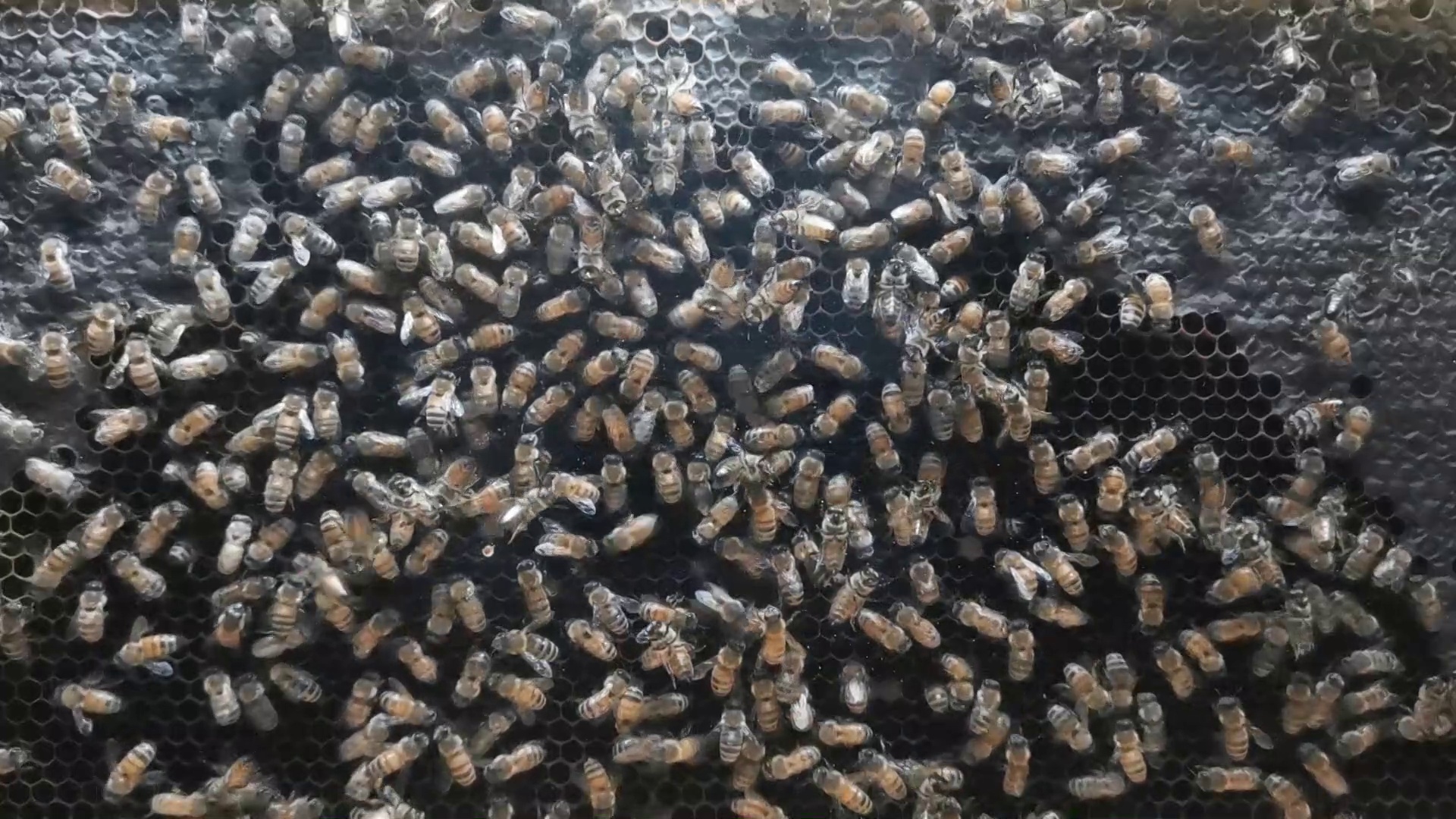

Supplement: Supplementary file 1 — Supplementary Information. [file 41598_2023_44718_MOESM1_ESM.zip › Dataset/test set-system_evaluation/test_set_15fps/122.jpg]

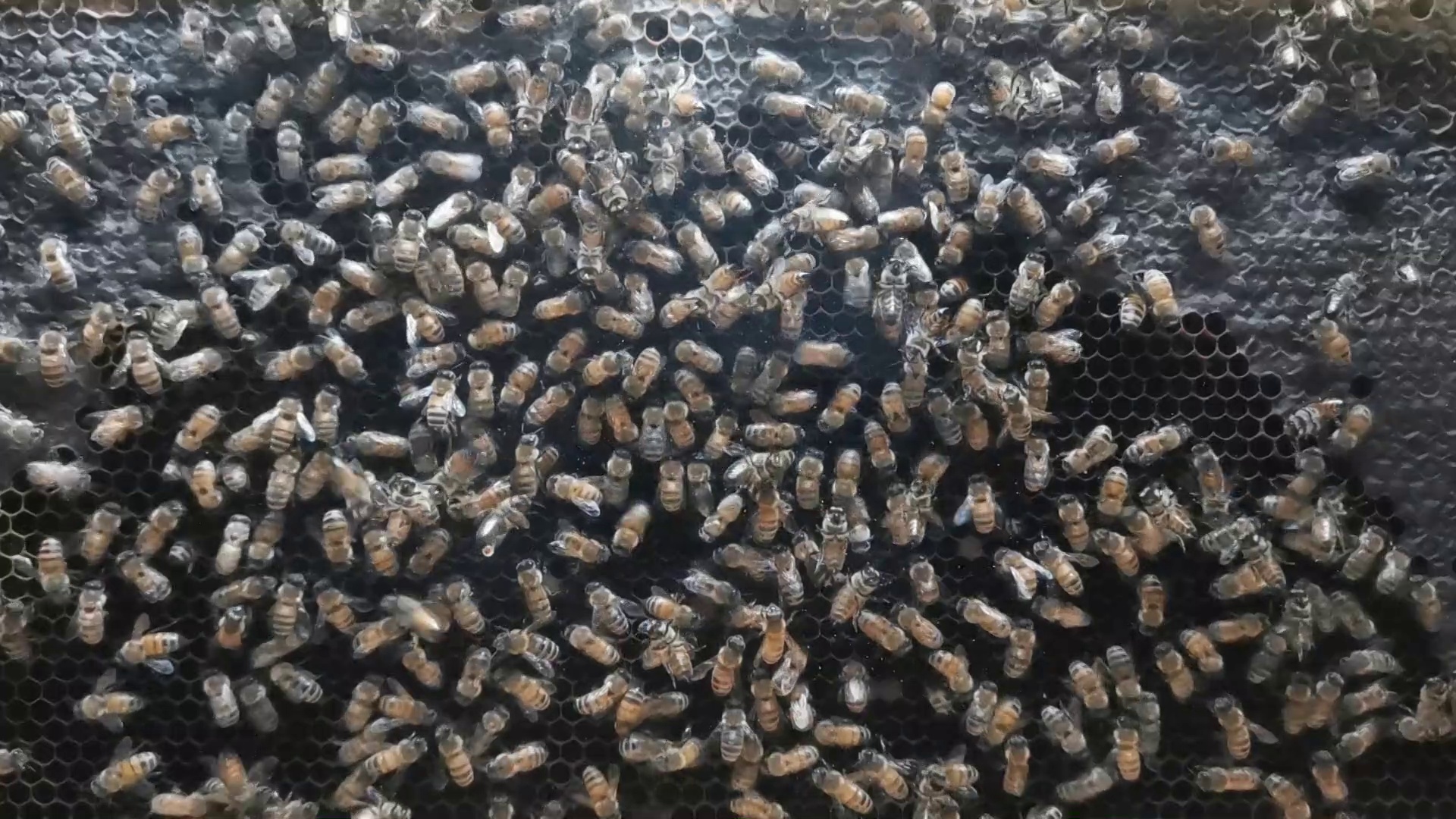

Supplement: Supplementary file 1 — Supplementary Information. [file 41598_2023_44718_MOESM1_ESM.zip › Dataset/test set-system_evaluation/test_set_15fps/118.jpg]

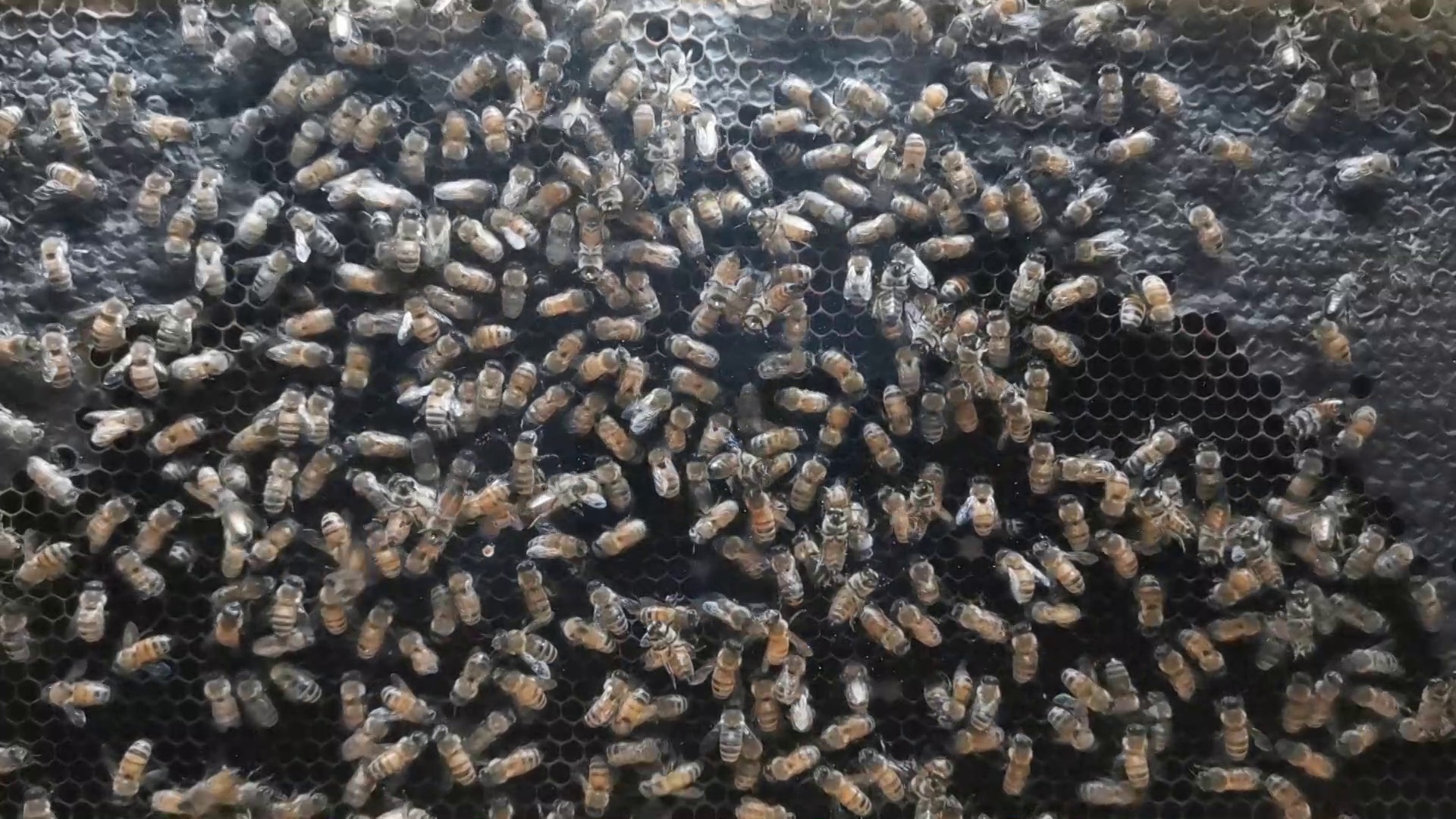

Supplement: Supplementary file 1 — Supplementary Information. [file 41598_2023_44718_MOESM1_ESM.zip › Dataset/test set-system_evaluation/test_set_15fps/126.jpg]

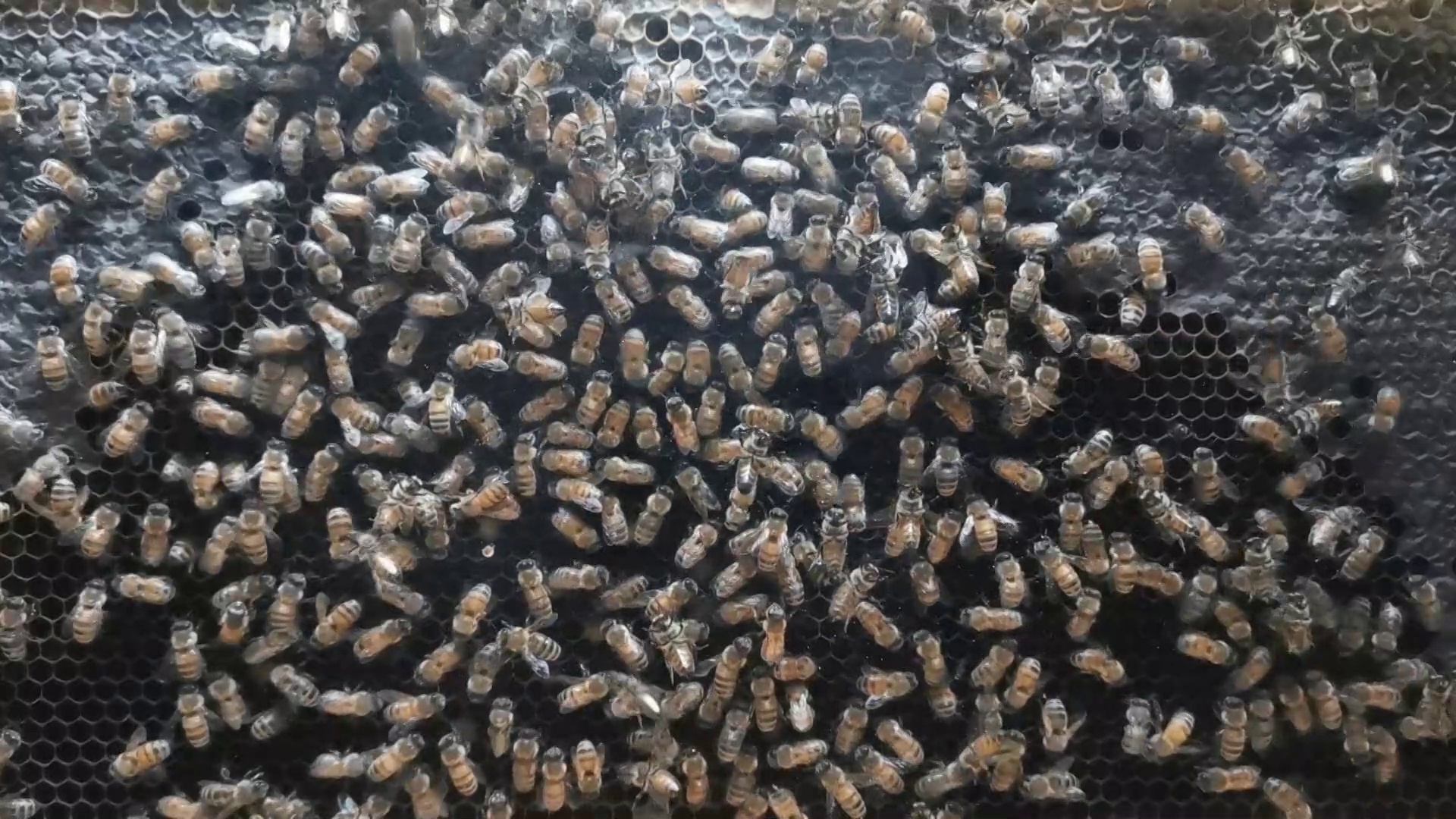

Supplement: Supplementary file 1 — Supplementary Information. [file 41598_2023_44718_MOESM1_ESM.zip › Dataset/test set-system_evaluation/test_set_15fps/068.jpg]

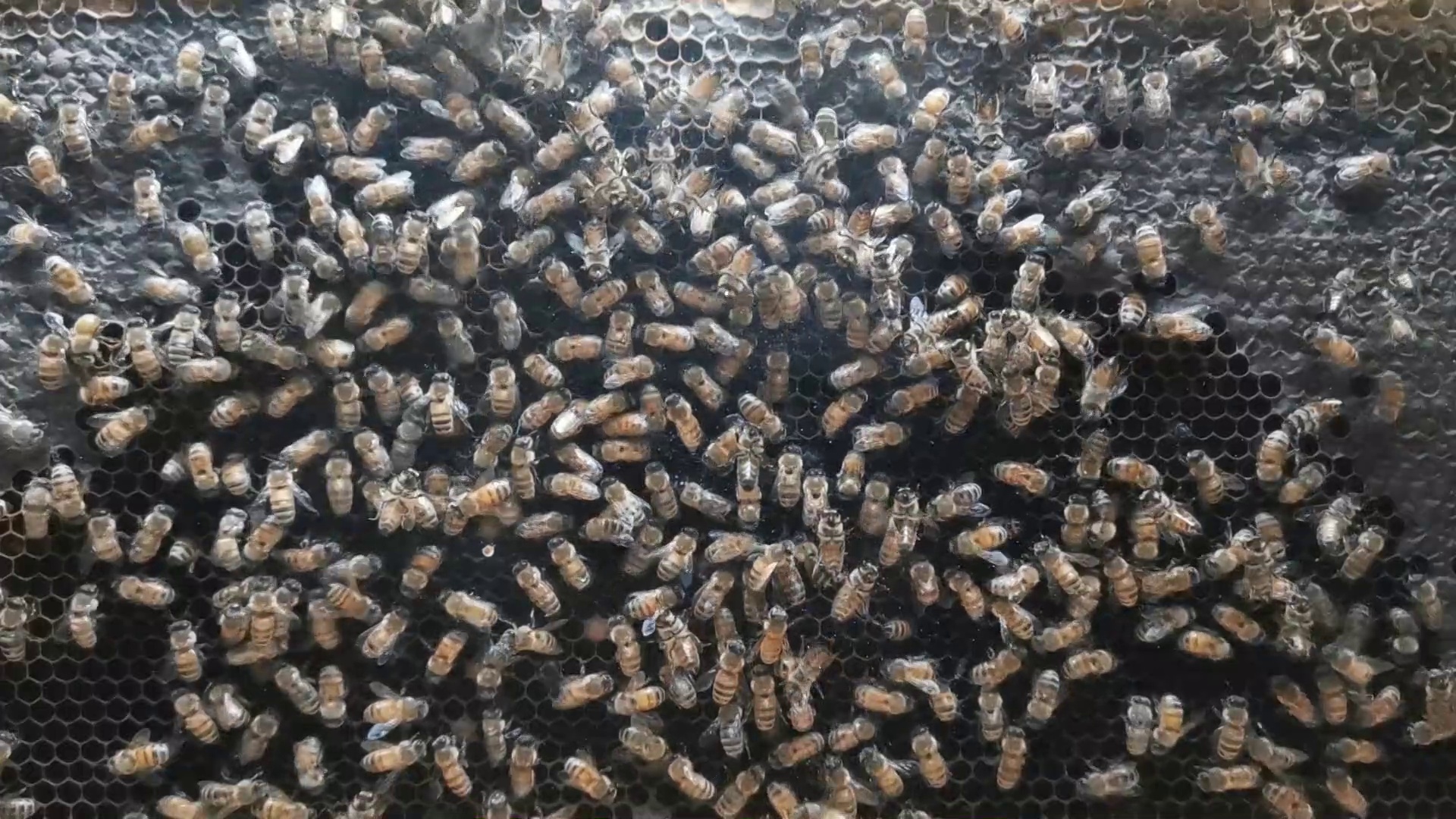

Supplement: Supplementary file 1 — Supplementary Information. [file 41598_2023_44718_MOESM1_ESM.zip › Dataset/test set-system_evaluation/test_set_15fps/035.jpg]

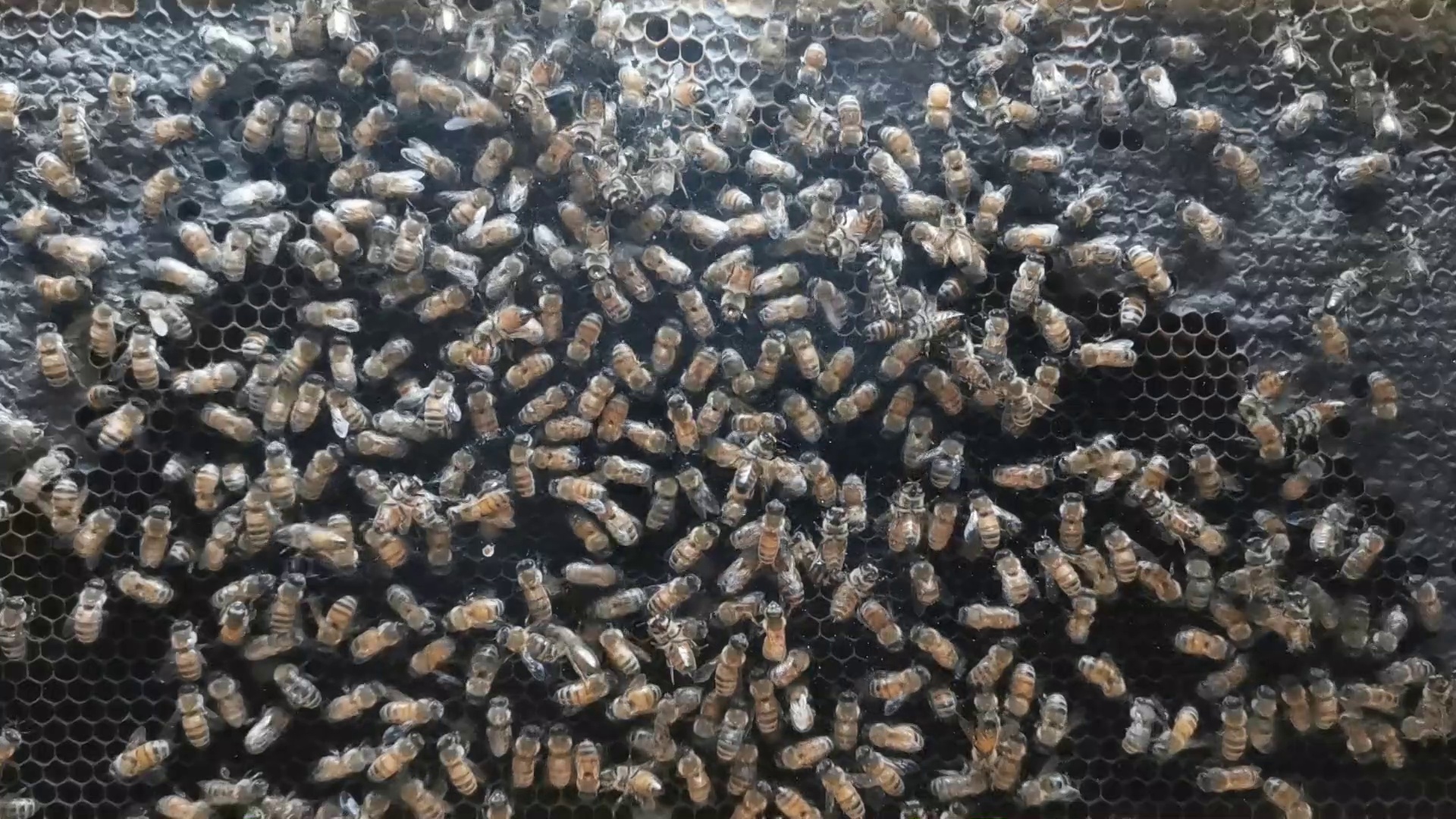

Supplement: Supplementary file 1 — Supplementary Information. [file 41598_2023_44718_MOESM1_ESM.zip › Dataset/test set-system_evaluation/test_set_15fps/075.jpg]

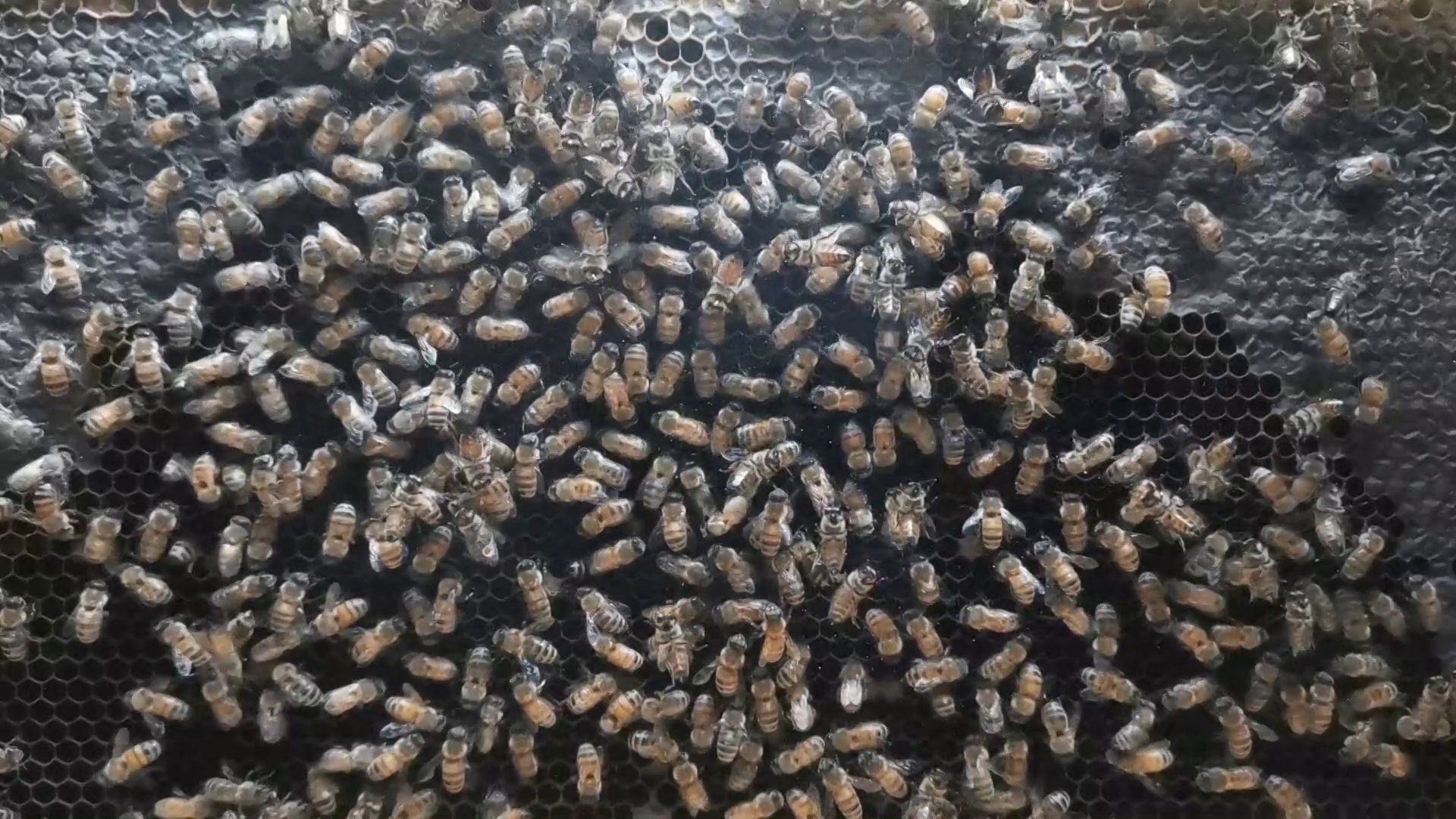

Supplement: Supplementary file 1 — Supplementary Information. [file 41598_2023_44718_MOESM1_ESM.zip › Dataset/test set-system_evaluation/test_set_15fps/097.jpg]

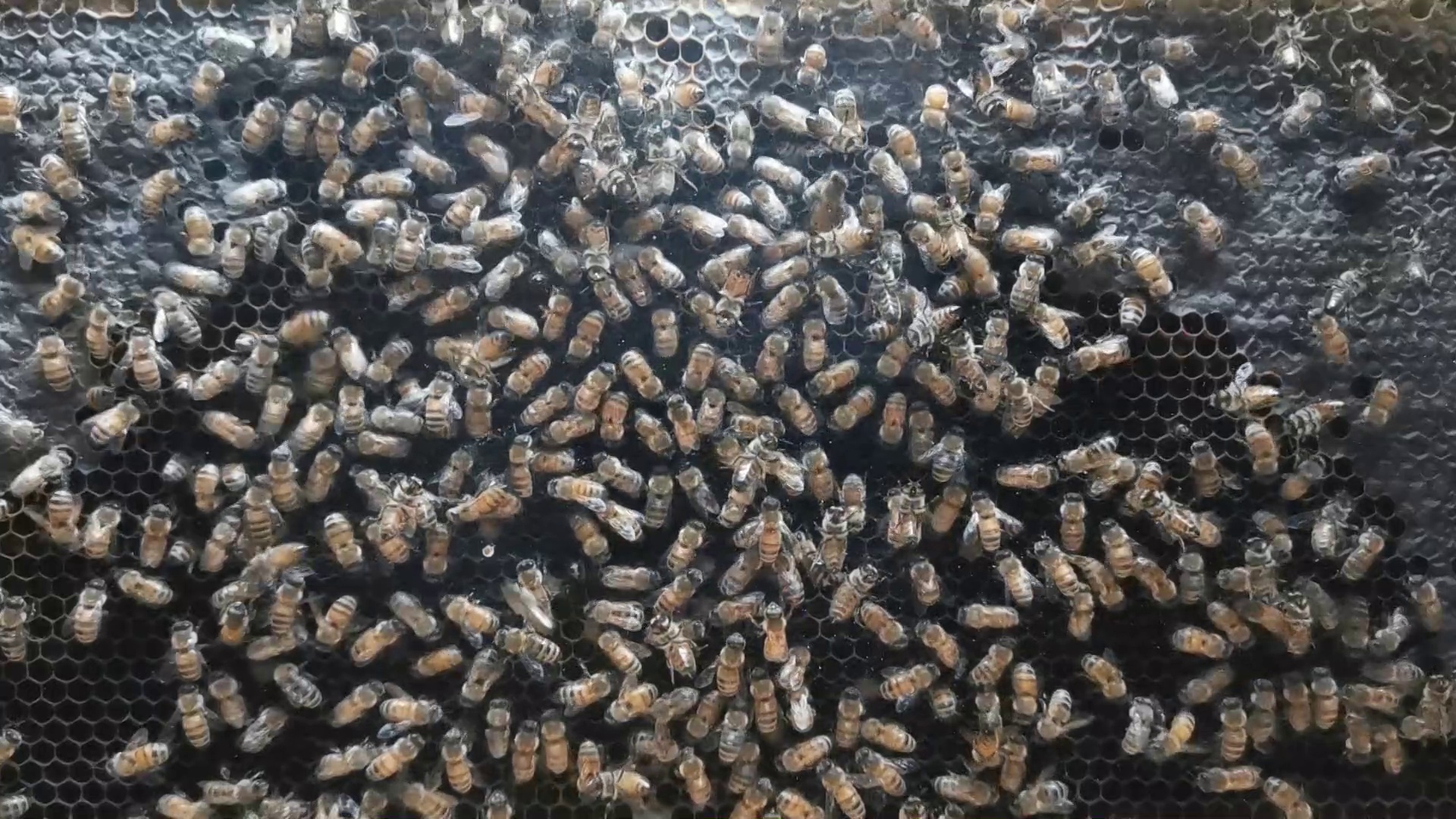

Supplement: Supplementary file 1 — Supplementary Information. [file 41598_2023_44718_MOESM1_ESM.zip › Dataset/test set-system_evaluation/test_set_15fps/079.jpg]

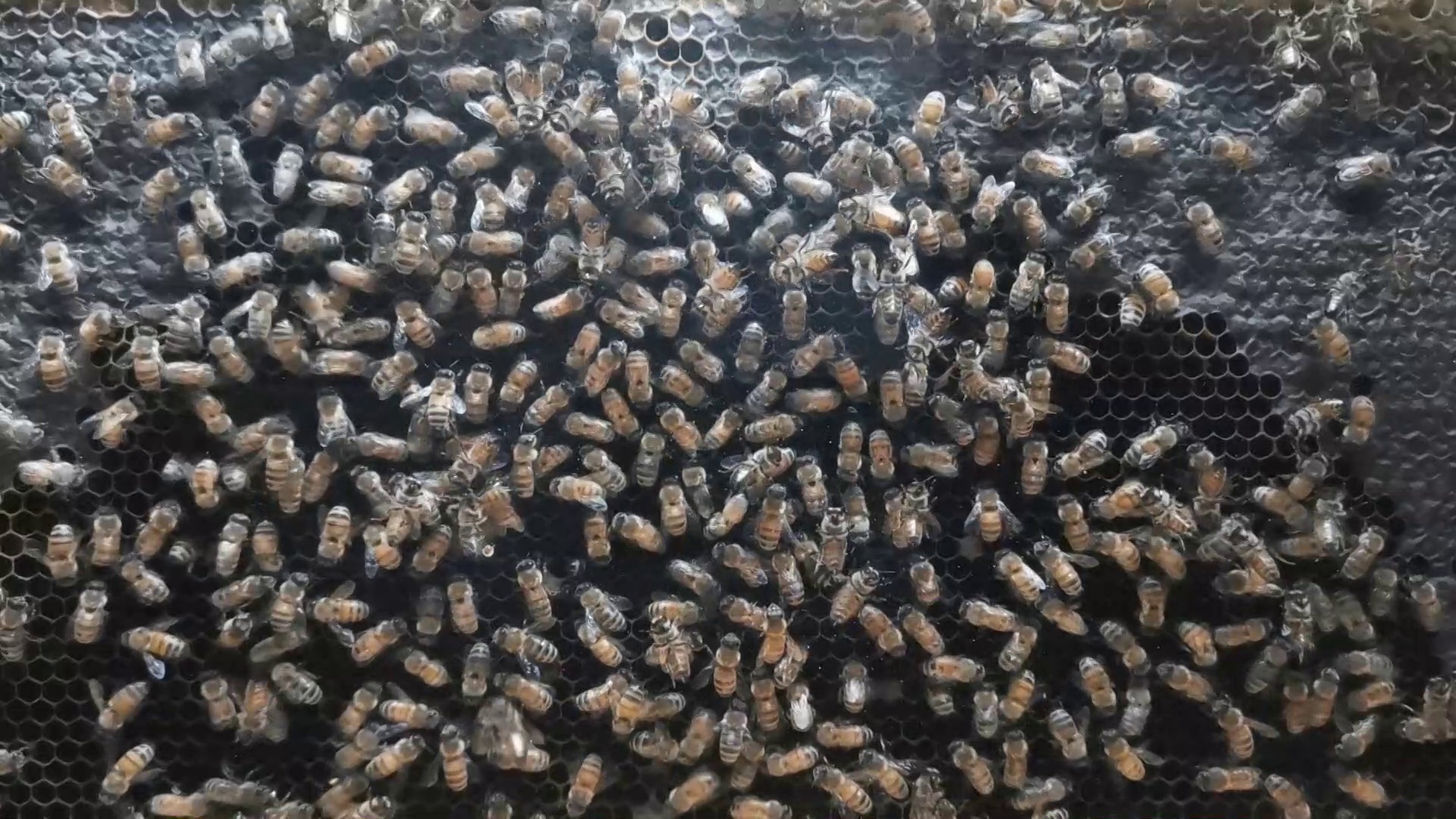

Supplement: Supplementary file 1 — Supplementary Information. [file 41598_2023_44718_MOESM1_ESM.zip › Dataset/test set-system_evaluation/test_set_15fps/108.jpg]

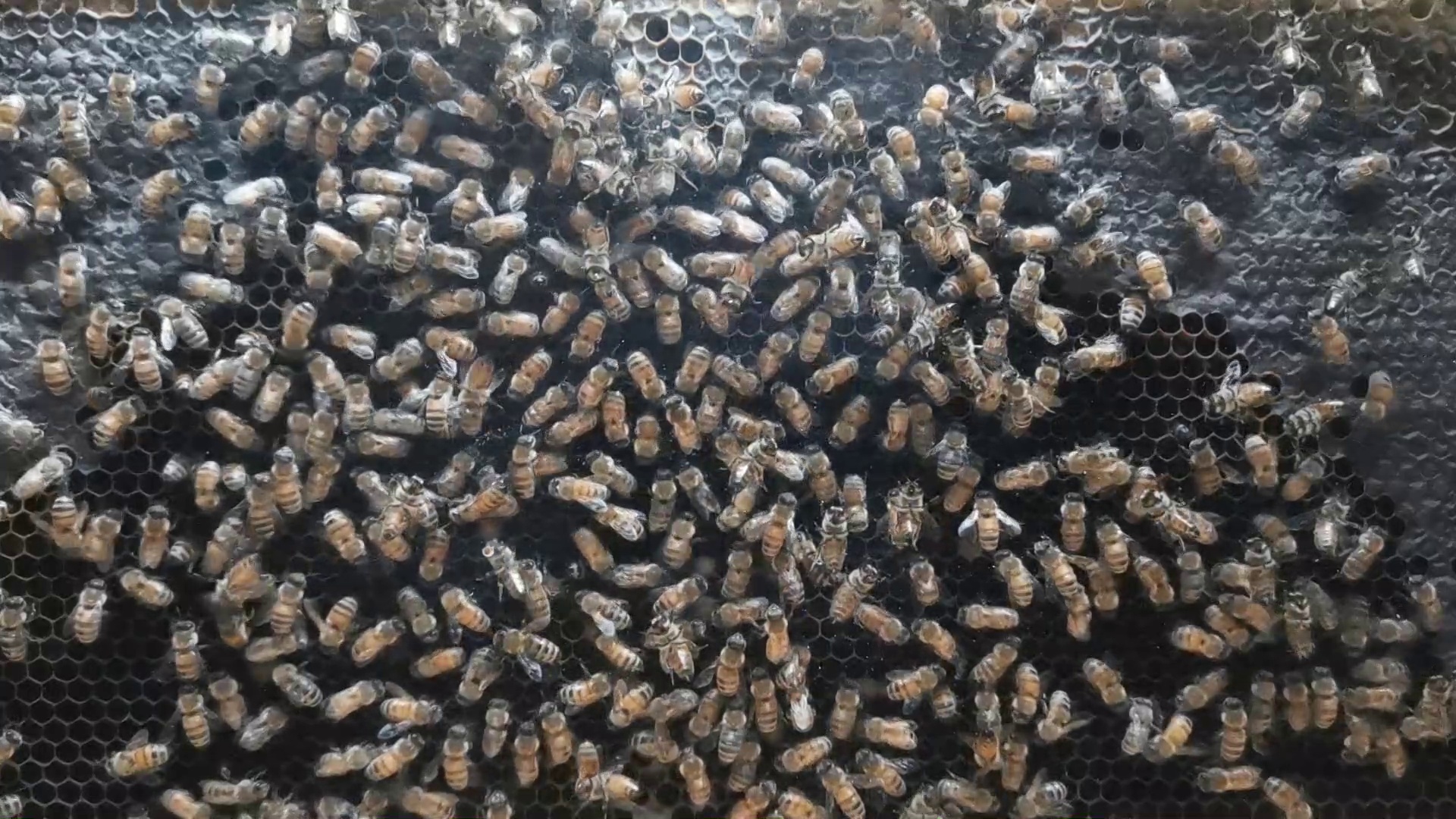

Supplement: Supplementary file 1 — Supplementary Information. [file 41598_2023_44718_MOESM1_ESM.zip › Dataset/test set-system_evaluation/test_set_15fps/083.jpg]

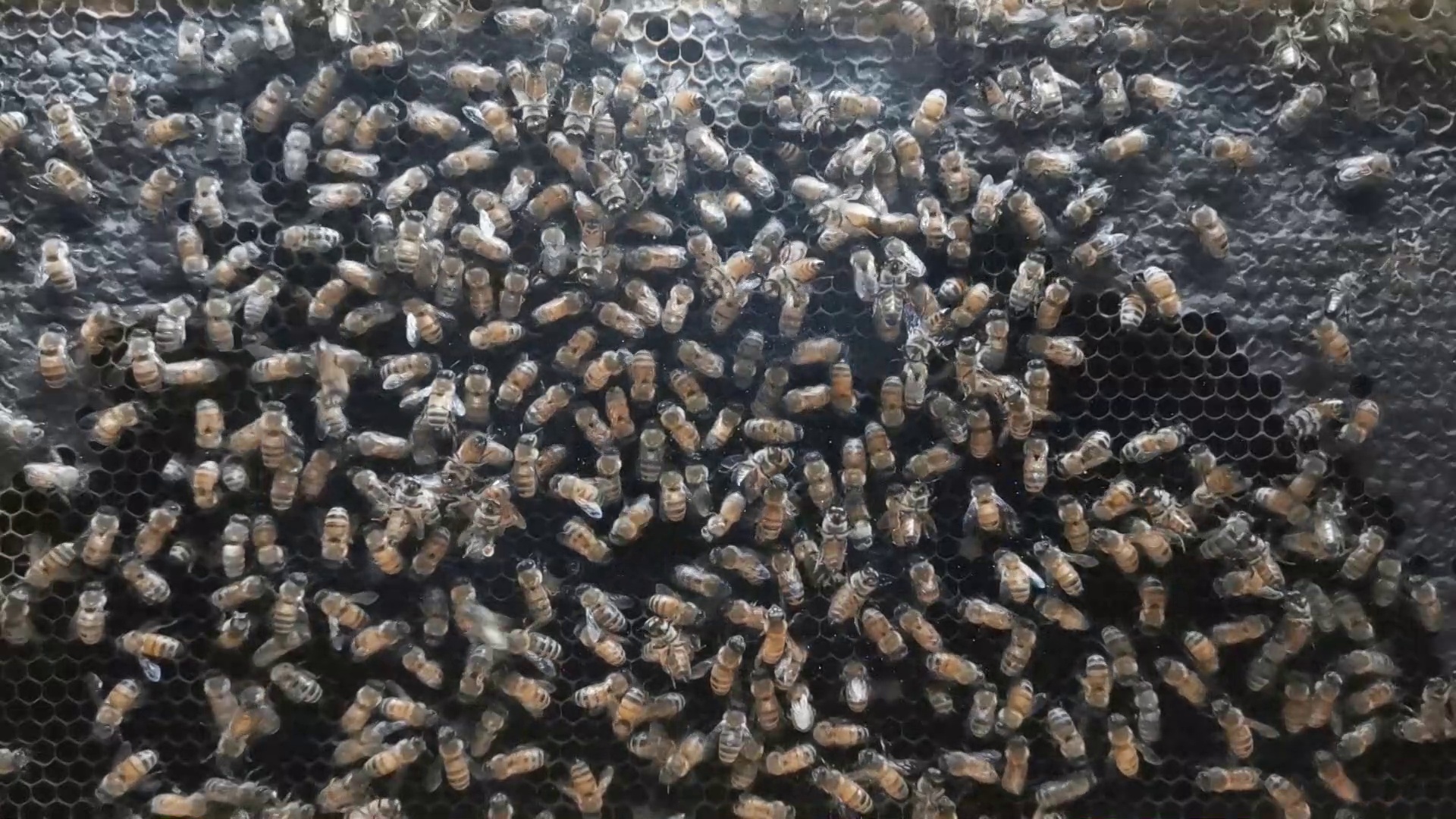

Supplement: Supplementary file 1 — Supplementary Information. [file 41598_2023_44718_MOESM1_ESM.zip › Dataset/test set-system_evaluation/test_set_15fps/113.jpg]

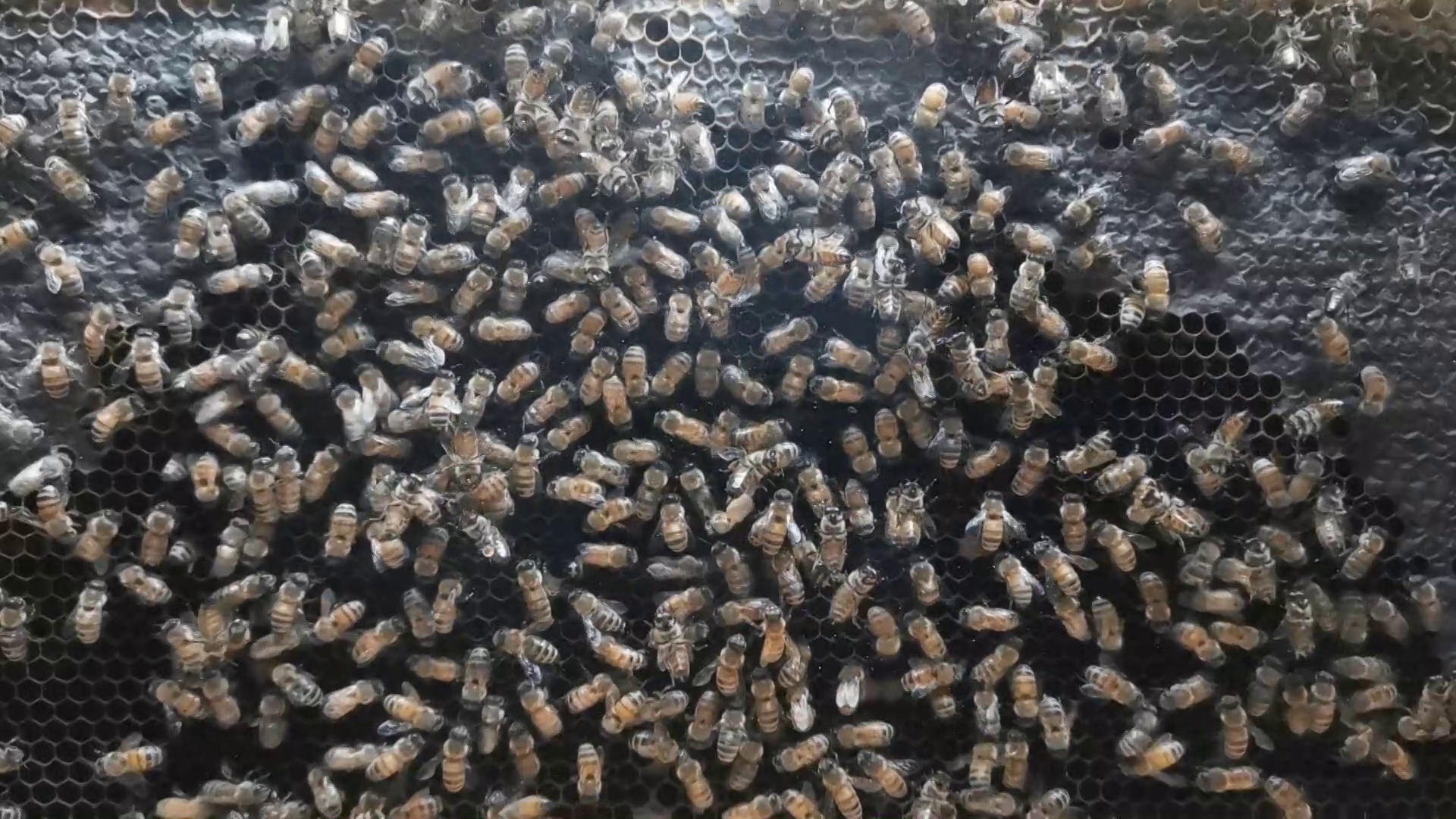

Supplement: Supplementary file 1 — Supplementary Information. [file 41598_2023_44718_MOESM1_ESM.zip › Dataset/test set-system_evaluation/test_set_15fps/094.jpg]

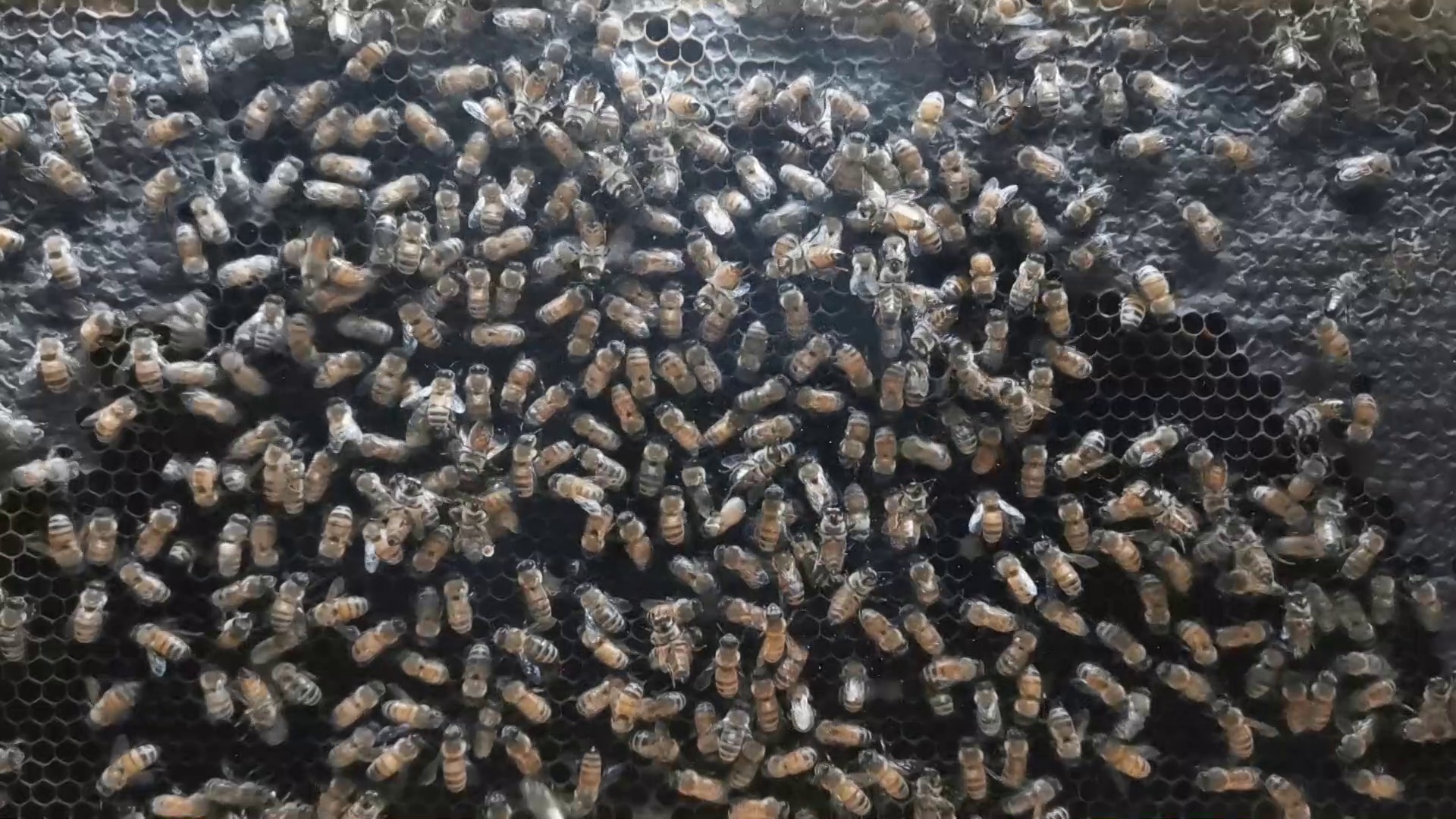

Supplement: Supplementary file 1 — Supplementary Information. [file 41598_2023_44718_MOESM1_ESM.zip › Dataset/test set-system_evaluation/test_set_15fps/105.jpg]

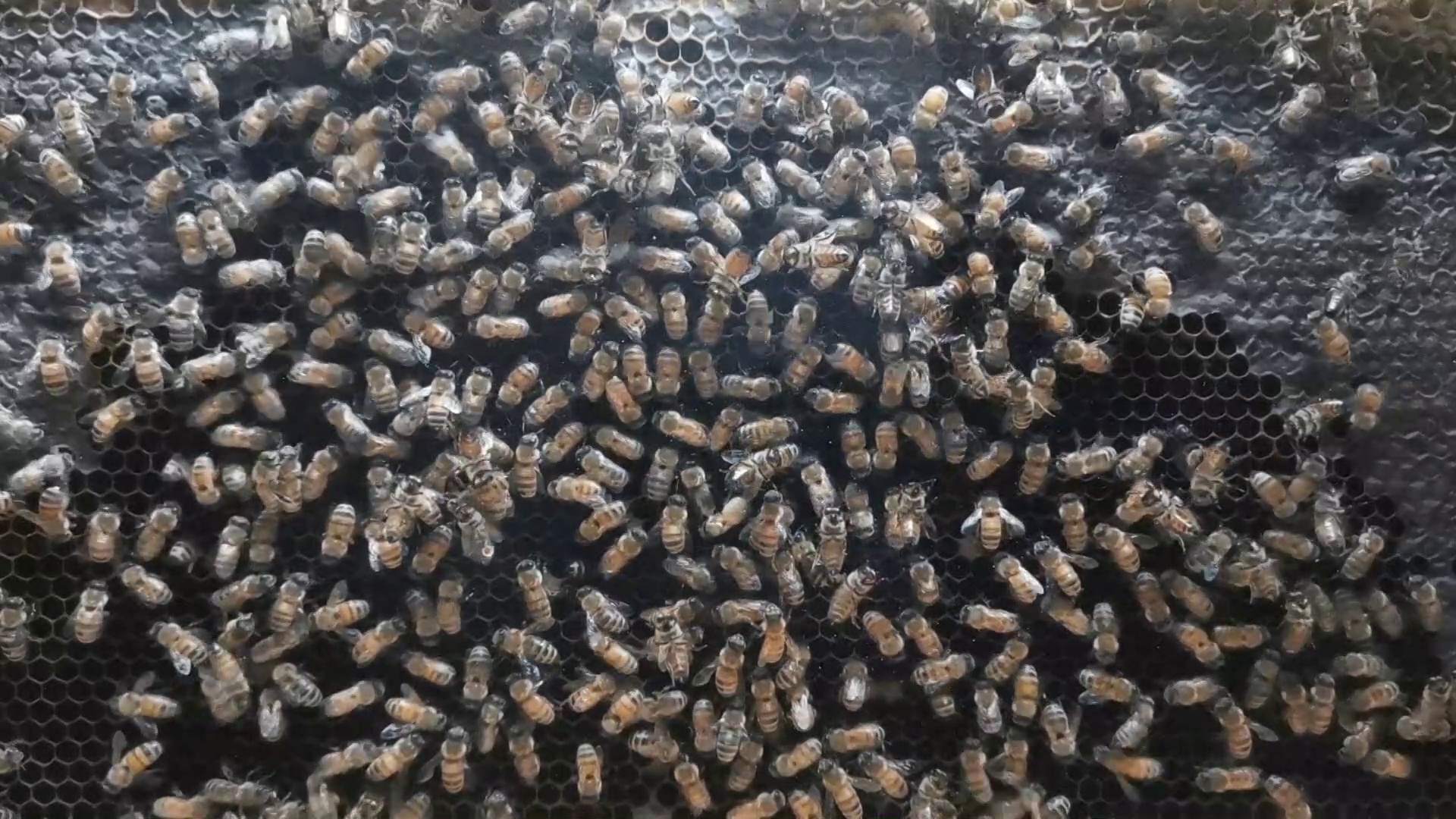

Supplement: Supplementary file 1 — Supplementary Information. [file 41598_2023_44718_MOESM1_ESM.zip › Dataset/test set-system_evaluation/test_set_15fps/099.jpg]

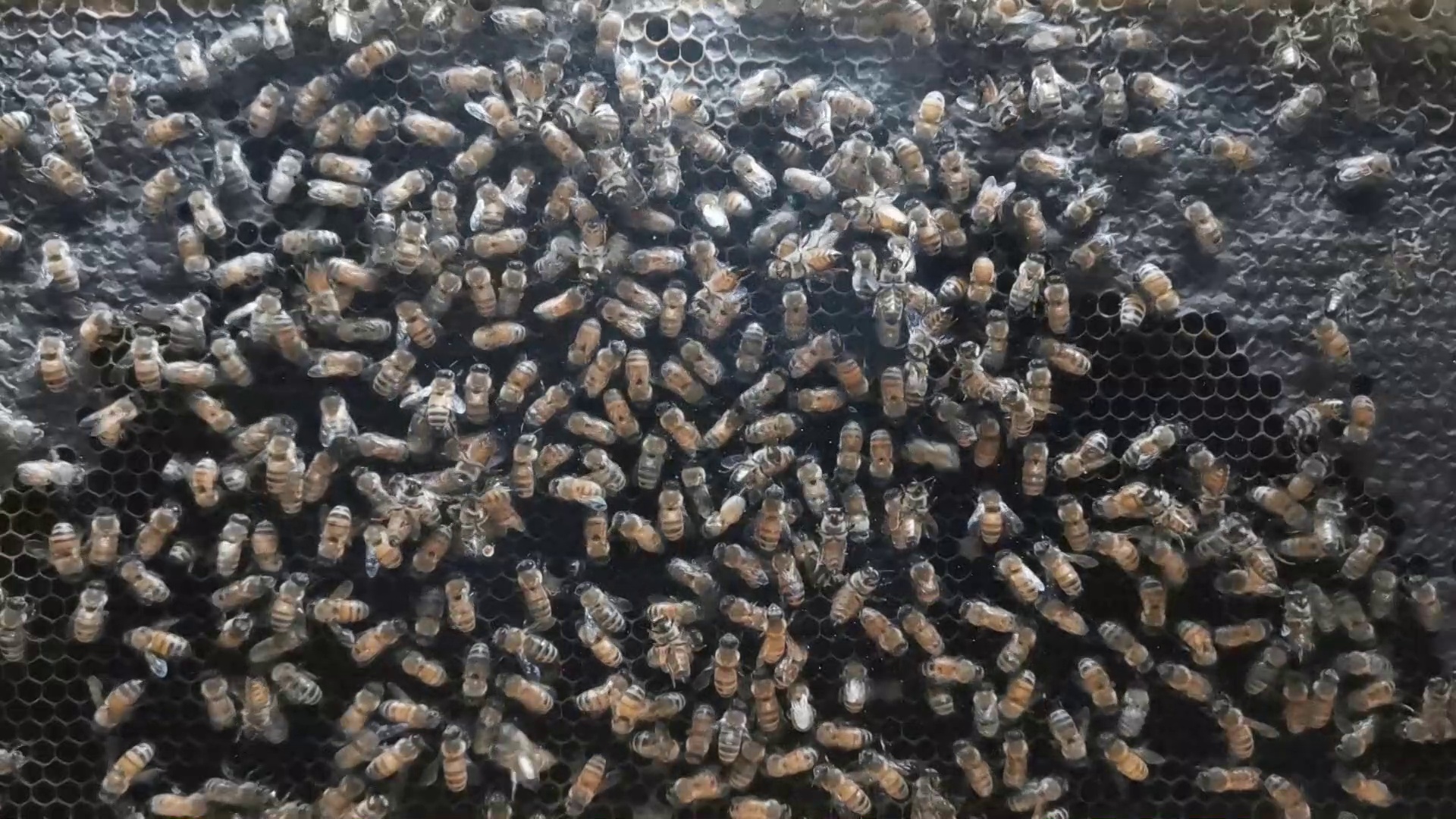

Supplement: Supplementary file 1 — Supplementary Information. [file 41598_2023_44718_MOESM1_ESM.zip › Dataset/test set-system_evaluation/test_set_15fps/107.jpg]

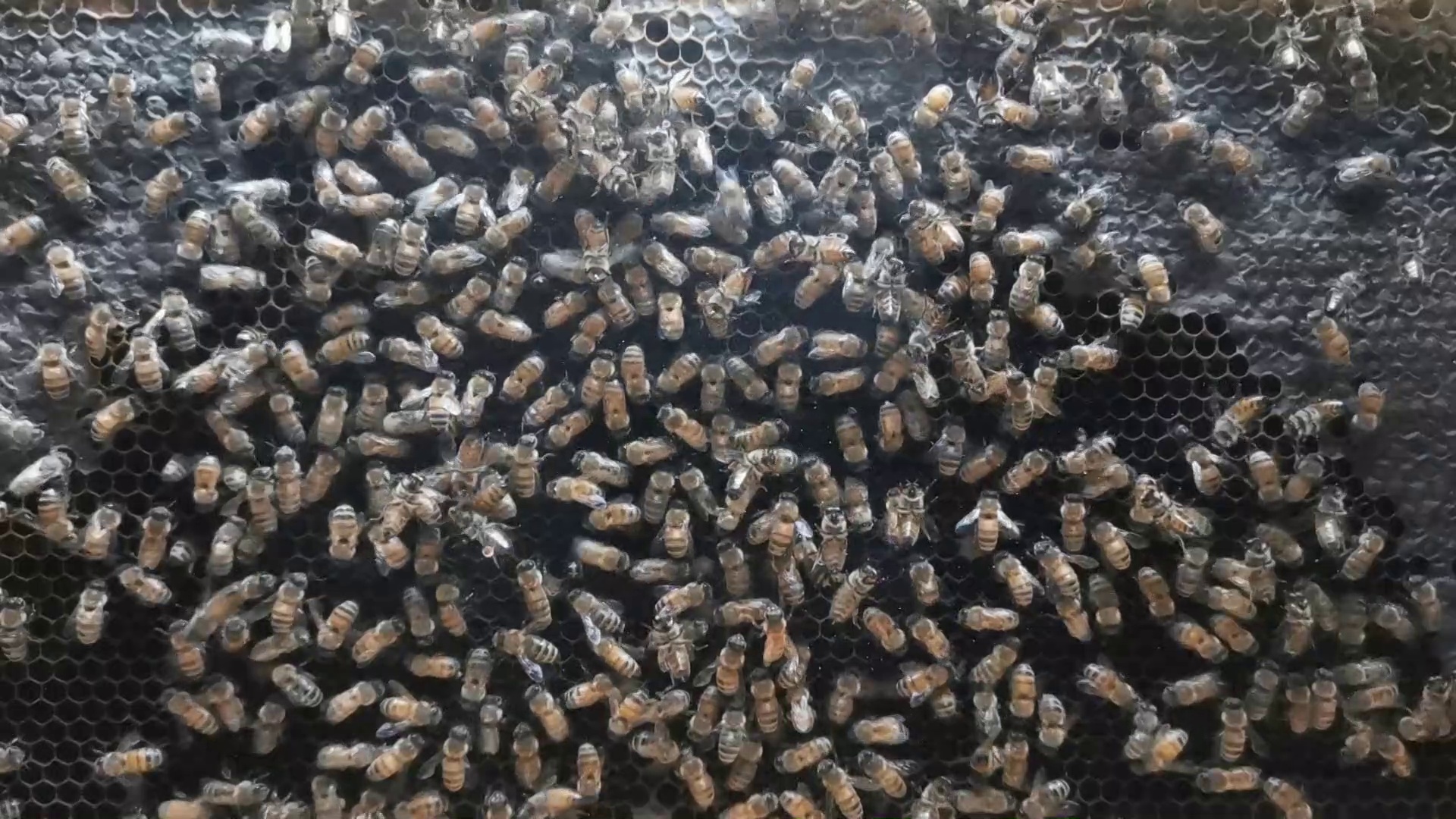

Supplement: Supplementary file 1 — Supplementary Information. [file 41598_2023_44718_MOESM1_ESM.zip › Dataset/test set-system_evaluation/test_set_15fps/090.jpg]

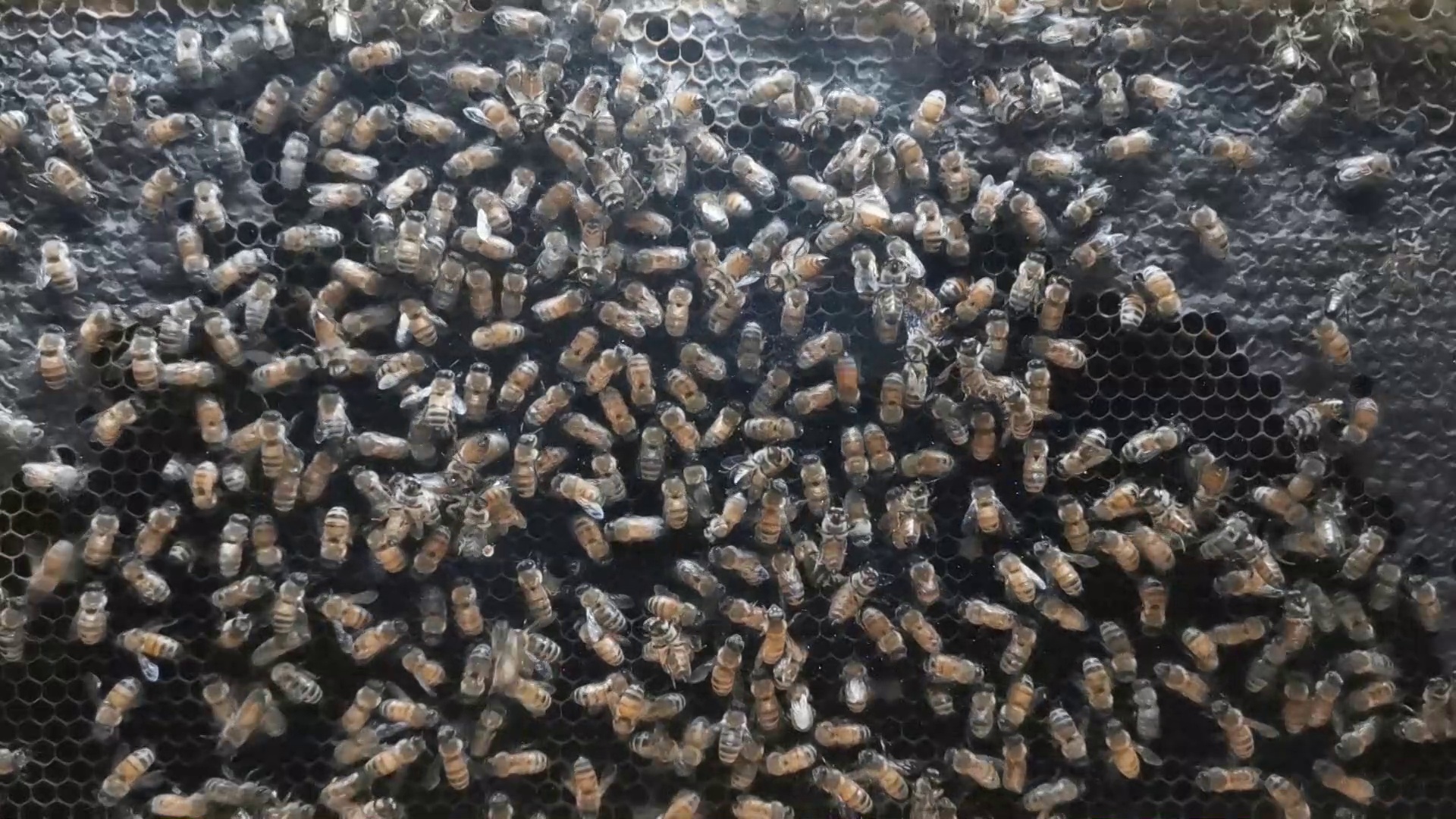

Supplement: Supplementary file 1 — Supplementary Information. [file 41598_2023_44718_MOESM1_ESM.zip › Dataset/test set-system_evaluation/test_set_15fps/111.jpg]

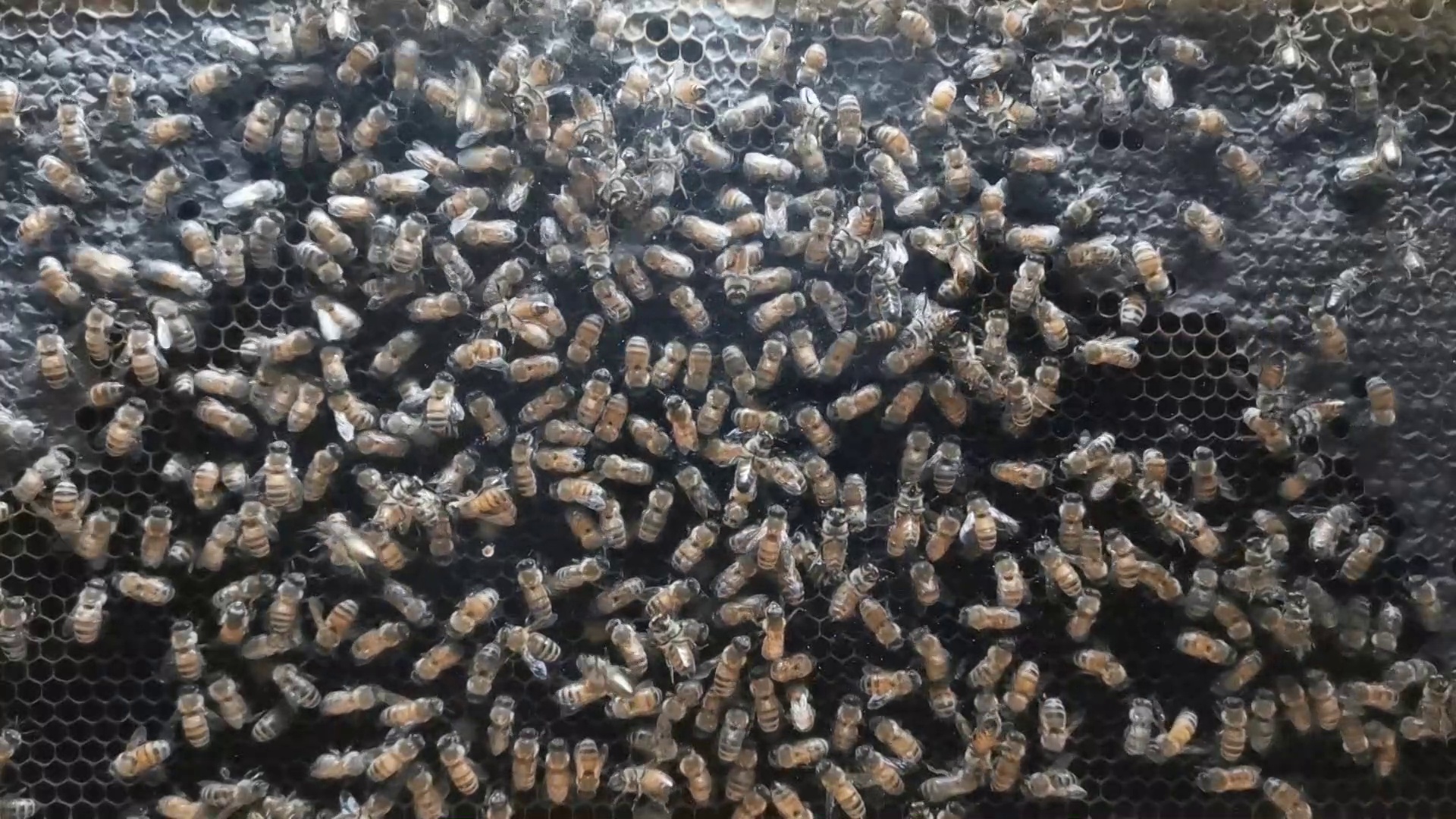

Supplement: Supplementary file 1 — Supplementary Information. [file 41598_2023_44718_MOESM1_ESM.zip › Dataset/test set-system_evaluation/test_set_15fps/071.jpg]

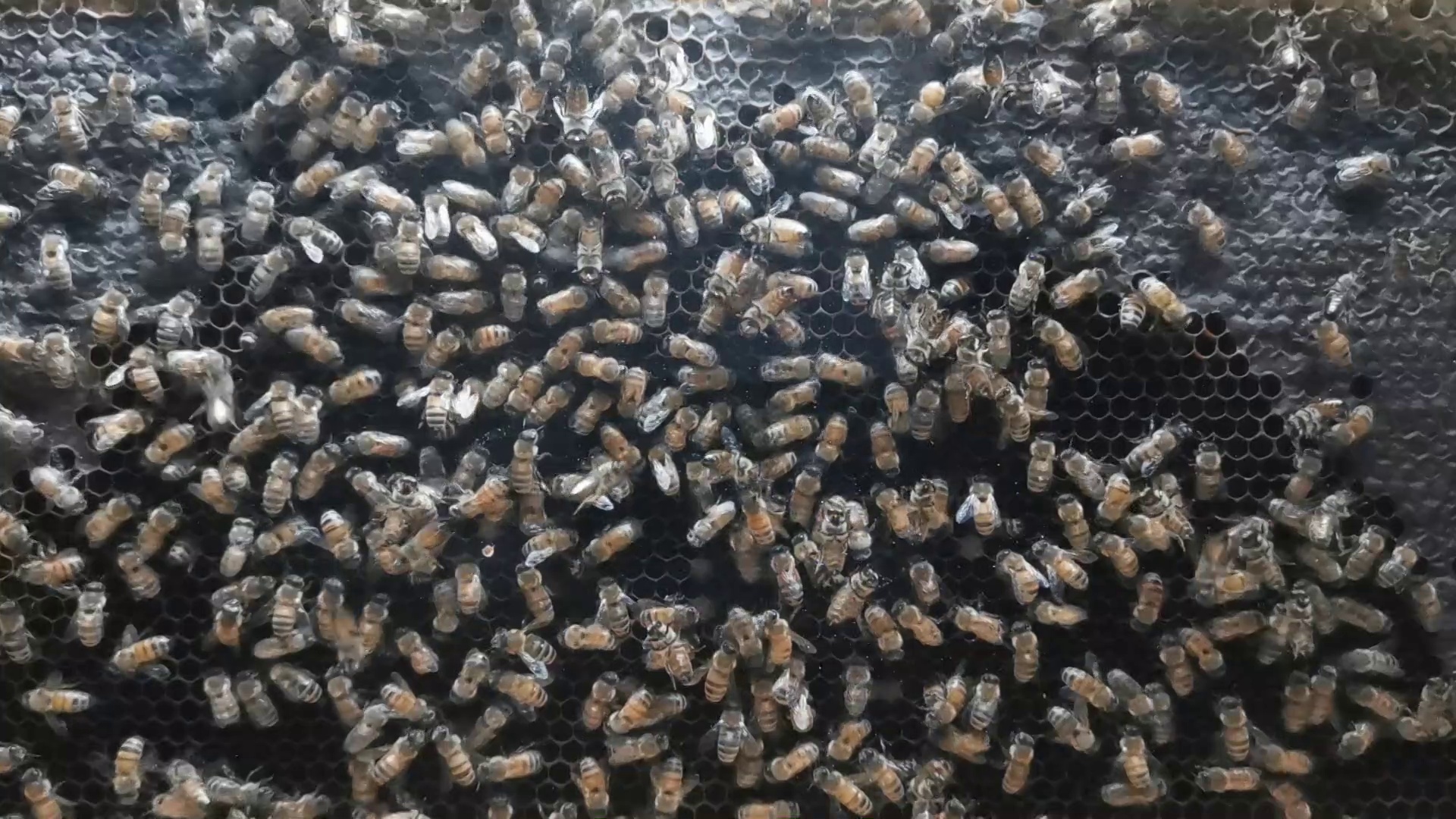

Supplement: Supplementary file 1 — Supplementary Information. [file 41598_2023_44718_MOESM1_ESM.zip › Dataset/test set-system_evaluation/test_set_15fps/131.jpg]

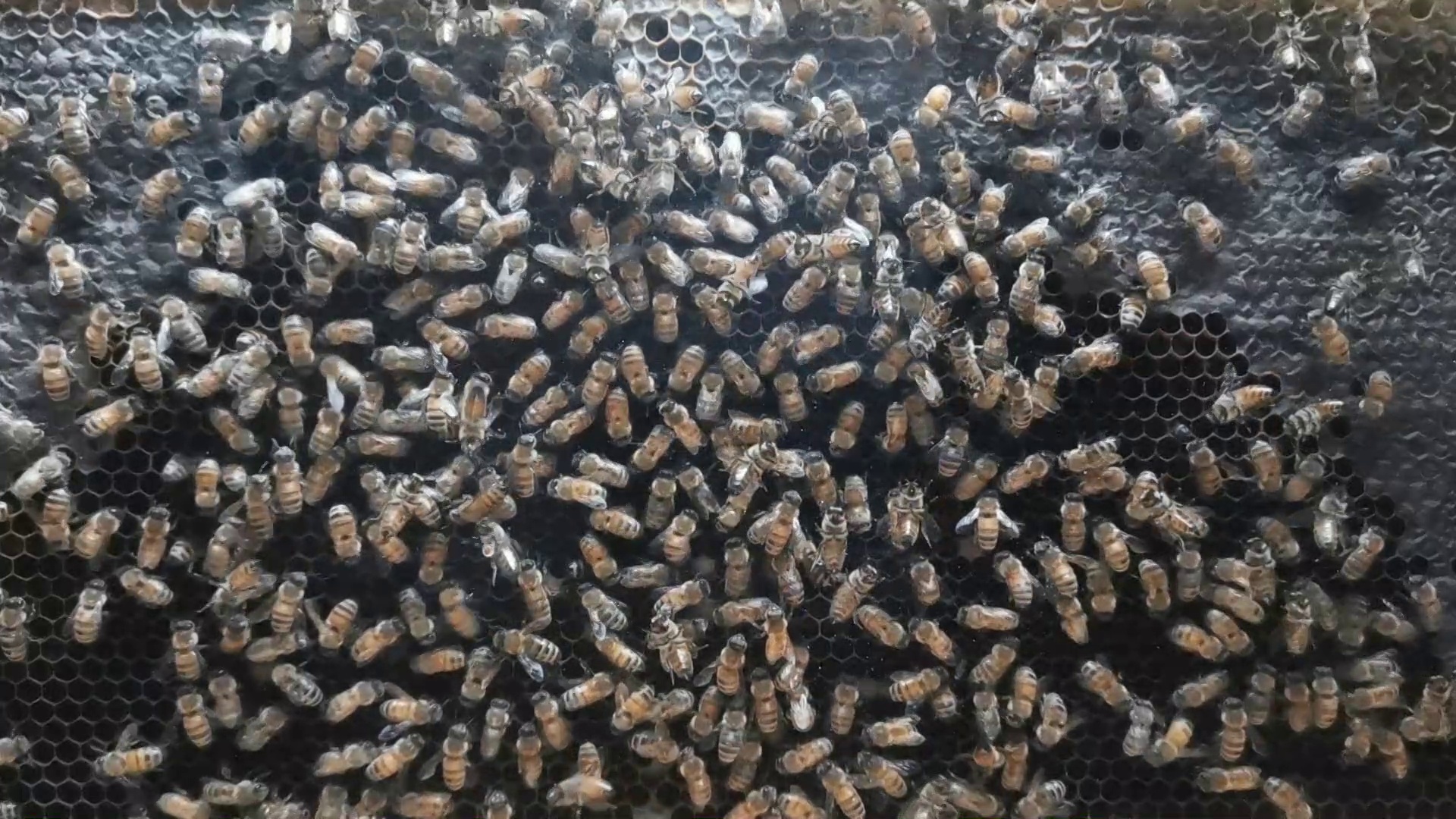

Supplement: Supplementary file 1 — Supplementary Information. [file 41598_2023_44718_MOESM1_ESM.zip › Dataset/test set-system_evaluation/test_set_15fps/086.jpg]

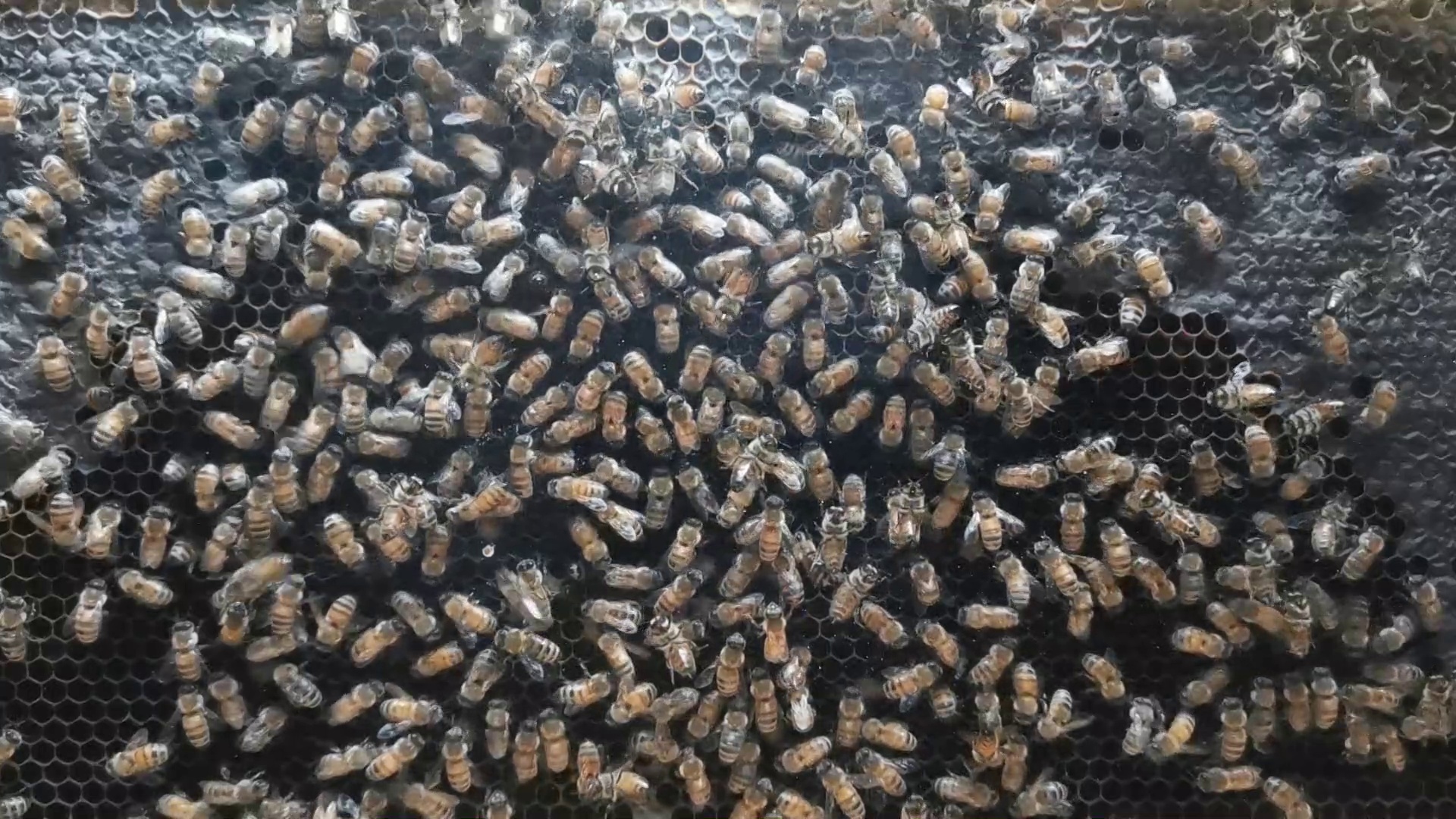

Supplement: Supplementary file 1 — Supplementary Information. [file 41598_2023_44718_MOESM1_ESM.zip › Dataset/test set-system_evaluation/test_set_15fps/080.jpg]
